# ePosters Virtual

**DOI:** 10.1111/ene.70630

**Published:** 2026-06-26

## Ageing and dementia

## EPV‐0001

### “CBI‐R BADL” composite as a tool to assess functional stages in Alzheimer's disease

#### P. Olea‐Rodríguez ^1^; M. Rodríguez‐Camacho^2^; L. Andrade‐Zumárraga^2^; E. Drzewiscki^2^; C. Barranco‐Riado^3^; M. Murcia‐Moya^3^; D. Romera‐Morales^4^; J. Arriola‐Infante^2^

##### ^*1*^ *Neurology, Torrecárdenas University Hospital, Almería, Spain;* ^*2*^ *Neurology, Cognitive Impairment Unit, Torrecárdenas University Hospital, Almería, Spain;* ^*3*^ *Nurse, Cognitive Impairment Unit, Torrecárdenas University Hospital, Almería, Spain;* ^*4*^ *Neuropsychologist, Cognitive Impairment Unit, Torrecárdenas University Hospital, Almería, Spain*

## EPV‐0002

### A novel NOTCH3 mutation in a patient with apathy and lacunar stroke: The importance of genotype‐phenotype correlations in CADASIL

#### I. Nikolakakis ^1^; A. Bacharis^1^; A. Ververi^2^; D. Parisis^1^; T. Afrantou^1^; N. Grigoriadis^1^; P. Ioannidis^1^

##### ^*1*^ *Second Department of Neurology, AHEPA University Hospital, Thessaloniki, Greece;* ^*2*^ *eDepartment of Genetics for Rare Diseases, Papageorgiou General Hospital of Thessaloniki, Thessaloniki, Greece*

## EPV‐0003

### Accelerated rise in vascular dementia being coded as a cause of death in Lithuania

#### E. Žilinskas ^1^; E. Audronytė^1^; A. Webb^2^; A. Dapkutė^1^; G. Kaubrys^1^; D. Jatužis^1^; R. Masiliūnas^1^

##### ^*1*^ *Clinic of Neurology and Neurosurgery, Institute of Clinical Medicine, Faculty of Medicine, Vilnius University, Vilnius, Lithuania;* ^*2*^ *Department of Brain Sciences, Imperial College London, UK*

## EPV‐0004

### Acceptance of fixed‐dose combination therapy with donepezil and memantine by patients and caregivers

#### L. Ontiveros Campos; C. Barranco Riado; P. Olea Rodríguez; L. Andrade Zumárraga; M. Iglesias Espinosa

##### Neurology Department, Hospital Universitario Torrecárdenas, Almería, Spain

## EPV‐0005

### AI modelling and Alzheimer's: How AI modelling coupled with biomarker findings improves pathophysiology discovery as well as diagnosis

#### E. Awodiya

##### Green Templeton College, University of Oxford, UK

## EPV‐0006

### Alcohol consumption's influence on clinical phenotype and Alzheimer's disease biomarkers

#### M. Muñoz Garcia; V. Stride‐Gonzalez; M. Gonzalez‐Sanchez; S. Llamas‐Velasco; V. Blanco‐Palmero; D. Perez‐Martinez; E. Morenas‐Rodriguez; A. Villarejo‐Galende

##### Cognitive Disorders Unit, Neurology Department, 12TH of October Hospital, Madrid, Spain

## EPV‐0007

### Alzheimer disease co‐pathology in a patient carrying pathogenic CSF1R mutation

#### G. Lombardi; V. Aprea; A. Romeo; R. Occhiuto; G. Gelosa; M. Catania; G. Di Fede; M. Ricci; M. Piccione; G. Rossi; P. Caroppo

##### Neurology 8 – Dementias and CNS Degenerative Diseases Unit, Fondazione IRCCS Istituto Neurologico Carlo Besta, Milan, Italy

## EPV‐0008

### Analytical and clinical validation of a blood‐based multiprotein biomarker panel for neurodegenerative disorders

#### G. Bentivenga ^1^; A. Mammana^2^; S. Baiardi^1^; E. Vittoriosi^1^; A. Mastrangelo^1^; E. Ruggeri^2^; M. Stanzani‐Maserati^2^; G. Rizzo^2^; R. Pantieri^2^; S. Capellari^1^; P. Parchi^1^

##### ^*1*^ *Department of Biomedical and Neuromotor Sciences (DiBiNeM), Bologna University, Bologna, Italy;* ^*2*^ *IRCCS, Istituto delle Scienze Neurologiche di Bologna, Bologna, Italy*

## EPV‐0009

### APOE ε4 and Alzheimer's disease: Effects on onset but not progression

#### Y. Dede ^1^; E. Sahin^1^; E. Erzurumluoglu^2^; Z. Guven^3^; M. Alaylıoglu^4^; G. Yılmaz Cakan^1^; S. Yoruk Oner^1^; Dursun^4^; B. Bilgic^1^; H. Hanagasi^1^; D. Gezen‐Ak^4^; S. Artan^2^; B. Samanci^1^

##### ^*1*^ *Behavioral Neurology and Movement Disorders Unit, Department of Neurology, Istanbul Faculty of Medicine, Istanbul University, Istanbul, Türkiye;* ^*2*^ *Department of Medical Genetics, Faculty of Medicine, Eskisehir Osmangazi University, Eskisehir, Türkiye;* ^*3*^ *Department of Genetics, Aziz Sancar Institute of Experimental Medicine, Istanbul University, Istanbul, Türkiye;* ^*4*^ *Brain and Neurodegenerative Disorders Research Laboratories, Department of Neuroscience, Institute of Neurological Sciences, Istanbul University‐Cerrahpasa, Istanbul, Türkiye*

## EPV‐0010

### Application of the ATN framework for routine clinical diagnosis of neurodegenerative disorders in a Spanish population

#### R. Manso Calderón; D. Gómez de la Torre Morales; M. Ravelo León; B. Rodriguez García; J. Aguilera Aguilera; J. Rodriguez Carrillo; I. Diaz Diaz; M. Luz Esteve; J. Gómez Sánchez; L. López Mesonero

##### Neurology Department, Salamanca University Hospital Complex, Salamanca, Spain

## EPV‐0011

### Association of microinfarcts, amyloid co‐pathology, and clinical phenotype with cognitive performance in Lewy body dementia

#### P. Stancu; A. Goodheart; R. Ye; S. Gomperts

##### Department of Neurology, Massachusetts General Hospital, Boston, Massachusetts, USA

## EPV‐0012

### Attention‐deficit/hyperactivity disorder as a risk factor for all‐cause dementia: A systematic review and meta‐analysis

#### A. Hegazi; H. Hussien; R. Mohamed

##### Faculty of Medicine, Mansoura University, Mansoura, Egypt

## EPV‐0013

### Attention‐enhanced deep learning for multi‐stage Alzheimer's disease detection and progression prediction: A systematic review and meta‐analysis

#### N. A. Mohammed ^1^; E. M. Soliman^2^; M. S. Sherif^3^; R. Mohamed^4^; A. Al‐Ghnaimat^5^; A. Moftah^6^; D. Abouda^7^; O. A. Khattab^8^; H. Abdelbaqi^9^

##### ^*1*^ *Biochemistry Division, Cairo University, Cairo, Egypt;* ^*2*^ *Faculty of Medicine Zagazig University, Zagazig, Egypt;* ^*3*^ *Damietta Faculty of Medicine, Al‐Azhar University, Daimetta, Egypt;* ^*4*^ *Faculty of Medicine, Alexandria University, Egypt;* ^*5*^ *Hashemite University, Jordan;* ^*6*^ *Faculty of Medicine, King Salman International University, South Sinai, Egypt;* ^*7*^ *Faculty of medicine, Alexandria university, Alexandria, Egypt;* ^*8*^ *Faculty of Science, Cairo University, Egypt;* ^*9*^ *Faculty of Medicine, Tanta University, Tanta, Egypt*

## EPV‐0014

### Attributable burden of Alzheimer's disease and other dementias due to smoking: A comparative analysis in trends across Europe (1990‐2023)

#### S. Wasti^1^; A. Wasti^2^; M. Iqbal ^1^

##### ^*1*^ *Department of Neurology, King Edward Medical University, Lahore, Pakistan;* ^*2*^ *Department of Neurology, Continental Medical College, Lahore, Pakistan*

## EPV‐0015

### Beta‐synuclein: A diagnostic and prognostic biomarker in early Alzheimer's disease

#### B. Samanci ^1^; M. Alaylioglu^2^; G. Yilmaz Cakan^1^; G. Iskan^1^; E. Sahin^1^; H. Gurvit^1^; E. Dursun^2^; B. Bilgic^1^; D. Gezen‐Ak^2^; H. Hanagasi^1^

##### ^*1*^ *Behavioral Neurology and Movement Disorders Unit, Department of Neurology, Istanbul Faculty of Medicine, Istanbul University, Istanbul, Türkiye;* ^*2*^ *Brain and Neurodegenerative Disorders Research Laboratories, Department of Neuroscience, Institute of Neurological Sciences, Istanbul University‐Cerrahpasa, Istanbul, Türkiye*

## EPV‐0016

### Biomarkers of neurodegenerative diseases

#### A. Medvedeva ^1^; N. Yakhno^2^

##### ^*1*^ *European Medical Center, Moscow, Russian Federation;* ^*2*^ *First Moscow State Medical University, Moscow, Russian Federation*

## EPV‐0017

### Cerebrospinal fluid LMO4 as a synaptic biomarker linked to Alzheimer's disease pathology and cognitive decline

#### Y. Chen; S. Li

##### Hebei Medical University, Hebei, China

## EPV‐0018

### Challenges in assessing neuropsychiatric symptoms in dementia via telemedicine: Lessons from the “TeleCogNition” project in the Aegean

#### E. Angelopoulou; I. Stamelos; J. Papatriantafyllou; D. Kontaxopoulou; K. Vourou; E. Smaragdaki; S. Papageorgiou

##### 1st Neurology Department, Aiginition University Hospital, National and Kapodistrian University of Athens, Greece

## EPV‐0019

### Structural brain ageing in the Kenya

#### C. Castelblanco Riveros ^1,2^; B. Alaka^1^; C. Bikeri^1^; A. Showkat^2^; L. Musili^1^; J. Shah^1^; J. Odhiambo^1^; J. Miano^3^; S. Ponda^3^; S. Gitau^3^; M. Saleh^4^; Z. Merali^1^; C. Ude‐Momoh^1,5^; T. Thesen^1,2^; K. Blackmon^1,2,6^

##### ^*1*^ *Aga Khan University, Brain and Mind Institute, Nairobi, Kenya;* ^*2*^ *Geisel School of Medicine at Dartmouth and Dartmouth College, Hanover, USA;* ^*3*^ *Aga Khan University, Department of Radiology, Nairobi, Kenya;* ^*4*^ *Aga Khan University Cancer Centre, Cancer Research Unit, Nairobi, Kenya;* ^*5*^ *Wake Forest University School of Medicine, North Carolina, USA;* ^*6*^ *Aga Khan University, Department of Population Health, Nairobi, Kenya*

## EPV‐0020

### Clinical criteria for primary age related tauopathy: Challenges in development and implementation

#### I. Carmo e Pinto ^1^; P. Faustino^2^; A. Silva‐Spínola^3^; M. Lima^4^; J. Durães^2^; L. Alves^1^; I. Baldeiras^3^; M. Tábuas‐Pereira^2^; I. Santana^2^

##### ^*1*^ *Neurology Department, ULS Lisboa Ocidental, Lisbon, Portugal;* ^*2*^ *Neurology Department, ULS Coimbra, Coimbra, Portugal;* ^*3*^ *Centre for Innovative Biomedicine and Biotechnology (CIBB), University of Coimbra, Coimbra, Portugal;* ^*4*^ *Centre for Research in Neuropsichology and Cognitive Behavioural Intervention, University of Coimbra, Coimbra, Portugal*

## EPV‐0021

### Clinical, electrophysiological and neuroimaging correlates of dysphagia risk in dementia

#### M. Cotta Ramusino ^1^; C. Imbimbo^1^; G. Perini^1^; S. Bernini^2^; M. Picascia^2^; L. Farina^3^; A. Pichiecchio^3^; A. Costa^1^; E. Caverzasi^3^; G. Cosentino^4^

##### ^*1*^ *Unit of Behavioral Neurology and Center for Cognitive Disorders and Dementias (CDCD), IRCCS Mondino Foundation, Pavia, Italy;* ^*2*^ *Cognitive Psychology Research Section, IRCCS Mondino Foundation, Pavia, Italy;* ^*3*^ *Advanced Imaging and Artificial Intelligence Center, IRCCS Mondino Foundation, Pavia, Italy;* ^*4*^ *Translational Neurophysiology Research Section, IRCCS Mondino Foundation, Pavia, Italy*

## EPV‐0022

### Cognitive reserve and confrontational naming in healthy aging

#### V. Čiernik Kevická ^1^; V. Čiernik Kevická^2^; Z. Fritzová^1^; P. Brandoburová^2^; P. Brandoburová^3^

##### ^*1*^ *Department of Communication Disorders, Comenius University, Bratislava, Slovakia;* ^*2*^ *MEMORY Centre, Bratislava, Slovakia;* ^*3*^ *Department of Psychology, Comenius University, Bratislava, Slovakia*

## EPV‐0023

### Data‐driven MRI patterns outperform volumetric measures for preclinical AD detection: Systematic review and meta‐analysis

#### N. A. Mohammed^1^; M. A. S. Sherif^2^; A. Al‐Ghnaimat^3^; R. Mohamed^4^; D. Atef Abouda^5^; B. Ayoub^6^; Z. Hasan ^7^; H. Abdelbaqi^8^; A. Mohamed^9^; S. M. Soliman^10^; D. Bazbaz^11^; A. Ahmed Radi^12^; Z. E. Khodary^13^

##### ^*1*^ *Biochemistry Division, Cairo University, Cairo, Egypt;* ^*2*^ *Damietta Faculty of Medicine, Al‐Azhar University, Daimetta, Egypt;* ^*3*^ *Hashemite University, Jordan;* ^*4*^ *Faculty of Medicine, Alexandria University, Egypt;* ^*5*^ *Faculty of Medicine, Alexandria University, Alexandria, Egypt;* ^*6*^ *Faculty of Medicine, Cairo University, Egypt;* ^*7*^ *Jordan University of Science and Technology Irbid, Jordan;* ^*8*^ *Faculty of Medicine, Tanta University;* ^*9*^ *MBBCh;* ^*10*^ *Flushing Hospital Medical Center;* ^*11*^ *An‐Najah National University, Palestine;* ^*12*^ *Faculty of Medicine, Minia University, Minia, Egypt;* ^*13*^ *Biophysics Department, Faculty of Science, Al‐Azhar University, Cairo, Egypt*

## EPV‐0024

### Diagnostic performance of 3D convolutional neural networks for predicting MCI‐to‐AD conversion: A systematic review and meta‐analysis

#### A. Wael Moftah ^1^; Z. E. Khodary^2^; D. Atef Abouda^3^; N. A. Mohammed^4^; Y. A. Elftah^5^; Z. Hasan^6^; E. M. Soliman^7^; A. Kertam^8^; A. Mehta^9^

##### ^*1*^ *Faculty of Medicine, King Salman International university, South Sinai, Egypt;* ^*2*^ *Biophysics Department, Faculty of Science, Al‐Azhar University, Cairo, Egypt;* ^*3*^ *Faculty of Medicine, Alexandria University, Alexandria, Egypt;* ^*4*^ *Biochemistry Division, Cairo University. Cairo, Egypt;* ^*5*^ *Special Physics Department, Faculty of Science, Al‐Azhar University, Cairo, Egypt;* ^*6*^ *Jordan University of Science and Technology, Irbid, Jordan;* ^*7*^ *Faculty of Medicine Zagazig University, Zagazig, Egypt;* ^*8*^ *Tu Lab for Diagnostic Research, Yale School of Medicine, Tompkin's East 2, New Haven, USA;* ^*9*^ *GMERS Medical College Gotri, Vadodara, Gujarat, India*

## EPV‐0025

### Diagnostic value of CSF tTau and tTau/pTau ratio in probable Creutzfeldt–Jakob disease: A Tertiary referral center cohort study

#### A. Sacramento ^1^; P. Faustino^1^; J. Durães^1^; M. Tábuas‐Pereira^1^; I. Baldeiras^2^; I. Santana^1^

##### ^*1*^ *Department of Neurology, Unidade Local de Saúde de Coimbra, Coimbra, Portugal;* ^*2*^ *Centre for Innovative Biomedicine and Biotechnology (CiBB), University of Coimbra, Coimbra, Portugal*

## EPV‐0026

### Early detection of dementia risk in CADASIL using integrated clinical, neuroimaging and immunogenetic markers

#### A. Ismatov; D. Tolibov; M. Namozov; R. Umarov; S. Mirdedaev

##### Department of Neurology, Tashkent State Medical University, Tashkent, Uzbekistan

## EPV‐0027

### Abstract withdrawn

## EPV‐0028

### Efficacy and Safety of phenserine and its enantiomer, buntanetap (Posiphen) in treatment of Alzheimer's disease, a systematic review and meta‐analysis

#### A. Shelbaya ^1^; M. Ali^2^; N. Hamam^3^; M. Elhussein^4^; A. Gado^5^; M. Fallaha^2^; A. Mohamed^6^; M. Abdelbaset^7^; A. Soliman^8^; N. Hendi^2^; M. Meshref^9^

##### ^*1*^ *Faculty of medicine, New Mansoura university, New Mansoura, Egypt;* ^*2*^ *Faculty of medicine, Ain Shams University, Cairo, Egypt;* ^*3*^ *Faculty of Medicine, Kasr Al‐Ainy, Cairo University, Cairo, Egypt;* ^*4*^ *Faculty of Medicine, University of Science and Technology, Omdurman, Sudan;* ^*5*^ *Faculty of Medicine, Tanta university, Tanta, Egypt;* ^*6*^ *Faculty Of Medicine Merit university, Sohag, Egypt;* ^*7*^ *Faculty of Medicine Zagazig University, Zagazig Egypt;* ^*8*^ *Faculty of Medicine, Newgiza University, Giza, Egypt;* ^*9*^ *Department of Neurology, Faculty of Medicine, Al‐Azhar University, Cairo, Egypt*

## EPV‐0029

### Egyptian dementia risk score: Machine learning model from EDN registry data

#### S. Heikal; A. Shoaib; M. Salama

##### The American University in Cairo, Cairo, Egypt

## EPV‐0030

### Electroencephalography as a predictive marker for early diagnosis of cognitive decline

#### V. Ignatova ^1^; L. Todorova^2^; S. Mantarova^1^

##### ^*1*^ *Clinic of Neurology, Military Medical Academy, Sofia, Bulgaria;* ^*2*^ *Institute of Biophysics and Biomedical Engineering, Bulgarian Academy of Sciences, Sofia, Bulgaria*

## EPV‐0031

### Elevated serum sodium is linked to increased amyloid‐dependent tau pathology, neurodegeneration, and cognitive impairment in Alzheimer's disease

#### Y. Chen; S. Li

##### Hebei Me.dical University, Hebei, China

## EPV‐0032

### Emotional and cognitive engagement in Alzheimer's disease during facilitated art sessions: Observational insights from a nursing home setting

#### F. Jasarevic

##### Acibadem University, School of Medicine, Istanbul, Türkiye

## EPV‐0033

### Evaluation of prescription appropriateness in hospitalized elderly patients at a neurology institute: Cross‐sectional study

#### E. Bokri ^1^; E. Jenhani^1^; M. Romdhan^1^; C. Ben Azzouz^1^; R. Zouari^2^; D. Ben Mohamed^2^; M. Saied^2^; S. Ben Sassi^2^; N. Hasni^1^

##### ^*1*^ *Department of Pharmacy, National Institute Mongi Ben Hmida of Neurologie, Tunis, Tunisia;* ^*2*^ *Department of Neurology, National Institute Mongi Ben Hmida of Neurologie, Tunis, Tunisia*

## EPV‐0034

### Examining time to diagnosis in a real‐world memory clinic cohort

#### S. Goeschl ^1^; S. Klotz^2^; S. Silvaieh^1^; T. Parvizi^1^; R. Wurm^1^; E. Berger‐Sieczkowski^1^; T. König^1^; H. Untersteiner^1^; G. Regelsberger^2^; E. Gelpi^2^; E. Stögmann^1^

##### ^*1*^ *Department of Neurology, Medical University of Vienna, Vienna, Austria;* ^*2*^ *Institute of Neurology, Medical University of Vienna, Vienna, Austria*

## EPV‐0035

### Features of functional cognitive disorder in patients with subjective cognitive complaints and mild cognitive impairment: A prospective study

#### J. Potočnik; S. Brezovar; E. Županič; S. Ibrulj; T. Rus; M. Kojović; M. Gregorič Kramberger; R. Berlot

##### Department of Neurology, University Medical Centre Ljubljana, Ljubljana, Slovenia

## EPV‐0036

### Frontotemporal dementia associated with motor neuron disease in a progranulin mutation carrier: An exceptional phenotype beyond haploinsufficiency

#### J. Román Rueda ^1^; M. Bernal Arjona^2^; E. García Roldan^2^; E. Franco Macías^3^; M. Marín Cabañas^2^

##### ^*1*^ *Internal Medicine Resident, Hospital Universitario Virgen del Rocío, Seville, Spain;* ^*2*^ *Neurologist, Cognitive Disorders Unit, Hospital Universitario Virgen del Rocío, Seville, Spain;* ^*3*^ *Head of the Cognitive Disorders Unit, Hospital Universitario Virgen del Rocío, Seville, Spain*

## EPV‐0037

### Frontotemporal dementia with C9orf72 mutation presenting with bizarre psychosis and right flail leg syndrome

#### A. Mazzonetto ^1^; G. Pigato^2^; C. Bussè^1^; S. Mozzetta^1^; D. Cecchin^3^; M. Corbetta^1^; A. Cagnin^1^

##### ^*1*^ *Department of Neuroscience (DNS), University of Padova, Padova, Italy;* ^*2*^ *Psychiatry Ward, Padova University Hospital, Padova, Italy;* ^*3*^ *Department of Medicine, Unit of Nuclear Medicine, University Hospital of Padova, Padova, Italy*

## EPV‐0038

### Hearing loss as a modifiable risk factor for Alzheimer's disease: Evidence from a Tunisian case‐control study

#### F. Cherif; A. Gharbi; Y. Abida; A. Mansouri; A. Atrous; A. Souissi; A. Gargouri; I. Kacem; R. Gouider

##### Neurology Department, LR18SP03 and Clinical Investigation Center Neurosciences and Mental Health, University Hospital Center Razi‐Mannouba, Tunis, Tunisia; Faculty of Medicine of Tunis, Tunis El Manar University, Tunisia

## EPV‐0039

### Hypersexuality and inappropriate sexual behaviors in Alzheimer's disease: A scoping review

#### Z. Bercini; M. Tondelli; A. Barbieri; N. Iacovino; C. Gallingani; R. Maramotti; D. Ballotta; G. Vinceti; S. Cossutti; C. Carbone; A. Chiari; G. Zamboni

##### Department of Biomedical, Metabolic and Neural Sciences, University of Modena and Reggio Emilia, Italy

## EPV‐0040

### Incremental value of 18F‐FDG PET and other technical investigations in the diagnosis of neurodegenerative cognitive disorders

#### M. Pannecoucke ^1^; J. Coryn^2^; M. Vander Poorten^2^; T. Van Vrekhem^1^; M. Miatton^1^; T. Van Langenhove^1^; E. Genbrugge^3^; D. Van Weehaeghe^4^; M. Scarioni^1^

##### ^*1*^ *Neurology Department, Ghent University Hospital, Ghent, Belgium;* ^*2*^ *Biomedical sciences, Ghent University, Ghent, Belgium;* ^*3*^ *Radiology Department, Ghent University Hospital, Ghent, Belgium;* ^*4*^ *Nuclear medicine Department, Ghent University Hospital, Ghent, Belgium*

## EPV‐0041

### Insulin resistance influences global cognition in patients with subjective cognitive decline and mild cognitive impairment

#### S. Mazzeo ^1^; M. Corbari^1^; A. Frijo^1^; E. Bortolin^1^; G. Bruschi^1^; A. Bombaci^1^; F. Pozzi^1^; A. Astengo^1^; E. Costa^2^; M. Filippi^3^; M. Salsone^1^

##### ^*1*^ *Neurology Unit, IRCCS Policlinico San Donato, Vita‐Salute San Raffaele University, Milan, Italy;* ^*2*^ *Service of Laboratory Medicine, IRCCS Policlinico S. Donato, Milan, Italy; Laboratory of Molecular Biology, IRCCS Policlinico San Donato, Milan, Italy;* ^*3*^ *Vita‐Salute San Raffaele University, and Neurology Unit, Neurorehabilitation Unit, and Neurophysiology Service, IRCCS Ospedale San Raffaele, Milan, Italy*

## EPV‐0042

### IN‐TeMPO: A multicenter trial of multidimensional interventions to reduce cognitive decline in older adults

#### L. Cuffaro ^1^; F. Pozzi^1^; G. Sala^1^; R. Daini^3^; F. Gasparini^12^; V. Solfrizzi^4^; F. Demarchi^5^; V. Bessi^6^; M. Bologna^7^; L. Petraglia^8^; A. Di Costanzo^9^; E. Cicero^10^; R. Monastero^11^; C. Ferrarese^1^

##### ^*1*^ *University of Milan Bicocca, Milan, Italy;* ^*3*^ *Department of Psychology, University of Milano‐Bicocca, Milan, Italy;* ^*4*^ *Department of Interdisciplinary Medicine, University of Bari Aldo Moro, Bari, Italy;* ^*5*^ *Department of Translational Medicine, University of Piemonte Orientale, Novara, Italy;* ^*6*^ *Department of Neuroscience, Careggi University Hospital, Florence, Italy;* ^*7*^ *Department of Human Neuroscience, University of Rome Sapienza, Rome, Italy;* ^*8*^ *Department of Translational Medical Sciences, Federico II University, Naples, Italy;* ^*9*^ *Department of Medicine and Health Sciences, Centre for Research and Training in Medicine of Aging, University of Molise, Campobasso, Italy;* ^*10*^ *Department G.F. Ingrassia, Section of Neurosciences, University of Catania, Catania, Italy;* ^*11*^ *Department of Biomedicine, Neuroscience and Advanced Diagnostics (BiND), University of Palermo, Palermo, Italy;* ^*12*^ *Department of Sistemic Informatics and Comunication, University of Milan Bicocca, Milan, Italy*

## EPV‐0043

### Is there a relationship between cognitive decline, surgical interventions and general anesthesia? A pilot retrospective observational study

#### C. Linguetta ^1^; A. Castiello^1^; S. Molino^1^; C. Criscuolo^2^; E. Salvatore^3^

##### ^*1*^ *Department of Neuroscience, Reproductive Sciences and Dentistry, University Federico II, Naples, Italy;* ^*2*^ *Centers for Cognitive Disorders and Dementia, Neurology University Hospital Federico II, Naples, Italy;* ^*3*^ *Department of Advanced Biomedical Sciences, University Federico II, Naples, Italy*

## EPV‐0044

### Long‐term treatment of Alzheimer's dementia with neuronavigated mechanical Shockwaves: A new effective and safe method

#### U. Sprick ^1^; A. Günes^1^; M. Beglau^1^; M. Köhne^2^

##### ^*1*^ *Department Neurostimulation, Alexius/Josef Clinic, Neuss, Germany;* ^*2*^ *Alexius/Josef Clinic, Neuss, Germany*

## EPV‐0045

### Mapping thirty years of Alzheimer's disease burden across Europe: Age‐standardized disability‐adjusted life year trends by region

#### S. Wasti^1^; A. Wasti^2^; S. Ali ^1^

##### ^*1*^ *Department of Neurology, King Edward Medical University, Lahore, Pakistan;* ^*2*^ *Department of Neurology, Continental Medical College, Lahore, Pakistan*

## EPV‐0046

### Neuroprotective potential of Mansonia gagei in a zebrafish model of Alzheimer's disease

#### M. Isac

##### Department of Biology, Faculty of Biology, Alexandru Ioan Cuza University of Iași, Iași, Romania

## EPV‐0047

### Neuropsychological assessment of a cohort with frontotemporal dementia

#### S. Lozano Veiga; R. Berbegal Serralta; E. Cañada Lahoz; A. Vieira Campos; P. Lorenzo Barreto; M. Carreras Rodríguez

##### Neurology Service, Hospital Universitario de La Princesa, Pinto, Spain

## EPV‐0048

### Neuropsychological assessment of older adults: Tools and methods, with insights from the UM6P Hospitals Geriatric Center in Benguerir (Morocco)

#### A. Azdad ^1^; L. Elbikri^2^; S. Rafik^3^; L. Tayebi^4^; K. Kinani^5^

##### ^*1*^ *Senior Psychologist & PhD Researcher, Mental Health Unit, Quality of Work Life Observatory, Mohammed VI Polytechnic University, Morocco;* ^*2*^ *Internal Medicine Physician & Clinical Research Fellow, UM6P Hospitals Geriatric Center, Faculty of Medical Sciences, Mohammed VI Polytechnic University, Benguerir, Morocco;* ^*3*^ *Physician with a geriatrics qualification, UM6P Hospitals Geriatric Center, Mohammed VI Polytechnic University, Morocco;* ^*4*^ *Internal Medicine Physician & Clinical Research Fellow, UM6P Hospitals Geriatric Center, Faculty of Medical Sciences, Mohammed VI Polytechnic University, Morocco;* ^*5*^ *Medical Director of the Hospital Facilities in the Health Care City, and Director of the Quality of Work Life Observatory, Mohammed VI Polytechnic University, Morocco*

## EPV‐0049

### Newly identified mutation in exon 8 of the PSEN1 gene: Atypical presentation of Alzheimer disease in a familial early‐onset dementia cluster

#### N. Mottola ^1^; S. Esposito^2^; M. De Stefano^2^; D. Buonanno^2^; D. Atripaldi^1^; G. Rummo^1^; A. Gallo^1^; A. Tessitore^1^; A. d'Ambrosio^1^

##### ^*1*^ *Department of Advanced Medical and Surgical Sciences, University of Campania Luigi Vanvitelli, Napoli, Italy;* ^*2*^ *I Neurology Division, AOU “Luigi Vanvitelli”, Naples, Italy*

## EPV‐0050

### NULISA synaptic biomarker signature distinguishes Alzheimer's disease from other dementias

#### C. Martinuzzo ^1^; A. Pilotto^1^; C. Tolassi^3^; M. Sauer^4^; A. Benedet^5^; A. Rondina^1^; A. Galli^1^; T. Merati^1^; C. Trasciatti^1^; I. Girotto^3^; G. di Molfetta^5^; I. Pola^5^; K. Tan^5^; W. Traichel^5^; S. Caratozzolo^1^; H. Zetterberg^5^; N. Ashton^5^; A. Padovani^1^

##### ^*1*^ *Department of Clinical and Experimental Sciences, Neurology Unit, University of Brescia, Brescia, Italy;* ^*3*^ *Neurobiorepository and Laboratory of Advanced Biological Markers, University of Brescia and ASST Spedali Civili Hospital, Brescia, Italy;* ^*4*^ *Clinical Neurochemistry Laboratory, Sahlgrenska University Hospital, Mölndal, Sweden;* ^*5*^ *Department of Psychiatry and Neurochemistry, Institute of Neuroscience & Physiology, the Sahlgrenska Academy at the University of Gothenburg, Mölndal, Sweden*

## EPV‐0051

### Oxytocin mitigates chronic sleep deprivation–Triggered apoptosis and neuroinflammation in the cerebro‐cerebellar axis of aged rats

#### M. Elsherbiny ^1^; H. Elkattawy^1^; N. Elsherbiny^2^; R. Wahid^3^; S. El‐sayed^3^; H. Henidi^4^; R. El‐Gamal^5^; S. Alshehri^6^; S. Basheeruddin Asdaq^7^; D. Elsherbini^8^

##### ^*1*^ *Department of Basic Medical Sciences, College of Medicine, AlMaarefa University, Diriyah, Riyadh, Saudi Arabia;* ^*2*^ *Department of Pharmaceutical Chemistry, Faculty of Pharmacy, University of Tabuk, Tabuk, Saudi Arabia;* ^*3*^ *Medical physiology department, Faculty of medicine, Zagazig University, Zagazig, Egypt;* ^*4*^ *Research Department, Natural and Health Sciences Research Center, Princess Nourah bint Abdulrahman University, Riyadh, Riyadh, Saudi Arabia;* ^*5*^ *Department of Medical Biochemistry and Molecular Biology, Faculty of Medicine, Mansoura University, Egypt;* ^*6*^ *Department of Pharmaceutics, College of Pharmacy, King Saud University, Riyadh, Saudi Arabia;* ^*7*^ *Department of Pharmacy Practice, College of Pharmacy, AlMaarefa University, Dariyah, Riyadh, Saudi Arabia;* ^*8*^ *Department of Clinical Laboratory Sciences, College of Applied Medical Sciences, Jouf University, Saudi Arabia*

## EPV‐0052

### Patterns of involvement on structural and functional neuroimaging in frontotemporal dementia

#### S. Lozano Veiga; R. Berbegal Serralta; E. Cañada Lahoz; A. Vieira Campos; P. Lorenzo Barreto; M. Carreras Rodríguez

##### Hospital Universitario de La Princesa, Neurology Service, Madrid, Spain

## EPV‐0053

### Plasma NfL and GFAP in the preclinical stages of neurodegenerative diseases: Insights from the UK Biobank

#### J. Buonocore

##### Neuroscience Research Center, University Magna Graecia, Catanzaro, Italy

## EPV‐0054

### Prevalence study of lewy body dementia: Clinical and laboratory analysis of a cohort from Rio de Janeiro/Brazil

#### V. Neri ^1^; V. neri^2^; V. Neri^3^; G. Souza^2^; G. Nascimento^2^; L. Fagundes^2^; L. Ribeiro^2^; D. Viana^3^; G. Licassali^3^

##### ^*1*^ *Neurology‐ Hospital da Lagoa, Rio de Janeiro, Brazil;* ^*2*^ *Neurology, Faculty of Medicine de Campos, Campos dos Goytacazes, Brazil;* ^*3*^ *Neurology‐ Alzheimer and Parkinson Disease Center, Campos dos Goytacazes, Brazil*

## EPV‐0055

### Primary progressive aphasia as the initial manifestation of hereditary diffuse leukoencephalopathy with spheroids: A case report

#### L. García‐Granados ^1^; A. Luque‐Ambrosiani^1^; I. Lopera‐Rodríguez^1^; J. Ciriero‐Macías^1^; J. Andrada‐Moreno^1^; M. de Miguel‐Escribano^2^; C. Villar‐Rodríguez^1^; M. Bernal Sánchez‐Arjona^1^

##### ^*1*^ *Department of Neurology, Hospital Universitario Virgen del Rocío, Sevilla, Spain;* ^*2*^ *Department of Internal Medicine, Hospital Universitario Virgen del Rocío, Sevilla, Spain*

## EPV‐0056

### Quantitative relationship between Lumipulse G Measures of pTau217 in plasma and in brain

#### R. Vandenberghe ^1^; S. Tomé^2^; S. De Meyer^1^; C. Lambrechts^3^; J. Goossens^3^; K. Poesen^4^; J. Vanbrabant^3^; E. Vanmechelen^3^; D. Thal^2^

##### ^*1*^ *Laboratory for Cognitive Neurology, KU Leuven, Leuven, Belgium;* ^*2*^ *Laboratory for Neuropathology, KU Leuven, Leuven, Belgium;* ^*3*^ *ADx Neurosciences, Zwijnaarde, Gent, Belgium;* ^*4*^ *Laboratory for Neurobiomarker Research, KU Leuven, Leuven, Belgium*

## EPV‐0057

### Random forest identifies stable CSF biomarkers for AD progression: Systematic review and meta‐analysis

#### N. A. Mohammed^1^; A. Wael Moftah^2^; E. M. Soliman^3^; Y. A. Elftah^4^; M. Mahmoud Fathy^5^; D. Atef Abouda^6^; A. Ahmed Radi^7^; T. Aziz El‐Ramly ^8^; H. Abdelbaqi^9^; S. M. Soliman^10^; D. Bazbaz^11^; A. Mohamed^12^; R. Mohamed^13^

##### ^*1*^ *Bachelor Degree of Science, Biochemistry Division, Cairo University, Cairo, Egypt;* ^*2*^ *Faculty of Medicine, King Salman International University, South Sinai, Egypt;* ^*3*^ *Faculty of Medicine Zagazig University, Zagazig, Egypt;* ^*4*^ *Special Physics Department, Faculty of Science, Al‐Azhar University, Cairo, Egypt;* ^*5*^ *Faculty of Medicine, Al Azhar University, New Damietta, Egypt;* ^*6*^ *Faculty of Medicine, Alexandria University, Alexandria, Egypt;* ^*7*^ *Faculty of Medicine, Minia University, Minia, Egypt;* ^*8*^ *Faculty of Medicine, Alexandria University, Alexandria, Egypt;* ^*9*^ *Faculty of Medicine, Tanta University;* ^*10*^ *Flushing Hospital Medical Center;* ^*11*^ *An‐Najah National University, Palestine;* ^*13*^ *Faculty of Medicine, Alexandria University, Egypt*

## EPV‐0058

### Real world implementation of brain health services for dementia prevention: Insights from the ICOPAD working group

#### F. Ribaldi ^1^; O. Grau‐Rivera^2^; A. Rostamzadeh^3^; F. Mangialasche^4^; G. Frisoni^1^; F. the International dBHS Network^1^

##### ^*1*^ *Geneva Memory Center, Geneva University Hospitals, Geneva, Switzerland;* ^*2*^ *Barcelonaβeta Brain Research Center (BBRC), Pasqual Maragall Foundation, Barcelona, Spain;* ^*3*^ *Department of Psychiatry, Faculty of Medicine and University Hospital Cologne, University of Cologne, Cologne, Germany;* ^*4*^ *FINGERS Brain Health Institute, Solna, Sweden*

## EPV‐0059

### Reversible Olanzapine‐induced dropped head syndrome in behavioral variant of frontotemporal dementia: A case report

#### V. Aprea; G. Lombardi; C. Villa; A. Romeo; G. Gelosa; G. Di Fede; P. Caroppo

##### 1. Neurology 8 – Dementias and CNS Degenerative Diseases Unit, Fondazione IRCCS Istituto Neurologico Carlo Besta, Milan, Italy

## EPV‐0060

### Revision of personal cases to select patients for anti‐amyloid therapy

#### M. Anselmi ^1^; C. Rais^2^; R. Di Giacopo^2^

##### ^*1*^ *Interdepartmental Centers of Medical Sciences, University of Trento, Trento, Italy;* ^*2*^ *Memory Clinic, Provincial Health Integrated University of Trento, Trento, Italy*

## EPV‐0061

### Serum neurofilament light chain differentiates subjective cognitive decline (SCD) from non‐SCD conditions in clinical practice

#### E. Sapio; F. Motolese; M. Di Giesi; S. Selvaggi; G. Massa Rolandino; I. Anzuino; A. Salvati; G. Annesi; D. Norata; F. Ursini; F. Capone; V. Di Lazzaro

##### Department of Neurology, Campus Biomedico di Roma, Italy

## EPV‐0062

### Sex‐specific associations between mild behavioral impairment and cognition according to APOE e4 in mild cognitive impairment

#### E. Angelopoulou ^1^; M. Hatzopoulou^2^; A. Despoti^2^; N. Tsinia^2^; V. Kamtsadeli^2^; M. Papadogiani^2^; V. Kyriakidis^2^; S. Papageorgiou^1^; J. Papatriantafyllou^2^

##### ^*1*^ *1st Neurology Department, Aiginition University Hospital, National and Kapodistrian University of Athens, Greece;* ^*2*^ *Third Age Day Care Center IASIS, Athens, Greece*

## EPV‐0063

### Silent pain: Altered expression in cognitive impairment

#### S. Rakusa; M. Sinkovic; M. Rakusa

##### Division of Neurology, University Medical Centre Maribor, Maribor, Slovenia

## EPV‐0064

### Six‐year retrospective analysis of sporadic Creutzfeldt–Jakob disease in a tertiary academic center

#### H. Yuksel; B. Alanko; S. Barakli; H. Bektas

##### Department of Neurology, Ankara Bilkent City Hospital, Ankara, Türkiye

## EPV‐0065

### Sleep spindle density and its correlation with cognitive performance in patients with Alzheimer's disease

#### L. Brsikian; A. Broutian; E. Fedotova; S. Illarioshkin

##### Russian Center of Neurology and Neurosciences, Moscow, Russian Federation

## EPV‐0066

### Streamlining Alzheimer's disease diagnosis: Real‐world validation of two‐cut‐off diagnostic models based on plasma p‐tau/Aβ42 ratios

#### M. Poli ^1^; C. Bonomi^1^; M. Di Donna^1^; I. Barberis^1^; E. Ginevra^1^; M. Nuccetelli^2^; S. Bernardini^2^; D. Centonze^3^; A. Martorana^1^; C. Motta^1^

##### ^*1*^ *Memory Clinic and Neurodegenerative Dementia Research Unit, Policlinico Tor Vergata, University of Rome “Tor Vergata”, Rome, Italy;* ^*2*^ *Department of Clinical Biochemistry and Molecular Biology, Policlinico Tor Vergata, University of Rome “Tor Vergata”, Rome, Italy;* ^*3*^ *IRCCS Neuromed, Pozzilli (IS), Italy*

## EPV‐0067

### Survival analysis in early‐onset Alzheimer's disease

#### J. Dinic ^1^; G. Mandic Stojmenovic^2^; E. Stefanova^2^; T. Stojkovic^2^

##### ^*1*^ *Faculty of Medicine, University of Belgrade, Belgrade, Serbia;* ^*2*^ *Center for Memory Disorders, Neurology Clinic, University Clinical Center of Serbia, Faculty of Medicine, University of Belgrade, Belgrade, Serbia*

## EPV‐0068

### Targeting perimenopausal HPO axis dyshomeostasis: A novel strategy to mitigate women's elevated Alzheimer's disease risk

#### Y. Zhao

##### Reproductive Medicine Department, Shijiazhuang People's Hospital, Hebei, China

## EPV‐0069

### The Protective role of cognitive reserve in the conversion from Mild cognitive impairment to Alzheimer's disease: A two‐year prospective study

#### D. Archetto ^1^; C. Ilardi^2^; E. Signoriello^1^; S. Bonavita^1^; C. Coppola^1^

##### ^*1*^ *Department of Advanced Medical and Surgical Sciences, University of Campania “L. Vanvitelli”, Naples, Italy;* ^*2*^ *Department of Education, Psychology and Communication, Suor Orsola Benincasa University, Naples, Italy*

## EPV‐0070

### Validation of plasma pTau217 versus cerebrospinal fluid biomarkers for the early diagnosis of Alzheimer's disease

#### M. Ruiz Perelló; F. Salazar Hernandez; M. Orgaz‐Morales; E. Conesa; M. Martínez Zarco; B. Gómez González; J. Bermejillo Barrera; D. Lopez Segura; A. Maija Savolainen; M. Lorente; A. Sancho; D. Vidal Mena; M. Ortega ortega; J. Sánchez‐Villalobos

##### Department of Neurology, Santa Lucía University Hospital, Cartagena, Spain

## EPV‐0072

### Weakly supervised CNNs overcome limited MRI annotations: Systematic review and meta‐analysis

#### A. Kertam ^1^; T. Aziz El‐Ramly^2^; N. A. Mohammed^3^; B. Ayoub^4^; A. Al‐Ghnaimat^5^; M. Mahmoud Fathy^6^; Y. A. Elftah^7^; D. Abouda^8^; A. Wael Moftah^9^; R. Mohamed^10^; A. Mehta^11^

##### ^*1*^ *Tu Lab for Diagnostic Research, Yale School of Medicine, Tompkin's East 2, New Haven, USA, Faculty of Medicine, Ain‐Shams University, Cairo, Egypt;* ^*2*^ *Faculty of Medicine, Alexandria University, Alexandria, Egypt;* ^*3*^ *Biochemistry Division, Cairo University, Cairo, Egypt;* ^*4*^ *Faculty of Medicine, Cairo University, Egypt;* ^*5*^ *Hashemite University;* ^*6*^ *Faculty of Medicine, Al Azhar university, New Damietta, Egypt;* ^*7*^ *Special Physics Department, Faculty of Science, Al‐Azhar University, Cairo, Egypt;* ^*8*^ *Faculty of Medicine, Alexandria University, Alexandria, Egypt;* ^*9*^ *Faculty of Medicine, King Salman International University, South Sinai, Egypt;* ^*10*^ *Faculty of Medicine, Alexandria University, Egypt;* ^*11*^ *GMERS Medical College Gotri, Vadodara, India*

## EPV‐0073

### Where do elderly patients with Alzheimer's disease die? A retrospective analysis

#### S. Wasti^1^; A. Wasti^2^; E. Shafquat ^1^

##### ^*1*^ *Department of Neurology, King Edward Medical University, Lahore, Pakistan;* ^*2*^ *Department of Neurology, Continental Medical College, Lahore, Pakistan*

## EPV‐0074

### YKL‐40 in cerebrospinal fluid as a neuroinflammatory marker associated with Tau pathology in Alzheimer's disease

#### V. Milošević ^1^; J. Bašić^2^; B. Djordjević^2^; T. Ristić^3^; A. Stojčev^3^

##### ^*1*^ *Clinic of Neurology, University Clinical Center Niš, Serbia;* ^*2*^ *Department of Biochemistry, Faculty of Medicine, University of Niš, Serbia;* ^*3*^ *Center for Medical and Clinical Biochemistry, University Clinical Center Niš, Serbia*

## Autonomic nervous system diseases

## EPV‐0075

### Adie–Holmes syndrome as the first manifestation of systemic disease: Report of two clinical cases

#### M. Marin; E. Campanyà; I. Saurina; C. Martinez; E. Vadillo; A. Anaya; M. Masjuan; L. Marín; D. Lopez

##### Department of Neurology, Hospital Universitari de Girona Dr. Josep Trueta, Girona, Spain

## EPV‐0076

### AIDS‐associated benign intracranial hypertension: A case report

#### H. Abbashar; A. Hussien; I. Hegazy; M. Alfatih; Mohamed; N. Adel; N. Abdalbagi; S. Adil

##### Daoud Research Group, Sudan

## EPV‐0077

### Autonomic features, stress and cortisol response in chronic cerebral ischemia compared with healthy population

#### D. Tolibov; D. Mirzaeva; S. Shokirov

##### Tashkent State Medical University, Tashkent, Uzbekistan

## EPV‐0078

### Autonomic nervous system abnormalities in Alzheimer's disease

#### C. Váraljai

##### Department of Neurosurgery and Neurointervention, Semmelweis University, Eger, Hungary

## EPV‐0079

### Autonomic regulation via dermal vibrotactile triggering of piezoelectric channels: An emerging nonmedication approach to symptom relief

#### P. Hurwitz

##### Clarity Science LLC, Narragansett, Rhode Island, USA

## EPV‐0080

### Biological factors of perinatal anxiety

#### A. Mikolajkow

##### College of Psychiatrists of Ireland, Dublin, Ireland

## EPV‐0081

### COVID‐19 aftermath: A pilot treatment protocol for autonomic dysregulation in patients with long COVID

#### D. Soare ^1^; A. Mircea^2^; C. Von Garnier^3^; C. Von Plessen^4^; M. Saubade^5^

##### ^*1*^ *“Carol Davila” University of Medicine and Pharmacy, Bucharest, Romania;* ^*2*^ *Department of Cardiology, Vaud University Hospital, Lausanne, Switzerland;* ^*3*^ *Department of Pulmonology, Vaud University Hospital, Lausanne, Switzerland;* ^*4*^ *COVID‐long Consultation, Unisanté, Lausanne, Switzerland;* ^*5*^ *Department of Prevention and Public Health, Unisanté, Lausanne, Switzerland*

## EPV‐0082

### Evaluation of the autonomic profile in Parkinsonian syndrome

#### C. Benyaiche ^1^; L. Errguig^2^

##### ^*1*^ *Medicine Department, Faculty of Medicine and Pharmacy of Rabat, Rabat, Morocco;* ^*2*^ *Clinical Neurophysiology Department, Hôpital des Spécialités de Rabat, Rabat, Morocco*

## EPV‐0083

### Harlequin syndrome: Half the face, all the clues

#### A. Pires; A. Monteiro Moreira; L. Fontão

##### Entre Douro e Vouga Local Health Unit, Santa Maria da Feira, Portugal

## EPV‐0084

### HPA‐axis dysregulation as diagnostic marker in somatoform autonomic dysfunction

#### D. Tolibov; S. Shokirov; G. Rakhimbaeva

##### Department of Neurology, Tashkent State Medical University, Tashkent, Uzbekistan

## EPV‐0085

### Idiopathic Harlequin syndrome in a police force personnel: A decade of misdiagnosis

#### P. Anshu; K. Bhoi; D. Jaiswal; D. Gupta

##### Department of General Medicine Sri Balaji Institute of Medical Sciences Raipur, Raipur, India

## EPV‐0086

### Neuroendocrine biomarkers for differential diagnosis of somatoform autonomic dysfunction

#### S. Shokirov; D. Tolibov; D. Mirzaeva

##### Tashkent State Medical University, Tashkent, Uzbekistan

## EPV‐0087

### Prefrontal rTMS as a potential route for recovery in post‐traumatic olfactory dysfunction

#### C. Di Fazio ^1^; B. Gregoric^2^; S. Palermo^2^

##### ^*1*^ *International School of Advanced Studies, University of Camerino, Camerino, Italy;* ^*2*^ *Department of Psychology, University of Turin, Turin, Italy*

## EPV‐0088

### Relation between body composition and functional state of autonomic nervous system

#### T. Dhanshetty; S. Bhardwaj

##### Department of Physiology and Pathophysiology, Medical Faculty No. 2, Uzhhorod National University, Uzhhorod, Ukraine

## EPV‐0089

### Somatoform autonomic dysfunction in women of reproductive age with iron deficiency anemia

#### F. Yusupov ^1^; R. Matmurodov^1^; X. Nurmamat^2^

##### ^*1*^ *Tashkent State Medical University, Tashkent, Uzbekistan;* ^*2*^ *Urgench State Medical Institute, Urgench, Uzbekistan*

## EPV‐0090

### Sympathetic hyperactivity in somatoform autonomic dysfunction: Salivary alpha‐amylase as a diagnostic marker

#### S. Shokirov; G. Rakhimbaeva; D. Tolibov

##### Department of Neurology, Tashkent State Medical University, Tashkent, Uzbekistan

## EPV‐0091

### When Burnout Affects the Brain: Psychovegetative dysfunction in physicians

#### M. Nurmukhamedova

##### Neurology, Child Neurology and Medical genetics, Tashkent State Medical University, Tashkent, Uzbekistan

## Cerebrovascular diseases

## EPV‐0092

### A comparative study of parenchymal lesion vs. non‐lesion presentations in cerebral venous thrombosis

#### C. Gutu ^1^; E. Sabanov^1^; M. Ursoi^1^; L. Cojocaru^2^; E. Manole^1^

##### ^*1*^ *“Nicolae Testemițanu” State University of Medicine and Pharmacy, Chisinau, Republic of Moldova;* ^*2*^ *“Diomid Gherman” Institute of Neurology and Neurosurgery, Chisinau, Republic of Moldova*

## EPV‐0093

### A new look at biomarkers of ischemic stroke

#### S. Kyranbayeva; S. Rustambekova; R. Muxtorov; F. Mallaev; S. Kalandarova

##### Tashkent State Medical University, Tashkent, Uzbekistan

## EPV‐0094

### A rare duo: Fabry disease and familial cerebral cavernomatosis

#### S. Gomes ^1^; L. Francisco^2^; R. Coutinho^3^; C. Morgado^1^; B. Campos^4^; J. Pinto^1^; J. Alves^1^

##### ^*1*^ *Neurology Department, Braga Local Health Unit, Braga, Portugal;* ^*2*^ *Neurology Department, Local Health Unit of Alto Minho, Viana do Castelo, Portugal;* ^*3*^ *Neuroradiology Department, Braga Local Health, Portugal;* ^*4*^ *Internal Medicine Department, Braga Local Health, Braga, Portugal*

## EPV‐0095

### Abdominal obesity metrics as independent predictors of stroke severity: Evidence from a population‐based cohort from Bosnia and Herzegovina

#### E. Dozic ^1^; M. Tiric‐Campara^3^; J. Djelilovic‐Vranic^1^; A. Dozic‐Sahmic^1^

##### ^*1*^ *Clinical Center University of Sarajevo, Sarajevo, Bosnia and Herzegovina;* ^*3*^ *General Hospital “Prim. Dr Abdulah Nakaš”, Sarajevo, Bosnia and Herzegovina*

## EPV‐0096

### Active malignancy as an independent predictor of long‐term mortality in cancer‐associated stroke

#### L. Alexandru ^1^; B. Raluca Stefania^2^; T. Cristina^2^

##### ^*1*^ *University and Emergency Hospital Bucharest, Department of Neurology, Bucharest, Romania;* ^*2*^ *“Carol Davila” University of Medicine and Pharmacy, Bucharest, Romania*

## EPV‐0097

### Acute ischemic cerebrovascular event following extreme breath‐hold freediving

#### Z. Gritsopoulou; S. Stasinou; A. Tsimpiktsioglou; M. Ioakeimidis; T. Doskas

##### Neurology Department, Athens Naval Hospital, Athens, Greece

## EPV‐0098

### Acute ischemic stroke in an HIV‐positive patient with syphilis: A diagnostic challenge

#### Z. Gritsopoulou; S. Stasinou; M. Ioakeimidis; T. Doskas

##### Neurology Department, Athens Naval Hospital, Athens, Greece

## EPV‐0099

### Adipokines, thyroid function and low‐grade inflammation in overweight and obese adults: implications for cardiometabolic and cerebrovascular risk

#### B. Teshayev ^1^; Z. Khalimova^2^

##### ^*1*^ *Kashkadarya Branch of Republican Specialized Scientific‐practical Medical Centre of Endocrinology named after Academician Yo.Kh.Turakulov, Karshi, Uzbekistan;* ^*2*^ *Republican specialized scientific‐Practical Medical Centre of Endocrinology named after Academician Yo.Kh. Turakulov, Karshi, Uzbekistan*

## EPV‐0100

### Age‐ and sex‐related differences in serum levels of vitamins B6, B9, and B12 in acute ischemic stroke

#### M. Asadullayev; G. Rakhimbaeva; N. Vakhabova; S. Kodirova; T. Khomidov

##### Tashkent State Medical University, Tashkent, Uzbekistan

## EPV‐0101

### Age‐specific genetic phenotypes of ischemic stroke in women of Uzbek ethnicity

#### S. Khudayarova; G. Rakhmatullayeva; A. Kadirova

##### Tashkent State Medical University, Tashkent, Uzbekistan

## EPV‐0102

### Alexia without agraphia: A rare case of cortical damage without splenial involvement

#### L. Chranioti; S. Karatzetzou; I. Koutroulou; N. Grigoriadis; T. Karapanayiotides

##### 2nd Department of Neurology, School of Medicine, Faculty of Health Sciences, Aristotle University of Thessaloniki, AHEPA University Hospital, Thessaloniki, Greece

## EPV‐0103

### Anatomical, functional and risk factor associations of domain‐specific cognitive impairment post ischemic stroke: A cross‐sectional study

#### S. Mahfouz ^1^; E. Mhanna^2^; H. Mattar^1^

##### ^*1*^ *Holy Spirit University of Kaslik, Jounieh, Lebanon;* ^*2*^ *Hôpital Avicenne, APHP, Bobigny, France*

## EPV‐0104

### Anesthetic strategy and functional outcomes after mechanical thrombectomy for acute ischemic stroke

#### A. Dębiec ^1^; J. Staszewski^1^; P. Piasecki^2^; K. Boniecka^1^; J. Winnicka^1^; A. Stępień^1^

##### ^*1*^ *Clinic of Neurology, Military Institute of Medicine‐ National Research Institute, Warsaw, Poland;* ^*2*^ *Department of Radiology, Military Institute of Medicine‐National Research Institute, Warsaw, Poland*

## EPV‐0105

### Anterior choroidal artery territory stroke: Insights from a short case series

#### B. Nitish Gopal; A. Prabhu

##### Department of Neurology, Kasturba Medical College, Manipal Academy of Higher Education, Manipal, Karnataka, India

## EPV‐0106

### Anticoagulation in cryptogenic stroke and ESUS: A network meta‐analysis

#### A. Srinivasan ^1^; P. Pitchan Velammal^2^; J. Jayavani^1^

##### ^*1*^ *Stanley Medical College, India;* ^*2*^ *Neurology, The University of Tennessee Health Science Center‐Memphis, USA*

## EPV‐0107

### Anticoagulation management in patients with perioperative ischemic stroke

#### J. Romero Ferro; J. Cortina García; A. Sánchez Asensio; L. Gil Martínez ^1^; R. Vera Lechuga; M. Matute Lozano; S. García Madrona; J. Masjuan Vallejo; A. De Felipe Mimbrera; A. Cruz Culebras

##### Department of Neurology, Hospital Ramón y Cajal, Madrid, Spain

## EPV‐0108

### Antithrombotic strategies for stroke prevention in atrial fibrillation: A meta‐analysis of contemporary evidence

#### M. Ali ^1^; M. Pandit^2^; T. Farooq^3^; A. Akram^4^; M. Kamel^5^; M. Qayyum^6^; M. Qasim^7^; A. Naseem^8^

##### ^*1*^ *Department of Molecular Medicine, University of Pavia, Pavia, Italy;* ^*2*^ *Faculty of Medicine, Medical University of Plovdiv, Plovdiv, Bulgaria;* ^*3*^ *Jinnah Sindh Medical University, Karachi, Pakistan;* ^*4*^ *Department of Pathology, Central Park Medical College, Lahore, Pakistan;* ^*5*^ *Al‐Quds University, School of Medicine, East Jerusalem, Palestine;* ^*6*^ *Northwest General Hospital and Research Center, Peshawar, Pakistan;* ^*7*^ *Allama Iqbal Medical College, Lahore, Punjab, Pakistan;* ^*8*^ *King Edward Medical University, Lahore, Pakistan*

## EPV‐0109

### Artificial intelligence–based prediction of hemorrhagic stroke risk in patients with type 2 diabetes mellitus

#### S. Akbaralieva; M. Ataniyazov; G. Rakhimbaeva

##### Tashkent State Medical University, Neurology Department, Tashkent, Uzbekistan

## EPV‐0110

### Assessment of health‐related quality of life in patients with hemorrhagic stroke using artificial intelligence

#### S. Akbaralieva; M. Ataniyazov; G. Rakhimbaeva

##### Tashkent State Medical University, Neurology Department, Tashkent, Uzbekistan

## EPV‐0111

### Association of 24‐hours change of temperature and long‐term disability in acute ischemic stroke

#### E. Maslias ^1^; D. Strambo^1^; N. Kakaletsis^2^; D. Lambrou^1^; G. Ntaios^3^; P. Michel^1^

##### ^*1*^ *Stroke Center, Neurology Service, Department of Clinical Neurosciences, Lausanne University Hospital and University of Lausanne, Lausanne, Switzerland;* ^*2*^ *Internal Medicine, AHEPA University Hospital, Aristotle University of Thessaloniki, Greece;* ^*3*^ *Department of Medicine, University of Thessaly Larissa, Greece*

## EPV‐0112

### Association of ABO blood groups with stroke etiology according to TOAST classification: A comparative study of large and small vessel atherosclerosis

#### H. Dikbas ^1^; G. Tonkaz^2^

##### ^*1*^ *Department of Neurology, Giresun University/Giresun, Türkiye;* ^*2*^ *Department of Radiology, Giresun University/Giresun, Türkiye*

## EPV‐0113

### Association of admission urea to creatinine ratio to the mortality in hemorrhagic stroke: A systematic review and meta‐analysis

#### S. Aftabi‐Yousefabad ^1^; R. Beheshti^2^; S. Hejazian^3^; A. Moradi^2^; A. Ebrahimian^2^; R. Zand^3^

##### ^*1*^ *School of Medicine, Istanbul Aydin University, Istanbul, Türkiye;* ^*2*^ *Student Research Committee, Tabriz University of Medical Sciences, Tabriz, Iran;* ^*3*^ *Department of Neurology, Penn State Neuroscience Institute, Hershey, USA*

## EPV‐0114

### Association of headache with white matter lesion severity and cognitive impairment in cerebral small vessel disease

#### V. Vershuta

##### Neurology Department, A.Ya. Kozhevnikov Clinic of Nervous System Diseases, I.M. Sechenov First Moscow State Medical University (Sechenov University), Moscow, Russian Federation

## EPV‐0115

### Association of lipoprotein(a) with atherosclerotic burden and long‐term vascular events in east Asian patients: A prospective cohort study

#### T. Kim

##### *Department of Neurology, Incheon St. Mary's Hospital, the Catholic University of Korea*, *Incheon; Korea*

## EPV‐0116

### Atraumatic acute subdural hematoma in moyamoya disease

#### A. Vătămănelu ^1^; A. Wacongne^1^; T. Parvu^1^; P. Lefevre^2^; D. Renard^1^

##### ^*1*^ *Department of Neurology, CHU Nîmes, University of Montpellier, France;* ^*2*^ *Department of Neuroradiology, CHU Montpellier, University of Montpellier, France*

## EPV‐0117

### Atrial fibrillation diagnosed after ischemic stroke: Risk factors, cardiac abnormalities, and the importance of screening

#### J. Ferreira Machado ^1^; T. Medeiros^2^; S. Tavares^2^; S. Moreira^2^; D. Fitas^2^; C. Duque^2^; M. Lima^2^; V. Tedim Cruz^1^

##### ^*1*^ *Neurology Department, Hospital Pedro Hispano, Local Health Unit of Matosinhos, Portugal;* ^*2*^ *Stroke Unit, Hospital Pedro Hispano, Local Health Unit of Matosinhos, Portugal*

## EPV‐0118

### Baseline anaemia, haemoglobin levels, and functional outcomes after mechanical thrombectomy: A systematic review and meta‐analysis

#### H. Sharma ^1^; K. Reeta^2^; A. Vohra^1^

##### ^*1*^ *MGUMST, Jaipur, Rajasthan, India;* ^*2*^ *AIIMS, New Delhi, India*

## EPV‐0119

### Bevacizumab associated PRES

#### A. Varka; D. Parissis; P. Tsianti; P. Ioannidis; N. Grigoriadis

##### 2nd Department of Neurology, AHEPA University Hospital, Aristotle University of Thessaloniki, Thessaloniki, Greece

## EPV‐0120

### Beyond admission scores: perioperative dynamics of mWFNS grade and outcomes in aneurysmal subarachnoid hemorrhage

#### A. Revurko; Y. Solodovnikova; A. Son

##### Department of Neurology and Neurosurgery, Odesa National Medical University, Odesa, Ukraine

## EPV‐0121

### Beyond contraception: Stroke as a complication of hormonal overdose

#### H. Mario; D. Renata; R. Virginia; M. César

##### Neurología. Hospital Regional Universitario de Málaga, Málaga, Spain

## EPV‐0122

### Beyond parenchymal hematoma: Prognostic impact of the modified graeb score in acute ICH

#### E. Basha; E. Ranxha; O. Taka; O. Cibuku; R. Uruci

##### Neurovascular Service, UHC “Mother Theresa” Tirana, Albania

## EPV‐0123

### Beyond reperfusion: Pre‐stroke brain vulnerability as the main determinant of long‐term cognitive outcome after ischemic stroke

#### W. Balcerzak ^1^; A. Gorzkowska^2^; A. Lasek‐Bal^2^

##### ^*1*^ *Doctoral School of Medical and Health Sciences, Medical University of Silesia, Katowice, Poland;* ^*2*^ *Department of Neurology, School of Health Sciences, Medical University of Silesia, Katowice, Poland*

## EPV‐0124

### Beyond speed: Quality‐adjusted door‐to‐needle time and functional outcome in acute ischemic stroke: A retrospective institutional cohort study

#### V. Spinei ^1^; A. Levca^1^; T. Stupac^1^; A. Grumeza^1^; O. Grosu^2^; V. Lisnic^3^

##### ^*1*^ *Emergency Care Department, Diomid Gherman Institute of Neurology and Neurosurgery, Hospital, Chisinau, Republic of Moldova;* ^*2*^ *Department of Neurology, Diomid Gherman Institute of Neurology and Neurosurgery, Hospital, Chisinau, Republic of Moldova;* ^*3*^ *Nicolae Testemitanu State University of Medicine and Pharmacy*

## EPV‐0125

### Beyond the posterior pattern: A case of atypical posterior reversible encephalopathy syndrome during hemodialysis

#### M. El Harmochi Daoud^1^; M. Muñoz Pasadas^1^; P. Gómez Ramírez ^1^; A. Sánchez Gómez^1^; A. Herrera Ortega^1^; M. Carrillo Carrillo^1^; V. Ros Escolar^1^; A. Hernández Gónzales^1^; M. Usero Ruíz^1^; A. Castellano Vicente^2^; M. Corrales Arroyo^1^; J. Vaamonde Gamo^1^

##### ^*1*^ *Hospital General Universitario Ciudad Real, Sapin;* ^*2*^ *Hospital Santa Bárbara, Puertollano, Spain*

## EPV‐0126

### Beyond the third cranial nerve: Bilateral ptosis due to central caudal nucleus involvement in midbrain stroke

#### A. Campos Villegas; L. Rivas Ramos; A. Guzmán Téllez; M. Mañez Sierra; E. Morales García

##### Hospital Virgen de la Victoria, Málaga, Spain

## EPV‐0127

### Biomarkers and recurrence risk in symptomatic intracranial atherosclerotic stenosis: A single‐centre retrospective study

#### I. Kugler ^1^; K. Antonenko^1^; K. Mettler^1^; M. Reichmuth^1^; W. Almiri^2^; N. Slavova^1^; J. Kaesmacher^2^; R. Wiest^3^; A. Hoffmann^2^; S. Fenzl^2^; P. Radojewski^3^; M. Heldner^1^

##### ^*1*^ *Department of Neurology, Inselspital, Bern University hospital and University of Bern, Bern, Switzerland;* ^*2*^ *Institute of Diagnostic and Interventional Neuroradiology, Inselspital, University Hospital and University of Bern, Bern, Switzerland;* ^*3*^ *Institute of Diagnostic and Interventional Neuroradiology, Inselspital, University Hospital and University of Bern, Bern, Switzerland and Translational Imaging Center, Sitem‐Insel, Bern, Switzerland*

## EPV‐0128

### Brazilian adaptation of the “iHELP stroke: Improving health and lifestyle programme” and feasibility study

#### P. da Cruz Peniche ^1^; O. Lennon^2^; P. Hall^2^; L. Amendoeira Almeida Lima^1^; J. Melo dos Santos Ferreira^1^; C. Danielli Coelho de Morais Faria^1^

##### ^*1*^ *Department of Physiotherapy, Universidade Federal de Minas Gerais, Belo Horizonte, Brazil;* ^*2*^ *School of Public Health, Physiotherapy and Sports Science, University College Dublin, Dublin, Ireland*

## EPV‐0129

### Bridging therapy versus endovascular thrombectomy in acute ischemic stroke: A meta‐analysis of randomized controlled trial

#### A. Jawaid; P. Kumar; D. Vibha

##### Department of Neurology, AIl India Institute of Medical Sciences, New Delhi, India

## EPV‐0130

### Burden of undiagnosed hypertension among stroke patients in Rwanda: A five‐year retrospective study

#### O. Uwishema

##### Department of Research and Education, Oli Health Magazine Organization, Kigali, Rwanda

## EPV‐0131

### Cancer and ischaemic stroke in a comprehensive stroke centre: A retrospective review

#### L. Gil Martinez; A. Sanchez Asensio; J. Cortina Garcia; J. Romero Ferro; N. Mena Garcia; M. Campos Jimenez; G. Cabañas Engeneios; R. Pastor Gonzalez; C. Matute Lozano; S. Garcia Madrona; A. de Felipe Mimbrera; A. Cruz Culebras; J. Masjuan Vallejo; R. Vera Lechuga

##### Neurology, Ramón y Cajal Hospital, Madrid, Spain

## EPV‐0132

### Cardiac abnormalities in the early ICU course after anterior communicating artery aneurysm rupture

#### D. Hnatovska; Y. Solodovnikova

##### Department of Neurology and Neurosurgery, Odesa National Medical University, Odesa, Ukraine

## EPV‐0133

### Cardioembolic stroke in the era of anticoagulation therapy (AT): A two‐year retrospective cohort study

#### M. Hernandez Garcia; D. Padilla León; S. del Águila Romero; M. Millet Oval; I. Owrang Calvo; A. Bartolomé Yumar; J. Rojo Aladro

##### Neurology Service, Hospital Universitario de Canarias, Tenerife, Spain

## EPV‐0134

### Carotid–cavernous fistula in Albania: A prospective case series on treatment modality and regression of ocular symptoms

#### V. Leka ^1^; E. Enesi^2^; A. Rroji^2^; O. Cibuku^3^; A. Agastra^1^; S. Dodaj^1^; M. Petrela^1^

##### ^*1*^ *Neuroradiology, Neurosurgery Department, American Hospital, Tirana, Albania;* ^*2*^ *Neuroradiology Department, Mother Teresa University Hospital Center, Tirana, Albania;* ^*3*^ *Stroke Unit, Mother Teresa University Hospital Center, Tirana, Albania*

## EPV‐0135

### Case report of brainstem variant of posterior reversible encephalopathy mimicking brainstem glioma

#### P. Jayadev Menon; E. Akmal; S. Gill; T. Al Mayhani

##### Department of Neurology, Royal Free Hospital, London, UK

## EPV‐0136

### Cerebellar syndrome secondary to a Guillain‐Mollaret triangle cavernous malformation mimicking a late‐onset cerebellar ataxia

#### G. Kyriakaki ^1^; A. Lefevre^2^; S. Alamowitch^1^; G. Gerschenfeld^1^; S. Ghazanfari^1^

##### ^*1*^ *Urgences Cérébro‐Vasculaires, Hopital Pitié‐Salpêtrière, GRC NOVA, Sorbonne Université, Paris, France;* ^*2*^ *Service de Neurochirurgie, Hopital Pitié‐Salpêtrière, Sorbonne Université, Paris, France*

## EPV‐0137

### Cerebral amyloid angiopathy related inflammation: A case report and a systematic review of the literature

#### A. Antoniou; K. Rizonaki; S. Loukou; M. Panourgia; E. Alexiou

##### Department of Neurology, Evaggelismos General Hospital of Athens, Greece

## EPV‐0138

### Characteristics of VA‐PICA junction aneurysm rupture in polycystic kidney disease

#### K. Yarova; Y. Solodovnikova

##### Department of Neurology and Neurosurgery, Odesa National Medical University, Odesa, Ukraine

## EPV‐0139

### Clinical and cognitive outcomes after bi‐thalamic stroke: Multicenter artery of percheron stroke registry

#### C. Meyruey

##### Neurology Division, Department of Clinical Neurosciences, Geneva University Hospital, University of Geneva, Geneva, Switzerland

## EPV‐0140

### Clinical and laboratory predictors of early hematoma expansion in spontaneous intracerebral hemorrhage: A systematic review and meta‐analysis

#### A. Elsaid

##### Faculty of Medicine Mansoura University, Mansoura, Egypt

## EPV‐0141

### Clinical and prognostic relevance of lipoproteins and their receptors in acute cerebrovascular disorders

#### T. Khomidov; M. Asadullayev; G. Rakhimbaeva; N. Vakhabova; S. Kodirova

##### Tashkent State Medical University, Tashkent, Uzbekistan

## EPV‐0142

### Clinical and radiological profile of early and late seizures after ischemic stroke

#### K. Tekaya; I. Ghachem; S. Younes

##### Department of Neurology, Taher Sfar University Hospital, Mahdia, Tunisia

## EPV‐0143

### Clinical and safety outcomes of dexmedetomidine use during mechanical thrombectomy: A meta‐analysis

#### H. Hussien; A. Hegazi; A. Elsaid

##### Faculty of Medicine, Mansoura University, Mansoura, Egypt

## EPV‐0144

### Clinical characteristics and lesion localization of organic emotional lability after ischemic stroke

#### M. Asadullayev; G. Rakhimbaeva; N. Vakhabova; T. Khomidov; F. Bokiyeva

##### Tashkent State Medical University, Tashkent, Uzbekistan

## EPV‐0145

### Clinical characteristics and prevalence of post‐stroke emotionalism: Findings from a prospective observational study

#### M. Ibodulloyeva

##### Department of Neurology, Tashkent State Medical University, Tashkent, Uzbekistan

## EPV‐0146

### Clinical features of cerebrovascular pathology and cerebral hemodynamics in patients with rheumatic disease

#### K. Samandarov ^1^; N. Inogamova^2^; S. Musayev^1^; Y. Musayeva^1^

##### ^*1*^ *Tashkent State Medical University, Tashkent, Uzbekistan;* ^*2*^ *Alfraganus University, Tashkent, Uzbekistan*

## EPV‐0147

### Clinical outcomes of mothership and drip‐and‐ship pathways in acute ischemic stroke: First‐year experience of a comprehensive stroke center

#### E. Costru‐Tasnic ^1^; A. Arabadji^2^; I. Teaca^2^; I. Preguza^1^; M. Arion^1^; O. Odainic^1^; E. Manole^2^; O. Gavriliuc^1^; V. Dolghi^1^

##### ^*1*^ *Diomid Gherman Institute of Neurology and Neurosurgery, Chisinau, Republic of Moldova;* ^*2*^ *Nicolae Testemitanu State University of Medicine and Pharmacy, Chisinau, Republic of Moldova*

## EPV‐0148

### Clinical, imaging, and outcome spectrum of intracranial atherosclerotic disease in first‐ever ischemic stroke

#### S. Sen; B. Ray; A. Biswas

##### Department of Neurology, Bangur Institute of Neurosciences, IPGMER and SSKMH, India

## EPV‐0149

### Collateral perfusion delay predicts infarct growth and futile reperfusion after thrombectomy: A retrospective CT perfusion study

#### S. Lee ^1^; H. Ki^2^; H. Roh^3^; J. Kwak^4^; J. Park^5^; H. Lee^2^; Y. Jeon^6^; S. Jung^7^; H. Yang^4^; C. Yu^4^; J. Lee^8^; H. Kim^9^

##### ^*1*^ *Department of Neurology, Daejeon St. Mary's Hospital, College of Medicine, The Catholic University of Korea, Seoul, Republic of Korea;* ^*2*^ *Department of Neurosurgery, Daejeon St. Mary's Hospital, College of Medicine, The Catholic University of Korea, Seoul, Republic of Korea;* ^*3*^ *Department of Radiology, Konkuk University Medical Center, Konkuk University School of Medicine, Seoul, Republic of Korea;* ^*4*^ *School of Electrical Engineering, Korea University, Seoul, Republic of Korea;* ^*5*^ *Department of Neurology, Konkuk University Medical Center, Konkuk University School of Medicine, Seoul, Republic of Korea;* ^*6*^ *Department of Neurosurgery, Konkuk University Medical Center, Konkuk University School of Medicine, Seoul, Republic of Korea;* ^*7*^ *DeepClue Inc. Daejeon, Republic of Korea;* ^*8*^ *Clinical Research Center, Asan Institute for Life Science, Asan Medical Center, University of Ulsan College of Medicine, Seoul, Republic of Korea;* ^*9*^ *Department of Radiology, Daejeon St. Mary's Hospital, College of Medicine, The Catholic University of Korea, Seoul, Republic of Korea*

## EPV‐0150

### Comparative effectiveness of different treatment approaches for acute ischemic stroke in patients aged 80 years and older

#### G. Sahakyan ^1^; M. Orduyan^2^; G. Hovhannisyan^1^; A. Adamyan^2^; K. Koshtoyan^2^; H. Manvelyan^1^

##### ^*1*^ *Yerevan State Medical University, Yerevan, Armenia;* ^*2*^ *Astghik Medical Center, Yerevan, Armenia*

## EPV‐0151

### Comparative efficacy and safety of DOACs versus aspirin in embolic stroke of undetermined source: A network meta‐analysis

#### P. Pitchan Velammal^1^; G. Srinivasan ^2^; J. Jayavani^2^

##### ^*1*^ *Neurology, The University of Tennessee Health Science Center‐Memphis, TN, USA;* ^*2*^ *Stanley Medical College, India*

## EPV‐0152

### COVID‐19 vaccination and the risk of stroke and thrombotic events: An umbrella review of 35 studies

#### N. Aderinto ^1^; A. Hasan^2^; M. Olalusi^3^; A. Abioye^4^; Mbah^5^; E. Motimbe^6^; O. Akinbo^7^

##### ^*1*^ *Ladoke Akintola University of Technology;* ^*2*^ *Neurology Department, University of Minnesota, Minneapolis, USA;* ^*3*^ *AMA School of Medicine, Makati, Philippines;* ^*4*^ *Alpha Specialist Hospital, Ibadan, Nigeria;* ^*5*^ *Ahmadu Bello University (ABU) Zaria, Nigeria;* ^*6*^ *University of Buea,Cameroon;* ^*7*^ *Department of Medicine, College of Health Sciences, Obafemi Awolowo University, Ile‐Ife, Osun state, Nigeria*

## EPV‐0153

### Decompressive craniectomy after intravenous thrombolysis for malignant MCA infarction: Feasibility and outcomes in a resource‐limited stroke center

#### A. Levca; S. Dorosenco; V. Spinei; T. Stupac; O. Grosu

##### Emergency Department, Diomid Gherman Institute of Neurology and Neurosurgery, Chișinău, Moldova

## EPV‐0154

### Deep medullary veins in cerebral amyloid angiopathy and hypertensive arteriopathy

#### X. Ye; S. Huang; S. Zhu

##### Department of Neurology, Tongji Hospital of Tongji Medical College, Huazhong University of Science, Wuhan, China

## EPV‐0155

### Determinants of mortality and functional outcome in spontaneous intracerebral hemorrhage: Insights from a tertiary center cohort

#### C. Conde Velasco ^1^; A. Bocero García^1^; R. De Torres Chacón^1^; E. Montero Ramirez^1^; A. Barragán Prieto^1^; A. Rincón Valencia^1^; I. Ruiz Salcedo^1^; J. Loscertales Castaños^1^; M. Gamero García^1^; A. Domínguez Mayoral^1^; L. Ruiz Bayo^1^; S. Pérez Sánchez^2^; J. Montaner Villalonga^2^

##### ^*1*^ *Department of Neurology, Hospital Virgen Macarena, Sevilla, Spain;* ^*2*^ *Grupo de Investigación Neurovascular, Instituto de Biomedicina de Sevilla, Sevilla, Spain*

## EPV‐0156

### Diagnostic and prognostic significance of MMP‐9 levels in the hyperacute phase of hemorrhagic stroke

#### S. Akbaralieva; M. Ataniyazov; G. Rakhimbaeva

##### Tashkent State Medical University, Neurology Department, Tashkent, Uzbekistan

## EPV‐0157

### Diagnostic and prognostic value of cystatin‐c levels in the hyperacute phase of hemorrhagic stroke

#### S. Akbaralieva; M. Ataniyazov; G. Rakhimbaeva

##### Tashkent State Medical University, Neurology Department, Tashkent, Uzbekistan

## EPV‐0158

### Diet‐related stroke burden in Europe: Persistent inequities and diverging trends across subregions

#### S. Wasti^1^; A. Wasti^2^; M. Iqbal ^1^

##### ^*1*^ *Department of Neurology, King Edward Medical University, Lahore, Pakistan;* ^*2*^ *Department of Neurology, Continental Medical College, Lahore, Pakistan*

## EPV‐0159

### Does patient's sex influence acute ischemic stroke clinical profile and early outcome? A single‐center RES‐Q registry analysis

#### C. Gutu ^1^; A. Dumitrascu^1^; I. Cojocaru^2^; E. Sabanov^1^; V. Dolghi^2^; O. Gavriliuc^2^; O. Odainic^2^; E. Manole^1^; E. Costru‐Tasnic^2^

##### ^*1*^ *“Nicolae Testemitanu” State University of Medicine and Pharmacy, Chisinau, Republic of Moldova;* ^*2*^ *“Diomid Gherman” Institute of Neurology and Neurosurgery, Chisinau, Republic of Moldova*

## EPV‐0160

### Drug‐coated balloon versus stent angioplasty in patients with intracranial atherosclerotic disease: A systematic review and meta‐analysis

#### A. Siam ^1^; O. Abbas^2^; N. Ahmed^3^; R. Haddad^4^; A. Siam^5^; S. Abdalsttar^1^

##### ^*1*^ *Faculty of Medicine, Cairo University, Cairo, Egypt;* ^*2*^ *Faculty of medicine, Al‐Azhar University, Cairo, Egypt;* ^*3*^ *Faculty of Medicine, Beni‐Suef university, Beni‐Suef, Egypt;* ^*4*^ *Faculty of Medicine, October 6 University, Egypt;* ^*5*^ *Faculty of science, Ain Shams University, Egypt*

## EPV‐0161

### Dual antiplatelet therapy after peripheral revascularisation: Implications for stroke and neurovascular risk—A systematic review & meta‐analysis

#### M. Ali ^1^; M. Pandit^2^; T. Farooq^3^; S. Abbas^3^; A. Tariq^4^; A. Asif^3^; A. Javed^3^; E. Siddiqui^3^; S. Khan^5^; M. Hemida^6^; M. Alamin^7^; Z. Shahriar^8^

##### ^*1*^ *Department of Molecular Medicine, University of Pavia, Pavia, Italy;* ^*2*^ *Faculty of Medicine, Medical University of Plovdiv, Plovdiv, Bulgaria;* ^*3*^ *Jinnah Sindh Medical University, Karachi, Pakistan;* ^*4*^ *Rawalpindi Medical University, Rawalpindi, Pakistan;* ^*5*^ *Department of Medicine and Surgery, Batterjee Medical College, Jeddah, Saudi Arabia;* ^*6*^ *Alexandria Faculty of Medicine, Alexandria, Egypt;* ^*7*^ *International University of Africa, Faculty of Medicine, Khartoum, Sudan;* ^*8*^ *Dhaka Medical College and Hospital (DMCH), Dhaka, Bangladesh*

## EPV‐0162

### Early LADDER strategy (low‐dose anticoagulation and directed dose escalation with reassessment) in patients with intra‐parenchymal haemorrhage and AF

#### R. Ramesh; Y. Kanagavel; L. Narasimhan R; S. Shanmugam; P. Hazeena; A. Mekala

##### Department of Neurology, Sri Ramachandra Institute of Higher Education and Research, Chennai, India

## EPV‐0163

### Effectiveness of stabilometric platform training in balance recovery in patients with vertebrobasilar insufficiency

#### M. Ismoilova ^1^; Y. Madjidova^2^; O. Kim^3^

##### ^*1*^ *Tashkent State Medical University, Tashkent, Uzbekistan;* ^*2*^ *Tashkent State Medical University, Tashkent, Uzbekistan;* ^*3*^ *Tashkent State Medical University, Tashkent, Uzbekistan*

## EPV‐0164

### Efficacy and safety of PCSK9 inhibitors in acute ischemic stroke: A systematic review and meta‐analysis

#### A. Hegazi; R. Mohamed; H. Hussien

##### Faculty of Medicine, Mansoura University, Mansoura, Egypt

## EPV‐0165

### Endothelial and metabolic markers across ischaemic stroke subtypes and functional outcome

#### K. Abdurakhmonova ^1^; G. Rakhimbaeva^1^; D. Najmutdinova^2^

##### ^*1*^ *Department of Neurology, Tashkent State Medical University, Tashkent, Uzbekistan;* ^*2*^ *Department of Internal Medicine and Endocrinology No. 2, Tashkent State Medical University, Tashkent, Uzbekistan*

## EPV‐0166

### Endovascular treatment for severe symptomatic stenosis of the petrocavernous segment of the internal carotid artery

#### C. Kang

##### Department of Neurology, Jeju National University Hospital, Jeju National University School of Medicine, Jeju, Republic of Korea

## EPV‐0167

### Etiologic patterns and prognostic determinants of ischemic stroke in young adults at a secondary care hospital

#### B. Meira; G. Bonifácio; R. Matos; A. Militão

##### Neurology, Hospital de São Bernardo, ULS Arrábida, Setúbal, Portugal

## EPV‐0168

### Evaluation of the Effectiveness of Safe Nutrition Program Applied to Acute Stroke Patients with Dysphagia

#### A. Cavusoglu ^1^; Z. Tulek^2^; E. Ekizoglu^3^

##### ^*1*^ *Istanbul Kültür University, Faculty of Health Sciences, Department of Nursing, Istanbul, Türkiye;* ^*2*^ *İstanbul University‐Cerrahpaşa, Florence Nightingale Faculty of Nursing, Istanbul, Türkiye;* ^*3*^ *Istanbul University, Istanbul Faculty of Medicine, Department of Neurology, Istanbul, Türkiye*

## EPV‐0169

### Factors influencing the occurrence and development of cerebrospinal fluid–vascular distension in children

#### L. Ziyakhodzhaeva; N. Hasanova

##### Tashkent State Medical University, Tashkent, Uzbekistan

## EPV‐0170

### From diplopia to recurrent stroke: A diagnostic journey towards primary CNS vasculitis

#### J. Aguilera Aguilera; B. Rodriguez Garcia; M. Ravelo León; J. Rodriguez Carrillo; M. Luz Esteve; Á. Criado Antón; L. Redondo Robles; J. Vizcaya Gaona

##### Salamanca University Hospital, Salamanca, Spain

## EPV‐0171

### From guidelines to practice: Acute care bundles in spontaneous intracerebral hemorrhage

#### P. Tavares de Almeida ^1^; L. Paredes^2^; H. Costa^1^; M. Veloso^1^; M. Rocha^1^; J. Novo^1^; T. Gregório^2^; P. Barros^1^

##### ^*1*^ *Stroke Unit, Neurology Department, Unidade Local de Saúde de Gaia e Espinho, Portugal;* ^*2*^ *Internal Medicine Department, Unidade Local de Saúde de Gaia e Espinho, Portugal*

## EPV‐0172

### From hyperthyroidism to transient ischemic attack

#### J. Vrbica; A. Bajunović; T. Rabi Žikić; S. Simić; Ž. Živanović

##### Neurology Clinic, University Clinical Centre of Vojvodina, Novi Sad, Serbia

## EPV‐0173

### Functional outcome in intracerebral hemorrhage: Predictive factors

#### K. Yessine; N. Salma; J. Emna; S. Houssem; H. Anis; B. Sana

##### Neurology Department, Sahloul Hospital, Tunisia

## EPV‐0174

### Greek word intelligibility test for neurologically impaired dysarthric patients

#### V. Liaki; F. Astroullaki; K. Konstantopoulos

##### Department of Speech and Language Therapy, University of the Peloponnese, Kalamata, Greece

## EPV‐0175

### Guideline recommendations vs real‐world practice: Oral anticoagulation at discharge after ischemic stroke in Armenia

#### G. Buniatyan ^2^; M. Asatryan^1^; A. Meliksetyan^1^; N. Nazinyan^1^; M. Balasanyan^1^; M. Ghambaryan^1^; H. Sargssian^1^; L. Barseghyan^1^; L. Madoyan^1^; O. Ezoyan^1^; N. Danielyan^1^; M. Margaryan^1^; A. Shiroyan^1^; I. Shadyan^1^; M. Grigorian^1^; N. Grigoryan^1^; A. Manukyan^2^; A. Vardanian^2^; N. Yeghiazaryan^1^

##### ^*1*^ *Erebouni Medical Center, Yerevan, Armenia;* ^*2*^ *Department of Neurology and Neurosurgery, National Institute of Health, Yerevan, Armenia*

## EPV‐0176

### Headache with multiple cranial nerve palsies: Proximal carotid dissection or transient perivascular inflammation of the carotid syndrome?

#### D. Rivero‐Rodríguez; A. Diezma Maritin; M. Martin martin; R. Rodriguez Garcia; N. Muñoz Pinto; F. Marquez Peñuela; D. Maroto Navas; J. Piernagorda Copado; C. Marsal Alonso; M. Morales Casado

##### Neurology department, Complejo Hospitalario Universitario de Toledo, Spanish Ministry of Health, Toledo, Spain

## EPV‐0177

### Hematological inflammatory indices in the early phase of acute ischemic stroke

#### M. Yakubova; M. Ikromova

##### Tashkent State Medical University, Department of Neurology and Medical Psychology, Uzbekistan

## EPV‐0178

### High‐dose sildenafil use as a potential trigger for cerebral venous sinus thrombosis: A case report

#### A. Toncoglaz ^1^; I. Cojocaru^2^; G. Corcea^2^; M. Sangheli^1^

##### ^*1*^ *Department of Neurology No. 1, Nicolae Testemitanu State University of Medicine and Pharmacy, Chisinau, Republic of Moldova;* ^*2*^ *Diomid Gherman Institute of Neurology and Neurosurgery, Chisinau, Republic of Moldova*

## EPV‐0179

### Higher frequency of cortical involvement in diffusion‐weighted imaging‐positive TIA patients

#### D. Moon; J. Park; S. Heo

##### Department of Neurology, Kyung Hee University Hospital, Seoul, Republic of Korea

## EPV‐0180

### Hormonal determinants and female sex‐specific risks in cerebral venous thrombosis

#### J. Oliveira; A. Morgadinho; T. Martins; L. Pereira; M. Rodrigues

##### Department of Neurology, Local Health Unit Alamada‐Seixal, Almada, Portugal

## EPV‐0181

### Idarucizumab for dabigatran reversal: A 10‐year single‐centre experience

#### T. Nožić Škerjanec; U. Čepin; M. Simerl Jožef; A. Trebše; M. Zupan; S. Frol

##### University Medical Centre Ljubljana, Department of Neurology, Ljubljana, Slovenia

## EPV‐0182

### Identification of ITGAM and TNFSF13B as core panoptosis genes in ischemic stroke and prediction of potential therapeutic drugs

#### R. Qin

##### Department of Neurology, The First Affiliated Hospital of Guangxi Medical University, China

## EPV‐0183

### Identifying diagnostic and treatment challenges in cerebral amyloid angiopathy: Results from a multicenter Bulgarian survey

#### M. Peycheva ^1^; T. Zdravkova^2^; G. Slavov^1^; D. Zlatareva^3^; Z. Harizanova^4^; V. Valkov^1^; N. Rifino^5^; B. Storti^5^; A. Bersano^5^

##### ^*1*^ *Department of Neurology, Medical University of Plovdiv, Plovdiv, Bulgaria;* ^*2*^ *Research Institute, Medical University of Plovdiv, Plovdiv, Bulgaria;* ^*3*^ *Department of Diagnostic Imaging, Medical University of Sofia, Sofia, Bulgaria;* ^*4*^ *Department of Anatomy, Histology and Embryology, Medical University of Plovdiv, Plovdiv, Bulgaria;* ^*5*^ *Neurological Institute Carlo Besta, Milan, Italy*

## EPV‐0184

### Imaging the skull–brain immune axis: Calvarial marrow asymmetry on MRI After thrombectomy and clinical outcomes

#### N. Liao ^1^; S. Chen^2^

##### ^*1*^ *Neurology Department, Taichung Veterans General Hospital, Taichung, Taiwan;* ^*2*^ *Institute of Clinical Medicine, National Yang Ming Chiao Tung University, Taipei, Taiwan*

## EPV‐0185

### Immune TTP presenting with acute dysphasia and hemiparesis: A case report

#### A. Bajunović; J. Vrbica; Ž. Živanović; T. Rabi Žikić; S. Simić

##### Neurology Clinic, University Clinical Centre of Vojvodina, Novi Sad, Serbia

## EPV‐0186

### Impact of hematoma volume and localization on clinical prognosis in intracerebral hemorrhage

#### S. Akbaralieva; M. Ataniyazov; G. Rakhimbaeva

##### Tashkent State Medical University, Neurology Department, Tashkent, Uzbekistan

## EPV‐0187

### Impact of intravesical gentamicin irrigation on catheter‐associated urinary tract infections in a comprehensive stroke unit

#### A. Dine; O. Çibuku; E. Ranxha

##### Neuro vascular Service, University Hospital “Mother Teresa” Tirane, Albania

## EPV‐0188

### Impact of reperfusion timing on interictal epileptiform discharges after ischemic stroke

#### M. Malesani ^1^; G. Furlanis^1^; M. Ajcevic^2^; K. Iscra^2^; E. Ricci^1^; E. Vincis^1^; Q. Magda^1^; P. Caruso^1^; M. Naccarato^1^; P. Manganotti^1^

##### ^*1*^ *University of Trieste, Clinical Unit of Neurology, Trieste, Italy;* ^*2*^ *University of Trieste, Department of Engineering and Architecture, Trieste, Italy*

## EPV‐0189

### Impact of the socioeconomic crisis on acute ischemic stroke management and outcomes in a university hospital in Lebanon

#### M. Ibrahim; E. Harrouk; H. Abboud; J. El Helou; C. Matta; K. Abou Khaled

##### Hôtel Dieu de France, Université Saint Joseph, Beirut, Lebanon

## EPV‐0190

### In lacunar stroke patients, high levels of HbA1c were associated with high burden of cerebral microbleeds and white matter hyperintensities

#### H. Park^1^; S. Jeong ^2^; J. Park^3^

##### ^*1*^ *DInha University Hospital, Neurology, Incheon, South Korea;* ^*2*^ *Dong‐Guk University Hospital, Neurology, Goyang, South Korea;* ^*3*^ *Catholic University of Korea Eunpyeong St. Mary's Hospital, Republic of Korea*

## EPV‐0191

### In stroke patients, women are associates with more worsening of aortic atheroma compared with men

#### H. Park ^1^; J. Cha^2^; J. Park^3^

##### ^*1*^ *Inha University Hospital, Incheon, Korea, Republic of Korea;* ^*2*^ *Dong‐A University Hospital, South Korea;* ^*3*^ *Catholic University of Korea Eunpyeong St. Mary's Hospital, South Korea*

## EPV‐0192

### Inflammation and immune mechanisms in Moyamoya disease: A literature review

#### F. Musa; Ademeta

##### University of Novi Sad, Novi Sad, Serbia

## EPV‐0193

### Influence of admission urea to creatinine ratio in ischemic stroke patients’ neurological outcomes: A systematic review and meta‐analysis

#### R. Beheshti ^1^; S. Hejazian^2^; A. Moradi^1^; A. Ebrahimian^1^; S. Aftabi‐Yousefabad^3^; R. Zand^2^

##### ^*1*^ *Student Research Committee, Tabriz University of Medical Sciences, Tabriz, Iran;* ^*2*^ *Department of Neurology, Penn State Neuroscience Institute, Hershey, PA, USA;* ^*3*^ *School of Medicine, Istanbul Aydin University, Istanbul, Türkiye*

## EPV‐0194

### Integrated lipid, cardiac, and hematologic biomarker ratios and their association with incident stroke in a high cardiovascular‐risk cohort

#### A. Liatsos; A. Papazoglou; D. Moysidis; A. Bekiaridou; A. Samaras; G. Giannakoulas; C. StudyGroup

##### 1. 1st Department of Cardiology, University General Hospital of Thessaloniki AHEPA, Thessaloniki, Greece

## EPV‐0195

### Integrating artificial intelligence with clinical and neuroimaging data for lacunar ischemic stroke diagnosis

#### M. Mirjalolkhonov; N. Vakhabova; G. Rakhimbaeva; Y. Musaeva

##### Neurology, Tashkent State Medical University, Tashkent, Uzbekistan

## EPV‐0196

### Interleukin‐6 (IL‐6) signalling pathway modulation in the acute and sub‐acute phases of ischaemic stroke for maximising therapeutic benefits

#### B. Natarajan; J. Kattoju

##### SVIMS‐Sri Padmavathi Medical College for Women, Tirupati, India

## EPV‐0197

### Interplay between arterial collateral circulation and venous hemodynamic patterns in atherosclerotic ischemic stroke

#### F. Mallaev; G. Rakhimbayeva; S. Shokirov

##### Tashkent State Medical University, Tashkent Uzbekistan

## EPV‐0198

### Intracerebral haemorrhage in Malta: Epidemiology, risk factors and current management practices since March 2024

#### M. Miruzzi; M. Mallia

##### Department of Neurosciences, Mater Dei Hospital, Msida, Malta

## EPV‐0199

### Intracerebral haemorrhage location and intensive care unit mortality: A MIMIC‐IV Cohort study

#### S. Biswas^1^; Y. Srivastava ^1^; S. Vasireddy^2^; J. Rajamani^3^; K. Jindal^5^; A. Jayakumar^6^

##### ^*1*^ *Department of Internal Medicine, Ivano‐Frankivsk National Medical University, Ivano‐Frankivsk, Ukraine;* ^*2*^ *Department of Neurology, NMC Specialty Hospital, Abu Dhabi, United Arab Emirates;* ^*3*^ *Department of Internal Medicine, University of Debrecen, Hungary;* ^*5*^ *Department of Internal Medicine Government Medical College, Patiala;* ^*6*^ *Department of General Medicine, Sri Ramachandra Medical College, Chennai, India*

## EPV‐0200

### Intravenous thrombolysis with rt‐PA in patients with acute ischemic stroke: Clinical experience from two Egyptian centers

#### E. Abed

##### Neurology Department, Faculty of medicine, Al‐Azhar University, Cairo, Egypt

## EPV‐0201

### Intravenous usage of Tenecteplase (TNK) versus Alteplase (rt‐PA) before mechanical thrombectomy for Acute Ischemic Stroke: A systematic review

#### M. Bondan Misaghi ^1^; L. Souza Guerrer^1^; L. Marques Bigliazzi^1^; R. Marques Mendes^2^; E. Boldori Vanz^1^; M. Sperotto Barth^1^; L. Frigo Roos^1^

##### ^*1*^ *Medicine, Universidade da Região de Joinville, Joinville, Brazil;* ^*2*^ *Neurology, Hospital Governador Celso Ramos, Florianópolis, Brazil*

## EPV‐0202

### Ischemic stroke due to atrial fibrillation and oral anticoagulation: A two‐decade longitudinal study

#### J. Oliveira ^1^; T. Martins^1^; I. Domingues^2^; P. Pereira^1^; M. Grunho^1^; M. Rodrigues^1^; L. Pereira^1^

##### ^*1*^ *Stroke Unit/Neurology Department, Hospital Garcia de Orta, Unidade Local de Saúde de Almada‐Seixal, Portugal;* ^*2*^ *Internal Medicine Department, Hospital Garcia de Orta, Unidade Local de Saúde de Almada‐Seixal, Portugal*

## EPV‐0203

### Ischemic stroke in hypereosinophilic syndrome: A case of cryptogenic stroke and multidisciplinary management

#### F. Calayır ^1^; L. Mammadlı^1^; D. Acar^1^; S. Men^2^; İ. Şengün^1^; S. Canbaz Kabay^1^

##### ^*1*^ *Dokuz Eylül University, Izmir, Türkiye;* ^*2*^ *Department of Radiology, Dokuz Eylül University, Izmir, Türkiye*

## EPV‐0204

### Ischemic stroke of the left hemisphere caused by tandem occlusion treated with mechanical thrombectomy and tandem stenting

#### P. Sobieszak‐Skura; M. Świtońska; W. Skura

##### Clinic of Neurology and Neurophysiology, University Hospital No. 2 in Bydgoszcz, Poland

## EPV‐0205

### Knowledge of cerebrovascular disease among patients aged 40–65 years in Eastern Poland

#### D. Jakubowicz‐Lachowska; M. Bargielski; M. Bazylewicz; M. Wierciński; E. Szyłak; M. Chorazy; A. Kułakowska

##### Department of Neurology, Medical University of Bialystok, Poland

## EPV‐0206

### Laboratory markers of plasma coagulation, platelet function and endothelial dysfunction in patients with cerebral small vessel disease

#### D. Khutorov; O. Tikhomirova; O. Startseva; N. Zybina

##### Clinical Neurology Department, The Nikiforov Russian Center of Emergency and Radiation Medicine, Saint‐Petersburg, Russian Federation

## EPV‐0207

### Late carotid in‐stent thrombosis causing ischemic stroke treated with intravenous thrombolysis

#### R. Mesa Martínez; J. Tejada García; L. García Tuñón Villaluenga

##### Neurology, Complejo Asistencial Universitario de León (CAULE), León, Spain

## EPV‐0208

### Limb‐shaking transient ischemic attacks due to severe middle cerebral artery stenosis: A case report

#### B. Çelik ^1^; G. Pek^1^; İ. Koç^1^; S. Ünal^2^; M. Sorgun^1^; C. Togay Işıkay^1^

##### ^*1*^ *Ankara University, Faculty of Medicine, Department of Neurology, Türkiye;* ^*2*^ *Ankara University, Faculty of Medicine, Department of Radiology, Türkiye*

## EPV‐0209

### Linguistic disparities in stroke care and recovery among long‐term care residents – A population‐based cohort study

#### W. Kim ^1^; W. Li^2^; M. Gibb^2^; C. Lee^3^; R. Talarico^2^; V. Taler^4^; L. Bjerre^1^; S. Jonhston^1^; S. Karunananthan^4^; C. Backman^1^; P. Tanuseputro^5^; M. Muray^1^

##### ^*1*^ *University of Ottawa, Ottawa, Canada;* ^*2*^ *Ottawa Hospital Research Institute, Ottawa, Canada;* ^*3*^ *ICES, Toronto, Canada;* ^*4*^ *Bruyère Health Research Institute, Ottawa, Ontario, Canada;* ^*5*^ *University of Hong Kong, Hong Kong*

## EPV‐0210

### Machine learning in stroke triage and workflow optimization for endovascular thrombectomy: A systematic review

#### S. Wasti^1^; F. Amin^1^; A. Wasti^2^; S. Chattha ^1^

##### ^*1*^ *Department of Neurology, King Edward Medical University, Lahore, Pakistan;* ^*2*^ *Department of Neurology, Continental Medical College, Lahore, Pakistan*

## EPV‐0211

### Management and outcomes of ischemic stroke or TIA in young patients with patent foramen ovale: A retrospective cohort study

#### A. Vukšić; K. Radoš; I. Benić; B. Malojčić

##### Department of Neurology, University Hospital Centre Zagreb, Croatia

## EPV‐0212

### Mechanical thrombectomy beyond 6 hours: Outcomes in a real‐world cohort

#### A. Arabadji ^1^; E. Costru‐Tașnic^2^; M. Gavriliuc^1^; I. Teacă^1^; I. Preguza^2^; M. Arion^2^; O. Gavriliuc^2^

##### ^*1*^ *Neurology No 1, “Nicolae Testemitanu” State University of Medicine and Pharmacy, Chisinau, Republic of Moldova;* ^*2*^ *“Diomid Gherman” Institute of Neurology and Neurosurgery, Chisinau, Republic of Moldova*

## EPV‐0213

### Mechanical thrombectomy in minor ischemic stroke (NIHSS ≤5) with large‐ or medium‐vessel occlusion: A retrospective cohort study

#### M. Andrade Ferreira ^1^; I. Ribeiro^2^; F. Assis Jacinto^1^; R. Garcia^2^; C. Duque^1^; V. Tedim Cruz^1^

##### ^*1*^ *Neurology Department, Unidade Local de Saúde de Matosinhos, Matosinhos, Portugal;* ^*2*^ *Neurorradiology Department, Unidade Local de Saúde de Matosinhos, Matosinhos, Portugal*

## EPV‐0214

### Multisegmental vascular disease following fluoroquinolone use: A case of simultaneous carotid and vertebral artery dissection

#### A. Orejón Sánchez; A. Garcia Rúa; D. Alonso Vallín; V. Garcia Garcia; B. Quintana López; C. Castaño Ron; P. Díaz Aguirre; C. Gonzalez Antón; S. Rodriguez Rodriguez; M. Temprano Fernández; M. Martínez Palicio

##### Neurologia. Hospital Universitario de Cabueñes, Gijon, Asturias, Spain

## EPV‐0215

### Neurological complications in children with sickle cell disease in Senegal: A retrospective study of 57 cases from January 2020 to October 2024

#### A. Djemo Yemo; F. Niakam M; M. Ndiaye

##### Dakar, CNHU de FANN, Dakar, Sénégal

## EPV‐0216

### Neuromodulation‐Driven Neuroplasticity for Motor Recovery After Stroke: A Systematic Review of Adjunctive Rehabilitation Strategies

#### L. García Kornhauser; M. Sierra Reyes; S. Pérez Servín; A. Guzmán Salazar

##### Universidad de Guanajuato, División Ciencias De La Salud León Guanajuato, México

## EPV‐0217

### Neuropsychological anxiety and opioid activity in chronic cerebral ischemia without somatovegetative syndrome

#### D. Mirzaeva; D. Tolibov; N. Abdurasulova

##### Tashkent State Medical University, Tashkent, Uzbekistan

## EPV‐0218

### Neurorecovery in the digital era: Smartwatches and smartphones in post‐stroke care – A scoping review of validation studies and clinical trials

#### J. Datinguinoo ^1^; J. Lokin^2^

##### ^*1*^ *Department of Neuroscience and Behavioral Medicine, University of Santo Tomas Hospital, Manila, Philippines;* ^*2*^ *Department of Neuroscience and Behavioral Medicine, University of Santo Tomas Hospital, Manila, Philippines*

## EPV‐0219

### On the definition and typology of cognitive deficits in stroke

#### F. Sepe ^1^; F. Ferrari^2^; B. Sorti^3^; M. Caterino^4^; G. Reale^5^; M. Pasca^6^; N. Brunellli^7^; S. Diamanti^8^

##### ^*1*^ *M.Bufalini, Cesena, Italy;* ^*2*^ *Dipartimento Scienze della Salute UPO Novara Italy;* ^*3*^ *IRCCS Carlo Besta, Milan, Italy;* ^*4*^ *San Giovanni di Dio e Ruggi d'Aragona, Salerno, Italy;* ^*5*^ *Policlinico Universitario Agostino Gemelli,Rome, Itlay;* ^*6*^ *Ospedale F. Ferrari, Casarano, Italy;* ^*7*^ *Vall d'Hebron University Hospita, Barcelona, Spain;* ^*8*^ *Fondazione IRCCS San Gerardo dei Tintori, Monza, Italy*

## EPV‐0220

### Oral anticoagulation in cerebral venous thrombosis: strategies and clinical outcomes after discharge

#### A. Morgadinho; J. Oliveira; T. Martins; L. Pereira; M. Rodrigues

##### Neurology Department, Unidade Local de Saúde de Almada‐Seixal, Almada, Portugal

## EPV‐0221

### Persistent gaps in the management of neurovascular diseases in Haiti: Challenges, consequences, and urgent perspectives

#### E. Douyon

##### Hôpital Immaculée Conception des Cayes, Haiti

## EPV‐0222

### Post‐stroke anxiety disorders and their psychocorrection

#### N. Maxamatjanova; Ibodullayev; S. Gafurova; G. Ishankhodjayeva; S. Yusuphodjayeva; G. Ernayeva

##### Tashkent State Medical University, Neurology and Medical Psychology Department, Tashkent, Uzbekistan

## EPV‐0223

### Potential prognostic value of blood biomarkers in clarifying the diagnosis of ischemic stroke

#### A. Levitskaia; P. Kamchatnov

##### Department of Neurology, Neurosurgery and Medical Genetics of the Institute of Neuroscience and Neurotechnology; N.I. Pirogov Russian National Research Medical University; Moscow, Russian Federation

## EPV‐0224

### Predictors of early neurological deterioration in acute minor ischemic stroke: A systematic review and meta‐analysis

#### A. Hegazi; H. Heussien; R. Mohamed

##### Faculty of Medicine, Mansoura University, Mansoura, Egypt

## EPV‐0225

### Predictors of malignant brain edema after mechanical thrombectomy for ischemic stroke patients: A systematic review and meta‐analysis

#### R. Mohamed ^1^; M. Eldesoky^1^; A. Hegazi^1^; M. Meshref^2^

##### ^*1*^ *Mansoura Manchester Program for Medical Education, Faculty of Medicine, Mansoura University, Mansoura, Egypt;* ^*2*^ *Neurology Department, Faculty of Medicine, Alazhar University, Cairo, Egypt*

## EPV‐0226

### Prescription of dual antiplatelet therapy beyond guidelines in acute ischemic stroke: Real‐world evidence

#### A. Beglaryan ^2^; G. Buniatyan^2^; A. Meliksetyan^1^; M. Asatryan^1^; N. Grigoryan^1^; R. Karapetyan^1^; N. Nazinyan^1^; H. Sargsyan^1^; M. Grigoryan^1^; A. Shiroyan^1^; I. Shadyan^1^; M. Ghambaryan^1^; A. Manukyan^2^; A. Vardanyan^2^; N. Yeghiazaryan^1^

##### ^*1*^ *Erebouni Medical Center, Yerevan, Armenia;* ^*2*^ *Department of Neurology and Neurosurgery, National Institute of Health, Yerevan, Armenia*

## EPV‐0227

### Primary vs rescue thrombectomy in the therapeutic management of acute ischemic stroke: A three‐year comparative study

#### A. Jiménez Barreto ^1^; D. Alonso Modino^2^; I. Tejera Martín^3^; A. Chávez Padrón^4^

##### ^*1*^ *Neurology Service, Hospital Universitario Nuestra Señora de la Candelaria, Santa Cruz de Tenerife, Spain;* ^*2*^ *Neurology Service, Hospital Universitario Nuestra Señora de la Candelaria, Santa Cruz de Tenerife, Spain;* ^*3*^ *Neurology Service, Hospital Universitario Nuestra Señora de la Candelaria, Santa Cruz de Tenerife, Spain;* ^*4*^ *Neurology Service. Hospital Universitario Nuestra Señora de la Candelaria, Santa Cruz de Tenerife, Spain*

## EPV‐0228

### Prioritizing components for a healthy lifestyle intervention post‐stroke: A cross‐country Patient and Public Involvement‐based descriptive analysis

#### P. da Cruz Peniche ^1^; O. Lennon^2^; P. Hall^2^; J. Melo dos Santos Ferreira^1^; L. Ferreira Marques^1^; G. Florentino Sant'Ana de Assunção^1^; N. Arantes Betancourt Rosa^1^; B. Cardoso Alves Martins^1^; C. Danielli Coelho de Morais Faria^1^

##### ^*1*^ *Department of Physiotherapy, Universidade Federal de Minas Gerais, Belo Horizonte, Brazil;* ^*2*^ *School of Public Health, Physiotherapy and Sports Science, University College Dublin, Dublin, Ireland*

## EPV‐0229

### Prognostic factors in patients with stroke undergoing endovascular treatment

#### J. Almeida Ferreira; J. Oliveira; T. Geraldes; L. Pereira; M. Rodrigues; C. Rodrigues

##### Department of Neurology, Hospital Garcia de Orta, Unidade Local de Saúde Almada‐Seixal, Almada, Portugal

## EPV‐0230

### Abstract withdrawn

## EPV‐0231

### Proposed clinical practice protocol for the treatment of stroke at the hospital level

#### A. Gonzalez ^1^; U. Yarbouh^2^; L. Acevedo^3^

##### ^*1*^ *General Medicine, CDI Ciudad Caribia, Caracas, Venezuela;* ^*2*^ *General Medicine, Caracas, Venezuela;* ^*3*^ *Neurosurgery Service, Hospital Vargas, Caracas, Venezuela*

## EPV‐0232

### Prourokinase in acute ischemic stroke: A review of current evidence

#### H. Jagunmolu

##### Department of Medicine, Ladoke Akintola University of Technology, Chs Oyo, Nigeria

## EPV‐0233

### Recanalization therapy of acute stroke in elderly: Two‐year real‐life prospective data from Armenia

#### M. Asatryan ^1^; I. Shadyan^1^; M. Ghambaryan^1^; H. Sargsyan^1^; R. Karapetyan^1^; N. Nazinyan^1^; M. Grigoryan^1^; A. Shiroyan^1^; A. Meliksetyan^1^; N. Grigoryan^1^; G. Buniatyan^1^; M. Balasanyan^1^; H. Ezoyan^1^; L. Barseghyan^1^; L. Madoyan^1^; M. Margaryan^1^; N. Danielyan^1^; M. Aghasaryan^2^; N. Yeghiazaryan^1^

##### ^*1*^ *Neurology Department & Stroke Unit, Erebouni Medical Center, Yerevan, Armenia.;* ^*2*^ *Department of Neurology, CH Sud Francilien, Corbeil‐Essonnes, France*

## EPV‐0234

### Recurrent cerebral hemorrhage as a late manifestation of 22q11.2 deletion syndrome: A case report and literature review

#### L. de Castro Rocha; D. de Pinho; P. Barros; H. Costa; M. Rocha; T. Gregório; L. Paredes; J. Novo

##### Unidade AVC, Unidade Local de Saúde de Gaia e Espinho, Portugal

## EPV‐0235

### Recurrent intracranial hemorrhage in a young adult revealing iatrogenic cerebral amyloid angiopathy

#### L. Portela Martínez ^1^; A. Iglesias Mohedano^2^; M. Lozano López^1^; J. Sosa Luis^1^; R. Boto Martínez^1^; A. Contreras Chicote^1^; J. García Domínguez^1^; M. Vales Montero^2^; F. Díaz Otero^2^; P. Vázquez Alen^2^; J. Darriba Alles^3^; A. García Pastor^2^

##### ^*1*^ *Neurology Department, Hospital General Universitario Gregorio Marañón, Madrid, Spain;* ^*2*^ *Vascular Neurology Section and Stroke Centre, Department of Neurology, Hospital General Universitario Gregorio Marañón, Madrid, Spain;* ^*3*^ *Neurosurgery Department, Hospital General Universitario Gregorio Marañón, Madrid, Spain*

## EPV‐0236

### Recurrent transient brachial paresis: When vascular etiology does not seem to explain everything

#### P. Grilo ^1^; A. Oliveira^1^; E. Campos Costa^2^

##### ^*1*^ *Neurology Department, Hospital Vila Franca de Xira, Unidade Local de Saúde do Estuário do Tejo, Portugal;* ^*2*^ *Neurology Department, Hospital de Cascais, Portugal*

## EPV‐0237

### Recurrent transient ischemic attacks associated with long‐term Nilotinib: A case of accelerated Atherosclerosis

#### L. Caballero Sánchez; M. Capra; C. Gómez López de San Román; I. Bermejo Casado; A. De Luca; M. Vargas Cobos; R. Alfonso González; D. Cerdán Santacruz; A. Castrillo Sanz; A. Mendoza Rodríguez

##### General Hospital of Segovia, Segovia, Spain

## EPV‐0238

### Repeated intravenous thrombolysis within one day for recurrent ischemic stroke

#### D. Černík ^1^; J. Macko^2^; J. Neumann^3^

##### ^*1*^ *Comprehensive Stroke Center, Department of Neurology, Masaryk Hospital, Krajska Zdravotni As, Ústí nad Labem, Comprehensive Stroke Center, Department of Neurology, Palacký University Medical School and University Hospital Olomouc, Czech Republic;* ^*2*^ *Stroke Center, Department of Neurology, Most Hospital, Krajska zdravotni as, Most, Czech Republic;* ^*3*^ *Stroke Center, Department of Neurology, Chomutov Hospital, Krajska Zdravotni As, Chomutov, Czech Republic*

## EPV‐0239

### Reperfusion therapy in stroke patients over 80 years: Risks and outcomes from a Stroke Center in the Republic of Moldova

#### T. Stupac ^1^; V. Spinei^1^; A. Levca^1^; I. Preguza^1^; A. Grumeza^1^; V. Lisnic^3^; O. Grosu^2^

##### ^*1*^ *Stroke Unit, Department of Neurology and Neurosurgery, Institute of Neurology and Neurosurgery “Diomid Gherman”, Hospital, Chisinau, Republic of Moldova;* ^*2*^ *Department of Neurology, Institute of Neurology and Neurosurgery “Diomid Gherman”, Hospital, Chisinau, Republic of Moldova;* ^*3*^ *State University of Medicine and Pharmacy “Nicolae Testemitanu”, Chisinau, Republic of Moldova*

## EPV‐0240

### Results of indication for primary air transport of patients with suspected stroke based on telephone verification of FAST+ positivity

#### J. Bilik^2^; D. Černík ^1^

##### ^*1*^ *Comprehensive Stroke Center, Department of Neurology, Palacký University Medical School and University Hospital Olomouc, Czech Republic;* ^*2*^ *Emergency Medical Service of the Usti nad Labem Region, Czech Republic*

## EPV‐0241

### Reversible cerebral vasoconstriction syndrome and non‐aneurysmal intracerebral haemorrhage: Diagnostic role of delayed digital subtraction angiography

#### Z. Jiang; N. Singh; A. Chandratheva

##### Neurological Institute, Cleveland Clinic London, London, UK

## EPV‐0242

### Reversible cerebral vasoconstriction syndrome associated with hydrogen sulphide exposure: A rare environmental trigger

#### J. Aguilera Aguilera; B. Rodriguez Garcia; D. Gómez de la Torre Morales; J. Rodríguez Carrillo; M. Luz Esteve; I. Díaz Díaz; J. Vizcaya Gaona; Í. Esain González; G. Carvalho Monteiro

##### Salamanca University Hospital, Salamanca, Spain

## EPV‐0243

### Robotic assisted stroke rehabilitation: Does it improve functional recovery and patient outcomes?

#### A. Ahmad; E. Ademeta

##### *Faculty of Medicine, University of Novi Sad*, *Novi Sad, Serbia*

## EPV‐0244

### Routine coagulation parameters and severity of neurological deficit in women with ischemic stroke

#### K. Manysheva

##### Dagestan State Medical University, Russian Federation

## EPV‐0245

### Screening for poststroke Screening for poststroke cognitive impairment (PSCI) in real practice

#### Z. Gdovinova^1^; D. Koren ^2^; M. Slavkovska^2^

##### ^*1*^ *Department of Neurology, P. J. Safarik University, Faculty of Medicine, Kosice, Slovakia;* ^*2*^ *Department of Neurology, University Hospital L. Pasteur, Kosice, Slovakia*

## EPV‐0246

### Seeds from another source: Iatrogenic cerebral amyloid angiopathy

#### F. Duca ^1^; A. Bettinelli^1^; E. Scelzo^2^; K. Khouri Chalouhi^2^; A. Priori^1^; L. Tancredi^2^

##### ^*1*^ *Department of Biomedical and Clinical Science, University of Milan, Milan, Italy;* ^*2*^ *Department of Neuroscience, ASST Santi Paolo e Carlo, Milan, Italy*

## EPV‐0247

### Sex differences in acute ischaemic stroke treated with reperfusion therapy: A retrospective registry analysis

#### M. Pfister ^1^; M. Peycheva^2^; C. Berger^1^; M. Costa^1^; L. Siegenthaler^1^; F. Huber^1^; I. Rizzo^1^; M. Krasteva^3^; M. Beyeler^1^; T. Bremova‐Ertl^1^; N. Slavova^1^; P. Mordasini^4^; R. Umarova^1^; P. Bücke^1^; K. Antonenko^1^; A. Hoffmann^5^; S. Pilgram‐Pastor^4^; M. Heldner^1^

##### ^*1*^ *Department of Neurology, Inselspital, Bern University hospital and University of Bern, Bern, Switzerland;* ^*2*^ *Department of Neurology, University Hospital “Saint George”, Medical University Plovdiv, Plovdiv, Bulgaria;* ^*3*^ *University Children's Hospital, Sofia, Bulgaria;* ^*4*^ *Department of Neuroradiologie, Kantonsspital Aarau, Aarau, Switzerland;* ^*5*^ *Institute of Diagnostic and Interventional Neuroradiology, Inselspital, University Hospital and University of Bern, Bern, Switzerland*

## EPV‐0248

### Sex differences in acute ischemic stroke thrombolysis: Insights from a real‐world registry

#### A. Levca ^1^; V. Spinei^1^; T. Stupac^1^; A. Grumeza^1^; V. Lisnic^2^; O. Grosu^1^

##### ^*1*^ *Emergency Department, Diomid Gherman Institute of Neurology and Neurosurgery, Chișinău, Republic of Moldova;* ^*2*^ *Nicolae Testemițanu State University of Medicine and Pharmacy, Chișinău, Republic of Moldova*

## EPV‐0249

### Sex differences in mechanical thrombectomy timing and early outcomes in acute ischemic stroke: A single‐center retrospective cohort study

#### E. Costru‐Tasnic ^1^; E. Manole^2^; A. Arabadji^2^; I. Teaca^2^; I. Preguza^1^; M. Arion^1^; O. Gavriliuc^1^; O. Odainic^1^

##### ^*1*^ *Diomid Gherman Institute of Neurology and Neurosurgery, Chisinau, Republic of Moldova;* ^*2*^ *Nicolae Testemitanu State University of Medicine and Pharmacy, Chisinau, Republic of Moldova*

## EPV‐0250

### Sneezing‐induced sudden headache as an initial manifestation of convexity subarachnoid hemorrhage: A case report

#### C. Gomez López de San Román ^1^; M. Vargas Cobos^1^; A. De Luca^1^; R. Alfonso González^1^; M. Capra^1^; S. Arribas Bernardo^2^; L. Caballero Sánchez^1^; I. Bermejo Casado^1^; D. Cerdán Santacruz^1^; A. Castrillo Sanz^1^; A. Mendoza Rodriguez^1^

##### ^*1*^ *Neurology Deparment, Segovia Hospital, Segovia, Spain;* ^*2*^ *Health Emergencies, Management of Castilla and León, Spain*

## EPV‐0251

### Splenial ischemic stroke in a young woman with systemic inflammation and probable serotonergic toxicity

#### K. Paposhvili ^1^; N. Rizhamadze^2^; T. Margania^3^

##### ^*1*^ *Department of Neurology, LTD New Hospitals, Tbilisi, Georgia;* ^*2*^ *Department of Neurology, LTD New Hospitals, Tbilisi, Georgia;* ^*3*^ *Department of Neurology, LTD New Hospitals, Tbilisi, Georgia*

## EPV‐0252

### Spontaneous triple flexion after thalamo‐capsular haemorrhagic stroke

#### I. Carmo e Pinto; J. Blazer Costa; A. Sobral Pinho; F. Serrazina

##### Neurology Department, ULS Lisboa Ocidental, Lisbon, Portugal

## EPV‐0253

### Stress, autonomic dysfunction, and cortisol profile in chronic cerebral ischemia with and without somatovegetative syndrome

#### D. Mirzaeva; D. Tolibov; S. Shokirov

##### *Tashkent State Medical University, Tashkent,* Uzbekistan

## EPV‐0254

### Stroke burden and risk factors in India, 1990–2023: Age–sex stratified GBD analysis

#### M. Nath ^1^; P. Kumar^2^; D. Vibha^1^

##### ^*1*^ *Department of Neurology, Neurosciences Center, All India Institute of Medical Sciences, New Delhi, India;* ^*2*^ *Clinical Research Unit, All India Institute of Medical Sciences, New Delhi, India*

## EPV‐0255

### Stroke characteristics and outcomes in two neurology units in a Sub‐Saharan African country

#### M. Njohjam

##### Department of Neurology, National University Hospital Center, Dakar, Senegal

## EPV‐0256

### Temporal trends in particulate matter‐attributable stroke across Europe. Insights from the global burden of disease 2023 study (1990‐2023)

#### S. Wasti^1^; A. Wasti^2^; M. Javed ^1^

##### ^*1*^ *Department of Neurology, King Edward Medical University, Lahore, Pakistan;* ^*2*^ *Department of Neurology, Continental Medical College, Lahore, Pakistan*

## EPV‐0257

### Tenecteplase versus alteplase before mechanical thrombectomy in acute ischaemic stroke: A retrospective comparative study

#### P. Diaz‐Corta; B. Lara‐Rodríguez; A. Paipa‐Merchan; H. Quesada‐García; A. Núñez‐Guillén; P. Cardona‐Portela

##### Neurology, Hospital Universitario de Bellvitge, Barcelona, Spain

## EPV‐0258

### Tenecteplase vs alteplase for large vessel occlusion stroke: An updated systematic review and meta‐analysis

#### M. Cierny^1^; D. Vodak ^2^; C. Moore^1^; C. Herath^1^; H. Attar^1^; R. Licenik^3^

##### ^*1*^ *Medical College of Wisconsin, Milwaukee, WI, USA;* ^*2*^ *Department of Neurology, St. Anne's University Hospital and Faculty of Medicine, Masaryk University, Brno, Czech Republic;* ^*3*^ *Acute Stroke Centre, North West Anglia NHS Foundation Trust, Peterborough, England, UK*

## EPV‐0259

### The beta‐blocker nebivolol therapy in acute ischemic stroke: Effects on nitric oxide metabolism

#### M. Ibodullaeva; K. Daminova

##### Department of Neurology and Clinical Psychology, Tashkent State Medical University, Tashkent, Uzbekistan

## EPV‐0260

### The evolving epidemiology of ischemic heart disease and ischemic stroke in Europe: Observational and genetic evidence from GBD 2023

#### H. Hu; F. Yang

##### The First Medical Center, Chinese PLA General Hospital, Beijing, China

## EPV‐0261

### The predictive value of the stroke oxidative‐inflammatory index in hemorrhagic stroke: A retrospective cohort study

#### A. Sönmezler; E. Altunışık; B. Yılmaz; S. Parlak; D. Alpar

##### Department of Neurology, Gaziantep City Research and Training Hospital, Gaziantep, Türkiye

## EPV‐0262

### The prevalence of cerebral amyloid angiopathy among the causes of hemorrhagic stroke

#### O. Novosadova; T. Semenova; V. Grigoryeva

##### Privolzhsky Research Medical University, Nizhny Novgorod, Russian Federation

## EPV‐0263

### The relationship between dehydration levels on admission, blood viscosity, and early neurological deterioration in patients with lacunar strokes

#### S. Han

##### Department of Neurology, Sanggye Paik Hospital, Inje University College of Medicine, Seoul, Republic of Korea

## EPV‐0264

### The role of inflammatory cytokine IL‐6 in cognitive impairment after acute ischemic stroke: A preliminary study

#### A. Sarnataro ^1^; A. Smimmo^2^; G. Riccio^1^; E. Spina^1^; F. Manganelli^1^

##### ^*1*^ *University of Naples Federico II, Naples, Italy;* ^*2*^ *Università della Campania Luigi Vanvitelli, Naples, Italy*

## EPV‐0265

### The Role of undiagnosed obstructive sleep apnea in recurrence of transient ischemic attacks and progression of Leukoaraiosis: A pilot cohort study

#### O. Kasimova; H. Daminova

##### Department of Neurology, Tashkent Medical University Hospital, Tashkent, Uzbekistan

## EPV‐0266

### Tocilizumab for the treatment of cerebral amyloid angiopathy‐related inflammation with elevated IL‐6: A case report

#### J. Huang; R. Guo; Z. Jia; Y. Sun; B. Li

##### Department of Neurology, The Second Hospital of Hebei Medical University, Shijiazhuang, China; Key Laboratory of Neurology (Hebei Medical University), Ministry of Education, China; Key Laboratory of Neurology of Hebei Province, Shijiazhuang, China

## EPV‐0267

### Trends and disparities in colon cancer and stroke‐related mortality in the United States: A retrospective study

#### S. Wasti^1^; A. Wasti^2^; F. Rizwan ^1^

##### ^*1*^ *Department of Neurology, King Edward Medical University, Lahore, Pakistan;* ^*2*^ *Department of Neurology, Continental Medical College, Lahore, Pakistan*

## EPV‐0268

### Trends and disparities in stroke‐related mortality in inflammatory bowel disease patients

#### S. Wasti^1^; A. Wasti^2^; E. Shafquat ^1^

##### ^*1*^ *Department of Neurology, King Edward Medical University, Lahore, Pakistan;* ^*2*^ *Department of Neurology, Continental Medical College, Lahore, Pakistan*

## EPV‐0269

### Umbrella review of endovascular thrombectomy time windows and outcomes in acute ischemic stroke

#### F. Aljamea ^1^; N. Aderinto^2^; D. Babalola^3^; A. Onyiа^4^; E. Mbah^5^; E. Motimbe^6^; E. Ogungbemi^7^; S. Kallah^8^

##### ^*1*^ *College of Medicine, Vision College, Riyadh, Saudi Arabia;* ^*2*^ *Department of Medicine and Surgery, Ladoke Akintola University of Technology, Ogbomosho, Nigeria;* ^*3*^ *University of Ilorin Teaching Hospital, Nigeria;* ^*4*^ *University of Nigeria Nsukka, Nigeria;* ^*5*^ *Ahmadu Bello University (ABU) Zaria, Nigeria;* ^*6*^ *University of Buea, Cameroon;* ^*7*^ *Rostov State Medical University, Rostov‐on‐Don, Russian Federation;* ^*8*^ *AMA School of Medicine, Philippines*

## EPV‐0270

### Using an artificial intelligence‐based machine learning approach to understand the future of declining burden of stroke in the United Kingdom

#### S. Wasti^1^; A. Wasti^2^; F. Rizwan ^1^

##### ^*1*^ *Department of Neurology, King Edward Medical University, Lahore, Pakistan;* ^*2*^ *Department of Neurology, Continental Medical College, Lahore, Pakistan*

## EPV‐0271

### Vascular risk burden as a predictor of clinical outcomes after thrombolysis in acute ischemic stroke: Single‐centre study

#### A. Solodjankina

##### Riga Stradiņš University, Riga East Clinical University Hospital, Riga, Latvia

## EPV‐0272

### Vasospasm after aneurysmal subarachnoid hemorrhage: Triggering factors and clinical outcomes

#### G. Hatzidaki; A. Tsamourgelis ^1^; I. Tyrovolas; G. Tagaris

##### Department of Neurology, Georgios Gennimatas General Hospital, Athens, Greece

## EPV‐0273

### Venous sinus stenosis in idiopathic intracranial hypertension: Intracranial compliance after best medical therapy versus venous sinus stenting

#### L. Souza Viana; F. Moulin de Moraes; S. Luiz De Andrade Matas; G. Sampaio Silva

##### Federal University of São Paulo, São Paulo, Brazil

## EPV‐0274

### Very late‐onset HTRA1‐related autosomal dominant cerebral small vessel disease misdiagnosed as multiple sclerosis

#### M. Mora Conde ^1^; M. Calvello^2^; S. Antonarakis^3^; A. Batté^3^; E. Ranza^3^; G. Padlina^4^; M. Raimondi^4^

##### ^*1*^ *Department of Geriatrics, Gruppo Ospedaliero Moncucco, Lugano, Switzerland;* ^*2*^ *Oncology Competence Center, Gruppo Ospedaliero Moncucco, Lugano, Switzerland;* ^*3*^ *Medigenome, Swiss Genomic Institute of Genomic Medicine, Geneva, Switzerland;* ^*4*^ *Department of Neurology, Gruppo Ospedaliero Moncucco, Lugano, Switzerland*

## EPV‐0275

### Wernekink's syndrome: A case report

#### M. González Gómez; E. Gismera Fontes; F. Sánchez Garcia; L. Cabral Esquea; L. Ballesteros Plaza

##### Department of Neurology. Guadalajara's University Hospital, Guadalajara, Spain

## EPV‐0276

### White blood cells to HDL ratio as a superior predictor of ischemic stroke clinical severity

#### F. Hakim; S. Daoud; S. Sakka; K. Moalla; N. Bouattour; N. Charfi; E. Smaoui; M. Damak

##### Neurology Department, Habib Bourguiba Hospital, Sfax, Tunisia

## Child neurology/developmental neurology

## EPV‐0277

### A Network meta‐analysis of stem cell for cerebral Palsy: Does route of administration and stem cell type matters?

#### K. Siregar ^1^; Y. Ramli^2^; S. Tanumihardja^3^

##### ^*1*^ *School of Medicine Maranatha Christian University, Bandung, Indonesia;* ^*2*^ *Department of Neurology, Faculty of Medicine, Universitas Indonesia, Jakarta, Indonesia;* ^*3*^ *Department of Neurology, Faculty of Medicine, Maranatha Christian University, Bandung, Indonesia*

## EPV‐0278

### A study of the clinical characteristics, etiology and predictors of severity of global developmental delay in children

#### A. Srivastava; V. Mani; A. Mishra; P. Chaurasiya

##### Department of Neurology, Sanjay Gandhi Postgraduate Institute of Medical Sciences, Lucknow, India

## EPV‐0279

### Acute peripheral facial palsy of neurological origin in the pediatric population

#### F. Dridi; M. Jamoussi; H. Klaa; Z. Miladi; A. Zioudi; M. Ben Hafsa; H. Benrhouma; T. Ben Younes; I. Kraoua

##### Pediatric Neurology Department, National Institute Mongi Ben Hmida of Neurology, Tunis, Tunisia

## EPV‐0280

### Age and diagnosis determine TMS Efficacy in preschoolers with developmental disorders

#### M. Askar

##### Kazakh National Medical University named after S.D. Asfendiyarov, Almaty, Kazakhstan

## EPV‐0281

### An investigation of stuttering prevalence in children in Tashkent, Uzbekistan

#### D. Nurmukhamedova; M. Nurmukhamedova

##### Department of Neurology, Child neurology and Medical Genetics, Tashkent State Medical University, Tashkent, Uzbekistan

## EPV‐0282

### Clinical and functional manifestations of diabetic cardiovascular autonomic neuropathy in children with type 1 diabetes

#### A. Sadirkhodjaeva; D. Ashurova; A. Malikova

##### Department of Propedeutics of Childhood Diseases No. 2, Tashkent State Medical University, Tashkent, Uzbekistan

## EPV‐0283

### Clinical and instrumental phenotyping of gait in subjects with Cri du Chat syndrome

#### B. Agostini ^1^; G. Vaghi^1^; M. Corrado^2^; V. Grillo^1^; L. Martinis^2^; M. Gjini^2^; I. Campese^2^; M. Giraudo^1^; F. Cammarota^1^; S. Ravaglia^2^; M. Croce^1^; V. De Giorgis^1^; D. Politano^1^; S. Castiglia^3^; D. Trabassi^3^; M. Serrao^3^; C. Danesino^4^; A. Guala^5^; C. Tassorelli^1^; R. De Icco^1^

##### ^*1*^ *Department of Brain and Behavioural Sciences, University of Pavia, Pavia, Italy;* ^*2*^ *Movement Analysis Research Section, IRCCS Mondino Foundation, Pavia, Italy;* ^*3*^ *Department of Medical and Surgical Sciences and Biotechnologies, “Sapienza” University of Rome, Polo Pontino, Latina, Italy;* ^*4*^ *Department of Molecular Medicine, University of Pavia, Pavia, Italy;* ^*5*^ *SOC Pediatria, Ospedale Castelli, Verbania, Verbania, Italy*

## EPV‐0284

### Clinical predictors of global developmental delay in Azerbaijani Patients with infantile epileptic spasms syndrome

#### U. Guliyeva; K. Salayev; S. Guliyeva; C. Isayev; R. Kaiyrzhanov

##### Department of Neuromuscular Diseases, UCL Queen Square Institute of Neurology, Queen Square, London, UK

## EPV‐0285

### Cognitive status and non‐specific school in children with attention‐deficit/hyperactivity disorder

#### Y. Madjidova

##### Department Neurology and Child Neurology, Tashkent State Medical University, Tashkent, Uzbekistan

## EPV‐0286

### Daily posture and movement profiles in Saudi children with cerebral palsy: An ActivPAL‐based comparative study

#### M. Alghadier ^1^; N. Almasoud^2^; D. Alharthi^3^; O. Alrshedi^4^; R. Albesher^5^; S. Alothman^6^

##### ^*1*^ *Department of Health and Rehabilitation Sciences, Prince Sattam bin Abdulaziz University, Saudi Arabia;* ^*2*^ *Maternity and Children's Hospital in Al‐Kharj, Alkharj, Saudi Arabia;* ^*3*^ *Alhada Armed Forces Hospital, Taif, Saudi Arabia;* ^*4*^ *King Khalid Hospital, Hail, Saudi Arabia;* ^*5*^ *Department of Rehabilitation Sciences, Princess Nourah bint Abdulrahman University, Riyadh, Saudi Arabia;* ^*6*^ *Lifestyle and Health Research Center, Health Sciences Research Center, Princess Nourah bint Abdulrahman University, Riyadh, Saudi Arabia*

## EPV‐0287

### Development of a smartphone‐based pose estimation framework for automated pediatric motor assessment

#### M. Alghadier

##### Prince Sattam bin Abdulaziz University, Al‐Kharj, Saudi Arabia

## EPV‐0288

### Diagnostic accuracy of machine learning for schizophrenia spectrum disorders in pediatrics: A systematic review and meta‐analysis

#### O. Abbas ^1^; A. Kedwany^2^; D. Dalia Atef Abouda^3^; M. Fathy^4^; L. Ahmed^3^; R. Sahsah^5^; A. Elareef^3^; R. haddad^6^; A. Fathallah^7^

##### ^*1*^ *Faculty of medicine, Al‐Azhar University, Cairo, Egypt;* ^*2*^ *Assiut University Hospitals, Assiut, Egypt;* ^*3*^ *Faculty of Medicine, Alexandria University, Alexandria, Egypt;* ^*4*^ *Faculty of Medicine, Al Azhar University, New Damietta, Egypt;* ^*5*^ *Bachelor of Pharmacy, Faculty of Pharmacy, Cairo University;* ^*6*^ *Faculty of Medicine, October 6 University, Egypt;* ^*7*^ *Faculty of Medicine, Minia University, Minia, Egypt*

## EPV‐0289

### Diagnostic pathway and neuro‐investigations in pediatric sydenham chorea: A Tunisian cohort study

#### C. Karray; M. Ben Hafsa; H. Klaa; Z. Miladi; A. Zioudi; M. Jamoussi; T. Ben Younes; I. Kraoua; H. Benrhouma

##### Department of Pediatric Neurology, National Institute Mongi Ben Hmida of Neurology, Tunis, Tunisia

## EPV‐0290

### Diagnostic strategy and genetic landscape of syndromic microcephalies

#### A. Kalfat ^1^; T. Ben Younes^1^; L. Kraoua^2^; S. Drunat^3^; Z. Miladi^1^; A. Zioudi^1^; H. Ben Rhouma^1^; I. Ben Youssef Turki^1^; L. Ben Jemaa^4^; H. Klaa^1^; I. Kraoua^1^

##### ^*1*^ *LR18SP04 and Pediatric Neurology Department, National Mongi Ben Hmida Institute of Neurology, Tunis, Tunisia;* ^*2*^ *Department of Genetics, Charles Nicolle Hospital, Tunis, Tunisia;* ^*3*^ *Department Of Genetics, Robert‐Debré Hospital, Paris, France;* ^*4*^ *Department Of Genetics, Mongi Slim Hospital, La Marsa, Tunisia*

## EPV‐0291

### Early detection of autism spectrum disorder in children with cerebral Palsy using motor‐adapted diagnostic tools

#### K. Rakhimova ^1^; U. Omonova^2^

##### ^*1*^ *Republican Children's Psychoneurological Hospital named after U.K. Kurbanov, Department of Neurology, Pediatric Neurology and Medical Genetics*, *Tashkent, Uzbekistan;* ^*2*^ *Department of Neurology, Pediatric Neurology and Medical Genetics*, *Tashkent; Uzbekistan*

## EPV‐0292

### Effectiveness of interventions for cognitive enhancement in down syndrome: A network meta‐analysis

#### A. Sharma ^1^; A. Agarwal^2^; A. Bakhsh^3^; G. Kaur^4^; M. Biradar^5^; K. B^1^; S. Muddana^6^; R. Raj^7^; V. Suresh^8^; P. Kola^9^; A. Nithyanandam^10^

##### ^*1*^ *Government Medical College, Omandurar, Chennai, Tamil Nadu, India;* ^*2*^ *Osmania Medical College, Hyderabad, India;* ^*3*^ *Netaji Subash Chandra Bose Medical College, Jabalpur, Madhya Pradesh, India;* ^*4*^ *All India Institute Of Medical Sciences, Patna, India;* ^*5*^ *All India Institute Of Medical Science, Raipur, India;* ^*6*^ *Mamata Academy of Medical Sciences, Hyderabad, India;* ^*7*^ *Atal Bihari Vajpayee Institute of Medical Sciences and Dr. RML Hospital, New Delhi, India;* ^*8*^ *University of Oxford, UK;* ^*9*^ *All India Institute Of Medical Sciences, New Delhi, India;* ^*10*^ *Tamil Nadu Government Multi‐Super Speciality Hospital, Omandurar Government Estate, Chennai, India*

## EPV‐0293

### Effectiveness of microcurrent therapy in the multidisciplinary rehabilitation of children with microcephaly

#### M. Mambetkarimova; Y. Nabievna

##### Andijan State Medical Institute, Andizhan, Uzbekistan

## EPV‐0294

### Endovascular thrombectomy for paediatric large vessel occlusion stroke: A rapid evidence synthesis

#### A. Ramkaran; N. Mahadeosingh

##### Royal College of Surgeons in Ireland School of Medicine and Health Sciences, Dublin, Ireland

## EPV‐0295

### Experience using cenobamate in refractory epilepsy in children

#### A. Morgadinho ^1^; J. Carvalho^2^; T. Barata Silvério^2^; J. Paulo Monteiro^2^

##### ^*1*^ *Neurology Department, Unidade Local de Saúde de Almada‐Seixal, Almada, Portugal;* ^*2*^ *Torrado da Silva Child Development Center, Paediatrics Department, Unidade Local de Saúde de Almada‐Seixal, Almada, Portugal*

## EPV‐0296

### Gene–environment interactions shaping pediatric neurological phenotypes

#### S. Kulkarni

##### Department of Medicine, Georgian National University SEU, Tbilisi, Georgia

## EPV‐0297

### Health education strategies for early autism recognition in childhood: Implications for child neurology in middle‐income settings

#### C. Pace

##### Federal University of Rio de Janeiro, Rio de Janeiro, Brazil

## EPV‐0298

### Innovative method for the rehabilitation of motor disorders in children with cerebral Palsy

#### S. Gulyamova ^1^; N. Ergasheva^2^

##### ^*1*^ *Republican Children's Psychoneurological Hospital named after U. Kurbanov, Tashkent, Uzbekistan;* ^*2*^ *Republican Children's Psychoneurological Hospital named after U. Kurbanov, Tashkent, Uzbekistan*

## EPV‐0299

### Leigh syndrome: Consider in cases of late neurodevelopmental regression

#### B. Amine; B. Samia; T. Nadia; L. Malika

##### Neurology Department, IBN SINA, CHU, Annaba, Algerie

## EPV‐0300

### Lipid metabolic disorders in neonatal bilirubin encephalopathy: Novel findings from proteomic analysis in a rat model

#### J. Lei ^1^; N. Tan^1^; L. Zhang^2^; M. Yu^1^; Z. Chen^1^; X. Zheng^1^; H. Guo^1^; H. Jiang^3^

##### ^*1*^ *Neonatology, The Fifth Affiliated Hospital, Sun Yat‐sen University, Zhuhai, China;* ^*2*^ *Cerebrovascular Diseases, The Fifth Affiliated Hospital, Sun Yat‐sen University, Zhuhai, China;* ^*3*^ *Rheumatology, The Fifth Affiliated Hospital, Sun Yat‐sen University, Zhuhai, China*

## EPV‐0301

### Metabolic changes in the brain of children and adolescents with diabetes mellitus, taking into account the presence or absence of cognitive deficit

#### M. Baxramov; D. Alidjanova; Y. Madjidova

##### Tashkent State Medical University¸ Tashkent, Uzbekistan

## EPV‐0302

### Neuroimaging methods in the early diagnosis of cerebral impairment in children and adolescents with type 1 diabetes mellitus

#### D. Alidjanova; M. Bakhramov

##### Tashkent State Medical University, Tashkent, Uzbekistan

## EPV‐0303

### Neurological and speech manifestations of developmental speech delay in children: Impact of residual brain damage

#### K. Berdieva; Sadiqova; A. Sadirhodjaeva

##### Department of Neurology, Pediatric Neurology and Medical Genetics of the Tashkent State Medical University, Tashkent, Uzbekistan

## EPV‐0304

### Neurological and speech phenotypes of developmental speech delay in children: The role of residual brain damage

#### K. Berdieva

##### Department of Neurology, Pediatric Neurology and Medical Genetics of the Tashkent State Medical University, Tashkent, Uzbekistan

## EPV‐0305

### Neurological burden in neurocutaneous syndromes: Epilepsy, cognitive impairment, and imaging patterns in children

#### E. Cherif; Z. Miladi; H. Klaa; A. Zioudi; M. Jamoussi; T. Ben Younes; M. Ben Hafasa; H. Benrhouma; I. Kraoua

##### Research Unit LR18SP04 and Department of Child and Adolescent Neurology, National Institute Mongi Ben Hmida of Neurology of Tunis, Tunisia

## EPV‐0306

### Neurological changes in children with congenital dysplasia of the lower limb joints

#### O. Yuryk

##### *State Institution “Institute of Traumatology and Orthopaedics of the National Academy of Medical Sciences of Ukraine”, Kyiv*, *Ukraine*

## EPV‐0307

### Neurometabolic phenotype of torch‐associated autism in children

#### N. Khusenova

##### Tashkent State Medical University, Tashkent, Uzbekistan

## EPV‐0308

### Neurophysiological and cognitive correlates of developmental language disorder in preschool children

#### V. Eladi; S. Hadjiu

##### *Department of Pediatrics, State University of Medicine and Pharmacy,* Chisinau; *Republic of Moldova*

## EPV‐0309

### NK cell mediated immune alteration at the maternal fetal interface regulates the fetal neural development after influenza infection

#### R. Liu ^1^; Y. Zhao^2^; X. Song^1^; W. Jiang^1^

##### ^*1*^ *Department of Neurology, Tianjin Medical University General Hospital, Tianjin, China;* ^*2*^ *Chinese Institutes for Medical Research, Capital Medical University, Beijing, China*

## EPV‐0310

### Non‐invasive neuromodulation with repetitive transcranial magnetic stimulation for children with Tourette syndrome and chronic tic disorders

#### A. Mirzoyan; T. Charnukha; S. Kulikova; G. Zabrodzets

##### Republican Research and Clinical Center of Neurology and Neurosurgery, Minsk, Belarus

## EPV‐0311

### Preliminary insights into neuroinflammatory mediators in children with autism spectrum disorder

#### T. Gakharia; T. Natsvlishvili; S. Bakhtadze

##### Tbilisi State Medical University, Tbilisi, Georgia

## EPV‐0312

### Prevention of static contractures in cerebral Palsy: Long‐term prognostic effects of early botulinum toxin therapy

#### R. Guluzade

##### Neurorehabilitation, Teaching Therapeutic Clinic, Baku, Azerbaijan

## EPV‐0313

### Self‐limiting epilepsies of childhood: Experience of the single referral centre in Serbia

#### N. Ivančević ^1^; M. Vlajković^2^; I. Ivančević^2^; B. Nikolić^1^; J. Jančić^1^

##### ^*1*^ *Clinic of Neurology and Psychiatry for Children and Youth, Faculty of Medicine, University of Belgrade, Serbia;* ^*2*^ *Faculty of Medicine, University of Belgrade, Serbia*

## EPV‐0314

### Trends in pediatric intracranial hemorrhage‐related mortality in the United States: A retrospective analysis

#### S. Wasti^1^; A. Wasti^2^; S. Waqas ^1^

##### ^*1*^ *Department of Neurology, King Edward Medical University, Lahore, Pakistan;* ^*2*^ *Department of Neurology, Continental Medical College, Lahore, Pakistan*

## EPV‐0315

### BEAM™ EEG biomarkers as early indicators of cognitive decline: Insights from a retrospective cohort with mild cognitive impairment

#### J. Bow‐Keola^1^; D. Vodak ^1^; Y. Pine^1^; N. Brenner^2^; K. Aoki^1^; K. Moriyama^1^; H. Ahn^2^; K. Yamauchi^1^; M. Read^1^; K. Borrello^1^; S. Nakahira^1^; E. Carrazana^3^; K. Liow^4^

##### ^*1*^ *Alzheimer's Neural Network EEG Research Laboratory, Hawaii Pacific Neuroscience, Honolulu, USA;* ^*2*^ *University of Hawaii, Department of Quantitative Health Sciences, Honolulu, USA;* ^*3*^ *University of Hawaii, John A. Burns School of Medicine, Honolulu, USA;* ^*4*^ *Memory Disorders Center and Alzheimer's Disease Research Unit, Hawaii Pacific Neuroscience, Honolulu, USA*

## EPV‐0316

### Cervical dystonia: The objective evaluation of muscle tone

#### M. Falletti ^1^; F. Asci^2^; A. Zampogna^1^; M. Patera^1^; V. Bellia^1^; L. Longo^1^; E. Fusco^1^; C. Pauletti^1^; A. Suppa^1^

##### ^*1*^ *Department of Human Neurosciences, Sapienza University of Rome, Italy;* ^*2*^ *Department of Neurosciences and Sensory Organs, AO San Giovanni – Addolorata, Rome, Italy*

## EPV‐0317

### Clinical and electrophysiological spectrum of monoclonal gammopathies in neuromuscular practice

#### Ö. Kurtkaya Koçak ^1^; F. Bingöl^2^; Ö. Ergin Beton^1^; Ş. Bilen^1^

##### ^*1*^ *Ankara Bilkent City Hospital, Ankara; Türkiye;* ^*2*^ *Düzce Atatürk State Hospital; Düzce, Türkiye*

## EPV‐0318

### Clinical value of somatosensory and motor evoked potentials in nitrous oxide–induced myelopathy

#### S. Palma ^1^; J. Morgadinho^1^; C. Alves^1^; H. Rego^1^; M. Pires^1^; C. Santos Silva^1,2^; P. Pereira^1^

##### ^*1*^ *Department of Neurology, Hospital Garcia de Orta, Unidade Local de Saúde Almada‐Seixal, Almada, Portugal;* ^*2*^ *Faculty of Medicine, Institute of Molecular Medicine, University of Lisbon, Lisbon, Portugal*

## EPV‐0319

### Cognitive disorders in patients with carbon monoxide poisoning: Risk factors and predictors for delayed neuropsychological sequelae

#### D. Tsakova ^1^; L. Neykova‐ Vasileva^2^; M. Gesheva^3^; A. Loukova^3^; F. Tsakov^3^; M. Petkova^3^; P. Vanev^3^

##### ^*1*^ *Consulting and Diagnostic Department, Military Medical Academy, Sofia, Bulgaria;* ^*2*^ *Department of Toxicology, Military Medical Academy, Sofia, Bulgaria;* ^*3*^ *Toxicology Clinic, Pirogov University Hospital, Sofia, Bulgaria*

## EPV‐0320

### Evaluation of the effectiveness of botulinum toxin type A for hemifacial spasm using the Jankovic rating scale in Sri Lanka

#### S. Gunasekara

##### Department of Physiology, Faculty of Medicine, University of Peradeniya, Peradeniya, Sri Lanka

## EPV‐0321

### Feasibility of a portable, low‐cost multichannel EEG system using commercially available components

#### A. Mittal; C. Kwon

##### Departments of Neurology, Epidemiology, Neurosurgery, and the Gertrude H Sergievsky Center, Columbia University, New York City, NY, USA

## EPV‐0322

### From focal targets to network phenotypes in deep brain stimulation and the circuit reframing of OCD

#### A. Vila ^1^; E. Mejia^1^; B. Carr^2^

##### ^*1*^ *University of Florida College of Medicine, Gainesville, USA;* ^*2*^ *University of Florida Department of Psychiatry, Gainesville, USA*

## EPV‐0323

### Neuroinflammatory effects of repetitive transcranial magnetic stimulation and their clinical relevance in depressive disorders

#### I. Khayredinova ^1^; R. Karimova^2^; I. Khayredinova^2^; I. Khayredinova^3^

##### ^*1*^ *Republican Specialized Scientific and Practical Scientific Medical Centre of Mental health, Tashkent, Uzbekistan;* ^*2*^ *Department of Psychiatry and Narcology, Tashkent State Medical University, Tashkent;* ^*3*^ *Department of Narcology and Adolescent Psychopathology, Center for the Development of Professional Qualification of Medical Workers, Tashkent, Uzbekistan*

## EPV‐0324

### Progressive ataxia at the metabolic–neurodegenerative crossroads: Central diabetes insipidus as a clue to juvenile metachromatic leukodystrophy

#### A. Lungu ^1^; G. Malureanu^1^; A. Miclausanu^2^; A. Rotaru^2^; D. Cuciureanu^1^

##### ^*1*^ *Univeristy of Medicine and Pharmacy, “Grigore T.Popa”, Iasi, Romania;* ^*2*^ *Emergency Hospital, “Prof.Dr.N.Oblu”, Iasi, Romania*

## EPV‐0325

### Referral pattern, waiting time and outcome distribution in EMG: A retrospective descriptive study

#### V. Alisoy; B. Çelenk; B. Boztepe; B. Altunan; B. Karpuz Seren; A. Ünal

##### *Namik Kemal University Hospital*, *Tekirdag, Türkiye*

## EPV‐0326

### Self‐resolving, isolated Anti‐GD3–positive atypical Guillain–Barré syndrome: Case report and literature review

#### E. Yarenci ^1^; Ö. Kurtkaya Koçak^2^

##### ^*1*^ *Department of Neurology, University of Health Sciences Ankara Bilkent City Hospital, Ankara, Türkiye;* ^*2*^ *Department of Neurology, Section of Clinical Neurophysiology, Ankara Bilkent City Hospital, Ankara, Türkiye*

## EPV‐0327

### Spindle coma associated with acute carbamazepine intoxication in a paediatric patient

#### J. Andrada Moreno ^1^; C. Villar Rodríguez^1^; P. Orive Rodríguez^2^; I. Lopera Rodríguez^1^; J. Ciriero Macías^1^; L. Garcia Granados^1^; D. Villagrán Sancho^1^

##### ^*1*^ *Neurology, Hospital Virgen del Rocio, Seville, Spain;* ^*2*^ *Neurophysiology, Hospital Virgen del Rocio, Seville, Spain*

## EPV‐0328

### The electroencephalography response to the olfactory stimulus: Comparing automated and individualized approaches

#### K. Bošnjak‐Matić^1^; M. Cifrek^1^; M. Habek^2^; M. Krbot Skoric ^2^

##### ^*1*^ *Faculty of Electrical Engineering and Computing, University of Zagreb, Zagreb, Croatia;* ^*2*^ *University Hospital Center Zagreb, Department of Neurology, Referral Center for Autonomic Nervous System Disorders, Zagreb, Croatia*

## EPV‐0329

### Unveiling neuroradiological and neurophysiological features in a novel proteolipid protein 1 (PLP1) related disorder

#### G. Micolonghi ^1^; S. Masciocchi^3^; L. Campiglio^1^; M. Zardoni^1^; M. Pengo^1^; V. Yahya^1^; A. De Grado^4^; E. Vola^1^; M. Gastaldi^3^; A. Priori^2^; T. Bocci^2^

##### ^*1*^ *Clinical Neurology Unit, “Azienda Socio‐Sanitaria Territoriale Santi Paolo e Carlo”, Department of Health Sciences, University of Milan, Milan, Italy;* ^*2*^ *“Aldo Ravelli” Center for Neurotechnology and Experimental Brain Therapeutics, Department of Health Sciences, University of Milan, Milan, Italy;* ^*3*^ *Neuroimmunology Research Section, IRCCS Mondino Foundation, Pavia, Italy;* ^*4*^ *Neurophysiology Unit, Fondazione IRCCS Istituto Neurologico “Carlo Besta”, Milan, Italy*

## Cognitive neurology/neuropsychology

## EPV‐0330

### “Neuroendocrine cognitive dysregulation syndrome” developmental structure in thyroid disorders

#### B. Ibadullayev; D. Komilova; O. Qadamova; I. Abdullayev; S. Dushamov

##### Urgench State Medical Institute, Urgench, Uzbekistan

## EPV‐0331

### Apraxia severity independently predicts functional decline across neurodegenerative movement and cognitive disorders

#### E. Smaragdaki^1^; M. Tsolaki^2^; N. Scarmeas^1^; C. Potagas^1^; L. Stefanis^1^; S. Papageorgiou^1^; V. Konstantinidis ^1^

##### ^*1*^ *First Department of Neurology, Medical School, National and Kapodistrian University of Athens, Eginition Hospital*, *Greece;* ^*2*^ *Aristotle University of Thessaloniki, Aristotle University of Thessaloniki, Faculty of Medicine, Greece*

## EPV‐0332

### Are there cognitive disfunction in myasthenia gravis? An Italian cohort study

#### M. Leonardi ^1^; R. Frangiamore^2^; A. Raggi^1^; M. Lanza^1^; G. Trucco^1^; A. Colombo^1^; A. Mazzilli^1^; A. Fornari^1^; S. Bonanno^2^; R. Mantegazza^2^; C. Antozzi^2^; F. Vanoli^2^; L. Maggi^2^; A. Marcassoli^1^

##### ^*1*^ *Neurology, Public Health and Disability Unit, Fondazione IRCCS Istituto Neurologico Carlo Besta, Milan, Italy;* ^*2*^ *Neurology 4 Unit, Neuroimmunology and Neuromuscolar diseases, Fondazione IRCCS Istituto Neurologico Carlo Besta, Milan, Italy*

## EPV‐0333

### Associations between sleep neurophysiology and cognitive performance in rem sleep behavior disorder: Evidence from a wearable PSG device

#### F. Bolengo^1^; L. Dell'Acqua^1^; E. Cini^1^; A. Della Valle^1^; F. Casoni^1^; P. Proserpio^1^; D. Levendowski^2^; A. Galbiati^1^; L. Ferini‐Strambi ^1^

##### ^*1*^ *Department of Clinical Neurosciences, Neurology‐Sleep Disorders Center, IRCCS San Raffaele Scientific Institute, Milan, Italy;* ^*2*^ *Advanced Brain Monitoring, Carlsbad, USA*

## EPV‐0334

### Central gain, peripheral restraint: A cholinergic strategy for Parkinson disease dementia

#### T. Cox ^1^; A. Dixon^2^; H. Randall^3^

##### ^*1*^ *Department of General Medicine, Gold Coast Hospital and Health Service, Gold Coast, Australia;* ^*2*^ *Department of Anaesthetics, Mater Hospital, South Brisbane, Australia;* ^*3*^ *Department of General Medicine, Royal Prince Alfred Hospital, Camperdown, Australia*

## EPV‐0335

### Chronic anti‐NMDAR encephalitis: An unusual course

#### H. El Mouhajir Mohamed; M. Gonzalez Campos; C. Mendez del Barrio

##### Neurology Service, Juan Ramón Jimenez Hospital, Huelva, Spain

## EPV‐0336

### Cognitive function in middle‐aged adults and its differential associations with sedentary behaviour type: Findings from the 1970 British birth cohort

#### L. Al Qadi ^1^; D. Saucier^1^; S. Rayner^2^; J. Pitre^1^; M. Savoie^1^; J. Stadler^1^; J. Jbilou^1^; M. O'Brien^1^

##### ^*1*^ *Faculty of Medicine and Health Sciences, Université de Sherbrooke, Moncton, Canada;* ^*2*^ *School of Physiotherapy, Faculty of Health, Dalhousie University, Halifax, Canada*

## EPV‐0337

### Comorbid psychoemotional disturbances with apathy in Parkinson's disease

#### S. Jumaniyozov; B. Muminov; O. Naimov; R. Matmurodov; S. Jumanazarova; K. Khalimova

##### Tashkent State Medical University, Tashkent, Uzbekistan

## EPV‐0338

### Corneal transplantation–associated probable iatrogenic Creutzfeldt–Jakob disease with cerebellar presentation and myopathic EMG findings

#### A. Yildirim; M. Balci; T. Tombul

##### Neurology, İstanbul Medeniyet University, Göztepe City Hospital, İstanbul, Türkiye

## EPV‐0339

### Creutzfeldt–Jakob disease in north Africa: Insights from a Tunisian case series

#### L. Bouafia; R. Zouari; D. Ben Mohamed; Z. Said; A. Rachdi; F. Nabli; S. Ben Sassi

##### Adult Neurology Department, Mongi‐Ben Hamida National Institute of Neurology, Tunis, Tunisia

## EPV‐0340

### Diagnostic accuracy and clinical utility of imaging and CSF biomarkers in sporadic CJD: A systematic review and meta‐analysis

#### M. Khashman ^1^; A. Saeed^1^; H. Al‐sha'ar^1^; A. Younis^1^; L. Kreshan^1^; H. Al Kayed^2^

##### ^*1*^ *School of Medicine, University of Jordan, Amman, Jordan;* ^*2*^ *Wellstar Cobb Hospital, Department of Internal Medicine, Austell, GA, USA*

## EPV‐0341

### Diagnostic Performance of the Number Connection Test for Minimal Hepatic Encephalopathy

#### D. Podgalo; K. Guliaeva; M. Nadinskaia

##### Sechenov University, Moscow, Russian Federation

## EPV‐0342

### Early cognitive impairment in CADASIL: The value of SDMT, MMSE and MRI findings

#### A. Ismatov; D. Tolibov

##### Department of Neurology, Tashkent State Medical University, Tashkent, Uzbekistan

## EPV‐0343

### Efficacy and safety of perioperative intranasal insulin in preventing postoperative delirium: A systematic review and meta‐analysis of RCTs

#### A. Amin ^1^; A. Mansour^1^; A. Sharkawy^2^; H. Makhlouf^3^; A. Mansour^1^; N. Gamil^4^; A. Suleiman^4^; S. Batarseh^5^; A. Elbataa^1^; M. Abouelmagd^6^

##### ^*1*^ *Faculty of Medicine, Al‐Azhar University, Cairo, Egypt;* ^*2*^ *Faculty of Medicine, South Valley University, Qena, Egypt;* ^*3*^ *Faculty of Medicine, Minia University, Minia, Egypt;* ^*4*^ *Faculty of Medicine, Kafr El‐Sheikh University, Kafr El‐Sheikh, Egypt;* ^*5*^ *Jordan University of Science and Technology, Faculty of Medicine, Irbid, Jordan;* ^*6*^ *Kasr‐alainy Faculty of Medicine, Cairo University, Cairo, Egypt*

## EPV‐0344

### Emotional disorders and quality of life in chronic lymphocytic leukemia

#### S. Jumanazarova; R. Matmurodov; G. Eshchanova

##### Tashkent State Medical University, Tashkent, Uzbekistan

## EPV‐0345

### From in‐ to outpatient: day‐hospital lumbar puncture for shunt prediction in NPH

#### R. Lopes ^1^; C. Guedes Vaz^1^; J. Fins^1^; F. Almeida^2^; R. Batata^3^; I. Laranjinha^1^

##### ^*1*^ *Neurology Department, Unidade Local de Saúde do Santo António, Porto, Portugal;* ^*2*^ *Neuroradiology Department, Unidade Local de Saúde do Santo António, Porto, Portugal;* ^*3*^ *Neurosurgery Department, Unidade Local de Saúde do Santo António, Porto, Portugal*

## EPV‐0346

### Hidden neuropsychological profiles in amyotrophic lateral sclerosis beyond standard cognitive screening

#### A. Radici^1^; A. Panzavolta^1^; D. Pain^2^; C. Lunetta^2^; C. Cerami ^1^

##### ^*1*^ *IUSS Cognitive Neuroscience (ICoN) Center, Scuola Universitaria Superiore IUSS, Pavia;* ^*2*^ *Istituti Clinici Scientifici Maugeri IRCCS, Brain e‐Health Aging (BeA) Laboratory, Department of Neurorehabilitation, Milan, Italy*

## EPV‐0347

### HIV‐associated neurocognitive disorder. A case report

#### L. Quirós Illán; L. Llorente Ayuso; M. Manzano Palomo

##### Neurology, Hospital Universitario Infanta Leonor, Madrid, Spain

## EPV‐0348

### Immediate effects of motor imagery on muscle strength in healthy young adults: A within‐subject study

#### B. Carvalho^1^; A. Patrício^1^; R. Brandão^2^; D. Tomás ^2^

##### ^*1*^ *Department of Physiotherapy, Escola Superior de Saúde Atlântica, Barcarena, Portugal;* ^*2*^ *Department of Physiotherapy, Escola Superior de Saúde do Alcoitão, Alcabideche, Portugal*

## EPV‐0349

### Insulin resistance, subclinical brachiocephalic atherosclerosis, and cognitive performance in young adults

#### A. Militovska ^1^; N. Nekrasova^2^; I. Darii^2^

##### ^*1*^ *Neurology Department, Ivankiv Central District Hospital, Ivankiv, Ukraine;* ^*2*^ *Neurology Department with Neurosurgery Course, Kharkiv National Medical University, Kharkiv, Ukraine*

## EPV‐0350

### Ketogenic dietary intervention in early cognitive decline: A pilot study in mild cognitive impairment and subjective cognitive decline

#### F. Filippi ^1^; S. Tartaglia^2^; I. Cancelli^3^; A. Pittino^1^; C. Del Regno^2^; C. Prezza^1^; N. Ditta^2^; V. Miele^2^; M. Milia^2^; G. Ermanis^1^; G. Gigli^2^; M. Valente^2^

##### ^*1*^ *Department of Medicine (DMED), University of Udine, Udine, Italy;* ^*2*^ *Clinical Neurology Unit, Department of Head‐Neck and Neurosciences, Azienda Sanitaria Universitaria Friuli Centra (ASUFC), Udine, Italy;* ^*3*^ *Neurology Unit, Udine University Hospital, Udine, Italy*

## EPV‐0351

### Mindfulness‐based intervention in idiopathic generalised epilepsy: results of a randomised controlled trial

#### V. Pytelova ^1^; J. Zalud^3^; M. Modrak^2^; P. Marusic^1^; J. Amlerova^1^

##### ^*1*^ *Department of Neurology, Second Faculty of Medicine, Charles University, Motol and Homolka University Hospital, Prague, Czech Republic;* ^*2*^ *Department of Bioinformatics, Second Faculty of Medicine, Charles University, Prague, Czech Republic;* ^*3*^ *Department of Psychology, Motol and Homolka University Hospital, Prague, Czech Republic*

## EPV‐0352

### Neurobehavioral trajectory of eating patterns and body image after bariatric surgery: A 9‐month prospective study

#### K. Kuchkarov; M. Rajapov; B. Vosikov; N. Yadgarova

##### Department of Psychiatry and Narcology, Tashkent State Medical University, Tashkent, Uzbekistan

## EPV‐0353

### Neurocognitive fatigue and burnout phenotypes among physicians in Uzbekistan: Cross‐sectional MBI and MFI‐20 analysis

#### N. Yadgarova; M. Kevorkova; N. Talipova

##### Department of Psychiatry and Narcology, Tashkent State Medical University, Tashkent, Uzbekistan

## EPV‐0354

### Night‐shift load and circadian disruption as drivers of neurocognitive fatigue and burnout in physicians: Cross‐sectional data from Uzbekistan

#### N. Yadgarova; M. Kevorkova; N. Talipova

##### Department of Psychiatry and Narcology, Tashkent State Medical University, Tashkent, Uzbekistan

## EPV‐0355

### Obstructive sleep apnea in adults with down syndrome: prevalence and screening limitations

#### N. Marcos; A. Durán; M. Callejo; A. Lagüela; V. Anciones; Á. López; M. Martínez; L. Pastor; J. Mellada; F. Angoso; A. Rodríguez‐Antigüedad

##### Neurology Department, Cruces University Hospital, Barakaldo, Spain

## EPV‐0356

### Preserved cerebral hemodynamics across gender during an activation task

#### M. Lisak ^1^; A. Junaković Munjas^1^; B. Canik^2^; A. Liampas^3^; M. Roje Bedeković^1^

##### ^*1*^ *Department of Neurology, University Hospital Centar Sestre milosrdnice, Zagreb, Croatia;* ^*2*^ *Kocaeli University Hospital, Kocaeli, Türkiye;* ^*3*^ *The Cyprus Institute of Neurology and Genetics, Nicosia, Cyprus*

## EPV‐0357

### Quality of life in patients with severe acquired brain injury in long‐term care facilities: a multi‐centre cross‐sectional study

#### M. Kluiving^1^; W. van Erp^1^; R. Akkermans^1^; R. Kohnen^3^; H. Anema^2^; K. Jurrius ^1^; D. Gerritsen^1^

##### ^*1*^ *Primary Care, Radboudumc, Nijmegen, the Netherlands;* ^*2*^ *Winkler Kliniek Pro Persona, Wolfheze, the Netherlands;* ^*3*^ *Livio, the Netherlands*

## EPV‐0358

### Recurrent traumatic direct carotid–cavernous fistula: Case report and literature review

#### V. Leka ^1^; E. Enesi^2^; A. Rroji^2^; O. Cibuku^3^; A. Agastra^1^; S. Dodaj^1^; M. Grada^1^; M. Petrela^1^

##### ^*1*^ *Neuroradiology and Neurosurgery Department American Hospital, Tirana, Albania;* ^*2*^ *Neuroradiology Department, Mother Teresa University Hospital Center, Tirana, Albania;* ^*3*^ *Stroke Unit Department, Mother Teresa University Hospital Center, Tirana, Albania*

## EPV‐0359

### Specificity of cognitive disorder structure in patients with advanced post‐traumatic epilepsy

#### I. Abdullayev ^1^; B. Ibadullayev^2^; O. Qadamova^3^; S. Dushamov^4^; D. Kamilova^5^

##### ^*1*^ *Urgench State Medical Instituite, Urgench, Uzbekistan;* ^*2*^ *Urgench State Medical Instituite, Urgench, Uzbekistan;* ^*3*^ *Urgench State Medical Instituite, Urgench, Uzbekistan;* ^*4*^ *Urgench State Medical Instituite, Urgench, Uzbekistan;* ^*5*^ *Urgench State Medical Instituite, Urgench, Uzbekistan*

## EPV‐0360

### The cerebellar stroke, beyond motor manifestations

#### C. Martinez‐Follana; I. Saurina; M. Marin; E. Campanya; M. Garcia‐Huguet; A. Anaya; E. Vadillo; J. Gich; J. Alvarez‐Cienfuegos

##### Neurology Department, Dr Josep Trueta Hospital, Girona, Spain

## EPV‐0361

### The impact of androgen deficiency on neuropsychological status and quality of life in men

#### U. Nabiev; R. Matmurodov

##### Department of Neurology and Medical Psychology, Tashkent State Medical University, Tashkent, Uzbekistan

## EPV‐0362

### The impact of migraine on cognitive processing and central auditory processing: A study evaluating patients' history

#### H. Yaman

##### Health Sciences and Technology Research Institute (SABITA), Regenerative and Restorative Medicine Research Centre (REMER), Clinical Electrophysiology, Neuroimaging and Neuromodulation Laboratory, Istanbul Medipol University, Istanbul, Türkiye

## EPV‐0363

### Toward earlier detection: A delphi Study on non‐vital‐sign early behavioural & cognitive indicators of postoperative delirium in surgical inpatients

#### A. Nazeer Ahamed ^1^; N. Hadi Almuamra^2^; Mustafa Ali^3^

##### ^*1*^ *RAK Medical & Health Sciences University, RAK, UAE;* ^*2*^ *Dubai Medical University, Dubai, UAE;* ^*3*^ *General Surgery in Department of Abu Dhabi , Sheikh Tahnoun Medical City, Al Ain, UAE*

## EPV‐0364

### Value of ptau217 in the differential diagnosis of Alzheimer's disease in a clinical cohort

#### C. Gómez López de San Román; M. Vargas Cobos; A. C de Luca; R. Alfonso González; M. Capra; L. Caballero Sánchez; I. Bermejo Casado; D. Cerdán Santacruz; A. Castrillo Sanz; A. Mendoza Rodriguez

##### Neurology Department, Segovia Hospital

## EPV‐0365

### When biomarkers conflict with the clinic: Positive RT‐QuIC in the absence of clinical Creutzfeldt‐Jakob disease – A case report

#### K. Ntoskas; C. Toilos; O. Sideri; I. Spanou; I. Dedes; A. Tsagkaropoulos; E. Kouremenos

##### Department of Neurology, Hellenic Air Force General Hospital, Athens, Greece

## EPV‐0366

### When cognition leaves a digital footprint: Evidence on passive smartphone‐based biomarkers

#### R. Mesa Martínez; J. Tejada García; L. García Tuñón Villaluenga

##### Neurology, Complejo Asistencial Universitario de León (CAULE), León, Spain

## Coma and chronic disorders of consciousness

## EPV‐0367

### Conscious but confused – What's in a name?

#### W. van Erp^1^; D. Driessen ^2^; L. de Boer^2^; A. van Doremalen^2^; H. Hoogenboezem^3^

##### ^*1*^ *Primary Care, Radboudumc, Nijmegen, the Netherlands;* ^*2*^ *Libra Rehabilitation & Audiology, Tilburg, the Netherlands;* ^*3*^ *Experience Expert, the Netherlands*

## EPV‐0368

### Diagnostic advances for patients with disorders of consciousness: The ultrasound assessment

#### M. Leonardi ^1^; F. Magnani^1^; M. Cacciatore^1^; F. Barbadoro^1^; C. Ippoliti^1^; L. Clementi^1^; M. Stanziano^2^; S. Marino^3^; M. Le Cause^3^; S. De Salvo^3^; R. Talarico^3^; F. La Porta^4^; L. Pometti^4^; B. Garoia^4^; A. Di Gioia^4^; S. Soccorsa^4^; L. Lucca^5^; M. Pugliese^5^; E. Bertoletti^6^; D. Finiguerra^6^; J. Navarro^7^; P. Arcuri^7^; G. Devalle^7^; R. Grisetti^8^; N. Rifino^9^; F. Prada^10^

##### ^*1*^ *SC Neurologia Salute Pubblica Disabilità, Fondazione IRCCS Istituto Neurologico Carlo Besta, Milan, Italy;* ^*2*^ *Neuroradiology Unit, Fondazione IRCCS Istituto Neurologico Carlo Besta, Milan, Italy;* ^*3*^ *IRCCS Centro Neurolesi Bonino Pulejo, Messina, Italy;* ^*4*^ *IRCCS Istituto delle Scienze Neurologiche di Bologna, Bologna, Italy;* ^*5*^ *Intensive Rehabilitation Unit, S. Anna Institute, Crotone, Italy;* ^*6*^ *Presidio Ospedaliero Santa Viola, Bologna, Italy;* ^*7*^ *IRCCS S. Maria Nascente, Fondazione Don Carlo Gnocchi, Milan, Italy;* ^*8*^ *Istituto Palazzolo, Fondazione Don Carlo Gnocchi, Milan, Italy;* ^*9*^ *Neurologia 9 Malattie Cerebrovascolari, Fondazione IRCCS Istituto Neurologico Carlo Besta, Milan, Italy;* ^*10*^ *Department of Neurological Surgery, Fondazione IRCCS Istituto Neurologico Carlo Besta, Milan, Italy*

## EPV‐0369

### EEG‐derived network parameters for the fine grading and economic bridging of cognition‐motor dissociation in DoC

#### T. Bröhl; N. Dissouky; K. Lehnertz; C. Helmstaedter

##### Department of Epileptology, University Hospital Bonn, Bonn, Germany

## EPV‐0370

### Exploring cardiac interoceptive self‐related processes in disorders of consciousness

#### M. Alökten ^1^; L. Hanoğlu^2^

##### ^*1*^ *Atlas Brain Research and Artificial Intelligence Center (A‐BRAIN), Istanbul Atlas University, Istanbul, Türkiye;* ^*2*^ *Department of Neurology, Istanbul Medipol University, Istanbul, Türkiye*

## EPV‐0371

### Family perspectives on prolonged disorders of consciousness after intensive neurorehabilitation: A nationwide qualitative study

#### M. Sharma – Virk; N. Kok; J. Lavrijsen; C. Utens; R. Koopmans; W. van Erp

##### Primary Care, Radboudumc, Nijmegen, the Netherlands

## EPV‐0372

### Higher incidence and prevalence of coma in the USA than in the UK

#### M. Hassani; S. Stückler; M. Amiri; M. Othman; D. Kondziella

##### Department of Neurology, Copenhagen University Hospital, Rigshospitalet, Copenhagen, Denmark

## EPV‐0373

### Specialized long‐term care for severe acquired brain injury in the netherlands: Organizing nationwide networks

#### K. Jurrius; W. van Erp; J. Lavrijsen; R. Koopmans

##### Primary Care, Radboudumc, Nijmegen, the Netherlands

## EPV‐0374

### TMS‐EEG as a unique tool to probe cortical dysconnectivity after severe TBI

#### L. Legostaeva ^1^; S. Casarotto^1^; G. Hassan^1^; G. Furregoni^1^; A. Comanducci^2^; P. Arcuri^2^; J. Navarro^2^; S. Sarasso^1^; M. Rosanova^1^; M. Massimini^1^

##### ^*1*^ *Department of Biomedical and Clinical Sciences, University of Milan, Milan, Italy;* ^*2*^ *IRCCS Fondazione Don Carlo Gnocchi ONLUS, Milan, Italy*

## EPV‐0375

### Value of bedside diagnostic tools for predicting outcome in acute disorders of consciousness

#### J. Zibold; M. Arcidiacono; C. Zeisberger; M. Schmidbauer; K. Dimitriadis

##### Department of Neurology, LMU University Hospital, LMU Munich, Munich, Germany

## Education in neurology

## EPV‐0376

### Anterior insula and dACC as regulatory hubs: Network convergence and brain–behavior links

#### A. Vila ^1^; E. Jetter^1^; B. Carr^2^

##### ^*1*^ *University of Florida College of Medicine, Gainesville, USA;* ^*2*^ *University of Florida Department of Psychiatry, Gainesville, USA*

## EPV‐0377

### Assessing two methods of medical education on prolonged seizures and Rapid and Early Seizure Termination (REST)

#### S. Rohani‐Montez^1^; K. Carpenter^1^; M. Khalaf^1^; C. Scot‐Smith ^1^; N. Gaspard^2^; L. Hirsch^3^; M. Toledo^4^; S. Lattanzi^5^

##### ^*1*^ *Medscape Education Global, London, UK;* ^*2*^ *Hôpital Universitaire de Bruxelles, Université Libre de Bruxelles, Brussels, Belgium;* ^*3*^ *Comprehensive Epilepsy Center, Department of Neurology, Yale University School of Medicine, New Haven, Connecticut, USA;* ^*4*^ *University of Barcelona, Epilepsy Unit, Neurology Department, Valle d'Hebron Hospital Barcelona, Spain;* ^*5*^ *Neurological Clinic, Department of Experimental and Clinical Medicine, Marche Polytechnic University, Ancona, Italy*

## EPV‐0378

### Assessment of nutritional knowledge in patients with multiple sclerosis in Opole and Silesian Province, Poland

#### V. Carbogno‐Barnabe^1^; M. Skrzypek^2^; I. Żabicka ^3^; P. Bednarczyk^3^; B. Łabuz‐Roszak^3^

##### ^*1*^ *Department of Nutrition Related Prevention, Faculty of Public Health in Bytom, Medical University of Silesia in Katowice, Poland;* ^*2*^ *Department of Biostatistics, Department of Epidemiology, Faculty of Public Health in Bytom, Medical University of Silesia in Katowice, Poland;* ^*3*^ *Department of Neurology, St. Jadwiga Provincial Specialist Hospital, Institute of Medical Sciences, University of Opole, Opole, Poland*

## EPV‐0379

### Awareness and perception of neurology as a career choice among medical students in a low‐resource setting

#### J. Oladipupo

##### Department of Medicine, University of Ilorin, Kwara State, Nigeria

## EPV‐0380

### Bridging knowledge and practice: Neurology trainees’ views on functional neurological disorders

#### D. de Pinho; R. Cagigal; A. Mendes

##### Neurology Department, Unidade Local de Saúde de Gaia e Espinho, Portugal

## EPV‐0381

### Bridging the gap: Assessing stroke knowledge disparities among future healthcare professionals in Sudan

#### M. Abdulrahman^1^; Y. Fadlelmoula^1^; I. Hussein^2^; B. Suliman^1^; M. Ibrahim^2^; S. Mokhtar ^2^

##### ^*1*^ *Alzaiem Alazhari University, Khartoum, Sudan;* ^*2*^ *University of Gadarif, Gadarif, Sudan*

## EPV‐0382

### Cinemeducation in neurology: Teaching empathy and bioethics through cinema and narrative ethics

#### M. Pagiantza ^3^; D. Kontogianni^1^; D. Tseligkas^1^; M. Gotsis^4^; E. Koutsouraki^2^; M. Spilioti^2^; V. Kimiskidis^2^

##### ^*1*^ *School of Medicine, Aristotle University of Thessaloniki, Thessaloniki, Greece;* ^*2*^ *First Department of Neurology, AHEPA University General Hospital of Thessaloniki, Aristotle University of Thessaloniki, Thessaloniki, Greece;* ^*3*^ *Department of Neurology, Clinical Neuroscience Center, University Hospital and University of Zurich, Zurich, Switzerland;* ^*4*^ *School of Cinematic Arts, University of Southern California, Los Angeles, CA, USA*

## EPV‐0383

### Computer‐aided localization in neurology: Integrating structured digital assessment with machine learning for automated diagnostic support

#### N. Yuldasheva; N. Odilova

##### Neurology Department, Akfa Medline University Hospital, Tashkent, Uzbekistan

## EPV‐0384

### Determinants of poor adherence to antihypertensive medication among primary hemorrhagic stroke patients in the north Indian population

#### D. Singh ^1^; P. Sengar^2^; A. Sharma^1^; V. Singh^1^; R. Chaurasia^1^

##### ^*1*^ *Department of Neurology, Institute of Medical Sciences, Banaras Hindu University, Varanasi, Uttar Pradesh, India;* ^*2*^ *Department of Pathology, Institute of Medical Sciences, Banaras Hindu University, Varanasi, Uttar Pradesh, India*

## EPV‐0385

### Education on biosimilars and JC virus testing in neurology: Knowledge gains through online CME

#### A. Stan ^1^; L. Fairley^1^; T. Barras^1^; H. Wiendl^2^

##### ^*1*^ *Medscape Education Global, London, UK;* ^*2*^ *Department of Neurology and Neurophysiology Medical Center, University of Freiburg, Freiburg, Germany*

## EPV‐0386

### Education on myasthenia gravis diagnosis results in practice changes and identifies diagnostic barriers

#### S. Rohani‐Montez; M. Khalaf; M. Breuer; G. Adair

##### Medscape Education Global, London, UK

## EPV‐0387

### Emotional knowledge integration through boundary‐spanning search in multiple sclerosis

#### J. Cegarra Sanchez; J. Garcia Granado; A. Relloso de la Fuente; A. Auyanet Matejkova; M. Morera Sanchez; M. Perez Vieitez; F. Cabrera Naranjo; A. Gonzalez Hernandez

##### Neurology Department, Hospital Doctor Negrín, Las Palmas de Gran Canaria, Spain

## EPV‐0388

### Epidemiology and educational implications of neurophobia in Germany, Austria and Switzerland: A trinational study

#### S. Goeschl ^1^; M. Krause^2^; S. Rahiminia^3^; F. Schmidt‐Graf^4^; E. Schaeffer^2^

##### ^*1*^ *School of Public Health, Imperial College London, London, UK;* ^*2*^ *Department of Neurology, University Hospital Schleswig‐Holstein, Kiel, Germany;* ^*3*^ *Department of Neurosurgery, University Hospital Zurich, Zurich, Switzerland;* ^*4*^ *Department of Neurology, München Klinik Bogenhausen, Munich, Germany*

## EPV‐0389

### From data to decision: The librarian–neurologist alliance in neurology education

#### F. Cappelletti ^1^; R. Bergamaschi^2^

##### ^*1*^ *Scientific Library, IRCCS Mondino Foundation, Pavia, Italy;* ^*2*^ *Multiple Sclerosis Centre, IRCCS Mondino Foundation, Pavia, Italy*

## EPV‐0390

### How ReCognition Health (RCH) has fast tracked patients to receive amyloid targeted treatment

#### E. MacSweeney^1^; G. Robinson ^2^; P. Muir^3^

##### ^*1*^ *Dr J Emer MacSweeney, ReCognition Health, London, UK;* ^*2*^ *Dr George Robinson, ReCognition Health, London, UK;* ^*3*^ *Penny Muir, ReCognition Health, London, UK*

## EPV‐0391

### How ReCognition Health (RCH) has fast tracked patients to receive Amyloid Targeted Treatment (ATT)

#### G. Robinson

##### ReCognition Health, London, UK

## EPV‐0392

### Improvements in implementing the latest practical strategies for myasthenia gravis after continuing medical education

#### S. Rohani‐Montez^1^; M. Calle^1^; F. David ^1^; R. Iorio^2^; J. Spillane^3^

##### ^*1*^ *Medscape Education Global, London, UK;* ^*2*^ *Department of Neuroscience, Università Cattolica del Sacro Cuore, Neurology Unit, Fondazione Policlinico Universitario A. Gemelli IRCCS, Rome, Italy;* ^*3*^ *National Hospital for Neurology and Neurosurgery, Guys and St Thomas' NHS Foundation Trust and UCLH, NHS Foundation Trust, London, UK*

## EPV‐0393

### Improving competence in addressing under‐ and over‐treatment in epilepsy through medical education

#### S. Rohani‐Montez ^1^; L. Thevathasan^1^; K. Carpenter^1^; C. Scot‐Smith^1^; R. Thomas^2^; B. Steinhoff^3^; M. Galovic^4^

##### ^*1*^ *Medscape Education Global, London, UK;* ^*2*^ *Newcastle University, Newcastle upon Tyne, UK;* ^*3*^ *University of Freiburg, Freiburg, Germany, Kork Epilepsy Centre, Kehl‐Kork, Germany;* ^*4*^ *Department of Neurology, Clinical Neuroscience Center, University Hospital and University of Zurich, Zurich, Switzerland*

## EPV‐0394

### Improving knowledge, competence and confidence through curriculum‐based continuing medical education in focal epilepsy

#### S. Rohani‐Montez ^1^; L. Thevathasan^1^; K. Carpenter^1^; C. Scot‐Smith^1^; M. Mula^2^; E. Ben‐Menachem^3^; B. Schmitz^4^; B. Steinhoff^5^

##### ^*1*^ *Medscape Education Global, London, UK;* ^*2*^ *St George's University of London, Atkinson Morley Regional Neuroscience Centre, St George's University Hospitals, NHS Foundation Trust, London, UK;* ^*3*^ *Institute for Clinical Neuroscience, Sahlgrenska Academy, Gothenburg University, Gothenburg, Sweden;* ^*4*^ *Department of Neurology, Vivantes Humboldt Hospital, Berlin, Germany;* ^*5*^ *University of Freiburg, Freiburg, Germany Medical Director Kork Epilepsy Centre Kehl‐Kork, Germany*

## EPV‐0395

### Knowledge and attitudes towards Parkinson's disease among the Sudanese population: A nationwide cross‐sectional study

#### S. Mokhtar^1^; M. Abdulrahman ^5^; M. Gasmalha^4^; I. Hussein^1^; A. Balla M. Ahmed^2^; Y. Fadlelmoula^3^; R. MohammedAli^1^

##### ^*1*^ *University of Gadarif, Gadarif, Sudan;* ^*2*^ *University of Khartoum, Khartoum, Sudan;* ^*3*^ *Gazira University, Wad Madani, Sudan;* ^*4*^ *University of Omdurman Islamic, Omdurman, Sudan;* ^*5*^ *Alzaiem Alazhari University, Khartoum, Sudan*

## EPV‐0396

### Medical education on shared decision‐making in epilepsy significantly improves knowledge of key topics to discuss with patients

#### S. Rohani‐Montez ^1^; L. Thevathasan^1^; K. Carpenter^1^; C. Scot‐Smith^1^; E. Ben‐Menachem^2^; B. Schmitz^3^

##### ^*1*^ *Medscape Education Global, London, UK;* ^*2*^ *Institute for Clinical Neuroscience, Sahlgrenska Academy, Gothenburg University, Gothenburg, Sweden;* ^*3*^ *Department of Neurology Vivantes, Humboldt Hospital, Berlin, Germany*

## EPV‐0397

### Medication reconciliation in hospitalized neurology patients: A cross‐sectional study

#### E. Bokri ^1^; M. Romdhan^1^; C. Ben Azzouz^1^; R. Zouari^2^; D. Ben Mohamed^2^; M. Saied^2^; S. Ben Sassi^2^; N. Hasni^1^

##### ^*1*^ *Department of Pharmacy, National Institute Mongi Ben Hmida of Neurologie, Tunis, Tunisia;* ^*2*^ *Department of Neurology, National Institute Mongi Ben Hmida of Neurologie, Tunis, Tunisia*

## EPV‐0398

### Meeting of the minds 2025: A student‐led model for advancing neuroeducation through immersive and inclusive learning

#### A. Alocious; T. Alocious; S. Gentleman

##### Imperial College London, London, UK

## EPV‐0399

### Neuroimmunology matters: Accelerating cumulative learning and improving outcomes through centralised education hubs

#### P. Chen^1^; J. White ^1^; H. Wiendl^2^

##### ^*1*^ *PeerVoice, Amsterdam, the Netherlands;* ^*2*^ *Clinic of Neurology and Neurophysiology, University Hospital Freiburg, Breisgau, Germany*

## EPV‐0400

### Online postgraduate neuronutrition program for neurologists: An interdisciplinary educational model

#### A. Badaeva ^1^; A. Danilov^2^; S. Kotlyarov^3^; I. Pirogova^3^; G. Filatova^4^

##### ^*1*^ *Department for Pathological Physiology, Sechenov University, Moscow, Russian Federation;* ^*2*^ *Department for Nervous Diseases, Institute for Professional Education, Sechenov University, Moscow, Russian Federation;* ^*3*^ *Department for Personalized and Preventive Medicine, Institute for Interdisciplinary Medicine, Moscow, Russian Federation;* ^*4*^ *Department for Clinical Immunology and Allergy, Russian University of Medicine, Russian Federation*

## EPV‐0401

### Patient and public willingness to accept disease‐modifying therapies for Alzheimer's and Parkinson's: Drivers and barriers

#### A. Klein^1^; J. Luker ^2^; K. Fisher^3^

##### ^*1*^ *UCB, Monheim am Rhein, Germany;* ^*2*^ *UCB, Slough, UK;* ^*3*^ *Experience Engineers Ltd, Gerrards Cross, UK*

## EPV‐0402

### Right patient, right pathway: Consultant‐led neurology referral management outcomes from a UK tertiary centre

#### S. Chowdhury; R. Mamun; S. Hussain; S. Sedehizadeh

##### Neurology, Nottingham University Hospitals NHS Foundation Trust, Nottingham, UK

## Environmental Neurology/Climate Change

## EPV‐0403

### Climate change as a disruptor of acute neurology: A surge and squeeze threat to time critical care

#### A. Ramkaran; N. Mahadeosingh

##### School of Medicine, Royal College of Surgeons in Ireland, Dublin, Ireland

## EPV‐0404

### Climatic predisposing factors for posterior circulation aneurysm rupture

#### K. Yarova; Y. Solodovnikova

##### Department of Neurology and Neurosurgery, Odesa National Medical University, Odesa, Ukraine

## EPV‐0405

### Clinical and neuropsychological characteristics of chronic cerebral ischemia in patients living in the Aral environmental crisis region

#### N. Dosbergenova; D. Usmanova

##### ^*1*^ *Department of Neurology, Tashkent State Medical University, Uzbekistan*

## EPV‐0406

### Environmental factors in multiple sclerosis focusing climate change

#### A. Landtblom

##### Department of Medical Sciences, Uppsala University, Uppsala, Sweden

## EPV‐0407

### Increasing incidence of Lyme disease in the Republic of Moldova: National data and potential neurological implications in climate change context

#### E. Mamolea ^1^; V. Lisnic^1^; E. Manole^1^; O. Grosu^2^

##### ^*1*^ *Neurology Department No 1, Nicolae Testemițanu State University of Medicine and Pharmacy; Chisinau, Republic of Moldova;* ^*2*^ *Institute of Neurology and Neurosurgery, Chisinau, Republic of Moldova*

## EPV‐0408

### Neuroplasticity in spaceflight: Adaptive and maladaptive brain changes in microgravity

#### F. Seifar; L. Speicher; D. Herrigel; M. Harrison; M. Freeman

##### Mayo Clinic, Jacksonville, Florida, USA

## EPV‐0409

### Rising temperatures, rising risks: Heatwave effects on Parkinson's disease

#### M. Auffret

##### France Développement Electronique (FDE), Monswiller, France

## Epilepsy

## EPV‐0410

### “The Silent Rust of the Brain”: A case of delayed diagnosis of idiopathic superficial siderosis of the CNS

#### K. Duve; S. Shkrobot; R. Nasalyk

##### I. Horbachevsky Ternopil National Medical University, Ternopil, Ukraine

## EPV‐0411

### “Seizure‐like phenomena (SLP)” in the context of propofol sedation after recovered cardiac arrest: A case report

#### I. Ruiz Salcedo; V. Carmona Bravo; A. Torres Moral; F. Sánchez Fernández; C. Conde Velasco; A. Rincón Valencia; J. Montaner Villalonga; S. Pérez Sánchez

##### Neurology Department, Hospital Universitario Virgen Macarena, Seville, Spain

## EPV‐0412

### A multimodal automated seizure diagnosis model for seizure detection and type classification using video‐electroencephalogram data

#### N. Lin ^1^; Z. Liang^2^; L. Li^2^; W. Gao^1^; Y. Gao^1^; H. Sun^1^; Q. Lu^1^

##### ^*1*^ *Department of Neurology, Peking Union Medical College Hospital, Beijing, China;* ^*2*^ *NetEase Media Technology Co., Ltd., Beijing, China*

## EPV‐0413

### A rare complication of radiotherapy: SMART syndrome

#### S. Dirkeç; İ. Bilgi; S. Üstün Özek

##### Department of Neurology, Prof. Dr. Cemil Taşcıoğlu City Hospital, Istanbul, Türkiye

## EPV‐0414

### A review of the burden of status epilepticus, approaches to its treatment, and any gaps in the Asia‐Oceanic region

#### K. Subramaniam ^1^; P. Purushottamahanti^2^; T. Kumagai^3^

##### ^*1*^ *Viatris, New Zealand;* ^*2*^ *Viatris, India;* ^*3*^ *Viatris, Japan*

## EPV‐0415

### A systematic review of health economic evaluations of treatments for patients with drug‐resistant epilepsy

#### D. Elabbasy ^2^; S. Jowett^1^; A. Gouveia^1^; I. Pokhilenko^1^; S. Evers^2^; M. Majoie^3^; G. van Mastrigt^2^

##### ^*1*^ *Health Economics Unit, Department of Applied Health Sciences, College of Medicine and Health, University of Birmingham, UK;* ^*2*^ *Care and Public Health Research Institute, Department of Health Services Research, Faculty of Health, Medicine and Life Sciences, Maastricht University, Maastricht, the Netherlands;* ^*3*^ *Academic Center for Epileptology (ACE), Maastricht University Medical Center and Kempenhaeghe, Maastricht, Heeze, the Netherlands*

## EPV‐0416

### A systematic review of human status epilepticus in organophosphate poisoning: A real‐world stage 1 plus model?

#### G. Magro; O. Marsico; F. Tosto; L. Rapisarda; G. Bilotti; G. Mastroianni; C. Ermio

##### Neurology Department, Lamezia Terme Hospital, Catanzaro, Italy

## EPV‐0417

### ABCC2 polymorphisms and antiseizure medication resistance: A systematic review and meta‐analysis with power assessment

#### A. Daskeviciute^1^; E. Zaboras ^2^; J. Navalinskas^3^; R. Mameniskiene^1^

##### ^*1*^ *Clinic of Neurology and Neurosurgery, Institute of Clinical Medicine, Faculty of Medicine, Vilnius University, Vilnius, Lithuania;* ^*2*^ *Faculty of Medicine, Vilnius University, Vilnius, Lithuania;* ^*3*^ *Clinic of Cardiac and Vascular Diseases, Institute of Clinical Medicine, Faculty of Medicine, Vilnius University, Lithuania*

## EPV‐0418

### Age and sex disparities in healthcare utilization and costs among epilepsy patients: A population‐based study in Korea

#### E. Jung

##### Chonnam National University Hospital, Gwangju, Korea

## EPV‐0419

### Applications of machine learning in predicting valproic acid pharmacokinetics, safety and treatment response in epilepsy

#### S. Wasti^1^; E. Shafquat ^1^; A. Wasti^2^

##### ^*1*^ *Department of Neurology, King Edward Medical University, Lahore, Pakistan;* ^*2*^ *Department of Neurology, Continental Medical College, Lahore, Pakistan*

## EPV‐0420

### Beyond Drug Resistance: Rethinking Early Surgery in Lesional Focal Epilepsy

#### F. Benninger; I. Goldberg

##### Epilepsy and EEG at Beilinson Hospital, Rabin Medical Center, Petach Tikva, Israel

## EPV‐0421

### Brain arteriovenous malformations and epilepsy – A retrospective analysis from a tertiary centre

#### M. Brás Monteiro ^1^; N. Ramniclal^2^; R. Armindo^2^; A. Santos^3^; A. Sobral‐Pinho^1^; F. Sá^1^

##### ^*1*^ *Serviço de Neurologia, Hospital Egas Moniz, Lisbon, Portugal;* ^*2*^ *Serviço de Neurorradiologia, Hospital Egas Moniz, Lisbon, Portugal;* ^*3*^ *Serviço de Neurocirurgia, Hospital Egas Moniz, Lisbon, Portugal*

## EPV‐0422

### Clinical and neurophysiological diagnosis and surgical treatment of post‐traumatic drug‐resistant temporal lobe epilepsy

#### S. Kravtsova; A. Vasilenko; A. Ulitin

##### Almazov National Medical Research Centre, St. Petersburg, Russian Federation

## EPV‐0423

### Clinical characteristics and outcomes of patients with refractory/super refractorry status epilepticus: A retrospective analysis

#### D. Çoban; K. Aslan‐Kara; T. Demir; Ş. Bıçakçı

##### Çukurova University Balcalı Hospital Department of Neurology, Adana, Türkiye

## EPV‐0424

### Clinical characteristics, etiology, and outcomes of motor Epilepsia Partialis continua: A 12‐patient cohort study

#### S. Dirkeç; S. Üstün Özek

##### Department of Neurology, Prof. Dr. Cemil Taşcıoğlu City Hospital, Istanbul, Türkiye

## EPV‐0425

### Clinical correlates of depressive symptoms and the treatment gap of depression among adults with epilepsy

#### S. Kasradze

##### Caucasus International University, Tbilisi, Georgia

## EPV‐0426

### Clinical outcomes of adult‐initiated cenobamate in developmental and epileptic encephalopathy versus other drug‐resistant epilepsies

#### D. Rivero‐Rodríguez; R. Almansa‐Castillo; C. Cabeza‐Alvarez

##### Neurology department, Complejo Hospitalario Universitario de Toledo, Spanish Ministry of Health, Toledo, Spain

## EPV‐0427

### Clinical, EEG and CSF predictors of outcome in status epilepticus: A retrospective study

#### K. Stanzelova; M. Kalinova; P. Marusic; D. Krysl

##### Department of Neurology, Second Faculty of Medicine, Charles University, Motol and Homolka University Hospital, Prague

## EPV‐0428

### Comparative analysis of wearable machine learning–based and EEG‐based seizure detection methods

#### G. Bambal

##### University of Buckingham, Buckinghamshire, UK

## EPV‐0429

### Comparative performance of the Montreal cognitive assessment and the mini‐mental state examination for cognitive screening in patients with epilepsy

#### R. Ben Dhia; L. Bouafia; M. Mhiri; N. Daoussi; M. Frih Ayed

##### Neurology department, Fatouma Bourguiba Hospital Monastir, Tunisia

## EPV‐0430

### Comparison of prognostic score performance in refractory status epilepticus treated with continuous intravenous anesthetic drugs at 90 days

#### D. Rivero‐Rodríguez ^1^; Y. Pernas‐Sánchez^2^; C. Cabeza‐Alvarez^1^; D. DiCapua‐Sacoto^3^; G. Pluck^4^; M. Morales Casado^1^

##### ^*1*^ *Neurology Department, Complejo Hospitalario Universitario de Toledo, Spanish Ministry of Health, Toledo, Spain;* ^*2*^ *Oncology department, Hospital Universitario Fundacion Alcorcon, Spanish Ministry of Health, Madrid, Spain;* ^*3*^ *Department of Neurology, Hospital de Especialidades Eugenio Espejo, Ministry of Public Health of Ecuador, Quito, Ecuador;* ^*4*^ *Department of Psychology, College of Human Sciences and Education, KIMEP University, Almaty, Kazakhstan*

## EPV‐0431

### Correlation of EEG findings outcome in Juvenile Myoclonic Epilepsy (JME) – A multi‐centric retrospective study

#### R. Bansal ^1^; A. Bansal^1^; C. Rathore^2^; N. Baheti^3^

##### ^*1*^ *Institute of Neurosciences, Medanta‐The Medicity, Gurugram, India;* ^*2*^ *Neurology Department, Zydus Hospital, Vadodara, India;* ^*3*^ *Neurology Department, Dr. Gm Taori Ciims Hospital, Nagpur, India*

## EPV‐0432

### Deep brain stimulation in drug‐resistant epilepsy: Our experience in clinical practice

#### E. Cañada Lahoz ^1^; P. Iriarte Uribe‐Echeverria^1^; A. Vieira Campos^1^; S. Lozano Veiga^1^; R. Berbegal Serralta^1^; M. Navas^2^; P. Pulido^2^; C. Torres^2^; L. Vega^3^; J. Pastor^3^; M. De Toledo Heras^1^

##### ^*1*^ *Neurología, Hospital Universitario de La Princesa, Madrid, Spain;* ^*2*^ *Neurocirugía, Hospital Universitario de La Princesa, Madrid, Spain;* ^*3*^ *Neurofisiología, Hospital Universitario de La Princesa, Madrid, Spain*

## EPV‐0433

### Detection of anxiety disorders in tension‐type headache and evaluation of the effectiveness of cognitive behavioral therapy

#### S. Gafurova; N. Maxamatjanova; S. Yusuphodjayeva; G. Ernayeva

##### Tashkent State Medical University, Neurology and Medical Psychology Department, Tashkent, Uzbekistan

## EPV‐0434

### Detection of anxiety–phobic disorders in epilepsy and evaluation of the effectiveness of medical‐psychological intervention

#### S. Gafurova; Z. Ibodullayev; N. Maxamatjanova; S. Yusuphodjayeva; G. Ernayeva

##### Tashkent State Medical University, Neurology and Medical Psychology Department, Uzbekistan, Tashkent

## EPV‐0435

### Dynamics of epilepsy in patients with supratentorial brain ventricular system tumours depending on surgical approach

#### S. Ahmedov ^1^; R. Egamberdiev^1^; B. Muminov^2^

##### ^*1*^ *Republican Specialized Scientific and Practical Medical Center of Neurosurgery;* ^*2*^ *Tashkent State Medical University, Tashkent, Uzbekistan*

## EPV‐0436

### Early cenobamate as a third‐line option in drug‐resistant focal epilepsy: A paradigm shift?

#### F. Dono ^1^; M. Russo^1^; R. Agosto^1^; G. Evangelista^1^; B. Nucera^2^; G. Falcicchio^3^; S. Sensi^1^

##### ^*1*^ *Department of Neuroscience, Imaging and Clinical Science, “G. d'Annunzio” University of Chieti‐Pescara, Chieti, Italy;* ^*2*^ *Department of Neurology, Hospital of Merano (SABES‐ASDAA), Merano, Italy;* ^*3*^ *Department of Translational Biomedicine and Neurosciences, University of Bari ‘Aldo Moro’, Bari, Italy*

## EPV‐0437

### Early interventions for children with epileptic encephalopathies: a systematic review

#### T. Siriwardana ^1^; C. Perera^1^; K. Lakmal^2^; D. Hettiarachchi^3^

##### ^*1*^ *Faculty of Medicine, University of Colombo, Sri Lanka;* ^*2*^ *Post Graduate Institute of Medicine, University of Colombo, Sri Lanka;* ^*3*^ *Department of Anatomy, Genetics and Biomedical Informatics Faculty of Medicine, University of Colombo, Sri Lanka*

## EPV‐0438

### EEG‐VL: Integrating visual features with large language models for automated seizure detection

#### N. LIN ^1^; Z. Liang^2^; Q. Lu^1^; W. Gao^1^; H. Sun^1^; Y. Gao^1^; Y. Dong^2^

##### ^*1*^ *Department of Neurology, Peking Union Medical College Hospital, Beijing, China;* ^*2*^ *NetEase Media Technology Co., Ltd., Beijing, China*

## EPV‐0439

### Effects of seizure frequency, EEG and MRI findings on quality of life and psychological burden in epilepsy

#### T. Fidan Çolak; S. Şen

##### Department of Neurology, Faculty of Medicine, Ondokuz Mayıs University, Samsun, Türkiye

## EPV‐0440

### Efficacy and safety of adjunctive cenobamate in patients with intellectual disability: A real world experience

#### A. De Matteis; A. Morano; E. Cerulli Irelli; M. Borioni; C. Di Bonaventura; C. Panzini; A. Mazzeo; A. D'Aniello; G. De Gennaro

##### Sapienza University of Rome, Rome, Italy

## EPV‐0441

### Efficacy and safety of omega‐3 fatty acid supplementation on seizure outcomes in epilepsy: A systematic review and meta‐analysis

#### K. Alidreesi; G. Sindi; A. Bamehriz; M. Almutairy; M. Bukari; S. Alqhtani; S. Makki; S. Makkawi

##### College of Medicine, King Saud bin Abdulaziz University for Health Sciences, Jeddah, Kingdom of Saudi Arabia

## EPV‐0442

### Epilepsy‐on‐a‐chip: In vitro platforms for seizure mechanisms and drug discovery

#### M. Nasir ^1^; A. Sheshadri^2^; M. Jaber^3^; Y. Canpolat^4^

##### ^*1*^ *Department of Medicine, Ivane Javakhishvili Tbilisi State University, Tbilisi, Georgia;* ^*2*^ *Department of Medicine, Ivane Javakhishvili Tbilisi State University, Tbilisi, Georgia;* ^*3*^ *School of Health Sciences, University of Georgia, Tbilisi, Georgia;* ^*4*^ *Department of Medicine, Dokuz Eylul University, Izmir, Türkiye*

## EPV‐0443

### Eslicarbazepine acetate exposure in pregnant women with epilepsy: From clinical development to 16 years post‐marketing experience

#### D. Lopes; V. di Foggia; C. Meneses; C. Sousa; J. Holenz; H. Gama

##### Bial‐Portela & Cª, S.A, Porto, Portugal

## EPV‐0444

### Facial emotion recognition in focal epilepsy: Localization is not the main factor

#### M. Grange

##### *Épileptologie, La Pitié Salpetrière*, *Paris, France*

## EPV‐0445

### Ferroptosis as a key mechanism in the pathogenesis of symptomatic epilepsy

#### R. Azizova; A. Abboskhonov; F. Mallaev

##### Tashkent State Medical University, Tashkent, Uzbekistan

## EPV‐0446

### Focal epilepsy with variable foci. Relevance of genetics in epilepsy

#### G. Torres Sánchez; H. El Mouhajir Mohamed; M. González Campos; A. Tapia Barrantes; J. Mederos Hernández; G. Velamazán Delgado

##### Juan Ramón Jiménez Hospital, Huelva, Spain

## EPV‐0447

### Focal myoclonic status epilepticus without coma presenting as perioral and cervical myoclonias in an elderly patient

#### L. Dominguez‐Jimenez; M. Garcia‐Ruiz; E. Lopez‐Valdes; A. Lafuente‐Almaden; P. Abizanda‐Saro; M. Alvarez‐Gutierrez; J. Melgar; M. Bonnin‐Bravo; P. Mayo‐Rodriguez

##### Neurology Department, Hospital Clínico San Carlos, Madrid, Spain

## EPV‐0448

### From benign infantile epilepsy to paroxysmal kinesigenic dyskinesia: The clinical spectrum of PRRT2‐related disorder

#### C. Hurtado Alcázar; R. Calle Calle; C. Santillana Ávila; Á. Morales Lahoz; I. Del Pino Díaz

##### Department of Neurology, San Cecilio Universitary Hospital, Granada, Spain

## EPV‐0449

### Genetic generalized epilepsy: phenotypic characterization and prognostic factors

#### L. Giordano ^2^; E. Cerulli Irelli^1^; A. Morano^1^; A. Mazzeo^1^; F. Giaculli^2^; M. Borioni^1^; P. Moro^1^; A. Giallonardo^1^; C. Di Bonaventura^1^

##### ^*1*^ *Department of Human Neurosciences, Sapienza University, Rome, Italy;* ^*2*^ *Institute of Research and Medical Care Neuromed, Pozzilli, Italy*

## EPV‐0450

### Gray matter heterotopia in adults: Clinical and radiologic features across morphologic subtypes

#### T. Cetindamar ^1^; I. Cetindamar^1^; E. Toplutas^1^; G. Ertan Akan^2^; L. Hanoglu^1^

##### ^*1*^ *Department of Neurology, Istanbul Medipol University, Istanbul, Türkiye;* ^*2*^ *Department of Radiology, Istanbul Medipol University, Istanbul, Türkiye*

## EPV‐0451

### Identification of risk factors and evaluation of clinical and electrophysiological features in epilepsy‐related psychosis

#### T. Talıbov ^1^; M. Inci^2^; G. Aliyeva^2^; O. Kızek^2^; A. Elmalı Yazıcı^2^; F. Uslu^2^; N. Bebek^2^

##### ^*1*^ *Istinye University, Topkapi LIV Hospital, Istanbul, Türkiye;* ^*2*^ *Istanbul University, Istanbul Faculty of Medicine, Department of Neurology, Istanbul, Türkiye*

## EPV‐0452

### Inflammatory‐micronutrient phenotype in epilepsy: Associations with EEG abnormalities, and seizure burden in an outpatient cohort

#### C. Rustamova ^1^; M. Yakubova^2^

##### ^*1*^ *Medion Innovation hospital, Department of Neurology, Uzbekistan;* ^*2*^ *Tashkent State Medical University, Department of Neurology and Medical Psychology, Uzbekistan*

## EPV‐0453

### Inherited instability of sleep architecture creating a light‐NREM seizure window in juvenile myoclonic epilepsy

#### M. Lamba; S. Roshan; V. Puri; N. Chaudhry

##### Department of Neurology, Govind Ballabh Pant Institute of Postgraduate Medical Education and Research, New Delhi, India

## EPV‐0454

### Lamotrigine monotherapy as an effective treatment option in drug‐resistant epilepsy associated with tuberous sclerosis complex

#### S. Shokhimardonov

##### Tashkent State Medical University, Tashkent, Uzbekistan

## EPV‐0455

### Late‐onset Rasmussen encephalitis, should early hemispherectomy be considered?

#### B. Jové; M. Olivera; O. Brengaret; N. Bennouna; M. Ruiz‐Vives; J. Tutusaus; E. Conde‐Blanco; M. Centeno; M. Carreño

##### Neurology, Hospital Clínic, Barcelona, Spain

## EPV‐0456

### Lennox–Gastaut syndromes secondary to chromosome 15 rearrangement: description of a case series and response to cannabidiol treatment

#### M. Callejo ^1^; A. Marinas^1^; P. Ceballos^2^; I. Garamendi^1^; I. Escalza^3^; A. Moreno^4^; M. Horvarth^5^; M. Martínez^1^; Á. López^1^; N. Marcos^1^; F. Angoso^1^; L. Pastor^1^; J. Mallada^1^; A. Rodriguez‐Antigüedad^1^

##### ^*1*^ *Neurology, Cruces University Hospital, Barakaldo, Spain;* ^*2*^ *Neurology, Laredo Hospital, Laredo, Spain;* ^*3*^ *Neurology, Galdakao University Hospital, Galdakao, Spain;* ^*4*^ *Neurology, Urduliz Hospital, Urduliz, Spain;* ^*5*^ *Neurophysiology, Cruces Hospital, Barakaldo, Spain*

## EPV‐0457

### Levetiracetam for new onset epilepsy in older adults: A 24 week prospective cohort study

#### A. Quka; I. Zekja; D. Disha; A. Hoxha; A. Kuqo; J. Kruja

##### Department of Neurology, UHC Mother Teresa, Tirana, Albania

## EPV‐0458

### Levetiracetam for seizure prophylaxis post‐neurosurgery: Prescription patterns and ADR monitoring

#### R. Baxi; P. Marathe; S. Kamat; S. Mishra; J. Jha; T. Nadkarni; D. Muzumdar

##### Seth GS Medical College & KEM Hospital, Mumbai

## EPV‐0459

### Machine learning‐based prediction of post‐stroke epilepsy

#### C. Chou ^1^; S. Peng^2^

##### ^*1*^ *Department of Neurology, Neurological Institute, Taipei Veterans General Hospital, Taipei, Taiwan;* ^*2*^ *Professional Master Program in Artificial Intelligence in Medicine, College of Medicine, Taipei Medical University, Taipei, Taiwan*

## EPV‐0460

### Multifocal epileptiform activity as an independent predictor of mortality in hospitalized patients

#### D. Carapinha; J. Silva; P. Neves; A. Martins; J. Peres

##### Neurology Department, ULS Amadora/Sintra, Portugal

## EPV‐0461

### Multimodal AI decision support for epilepsy surgery under functional uncertainty

#### T. Alecu

##### Department of Neurosurgery, County Emergency Clinical Hospital Sibiu, Sibiu, Romania

## EPV‐0462

### Next‐generation sequencing: application for Kazakhstani patients with epilepsy

#### A. Kassenova ^1^; A. Gabdulkayum^1^; M. Bayanova^2^; U. Kairov^1^; A. Akilzhanova^1^; D. Yerezhepov^1^

##### ^*1*^ *Center for Life Sciences, National Laboratory Astana, Nazarbayev University, Astana, Kazakhstan;* ^*2*^ *National Scientific Center for Mother and Child Health, University Medical Center, Astana, Kazakhstan*

## EPV‐0463

### NGS‐based identification of genetic variants potentially influencing AED response in pediatric epilepsy

#### D. Yerezhepov ^1^; A. Gabdulkayum^1^; M. Bayanova^2^; U. Kairov^1^; A. Akilzhanova^1^

##### ^*1*^ *Center for Life Sciences, National Laboratory Astana, Nazarbayev University, Astana, Kazakhstan;* ^*2*^ *National Scientific Center for Maternal and Child Health, University Medical Center, Astana, Kazakhstan*

## EPV‐0464

### Optimization of treatment strategy in DEE‐SWAS: Role of early hormonal therapy

#### S. Shokhimardonov

##### Tashkent State Medical University, Tashkent, Uzbekistan

## EPV‐0465

### Post‐encephalitic epilepsy after autoimmune encephalitis

#### A. Morgadinho; T. Santana; I. Marques

##### Neurology Department, Unidade Local de Saúde de Almada‐Seixal, Almada, Portugal

## EPV‐0466

### Preoperative and postoperative evaluation of cognitive functions in patients undergoing temporal and extratemporal epilepsy surgery

#### G. Aliyeva^1^; D. Özkaptan^2^; P. İşçen^1^; Ö. Kizek ^1^; A. Elmalı^1^; N. Şirin İnan^1^; F. Uslu İlgen^1^; N. Bebek^1^

##### ^*1*^ *Istanbul University, Istanbul Faculty of Medicine, Department of Neurology, Istanbul, Türkiye,* ^*2*^ *University of Health Sciences Kartal Dr Lütfi Kırdar City Hospital, Department of Neurology, Istanbul, Türkiye*

## EPV‐0467

### Prevalence of depression in patients with epilepsy and evaluation of the effectiveness of medical‐psychological interventions

#### S. Gafurova

##### Neurology, Tashkent State Medical University, Tashkent, Uzbekistan

## EPV‐0468

### Psychiatric adverse‐event monitoring in epilepsy: Lesson from a systematic literature review of eslicarbazepine acetate, lacosamide, and levetiracetam

#### V. Villanueva^1^; M. Mula^2^; S. Rheims^3^; M. Comellas^4^; L. Lizán^4^; L. Esteban^4^; M. Rodriguez^5^; V. Di Foggia ^6^

##### ^*1*^ *Hospital Universitario y Politécnico La Fe, Valencia, Spain. Member of ERN EPICARE;* ^*2*^ *Atkinson Morley Regional Neuroscience Centre, St George's University Hospital NHS Foundation Trust, London, UK;* ^*3*^ *Department of Functional Neurology and Epileptology, Hospices Civils de Lyon and University of Lyon, Lyon, France; Lyon's Neuroscience Research Center, INSERM U1028/CNRS UMR 5292 and Lyon 1 University, Lyon, France; Epilepsy Institute, Lyon, France;* ^*4*^ *Outcomes'10, Castellón, Spain;* ^*5*^ *Laboratorios Bial S.A., Spain;* ^*6*^ *Bial – Portela CA, SA, Portugal*

## EPV‐0469

### Pyridoxine for preventing and treating levetiracetam‐induced behavioural and neuropsychiatric adverse effects in patients with epilepsy

#### S. Wasti^1^; A. Wasti^2^; M. Iqbal^1^; M. Shahid^1^; M. Javed ^1^

##### ^*1*^ *Department of Neurology, King Edward Medical University, Lahore, Pakistan;* ^*2*^ *Department of Neurology, Continental Medical College, Lahore, Pakistan*

## EPV‐0470

### Quality of life, self stigma, and associated factors in patients with epilepsy in Colombo, Sri Lanka

#### A. Rajendiran ^1^; I. Abhimani^1^; H. Adikari^1^; A. Ahamed^1^; Y. Rajapakse^2^

##### ^*1*^ *Faculty of Medicine, University of Colombo, Sri Lanka;* ^*2*^ *Department of Anatomy, Genetics and Biomedical Informatics, Faculty of Medicine, University of Colombo, Sri Lanka*

## EPV‐0471

### Rasmussen encephalitis: clinical spectrum, complementary investigations, and therapeutic strategies from a case series

#### L. Amador‐Castilla ^1^; D. Campos‐Fernández^1,2^; M. Toledo^1,2^; E. Santamarina^1,2^; M. Garcés^2^; M. Quintana^2^; E. Fonseca^1,2^; M. Sueiras‐Gil^3,4^; V. Thonon^3^; S. Sarria Estrada^5^; P. Francis Schell^5^; L. Abaira^1,2^

##### ^*1*^ *Epilepsy Unit, Neurology Department, Medicine Department, Vall d’Hebron University Hospital, Barcelona, Spain,* ^*2*^ *Research group on Status Epilepticus and Acute Seizures, Vall d'Hebron Research Institute (VHIR), Vall d’Hebron University Hospital, Barcelona, Spain,* ^*3*^ *Neurophysiology Department, Vall d'Hebron University Hospital, Barcelona, Spain,* ^*4*^ *Neurotraumatology and Neurosurgery Research Unit (UNINN), Vall d’Hebron Research Institute (VHIR), Barcelona, Spain,* ^*5*^ *Neuroradiology Unit, Radiology Department, Medicine Department, Vall d'Hebron University Hospital, Barcelona, Spain*

## EPV‐0472

### Real‐life experience of switching from branded to generic eslicarbazepine acetate

#### Y. Winter ^1^; T. Hammen^2^; S. Groppa^1^; Y. Koo^3^; C. Meudt^4^

##### ^*1*^ *Department of Neurology, Saarland University Medical Center, University of Saarland, Homburg, Germany;* ^*2*^ *Clinic for Neurology, Westpfalz‐Klinikum Kaiserslautern, Kaiserslautern, Germany;* ^*3*^ *Department of Neurology, Asan Medical Center, University of Ulsan College of Medicine, South Korea;* ^*4*^ *Department of Pediatrics, Frankfurt Hoechst Hospital, Frankfurt, Germany*

## EPV‐0473

### Refractory status epilepticus in posterior reversible encephalopathy syndrome resolved with perampanel after failure of barbiturate coma

#### A. Herrera Ortega; M. Muñoz Pasadas; J. Vaamonde Gamo; P. Gómez Ramírez; A. Sánchez Gómez; M. El Harmochi Daoud; V. Ros Escolar; M. Carrillo Carrillo

##### Department of Neurology, General University Hospital of Ciudad Real, Ciudad Real, Spain

## EPV‐0474

### Retrospective prescription based analysis of levetiracetam usage in various types of epilepsies in Indian outpatient's settings

#### A. Jose ^1^; D. Ruikar^2^; G. Chaudhary^3^; P. Mate^1^; G. Chavan^1^

##### ^*1*^ *Lupin Pharma Ltd, Mumbai, India;* ^*2*^ *MIMSR Medical College, Latur, India;* ^*3*^ *HBT Medical College and RN Cooper Hospital, Mumbai, India*

## EPV‐0475

### Routine EEG versus 24‐hour EEG in the discontinuation of anti‐seizure medications in epileptic patients with long‐term seizure freedom

#### J. Almada Silva; S. Bernardo; P. Lopes das Neves; A. Martins; J. Peres

##### Neurology Department of Unidade Local de Saúde Amadora/Sintra, Portugal

## EPV‐0476

### Seizure control in mesiotemporal epilepsy after 6 months of treatment by means of focal cortical stimulation

#### Y. Winter ^1^; D. Keiner^2^; J. Federspiel^1^; T. Hammen^3^; M. Glaser^4^; J. Oertel^2^; S. Groppa^1^

##### ^*1*^ *Department of Neurology, Saarland University Medical Center, Homburg, Germany;* ^*2*^ *Department of Neurosurgery, Saarland University Medical Center, Homburg, Germany;* ^*3*^ *Clinic for Neurology, Westpfalz‐Klinikum Kaiserslautern, Kaiserslautern, Germany;* ^*4*^ *Department of Neurosurgery, University Medical Center of the Johannes Gutenberg University Mainz, Mainz, Germany*

## EPV‐0477

### Seizure frequency as a determinant of cognitive impairment severity in epilepsy

#### M. Ibodullaeva; K. Daminova

##### Department of Neurology and Clinical Psychology, Tashkent State Medical University, Tashkent, Uzbekistan

## EPV‐0478

### Seizure worsening as a sentinel sign of penicillamine‐induced aplastic anemia in wilson disease

#### A. Manole; R. Rotaru; D. Chițimuș; N. Lupescu; I. Georgescu; F. Pleșa; C. Sîrbu

##### Neurology Department, Carol Davila University Central Emergency Military Hospital, Bucharest, Romania

## EPV‐0479

### Seizures reported under stroke code: What is truly stroke?

#### C. Erkalayci Kerekli; H. Komurcu

##### Neurology Department, Health Sciences University Fatih Sultan Mehmet Training and Research Hospital, Istanbul, Türkiye

## EPV‐0480

### Abstract withdrawn

## EPV‐0481

### Sex‐specific differences in presentation, management and outcomes in status epilepticus: A retrospective cohort study

#### L. Möller^1^; J. Winkel^1^; C. Jünemann ^1^; P. Tsalouchidou^2^; L. Habermehl^1^; K. Menzler^1^; S. Knake^1^

##### ^*1*^ *Philipps University Marburg, Department of Neurology, Epilepsy Center Hessen, Marburg, Germany;* ^*2*^ *Second Department of Neurology, “Attikon” University Hospital, School of Medicine, National and Kapodistrian University of Athens, Athens, Greece*

## EPV‐0482

### Siamese‐ES: A multi‐task learning framework leveraging sleep features for interictal epileptiform discharge detection

#### W. Gao ^1^; N. Lin^1^; P. Hu^2^; L. Li^2^; Q. Lu^1^; H. Sun^1^

##### ^*1*^ *Department of Neurology, Peking Union Medical College Hospital, Beijing, China;* ^*2*^ *NetEase Media Technology Co., Ltd., Beijing, China*

## EPV‐0483

### Status epilepticus: Critical review and experience. First step towards an effective protocol

#### M. Martínez Palicio; D. Alonso Vallín; A. O Sanchez; A. Sanchez Rodriguez; M. Sanchez‐Suárez López

##### Hospital Universitario de Cabueñes, Gijón, Spain

## EPV‐0484

### The double twenty: Unmasking a rare chromosomal epilepsy in a young adult

#### B. Chelaru; S. Petrescu; C. Gheti; M. Martoiu; A. Daneasa; C. Panea

##### Neurology, Elias Emergency University Hospital, Bucharest, Romania

## EPV‐0485

### The epidemiology, cultural perceptions, and management of epilepsy in roma communities: A systematic review

#### S. Wasti^1^; A. Wasti^2^; S. Chattha ^1^

##### ^*1*^ *Department of Neurology, King Edward Medical University, Lahore, Pakistan;* ^*2*^ *Department of Neurology, Continental Medical College, Lahore, Pakistan*

## EPV‐0486

### The role of stress sensitivity in the genetics of juvenile myoclonic epilepsy

#### D. Pal ^1^; E. Sanders^2^; L. Strug^2^

##### ^*1*^ *Basic and Clinical Neuroscience, King's College London, London, UK;* ^*2*^ *University of Toronto, Toronto, Canada*

## EPV‐0487

### Circadian distribution of seizures and their impact on sleep structure in adults with epilepsy

#### L. Atabekyan ^1^; E. Balian^1^; N. Nadryan^2^; H. Hovakimyan^1^; S. Khachatryan^2^

##### ^*1*^ *Somnus Neurology Clinic, Armenia;* ^*2*^ *National Institute of Health, Armenia*

## EPV‐0488

### Transforming care for people with tuberous sclerosis complex: A collaborative, nurse‐led evolution toward a national multidisciplinary model

#### C. Behan ^1^; M. Vasseghi^5^; A. Gough^2^; L. McEvoy^2^; S. McAnallen^3^; C. Kennedy^3^; D. Ferguson^2^; C. Doherty^4^

##### ^*1*^ *School of Nursing and Midwifery, RCSI University of Medicine and Health Sciences, Dublin, Ireland;* ^*2*^ *Neurology Department, St. James's Hospital, Dublin, Ireland;* ^*3*^ *Nephrology Department, St. James's Hospital, Dublin, Ireland;* ^*4*^ *Academic Unit of Neurology, Trinity College Dublin, Ireland;* ^*5*^ *TSC Ireland, Dublin, Ireland*

## EPV‐0489

### Transition of care from paediatric to adult epilepsy services in malta: A clinical review

#### K. Micallef; M. Miruzzi; M. Mallia

##### Department of Neurosciences, Mater Dei Hospital, Msida, Malta

## EPV‐0490

### Use of cenobamate in a patient with super‐refractory status epileticus

#### R. Alves Simões ^1^; B. Machado Antunes^1^; A. Patrícia Antunes^1,2^; A. Rita Peralta^1,2,3,4^; S. Parreira^1,3,4^; C. Bentes^1,2,3,4^; L. Albuquerque^1,2^

##### ^*1*^ *Neurology Service, Department of Neuroscience and Mental Health, Santa Maria Hospital, Santa Maria Local Health Unit, Lisbon, Portugal;* ^*3*^ *EEG Laboratory/Sleep Disorders, Neurology Service, Department of Neuroscience and Mental Health, Santa Maria Hospital, Santa Maria Local Health Unit, Lisbon, Portugal;* ^*4*^ *Reference Center for Refractory Epilepsies (EpiCARE Member), Neurology Service, Department of Neuroscience and Mental Health, Santa Maria Hospital, Santa Maria Local Health Unit, Lisbon, Portugal*

## EPV‐0491

### Vagus nerve stimulation for RSE/SRSE: Systematic review of survival and seizure freedom outcomes

#### G. Evangelista; M. Di Pietro; F. Dono; R. Agosto; M. Russo; S. Sensi

##### Department of Neuroscience, Imaging and Clinical Science, “G. d’Annunzio” University of Chieti‐Pescara, Chieti, Italy

## EPV‐0492

### Velopharyngeal myoclonic status as a manifestation of cortical stroke

#### D. Arcila Salazar ^1^; B. del Moral Sahuquillo^1^; Á. Antón Conejos^1^; M. Fernández Cotero^2^

##### ^*1*^ *Neurology, HCU Lozano Blesa, Zaragoza, Spain;* ^*2*^ *Otorhinolaryngology, HCU Lozano Blesa, Zaragoza, Spain*

## EPV‐0493

### Where do young individuals with epilepsy die? A study of mortality locations in patients under 18

#### S. Wasti ^1^; A. Wasti^2^

##### ^*1*^ *Department of Neurology, King Edward Medical University, Lahore, Pakistan;* ^*2*^ *Department of Neurology, Continental Medical College, Lahore, Pakistan*

## Ethics in neurology

## EPV‐0494

### Decision‐making capacity and safeguards in voluntary assisted dying for neurodegenerative disorders

#### C. Gericke

##### School of Medicine, Australian National University, Canberra, Australia & Canberra Epilepsy Specialist Centre, Deakin Private Hospital, Canberra, Australia

## EPV‐0495

### Epistemic authority in adaptive DBS: When neural metrics compete with lived experience

#### A. Vila^1^; E. Mejia ^1^; B. Carr^2^

##### ^*1*^ *University of Florida College of Medicine, Gainesville, FL, USA;* ^*2*^ *University of Florida Department of Psychiatry, Gainesville, FL, USA*

## EPV‐0496

### Ethical and Governance Challenges in Neurotechnology: Implications for Clinical Neurology

#### T. Roth ^1^; M. Ienca^1^; L. Cabrera^2^; E. Schiller^1^; J. Illes^3^

##### ^*1*^ *Laboratory of Ethics of AI & Neuroscience, Institute for History and Ethics in Medicine, Technical University of Munich, Munich, Germany;* ^*2*^ *Neuroethics Lab, Rock Ethics Institute, and Department of Engineering Science and Mechanics, Pennsylvania State University, University Park, USA;* ^*3*^ *National Core for Neuroethics, Division of Neurology, Department of Medicine, University of British Columbia, Vancouver, Canada*

## EPV‐0497

### Presymptomatic disease‐modifying therapies in neurodegenerative disorders: Clinical promise and ethical challenges

#### A. Klein^1^; J. Luker ^2^; R. Harris^3^; J. Hearn^4^

##### ^*1*^ *UCB, Monheim am Rhein, Germany;* ^*2*^ *UCB, Slough, UK;* ^*3*^ *Customer Faithful Limited, Peterborough, UK;* ^*4*^ *Manchester Metropolitan University, Manchester, UK*

## EPV‐0498

### The ethics of chiropractic cervical spinal manipulation

#### C. Gericke

##### School of Medicine, Australian National University, Canberra, Australia & Canberra Epilepsy Specialist Centre, Deakin Private Hospital, Canberra, Australia

## Headache

## EPV‐0499

### “Interictal photophobia as a potential risk factor for migraine chronification and treatment resistance: A prospective study proposal”

#### M. Alkhawaja; M. Fragoso; J. Paz; P. Comes; G. Salazar

##### Consorci sanitari de Terrassa (CST), Barcelona, Spain

## EPV‐0500

### A Comparison of the efficacy, tolerability and adverse effects of two Anti‐CGRP (Erenumab and Galcanezumab) in migraine prevention

#### M. El‐Lahawi; S. Ibrahim

##### Neurology Department, SKMC, Abu Dhabi, UAE

## EPV‐0501

### Absenteeism caused by migraine among Norwegian hospital nurses

#### K. Alstadhaug ^1^; A. Wisth^1^; R. Jensen^2^; S. Ose^3^

##### ^*1*^ *Department of Neurology/Nordland Hospital, Bodo, Norway;* ^*2*^ *Danish Headache Centre, Rigshospitalet‐Glostrup, University of Copenhagen, Copenhagen, Denmark.;* ^*3*^ *SINTEF, Trondheim, Norway*

## EPV‐0502

### Achieving higher standards for migraine prevention in a series of 639 patients with chronic migraine treated with OnabotulinumtoxinA

#### C. Sánchez‐Rodríguez; E. Varas‐Martín; C. Montero Grande; P. Jiménez Caballero; Á. Sierra‐Mencía; A. Recio‐García; P. Caballero‐Lillo; M. Peñas‐Martínez; A. Gonzalez‐Martinez; Á. Guerrero‐Peral

##### Headache Unit, Neurology Department, Hospital Clínico Universitario de Valladolid, Valladolid, Spain

## EPV‐0503

### Alterations in interoception among patients with medication‐overuse headache

#### G. Ustun^1^; S. Aksu^2^; S. Bek^1^; G. Kutlu ^1^

##### ^*1*^ *Mugla SK UNiversity, School of Medicine, Department of Neurology, Muğla, Turkey;* ^*2*^ *Mugla SK UNiversity, School of Medicine, Department of Physiology, Muğla, Turkey*

## EPV‐0504

### Atogepant for migraine prevention, including patients previously treated with anti‐CGRP therapies: A real‐world cohort

#### R. Nunes Rato; A. Neves; C. Sousa; R. Dias; M. Pinto; M. Pinto; A. Costa

##### Department of Neurology, Unidade Local de Saúde São João, Porto

## EPV‐0505

### Beyond headache freedom: Crystal‐clear and unclear days to assess residual interictal burden in migraine treated with CGRP monoclonal antibodies

#### F. Chianese ^1^; M. Silvestro^1^; I. Orologio^1^; R. Ornello^2^; F. Trojsi^1^; A. Tessitore^1^; S. Sacco^2^; A. Russo^1^

##### ^*1*^ *Headache Centre, Department of Advanced Medical and Surgical Sciences, University of Campania “Luigi Vanvitelli”, Naples, Italy;* ^*2*^ *Department of Biotechnological and Applied Clinical Sciences, University of L' Aquila, L' Aquila, Italy*

## EPV‐0506

### Beyond headache frequency: Sex‐specific clinical and lifestyle signatures of migraine chronicity

#### D. Onan ^1^; E. Uludüz^2^; S. Eyüboğlu^3^; M. İskender^2^; D. Sucu^4^; S. Evin^5^; A. Özge^6^; D. Uludüz^7^

##### ^*1*^ *Department of Physiotherapy and Rehabilitation, Faculty of Health Sciences, Yozgat Bozok University, Yozgat, Türkiye;* ^*2*^ *Koc University Medical Faculty, Grade III Medical Student, Istanbul, Türkiye;* ^*3*^ *Department of Psychology, Brain 360 Holistic Approach Center, Istanbul, Türkiye;* ^*4*^ *Mersin University School of Medicine, Department of Biostatistics and Medical Informatics, Mersin University, Mersin, Türkiye;* ^*5*^ *İslahiye Public Hospital, Gaziantep, Türkiye;* ^*6*^ *Mersin University School of Medicine, Department of Neurology, Algology and Clinical Neurophysiology, Mersin, Türkiye;* ^*7*^ *Department of Neurology, Cerrahpaşa Faculty of Medicine, Istanbul University Cerrahpaşa, Istanbul, Türkiye*

## EPV‐0507

### Beyond pain: impact of atogepant on migraine‐related symptoms

#### C. Sousa; A. Neves; R. Rato; R. Dias; M. Pinto; M. Pinto; A. Costa

##### Department of Neurology, São João University Hospital, Porto, Portugal

## EPV‐0508

### Breath holding cerebrovascular responses in migraine: longitudinal fNIRS comparison of CGRP mAb and oral preventives

#### Y. Kim

##### Department of Neurology, Hallym University Sacred Heart Hospital, Hallym University College of Medicine, Anyang, Republic of Korea

## EPV‐0509

### Cerebrospinal fluid biomarkers in idiopathic intracranial hypertension: Targeted proteomics analysis

#### B. Arora; A. Pandit; S. Shree; P. Tangri; R. Singh; M. Nath

##### Department of Neurology, AIIMS, New Delhi, India

## EPV‐0510

### CGRP level in patients with vestibular migraine and the therapeutic effect of onabotulinumtoxinA

#### B. Holmuratova; N. Rashidova; K. Khalimova

##### Department of Neurology, Tashkent State Medical University, Tashkent, Uzbekistan

## EPV‐0511

### Changes in muscle tone after electrotherapy in patients with chronic migraine and cervicalgia

#### L. Zharashueva ^1^; M. Naprienko^2^; L. Smekalkina^2^

##### ^*1*^ *Department of Nervous Diseases and Neurosurgery, Sechenov University, Moscow, Russian Federation;* ^*2*^ *Department of Sports medicine and medical rehabilitation, Sechenov University, Moscow, Russian Federation*

## EPV‐0512

### Characterisation of patients with new daily persistent headache treated at a tertiary care hospital

#### S. Sánchez Gamino; J. Pérez Ceballos; I. Rodríguez Lavado; L. García Trujillo; M. Castro Sánchez

##### Hospital Regional Universitario de Málaga, Málaga, Spain

## EPV‐0513

### Clinical characteristics and outcomes of patients with idiopathic intracranial hypertension treated by transvenous venous sinus stenting

#### R. Nadig ^1^; S. Deepalam^2^; S. GG^1^; S. Shivde^1^; S. Badachi^1^; A. Huddar^1^; T. Mathew^1^; G. Sarma^1^

##### ^*1*^ *Department of Neurology, St Johns Medical College Hospital Bengaluru, India;* ^*2*^ *Department of Radiology, St Johns Medical College Hospital Bengaluru, India*

## EPV‐0514

### Clinical outcomes of rimegepant in acute and prophylactic migraine therapy in a real‐world setting

#### N. Öksüz ^1^; E. Ekizoğlu^2^; N. Tepe^3^; R. Karacı^4^; T. Cerrahoğlu Şirin^5^; A. Atalar^6^; E. Acıman Demirel^7^; B. Polat^8^; P. Yalınay Dikmen^9^; D. Vurallı^10^; H. Genç^11^; F. Domaç^4^; E. Ilgaz Aydınlar^9^; E. Kocasoy Orhan^2^; A. Özge^1^

##### ^*1*^ *Department of Neurology, Mersin University, School of Medicine, Mersin, Türkiye;* ^*2*^ *Department of Neurology, Istanbul University, Istanbul Faculty of Medicine, Istanbul, Türkiye;* ^*3*^ *Department of Neurology, Balıkesir University, School of Medicine, Balıkesir, Türkiye;* ^*4*^ *Department of Neurology, University of Health Sciences, Erenköy Psychiatry and Neurological Diseases Training and Research Hospital, Istanbul, Türkiye;* ^*5*^ *Department of Neurology, University of Health Sciences, Şişli Hamidiye Etfal Training and Research Hospital, Istanbul, Türkiye;* ^*6*^ *Department of Neurology, Istanbul Physical Therapy and Rehabilitation Training and Research Hospital, Istanbul, Türkiye;* ^*7*^ *School of Medicine, Neurology Department, Zonguldak Bülent Ecevit University, School of Medicine, Zonguldak, Türkiye;* ^*8*^ *Department of Neurology, Düzce University, School of Medicine, Düzce, Türkiye;* ^*9*^ *Department of Neurology, Acıbadem University, School of Medicine, Istanbul, Türkiye;* ^*10*^ *Department of Neurology and Algology, Neuropsychiatry Center, Neuroscience and Neurotechnology Center of Excellence (NÖROM) Gazi University, Faculty of Medicine, Ankara, Türkiye;* ^*11*^ *Department of Neurology, Gaziantep City Hospital, Health Application and Research Center, Gaziantep, Türkiye*

## EPV‐0515

### Clinical outcomes of topiramate and onabotulinumtoxinA in chronic migraine prophylaxis

#### B. Holmuratova ^1^; N. Rashidova^1^; K. Khalimova^1^; Z. Muminova^2^

##### ^*1*^ *Neurology Department, Tashkent State Medical University, Tashkent, Uzbekistan;* ^*2*^ *Department of Obstetrics and Gynecology, Tashkent State Medical University, Tashkent, Uzbekistan*

## EPV‐0516

### Cluster headache and sleep: A questionnaire‐based cross‐sectional study on the bidirectional relationship between attacks and sleep disturbances

#### A. Chotoura ^1^; H. Göbel^2^; K. Heinze‐Kuhn^2^; A. Heinze^2^; C. Göbel^3^

##### ^*1*^ *Faculty of Medicine, Kiel University, Kiel, Germany;* ^*2*^ *Kiel Migraine and Headache Centre, Kiel, Germany;* ^*3*^ *Department of Neurology, University Hospital Schleswig‐Holstein, Campus, Kiel, Germany*

## EPV‐0517

### Cluster‐BIO: Cross‐sectional study of plasma biomarkers (CGRP, PACAP‐38, and VIP) in patients with cluster headache

#### L. Portocarrero‐Sánchez ^1^; F. Laso‐García^2^; N. Díaz‐Gamero^2^; A. Calzado‐González^2^; M. Ruiz‐Castrillo^1^; L. Zaballa‐Pérez^1^; J. Díaz‐de‐Terán^2^

##### ^*1*^ *Department of Neurology, Hospital Universitario La PaZ, Madrid, Spain;* ^*2*^ *Department of Neurology, Hospital Universitario La PaZ, Hospital La Paz Institute for Health Research – IdiPAZ (La Paz University Hospital – Universidad Autónoma de Madrid), Madrid, Spain*

## EPV‐0518

### COACT study: CGRPmAbs + OnabotulinumtoxinA assessment of chronic migraine treatments study

#### B. Torphy; M. Smith; S. Lamba; S. Koseli; A. Rakhangi; S. Dairo

##### Chicago Headache Center and Research Institute, Chicago, USA

## EPV‐0519

### Cognitive complaints in migraine: response to preventive treatment with atogepant

#### M. Costa Taveira; F. Dourado Sotero; M. Dias da Costa; R. Rodrigues; I. Pavão Martins

##### Serviço de Neurologia, Departamento de Neurociências e Saúde Mental, ULS Santa Maria, Lisbon, Portugal

## EPV‐0520

### Cognitive flexibility, resilience and executive control in chronic migraine with and without medication overuse

#### Y. Şengül^1^; E. Gülhan ^2^; F. Ülker^1^

##### ^*1*^ *Department of Neurology, Faculty of Medicine, Çanakkale Onsekiz Mart University, Çanakkale, Türkiye;* ^*2*^ *Faculty of Medicine, Çanakkale Onsekiz Mart University, Çanakkale, Türkiye*

## EPV‐0521

### Comparative efficacy and safety of magnesium sulfate versus metoclopramide for treating acute migraines: A systematic review and meta‐analysis

#### S. Wasti^1^; A. Wasti^2^; M. Abdullah ^1^; Y. Mir^1^

##### ^*1*^ *Department of Neurology, King Edward Medical University, Lahore, Pakistan;* ^*2*^ *Department of Neurology, Continental Medical College, Lahore, Pakistan*

## EPV‐0522

### Comparative efficacy and safety of metoclopramide versus sumatriptan in acute management of migraine: A systematic review and meta‐analysis

#### S. Wasti^1^; A. Wasti^2^; M. Shahid ^1^

##### ^*1*^ *Department of Neurology, King Edward Medical University, Lahore, Pakistan;* ^*2*^ *Department of Neurology, Continental Medical College, Lahore, Pakistan*

## EPV‐0523

### Comparative efficacy and safety of propranolol versus flunarizine for migraine prophylaxis: A systematic review and meta‐analysis

#### S. Wasti^1^; A. Wasti^2^; S. Waqas ^1^

##### ^*1*^ *Department of Neurology, King Edward Medical University, Lahore, Pakistan;* ^*2*^ *Department of Neurology, Continental Medical College, Lahore, Pakistan*

## EPV‐0524

### Comparing neurologist and neurologist‐customised semantic search engine management plans for chronic migraine: A prospective pilot study from Kenya

#### A. Vakil ^1^; J. Shah^2^; H. Ekea^1^; D. Sokhi^2^

##### ^*1*^ *Department of Medicine, Aga Khan University Medical College of East Africa (Nairobi Campus);* ^*2*^ *Brain and Mind Institute, Aga Khan University, Kenya*

## EPV‐0525

### Data of efficacy and safety of Atogepant in the preventive treatment of migraine in a cohort of highly treatment‐refractory migraine patients

#### M. Morra; R. Ferri; E. Virgilio; F. Masuzzo; F. De Mattei; M. Matta; M. Torrieri; M. Clerico

##### SSD Specialist Neurological Pathologies, University Hospital San Luigi Gonzaga, Orbassano, Italy

## EPV‐0526

### Diagnostic yield of non‐contrast cranial CT in chronic tension‐type headache: A retrospective cohort

#### M. Acharya ^1^; A. Debnath^2^; S. Rana^1^

##### ^*1*^ *Dr. B C Roy Multispecialty Medical Research Centre, IIT Kharagpur, Kharagpur, India;* ^*2*^ *Department of Neuromedicine, Bankura Sammilani Medical College, Bankura, India*

## EPV‐0527

### Distinct clinical features in adult and pediatric HaNDL syndrome: A literature‐based analysis

#### G. Cabral ^1^; M. Brás Monteiro^1^; M. Viana Baptista^1^

##### ^*1*^ *Neurology Department, Hospital de Egas Moniz, Unidade Local Saúde Lisboa Ocidente, Portugal;* ^*2*^ *Chronic Diseases Research Center (CEDOC), NOVA Medical School, Universidade NOVA de Lisboa, Lisbon, Portugal*

## EPV‐0528

### Effectiveness and safety in patients receiving atogepant

#### P. López‐Grueiro Valcarce; M. Ruiz Castrillo; F. Díaz de Terán Velasco; L. Portocarrero Sánchez

##### Neurology, Hospital Universitario La Paz, Madrid, Spain

## EPV‐0529

### Effectiveness of pulsed radiofrequency for migraine: A systematic review and meta‐analysis

#### H. Hussien; A. Hegazi; M. SeragEldin

##### Faculty of Medicine Mansoura University, Mansoura, Egypt

## EPV‐0530

### Effects of astrocytic metabolic dysfunction and glucose stress on gene expression during cortical spreading depression

#### S. Khan

##### *Wayne State University*, *Detroit; USA*

## EPV‐0531

### Efficacy and safety of Eptinezumab 100 mg and 300 mg for migraine prevention: A systematic review and meta‐analysis of randomized trials

#### M. Koirala; A. Pandit

##### Department of Internal Medicine, Chitwan Medical College Teaching Hospital, Bharatpur, Nepal

## EPV‐0532

### Efficacy and safety of fentanyl for prophylaxis against postdural puncture headache in cesarean section: A meta‐analysis

#### S. Wasti^1^; A. Wasti^2^; M. Shahid ^1^

##### ^*1*^ *Department of Neurology, King Edward Medical University, Lahore, Pakistan;* ^*2*^ *Department of Neurology, Continental Medical College, Lahore, Pakistan*

## EPV‐0533

### Efficacy and safety of Gabapentin versus placebo against postdural puncture headache: A systematic review and meta‐analysis

#### S. Wasti^1^; A. Wasti^2^; F. Amin ^1^

##### ^*1*^ *Department of Neurology, King Edward Medical University, Lahore, Pakistan;* ^*2*^ *Department of Neurology, Continental Medical College, Lahore, Pakistan*

## EPV‐0534

### Efficacy and safety of magnesium in preventing post‐dural puncture headache after cesarean delivery: A systematic review

#### S. Wasti^1^; A. Wasti^2^; S. Waqas ^1^

##### ^*1*^ *Department of Neurology, King Edward Medical University, Lahore, Pakistan;* ^*2*^ *Department of Neurology, Continental Medical College, Lahore, Pakistan*

## EPV‐0535

### Efficacy and safety of ondansetron versus conservative treatment in post‐dural puncture headache prophylaxis in cesarean section: A meta‐analysis

#### S. Wasti^1^; A. Wasti^2^; F. Amin ^1^

##### ^*1*^ *Department of Neurology, King Edward Medical University, Lahore, Pakistan;* ^*2*^ *Department of Neurology, Continental Medical College, Lahore, Pakistan*

## EPV‐0536

### Efficacy and safety of Rimegepant 75 mg for migraine prevention: A meta‐analysis of randomized controlled trials

#### A. Pandit; M. Koirala

##### Department of Internal Medicine, Chitwan Medical College Teaching Hospital, Bharatpur, Nepal

## EPV‐0537

### Efficacy and safety of valproate versus topiramate for pediatric migraine prophylaxis: A systematic review

#### S. Wasti^1^; A. Wasti^2^; M. Berkinbayeva^3^; S. Cano‐Reyes^4^; V. Kuo^5^; M. Iqbal^1^; M. Saddique ^1^

##### ^*1*^ *Department of Neurology, King Edward Medical University, Lahore, Pakistan;* ^*2*^ *Department of Neurology, Continental Medical College, Lahore, Pakistan;* ^*3*^ *Department of Neurology, Asfendiyarov Kazakh National Medical University, Almaty, Kazakhstan;* ^*4*^ *Department of Neurology, Hospital de la Misericordia/Universidad Nacional de Colombia. Bogotá D.C., Colombia;* ^*5*^ *Department of Neurology, National Taiwan University, Taipei City, Taiwan*

## EPV‐0538

### Efficacy of coenzyme Q10 for migraine: a systematic review, meta‐analysis, and trial sequential analysis

#### K. Alidreesi ^1^; G. Alsamiri^1^; M. Bukari^1^; M. Almutairy^1^; R. Alharthi^1^; R. Abuhadi^1^; M. Halabi^1^; A. Attar^2^; A. Alsabban^3^

##### ^*1*^ *College of Medicine, King Saud bin Abdulaziz University for Health Sciences, Jeddah, Kingdom of Saudi Arabia;* ^*2*^ *Neurology Department, King Abdulaziz Medical City, Jeddah, Kingdom of Saudi Arabia;* ^*3*^ *Family Medicine Department, King Abdulaziz Medical City, Jeddah, Kingdom of Saudi Arabia*

## EPV‐0539

### Efficacy of Rimegepant 75 mg for acute migraine treatment: A systematic review and meta‐analysis of randomized controlled trials

#### A. Pandit; M. Koirala

##### Department of Internal Medicine, Chitwan Medical College Teaching Hospital, Bharatpur, Nepal

## EPV‐0540

### Eptinezumab for difficult to treat migraine: Who responds and who need more

#### A. Visentini; E. Ratto; F. Genovese; B. Colombo; R. Messina; M. Filippi

##### Neurology Unit IRCCS San Raffaele Scientific Institute, Milan, Italy

## EPV‐0541

### Frequency of migraine phenotype in individuals at risk for MEFV variations: A neuroinflammatory reflection of familial mediterranean fever

#### Y. Guldiken^1^; M. Attaalla ^2^; A. Bakan^3^; F. Silan^4^

##### ^*1*^ *Department of Neurology, Faculty of Medicine, Canakkale Onsekiz Mart University, Canakkale, Türkiye;* ^*2*^ *Faculty of Medicine, Canakkale Onsekiz Mart University, Canakkale, Türkiye;* ^*3*^ *Faculty of Medicine, Canakkale Onsekiz Mart University, Canakkale, Türkiye;* ^*4*^ *Department of Medical Genetics, Faculty of Medicine, Canakkale Onsekiz Mart University, Canakkale, Türkiye*

## EPV‐0542

### Global prevalence of medication‐overuse headache: A systematic review and meta‐analysis

#### S. Wasti^1^; S. Ali ^1^; A. Wasti^2^

##### ^*1*^ *Department of Neurology, King Edward Medical University, Lahore, Pakistan;* ^*2*^ *Department of Neurology, Continental Medical College, Lahore, Pakistan*

## EPV‐0543

### Headache features among military personnel hospitalized following combat deployment

#### M. Bozhenko; V. Ivanyk; U. Fomenko; N. Bozhenko

##### Neurology Department, Danylo Halytsky Lviv National Medical University, Lviv, Ukraine

## EPV‐0544

### Headache in chiari malformation type I: Clinical phenotypes and diagnostic pitfalls

#### N. Najafbayli

##### Azerbaijan Medical University, Baku, Azerbaijan

## EPV‐0545

### High standards of migraine control in patients treated with anti‐CGRP monoclonal antibodies

#### A. Martins ^1^; E. Pinto^2^; M. Baptista^3^; G. Cabral^4^

##### ^*1*^ *Neurology Department, Hospital de Egas Moniz, Lisboa, Portugal;* ^*2*^ *Health superior School, Algarve University, Faro, Portugal;* ^*3*^ *Lisbon Clinical Academic Center, NOVA Medical School, Universidade NOVA, Lisboa;* ^*4*^ *Lisbon Clinical Academic Center, NOVA Medical School, Universidade NOVA, Lisboa*

## EPV‐0546

### How does war affect the next generation of doctors? Cognitive flexibility and headache burden among sudanese medical students

#### S. Mokhtar ^1^; M. Abdulrahman^2^; M. Ahmed^1^; Y. Fadlelmoula^3^; M. Gasmalha^4^

##### ^*1*^ *University of Gadarif, Gadarif, Sudan;* ^*2*^ *Alzaiem Alazhari University, Khartoum, Sudan;* ^*3*^ *Gazira University, Wad Madani, Sudan;* ^*4*^ *University of Omdurman Islamic, Omdurman, Sudan*

## EPV‐0547

### Immunogenetic drivers of migraine: Evidence for microglial inflammasome and IL‐1β involvement

#### B. Guerra Leal ^1^; A. Teixeira^2^; C. Carvalho^2^; S. Brás^2^; M. Alves Ferreira^3^; A. Dias^4^; C. Lemos^5^

##### ^*1*^ *Unit for Multidisciplinary Research in Biomedicine; Immunogenetics Laboratory, School of Medicine and Biomedical Sciences, University of Porto; Laboratory for Integrative and Translational Research in Population Health (ITR), Porto, Portugal;* ^*2*^ *Immunogenetics Laboratory, Department of Molecular Pathology and Immunology, School of Medicine and Biomedical Sciences, University of Porto, Porto, Portugal;* ^*3*^ *Unit for Multidisciplinary Research in Biomedicine; School of Medicine and Biomedical Sciences, University of Porto; i3S – Instituto de Investigação e Inovação em Saúde, Universidade do Porto; CGPP‐IBMC – Center for Predictive and Preventive Genetics Univ;* ^*4*^ *Unit for Multidisciplinary Research in Biomedicine; School of Medicine and Biomedical Sciences, University of Porto; i3S – Institute for Research and Innovation in Health, University of Porto, Porto Portugal;* ^*5*^ *Unit for Multidisciplinary Research in Biomedicine; Laboratory for Integrative and Translational Research in Population Health (ITR), Porto, Portugal*

## EPV‐0548

### Impact of ATOgepant on interictal burden and crystal CLEAR days in difficult‐to‐treat migraine: The ATO‐CLEAR study

#### L. Zaballa Pérez ^1^; L. Portocarrero Sánchez^1^; M. Gómez Fábrega^1^; M. Ruiz Castrillo^1^; J. Díaz de Terán^1,2,3^

##### ^*1*^ *La Paz University Hospital, Madrid, Spain;* ^*2*^ *La Paz University Hospital, Madrid, Spain;* ^*3*^ *Hospital La Paz Institute for Health Research, Autónoma University of Madrid, Madrid, Spain*

## EPV‐0549

### Impact of premonitory symptoms on quality of life in patients with migraine without aura

#### D. Spajic^1^; F. Bukulin^1^; Z. Popovic ^2,3^; D. Janculjak^2,3^

##### ^*1*^ *Faculty of Medicine Osijek, Josip Juraj Strossmayer University of Osijek, Osijek, Croatia;* ^*2*^ *Faculty of Medicine Osijek, Josip Juraj Strossmayer University of Osijek, Osijek, Croatia;* ^*3*^ *Department of Neurology, University Hospital Center Osijek, Osijek, Croatia*

## EPV‐0550

### Impact of sex on clinical presentation and diagnostic delay in cluster headache

#### L. Portocarrero‐Sánchez ^1^; L. Zaballa‐Pérez^1^; J. Díaz‐de‐Terán^2.3^

##### ^*1*^ *Neurology, Hospital Universitario La Paz, Madrid, Spain;* ^*2*^ *Neurology, Hospital Universitario La Paz, Madrid, Spain;* ^*3*^ *Hospital La Paz Institute for Health Research – IdiPAZ (La Paz University Hospital – Universidad Autónoma de Madrid) Madrid, Spain*

## EPV‐0551

### Implementation and impact of medication reviews on patients eligible for eptinezumab treatment in severe migraine in a pain clinic

#### V. Arcani ^1^; V. Giraudi^1^; A. Bruyat^2^; A. Donnet^3^; G. Hache^1^

##### ^*1*^ *Aix‐Marseille Université, AP‐HM, Hôpital de La Timone, Service Pharmacie, Marseille, France;* ^*2*^ *Aix Marseille Université, Marseille, France;* ^*3*^ *Aix‐Marseille Université, AP‐HM, Hôpital de La Timone, Centre d'Evaluation et de Traitement de la Douleur, Marseille, France*

## EPV‐0552

### Interpretation of increased resistive index in the middle meningeal artery during migraine attacks

#### Y. Güldiken^1^; E. Gülhan ^2^

##### ^*1*^ *Department of Neurology, Faculty of Medicine, Çanakkale Onsekiz Mart University, Çanakkale, Türkiye;* ^*2*^ *Faculty of Medicine, Çanakkale Onsekiz Mart University, Çanakkale, Türkiye*

## EPV‐0553

### Intracranial hypertension due to a giant Pacchionian granulation: A case report with complete endovascular resolution

#### E. Lucas Oliver; P. Cabezudo García; P. Serrano Castro

##### Neurología, Hospital Regional Universitario De Málaga, Málaga, Spain

## EPV‐0554

### Less is more: Low‐dose divalproex for recurrent dialysis headache

#### L. Tablieh; M. Yousaf; M. Hussain; F. Nauman

##### *Virginia Tech Carilion School of Medicine*, *Roanoke, USA*

## EPV‐0555

### Machine learning for differential diagnosis of migraine: A systematic review

#### S. Wasti^1^; F. Amin^1^; A. Wasti^2^; S. Chattha ^1^

##### ^*1*^ *Department of Neurology, King Edward Medical University, Lahore, Pakistan;* ^*2*^ *Department of Neurology, Continental Medical College, Lahore, Pakistan*

## EPV‐0556

### Machine learning in headache: A systematic review of diagnostic and classification approaches

#### S. Wasti^1^; F. Amin ^1^; A. Wasti^2^

##### ^*1*^ *Department of Neurology, King Edward Medical University, Lahore, Pakistan;* ^*2*^ *Department of Neurology, Continental Medical College, Lahore, Pakistan*

## EPV‐0557

### Medication misuse of CGRP‐targeted therapies without adverse effects in chronic migraine

#### R. Rodrigues ^1^; M. Taveira^1^; F. Sotero^1^; I. Martins^2^

##### ^*1*^ *Neurology, Department of Neurosciences and Mental Health, Unidade Local de Saúde de Santa Maria, Lisbon, Portugal;* ^*2*^ *Centro de Estudos Egas Moniz, Clínica Universitária de Neurologia, Faculty of Medicine, University of Lisbon, Lisbon, Portugal*

## EPV‐0558

### Migraine and pregnancy: Can we do better?

#### Á. Antón Conejos ^1^; D. Arcila Salazar^1^; E. Garcés Becerril^1^; D. Ginarte Milanés^1^; L. Pérez Jiménez^2^; J. Cajape Mosquera^1^; S. Santos Lasaosa^1^

##### ^*1*^ *Neurology, Hospital Clínico Universitario Lozano Blesa, Zaragoza, Spain;* ^*2*^ *Instituto de Investigación Sanitaria Aragón, Zaragoza, Spain*

## EPV‐0559

### Migraine diagnosis across Europe: EMHA migraine in women survey results, H2 2025

#### P. Pozo‐Rosich ^1^; E. Ruiz de la Torre^2^; P. Goadsby^3^; D. Watson^4^; E. Couturier^5^; A. MacGregor^6^; R. Nappi^7^; E. Merki^8^; D. Hurtado^9^; C. Fairley^10^

##### ^*1*^ *Headache Clinic, Neurology Department, Vall d’Hebron University Hospital, Barcelona, Spain;* ^*2*^ *European Migraine & Headache Alliance (EMHA);* ^*3*^ *Division of Biomedical Sciences, King Abdullah University of Science and Technology, Saudi Arabia; and King's College London, London, UK;* ^*4*^ *GPwER headache, Department of Neurology, Aberdeen Royal Infirmary, Aberdeen, Scotland;* ^*5*^ *MD, Consultant Neurologist, Head of Department of Neurology, Neurologie Centrum Amsterdam, Amsterdam, The Netherlands;* ^*6*^ *Centre for Neuroscience, Surgery and Trauma, Barts and the London School of Medicine and Dentistry, London, UK; and Centre for Reproductive Medicine, St Bartholomew's Hospital, London, UK;* ^*7*^ *Department of Clinical, Surgical, Diagnostic and Pediatric Sciences, University of Pavia, Pavia, Italy; and Research Center for Reproductive Medicine, Gynecological Endocrinology and Menopause, IRCCS S. Matteo Foundation, Pavia, Italy;* ^*8*^ *University of Zürich, Zürich, Switzerland;* ^*9*^ *Prescient Healthcare Group;* ^*10*^ *Prescient Healthcare Group*

## EPV‐0560

### Migraine in low‐income community: Prevalence, care practice and access barriers – a cross‐sectional study

#### M. Sedano ^1^; C. Macalintal^2^

##### ^*1*^ *Department of Neurosciences, Asian Hospital Medical Center, India;* ^*2*^ *Department of Neurosciences, Asian Hospital Medical Center, India*

## EPV‐0561

### Migraine with aura in female patients with psoriasis: A systematic review

#### D. Moreira do Amaral ^1^; L. Fernandes^2^

##### ^*1*^ *São Francisco University, Bragança Paulista, Brazil;* ^*2*^ *São Francisco University, Bragança Paulista, Brazil*

## EPV‐0562

### Migrainous infarction masked by Patent Foramen Ovale: Diagnostic challenge and preventive management

#### C. Hurtado Alcázar; J. Ruiz del Amo; C. Santillana Ávila; P. Guirado Ruiz; I. Villegas Rodríguez

##### Department of Neurology, San Cecilio Universitary Hospital, Granada, Spain

## EPV‐0563

### OnabotulinumtoxinA for occipital neuralgia: results from a series of 15 patients

#### C. Montero‐Grande; P. Jiménez Caballero; C. Sánchez‐Rodríguez; E. Varas‐Martín; Á. Sierra‐Mencía; A. Recio‐García; M. Peñas‐Martínez; Y. González‐Osorio; R. Areses‐Calderón; Á. Guerrero Peral

##### Neurology, Hospital Clínico Universitario de Valladolid, Valladolid, España

## EPV‐0564

### OnabotulinumtoxinA in patients with painful trigeminal neuropathy. Experience in a headache unit

#### P. Jiménez‐Caballero; C. Montero‐Grande; C. Sánchez‐Rodríguez; E. Varas‐Martín; Á. Sierra‐Mencía; A. Recio‐García; M. Peñas‐Martínez; Y. González‐Osorio; R. Areses‐Calderón; Á. Guerrero‐Peral

##### Headache Unit, Neurology Department, Hospital Clínico Universitario, Valladolid, Spain

## EPV‐0565

### Outcomes of blind epidural blood patching in intracranial hypotension

#### H. Qazi; G. Kennedy; S. Cope

##### Sunderland Royal Hospital, Sunderland, UK

## EPV‐0566

### Peripheral blood electrolyte profiles in patients with migraine with and without aura

#### S. Jorquera‐Ortega ^1^; M. Almeida^1^; S. Sánchez^2^; M. Conversa^3^; A. Barbosa^3^; Y. González Osorio^4^; Á. Sierra^4^; Á. Guerrero‐Peral^4^; A. Gonzalez‐Martinez^1^

##### ^*1*^ *Faculty of Medicine*, *Autonomous University of Madrid*, *Madrid, Spain; Hospital Universitario de la Princesa, Madrid, Spain; Instituto de Investigación Sanitaria Princesa (IIS‐Princesa), Madrid, Spain;* ^*2*^ *Laboratory Service, Hospital Universitario de la Princesa, Madrid, Spain;* ^*3*^ *Neuroradiology Service, Hospital Universitario de la Princesa, Madrid, Spain;* ^*4*^ *Headache Unit, Neurology Service, Hospital Clínico Universitario de Valladolid, Valladolid, Spain*

## EPV‐0567

### Prevalence and characteristics of headaches in adolescents – is problem being underestimated? Subgroups analysis of the HY‐LIFE study

#### K. Różycka ^1^; N. Siwak^1^; J. Bielewicz^2^; K. Rejdak^2^

##### ^*1*^ *Student Science Club at the Chair and Department of Neurology, Medical University of Lublin, Lublin, Poland;* ^*2*^ *Department of Neurology, Medical University of Lublin, Lublin, Poland*

## EPV‐0568

### Prevalence and clinical features of cough headache: A systematic review with meta‐analysis

#### A. Osiowski; M. Osiowski; D. Taterra

##### Jagiellonian University Medical College, Kraków, Poland

## EPV‐0569

### Prophylactic efficacy and safety of sphenopalatine ganglion block versus greater occipital nerve block against headache disorders: A meta‐analysis

#### S. Wasti^1^; A. Wasti^2^; S. Shahid ^1^

##### ^*1*^ *Department of Neurology, King Edward Medical University, Lahore, Pakistan;* ^*2*^ *Department of Neurology, Continental Medical College, Lahore, Pakistan*

## EPV‐0570

### Real‐world effects of onabotulinumtoxinA and anti‐CGRP monoclonal antibodies on disability and clinical phenotype in chronic migraine

#### B. Adamczyk; K. Wierzbicki; M. Baczkowska; W. Wiącek; K. Trąbka; R. Wąsek; M. Adamczyk‐Sowa

##### Department of Neurology, Faculty of Medical Sciences in Zabrze, Medical University of Silesia in Katowice, Zabrze, Poland

## EPV‐0571

### Real‐world efficacy and tolerability of atogepant for migraine prevention after 6 months of treatment: a double center experience

#### M. Miccoli ^1^; L. La Volpe^1^; A. Galli^3^; M. Secchi^2^; G. Querzola^3^; R. Altavilla^3^; A. Priori^1^; C. Manfredi^2^

##### ^*1*^ *Department of Health Sciences, Aldo Ravelli Center for Neurotechnology and Experimental Brain Therapeutics, University of Milan, Milan, Italy;* ^*2*^ *Neurology Unit, ASST Santi Paolo e Carlo, San Paolo University Hospital, Milan, Italy;* ^*3*^ *Neurology Unit, ASST Santi Paolo e Carlo, San Carlo Hospital Milan, Italy*

## EPV‐0572

### Real‐world long‐term effectiveness and retention of CGRP‐targeted therapies: A 4‐year retrospective study in a Thai tertiary headache clinic

#### C. Chansakul ^1^; C. Thearapati^2^; N. Jindawong^1^; K. Vongvaivanich^1^

##### ^*1*^ *Neuroscience Center, Bangkok International Hospital, Bangkok, Thailand;* ^*2*^ *Faculty of Medicine, Bangkok Thonburi University, Bangkok, Thailand*

## EPV‐0573

### Remote electrical neuromodulation (Ren) with nerivio device. Our experience in 4 cases of new daily persistent headache (Ndph)

#### J. Tutusaus; N. Bennouna; B. Jové; M. Ruiz; D. Asín; V. Obach‐Irimia; R. Armand; A. Grajea; M. Marco; A. Sosa; T. Topczewski; S. Fernández; N. Fabregat; V. Obach

##### Neurology Department, Hospital Clínic de Barcelona, Spain

## EPV‐0574

### Retrospective evaluation of patients with idiopathic intracranial hypertension: A single‐center experience

#### Ç. Şişman ^1^; B. Eren^1^; C. Erkol^2^; Ö. Çoşkun^3^; D. Aksoy^4^; U. Emre Toprak^1^

##### ^*1*^ *Istanbul Research and Education Hospital, Department of Neurology, Istanbul, Turkey;* ^*2*^ *St Lakere;* ^*3*^ *University of Health Sciences, Department of Anatomy, Istanbul, Turkey;* ^*4*^ *Istanbul Research and Education Hospital, Department of Radiology, Istanbul, Turkey*

## EPV‐0575

### rTMS in treating patients with chronic migraine and depression: A real‐life exploratory analysis

#### D. Quartana ^1^; F. Sperli^1^; F. Celotto^1^; C. Favarato^1^; G. Vesco^1^; R. Ferri^2^; M. Clerico^2^; A. Di Sapio^1^

##### ^*1*^ *CRESM SCDO Neurology‐University Hospital S. Luigi Gonzaga, Orbassano, Turin, Italy;* ^*2*^ *SSD Patologie Neurologiche Specialistiche and Univerisy of Turin‐University Hospital S. Luigi Gonzaga, Orbassano, Turin, Italy*

## EPV‐0576

### Serum and CSF neurofilament light chain and glial fibrillary acidic protein levels in idiopathic intracranial hypertension

#### N. Krajnc ^1^; M. Ponleitner^1^; J. Nolte^1^; M. Bertich^2^; N. Müller^1^; C. Stapf^2^; S. Zaic^1^; S. Macher^1^; W. Marik^3^; K. Novak^4^; C. Wöber^1^; B. Pemp^2^; G. Bsteh^1^

##### ^*1*^ *Department of Neurology, Medical University of Vienna, Vienna, Austria;* ^*2*^ *Department of Ophthalmology, Medical University of Vienna, Vienna, Austria;* ^*3*^ *Department of Neuroradiology, Medical University of Vienna, Vienna, Austria;* ^*4*^ *Department of Neurosurgery, Medical University of Vienna, Vienna, Austria*

## EPV‐0577

### Sex differences in headache unit referral in patients with chronic migraine

#### E. Varas‐Martín; I. Ros‐González; Á. Sierra‐Mencía; A. Recio‐García; P. Caballero‐Lillo; Y. González‐Osorio; C. Sánchez Rodríguez; M. Peñas‐Martínez; Á. Guerrero‐Peral; A. González‐Martínez

##### Headache Unit, Neurology Department, University Clinical Hospital, Valladolid, Spain

## EPV‐0578

### Short‐term effectiveness of new anti‐CGRP therapies in chronic and episodic migraine

#### M. Ruiz Perelló; F. Salazar Hernandez; B. Gómez González; D. Lopez Segura; A. Maija Savolainen; M. Lorente; A. Sancho; D. Vidal Mena; I. Díaz; M. Ortega Ortega; J. Garcia Carmona

##### 1‐Department of Neurology, Santa Lucía University Hospital, Cartagena, Spain

## EPV‐0579

### Sleep disorders in medication‐overuse headache: prevalence, associated factors, and reversibility after treatment

#### E. Lebedeva; I. Kniazeva

##### The Ural State Medical University, International Medical Center “Europe‐Asia”, Yekaterinburg, Russian Federation

## EPV‐0580

### Spontaneous Intracranial Hypotension (SIH): heterogeneity in clinical, radiological, and therapeutic responses—A case series

#### C. Santos Martín ^1^; L. Quirós Illán^1^; E. Martínez Acebes^1^; B. Givica Pérez^2^; L. Llorente Ayuso^1^

##### ^*1*^ *Department of Neurology, Hospital Universitario Infanta Leonor, Madrid, Spain;* ^*2*^ *Department of Radiology, Hospital Universitario Infanta Leonor, Madrid, Spain*

## EPV‐0581

### Spontaneous intracranial hypotension mimicking status migrainosus in a woman with a known history of classic migraine

#### A. Tsimpiktsioglou; S. Stasinou; M. Ioakeimidis; C. Deligianni; T. Doskas

##### Neurology Department, Athens Naval Hospital, Athens, Greece

## EPV‐0582

### Abstract withdrawn

## EPV‐0583

### Study of blood CGRP level patterns in migraine subtypes and other headaches in 1 tertiary healthcare setup of north India

#### A. Sastry

##### Neurology, GEMS Medical College Srikakulam Andhra Pradesh India, India

## EPV‐0584

### Targeted gut‐focused dietary intervention in migraine patients with gastrointestinal comorbidity: A real‐world observational study

#### S. Szatmari ^1^; V. Hadnagy^2^; O. Ban^2^; R. Schwab^3^; L. Mechtler^4^

##### ^*1*^ *Semmelweis University Department of Neurology, Budapest, Hungary;* ^*2*^ *Mind Brain‐Gut Center, Budapest, Hungary;* ^*3*^ *Biomteam, Budapest, Hungary;* ^*4*^ *Dent Neurologic Institute, Roswell Park, Comprehensive Cancer Center, State University of New York at Buffalo, New York, USA*

## EPV‐0585

### Targeted intermittent atogepant for breakthrough attacks in partial responders to fremanezumab: A real‐world case series

#### K. Park

##### Wiltse Memorial Hospital, Suwon, Republic of Korea

## EPV‐0586

### Tension‐type headache and new postural headache: Clues to low cerebrospinal fluid pressure

#### A. Morgadinho; J. Ferreira; T. Geraldes; L. Pereira

##### Neurology Department, Unidade Local de Saúde de Almada‐Seixal, Almada, Portugal

## EPV‐0587

### The impact of Erenumab and Galcanezumab upon quality of life among migraine patients in Kuwait

#### T. Alotaibi; M. Alajmi; M. Alkandari; J. Al‐Hashel; S. Farouk

##### Department of Neurology, Ibn Sina Hospital, Safat, Kuwait City, Kuwait, Department of Medicine, Faculty of Medicine, Health Sciences Centre, Kuwait University, Kuwait City, Kuwait

## EPV‐0588

### The Nordic Chronic Migraine Trial of CGRP monoclonal antibody and onabotulinumtoxin A dual therapy—A randomized placebo‐controlled double‐blind trial

#### B. Bezgal ^1^; K. Wesnes^2^; K. Müller^3^; Z. Gadan^4^; H. Øyen Flemmen^5^; A. Poole^6^; M. Aalstad‐Johansen^7^; M. Bjørk^8^; K. Jocobsen^9^; A. Roy^10^; A. Vesterås^11^; C. Sundal^12^; K. Devik^13^; A. Dueland^14^; L. Hofsøy Steffensen^15^; S. Mathisen^16^; Å. Hagen Morsund^17^; L. Stovner^18^; M. Matharu^19^; M. Toft^20^; H. Winther Schytz^21^; M. Linde^22^; T. Winsløff^23^; D. Dodick^24^; E. Tronvik^25^; A. Aamodt^26^

##### ^*1*^ *Department of Neurology, Oslo University Hospital, Oslo, Norway;* ^*2*^ *Norwegian Centre for Headache Research (NorHead), The Norwegian University of Science and Technology, Trondheim, Norway;* ^*3*^ *Department of Neurology, Sørlandet Hospital Trust, Kristiansand, Norway;* ^*4*^ *Department of Neurology, Østfold Hospital Trust, Sarpsborg, Norway;* ^*5*^ *Department of Neurology, Telemark Hospital Trust, Skien, Norway;* ^*6*^ *Oslo Headache Centre, Oslo, Norway;* ^*7*^ *Department of Neurology, Innlandet Hospital Trust, Lillehammer, Norway;* ^*8*^ *Department of Neurology, Haukeland University Hospital, Bergen, Norway;* ^*9*^ *Department of Neurology, Oslo University Hospital, Oslo, Norway;* ^*10*^ *Department of Neurology, Oslo University Hospital, Oslo, Norway;* ^*11*^ *Department of Neurology, Oslo University Hospital, Oslo, Norway;* ^*12*^ *NeuroClinic Norway, Lillestrøm, Norway;* ^*13*^ *Department of Neurology Namsos Hospital, Namsos, Norway;* ^*14*^ *Sandvika Neuro center, Sandvika, Norway;* ^*15*^ *Department of Neurology, University Hospital of North Norway, Tromsø, Norway;* ^*16*^ *Department of Neurology, Stavanger University Hospital, Stavanger, Norway;* ^*17*^ *Department of Neurology, Molde Hospital, Molde, Norway;* ^*18*^ *Norwegian Centre for Headache Research (NorHead), The Norwegian University of Science and Technology, Trondheim, Norway;* ^*19*^ *National Hospital for Neurology and Neurosurgery, University College London Hospital, London, UK;* ^*20*^ *Department of Neurology, Oslo University Hospital, Oslo, Norway;* ^*21*^ *Danish Headache Center, Department of Neurology, Rigshospitalet Glostrup, Copenhagen, Denmark;* ^*22*^ *Department of Neurology, Sahlgrenska University Hospital, Gothenburg, Sweden;* ^*23*^ *Institute of Clinical Medicine, Faculty of Medicine, University of Oslo, Oslo Norway;* ^*24*^ *Mayo Clinic International, Arizona, USA;* ^*25*^ *Norwegian Centre for Headache Research (NorHead), The Norwegian University of Science and Technology, Trondheim, Norway;* ^*26*^ *Department of Neurology, Oslo University Hospital, Oslo, Norway*

## EPV‐0589

### The relationship between computer vision syndrome, technostress, and headache: A multicenter cross‐sectional study

#### F. Eren ^1^; A. Atılgan^1^; G. Eren^2^; O. Ildiz^3^; A. Yildogan^4^; G. Ongun^5^; S. Tenekeci^6^; A. Demir^3^; S. Guzel^7^

##### ^*1*^ *Selcuk University Medical Faculty, Department of Neurology, Konya, Türkiye;* ^*2*^ *Selcuklu District Health Directorate, Konya, Türkiye;* ^*3*^ *Konya City Hospital, Department of Neurology, Konya, Türkiye;* ^*4*^ *Omer Halis Demir University, Clinic of Neurology, Nigde, Türkiye;* ^*5*^ *Istinye University Gaziosmanpasa Medical Park Hospital, Clinic of Neurology, Istanbul, Türkiye;* ^*6*^ *Aksaray Education and Research Hospital, Clinic of Neurology, Aksaray, Türkiye;* ^*7*^ *Selcuk University Faculty of Health Sciences, Department of Health Management, Konya, Türkiye*

## EPV‐0590

### Three decades of tension‐type Headache burden in Europe: Regional variation in age‐standardized disability‐adjusted life years

#### S. Wasti^1^; A. Wasti^2^; M. Javed ^1^

##### ^*1*^ *Department of Neurology, King Edward Medical University, Lahore, Pakistan;* ^*2*^ *Department of Neurology, Continental Medical College, Lahore, Pakistan*

## EPV‐0591

### Tryptophan used as a dietary supplement: A new option for migraine treatment?

#### G. Nagy‐Grócz ^1^; T. Körtési^1^; L. Ajkay‐Donáth^2^; E. Vágvölgyi‐Sümegi^1^; Z. Galla^3^; J. Tajti^4^; L. Vécsie^5^

##### ^*1*^ *Department of Theoretical Health Sciences and Health Management, Faculty of Health Sciences and Social Studies, University of Szeged, Hungary;* ^*2*^ *Doctoral School of Experimental and Preventive Medicine, University of Szeged, Hungary;* ^*3*^ *Department of Pediatrics, Albert Szent‐Györgyi Faculty of Medicine, University of Szeged, Hungary;* ^*4*^ *Department of Neurology, Albert Szent‐Györgyi Faculty of Medicine, University of Szeged, Hungary;* ^*5*^ *HUN‐REN‐SZTE Neuroscience Research Group, University of Szeged, Hungary*

## EPV‐0592

### Use of corticosteroid‐containing greater occipital nerve injections in the perinatal period

#### N. Christou ^1^; A. Gimson^1^; F. Greenwood^2^; S. Maniataki^2^; D. Wei^2^; S. Afridi^3^; F. Puledda^1^; P. Goadsby^4^

##### ^*1*^ *NIHR King's Clinical Research Facility, & SLaM Biomedical Research Centre, and The Wolfson Sensory, Pain and Regeneration Centre (SPaRC), Institute of Psychiatry, Psychology and Neuroscience, King's College London, UK;* ^*2*^ *Department of Neurology, Kings College Hospital, London UK;* ^*3*^ *Guy's and St Thomas NHS Trus, London UKt;* ^*4*^ *NIHR King's Clinical Research Facility, & SLaM Biomedical Research Centre, and The Wolfson Sensory, Pain and Regeneration Centre (SPaRC), Institute of Psychiatry, Psychology and Neuroscience, King Abdullah University of Science & Technology, Thuwal, Saudi Arabia*

## Higher cortical functions

## EPV‐0593

### Baseline kynurenine pathway metabolites and post‐stroke cognitive performance: a prospective cohort study

#### E. Abed

##### Neurology Department, Faculty of Medicine, Al‐Azhar University, Cairo, Egypt

## EPV‐0594

### Functional MRI‐informed EEG identification of active cortical regions in a silent naming task

#### L. Zanchi^1^; J. Lanzone^2^; S. Basaia^1^; L. Lumaca^1^; A. Riva^1^; M. Cursi^3^; E. Canu ^4^; M. Filippi^5^; F. Agosta^4^

##### ^*1*^ *Neuroimaging Research Unit, Division of Neuroscience, IRCCS San Raffaele Scientific Institute, Milan, Italy; and Neurotech Hub, Vita‐Salute San Raffaele University, Milan, Italy;* ^*2*^ *Neurophysiology Service, IRCCS San Raffaele Scientific Institute, Milan, Italy; and Neurotech Hub, Vita‐Salute San Raffaele University, Milan, Italy;* ^*3*^ *Neurophysiology Service, IRCCS San Raffaele Scientific Institute, Milan, Italy;* ^*4*^ *Neuroimaging Research Unit, Division of Neuroscience, and Neurology Unit, IRCCS San Raffaele Scientific Institute, Milan, Italy; and Neurotech Hub, Vita‐Salute San Raffaele University, Milan, Italy;* ^*5*^ *Neuroimaging Research Unit, Division of Neuroscience, Neurology Unit, Neurorehabilitation Unit, and Neurophysiology Service, IRCCS San Raffaele Scientific Institute, Milan, Italy; and Neurotech Hub, Vita‐Salute San Raffaele University, Milan, Italy*

## EPV‐0595

### Functional neuroplasticity in response to a giant left‐sided arachnoid cyst: An fMRI and DTI analysis of language preservation

#### S. Cinar ^1^; G. Caliskan Ersu^1^; F. Bayramoglu^2^; M. Ozdag^1^

##### ^*1*^ *Department of Neurology, Sultan Abdulhamid Han Training and Research Hospital, University of Health Sciences, Istanbul, Türkiye;* ^*2*^ *Department of Radiology, Sultan Abdulhamid Han Training and Research Hospital, University of Health Sciences, Istanbul, Türkiye*

## EPV‐0596

### Method for assessing stereognosis to evaluate the morphofunctional organization of tertiary cortical areas in preschool children

#### L. Ziyakhodzhaeva; N. Hasanova

##### Tashkent State Medical University, Tashkent, Uzbekistan

## EPV‐0597

### Mixed reality‐based functional screening for hemispatial neglect in acute right parietal stroke

#### F. Özel Uvalıoğlu; G. Yavaş; B. Acar Çinleti; T. Mengi

##### Department of Neurology, Izmir Democracy University, Buca Seyfi Demirsoy Training and Research Hospital, İzmir, Türkiye

## EPV‐0598

### Oculomotor apraxia as a window into visuomotor network stroke

#### A. Popescu; M. Trandafir; E. Ivan; C. Sirbu; T. Vasile

##### Neurology Department, “Dr. Carol Davila” Central Military Emergency University Hospital, Bucharest, Romania

## EPV‐0599

### Survey on the actual conditions of traumatic brain injury resulting from bicycle traffic accidents in Japan

#### S. Terajima; S. Aoki; K. Yoshida; M. Abo

##### Department of Rehabilitation Medicine, The Jikei University School of Medicine, Minato, Japan

## EPV‐0600

### Telemedicine‐based assessment of limb apraxia in neurodegenerative diseases: Feasibility and diagnostic implications

#### E. Smaragdaki; I. Stamelos; E. Angelopoulou; K. Vourou; D. Kontaxopoulou; J. Papatriantafyllou; L. Stefanis; V. Konstantinidis; S. Papageorgiou

##### First Department of Neurology, Medical School, National and Kapodistrian University of Athens, Eginition Hospital, Athens, Greece

## History of neurology

## EPV‐0601

### A concept of autoimmune etiology of ALS‐ a historical evolution and current state

#### R. Kanarkowski

##### Warsaw University, Warsaw, Poland

## EPV‐0602

### Historical perspectives on epilepsy care in rural areas in the Philippines

#### K. Po

##### Department of Internal Medicine, Bacolod Queen of Mercy Hospital, Bacolod City, Negros Occidental, Philippines

## EPV‐0603

### Merging culture‐bound hyperstartle syndromes into a single nosological entity: What survived from Tourette's original description of tic disease?

#### R. Damjanovic ^1^; J. Stamenovic^1,4^; I. Stankovic^2,5^; D. Aleksic^3,6^

##### ^*1*^ *Clinic for Neurology, University Clinical Center of Nis, Nis, Serbia;* ^*2*^ *Clinic for Neurology, University Clinical Center of Serbia, Belgrade, Serbia;* ^*3*^ *Clinic for Neurology, University Clinical Center of Kragujevac, Kragujevac, Serbia;* ^*4*^ *Faculty of Medicine, University of Nis, Serbia;* ^*5*^ *Faculty of Medicine, University of Belgrade, Serbia;* ^*6*^ *Faculty of Medical Sciences, University of Kragujevac, Serbia*

## EPV‐0604

### Neuro‐FakeNews: Major historical errors in neurology

#### J. Sosa Luis; D. Machado Sampedro; L. Portela Martínez; M. Lozano López; R. Boto Martínez; G. Lafuente Gómez

##### Neurology Department, Hospital General Universitario Gregorio Marañón, Madrid, Spain

## EPV‐0605

### The awards of Hungarian neurology in the last 90 years

#### D. Bereczki

##### Department of Neurology, Semmelweis University, Budapest, Hungary

## Infectious diseases

## EPV‐0606

### A diagnostic paradox: Central nervous system tuberculosis in the setting of parvovirus B19 infection

#### H. Moutran‐Barroso; A. Baez‐Orozco; G. Bonfante‐Zakzuk; S. Martínez‐García; G. Sánchez‐Rincón

##### Los Cobos Medical Center, Universidad El Bosque, Bogotá, Colombia

## EPV‐0607

### A treatable mimic of dementia: Neurosyphilis with progressive speech apraxia and brain atrophy—Two cases and literature review

#### G. Marchisella

##### Department of Biomedical, Metabolic and Neurosciences, Università degli Studi di Modena e Reggio Emilia, Modena, MO, Italy

## EPV‐0608

### A wicked malady tale‐acute dystonia and residual hemidystonia following viral infection in high‐risk acute lymphoblastic leukaemia pediatric patient

#### P. Batra ^1^; P. Pathak^2^

##### ^*1*^ *Resident Department of Neurology, IMS BHU, India;* ^*2*^ *Department of Neurology, IMS BHU, Varanasi, India*

## EPV‐0609

### Aseptic meningitis revisited: The meninges strike back

#### D. de Pinho; L. Rocha; H. Felgueiras; M. Branco; M. Malaquias

##### Neurology Department, Unidade Local de Saúde de Gaia e Espinho, Portugal

## EPV‐0610

### Association of level of csf sugar, protein and cells with mortality amongst cases with bacterial and tubercular meningitis

#### V. Nandmer

##### Gandhi Medical College Hamidia Hospital, Bhopal, India

## EPV‐0611

### Clinical and neurophysiological features of post‐COVID and Diabetic Polyneuropathy

#### S. Usmanov; Y. Madjidova

##### Neurology, Tashkent Children's Hospital, Tashkent, Uzbekistan

## EPV‐0612

### Clinical cases of bacterial meningoencephalitis during pregnancy

#### M. Vasilieva; A. Bondarciuc; A. Bejenari; E. Zota; D. Belitei; G. Oglinda‐Catirau; S. Groppa

##### Institute of Emergency Medicine, Clinical Department of Neurology, Epileptology and Internal Disease, Chisinau, Republic of Moldova

## EPV‐0613

### Clinical spectrum and cerebrospinal fluid predictors of adverse outcomes in Central Nervous System Tuberculosis – A retrospective cohort study

#### V. Velummylum; A. Pathirage; P. Ruwanpathirana; B. Senanayake

##### Institute of Neurology, National hospital of Sri Lanka

## EPV‐0614

### Diagnostic challenges in multifocal brain lesions: A case of HIV‐related toxoplasmic encephalitis

#### A. Afanasieva; M. Liksunova; M. Bodnar; T. Slobodin

##### Department of Neurology, LLC “Dobrobut‐Clinic”, Kyiv, Ukraine

## EPV‐0615

### Disseminated neurocysticercosis: Clinical manifestations, diagnostic challenges, and therapeutic outcomes

#### A. Kumar ^1^; N. Lall^2^; D. Joshi^1^; A. Dwivedi^1^; V. Singh^1^; A. Pathak^1^; R. Chaurasia^1^; V. Mishra^1^; N. Srivastava^1^

##### ^*1*^ *Institute of Medical Sciences, Banaras Hindu University, Varanasi, India;* ^*2*^ *Indira Gandhi Institute of Medical Sciences, Patna, India*

## EPV‐0616

### Elsberg syndrome presenting as acute lumbosacral radiculitis: two case reports

#### N. Christou; A. Pilavas; C. Pilava; I. Kalpachtsidou; E. Angastinioti; S. Skarpari; P. Tsouloupas; S. Lambrianides

##### Apollonion Private Hospital, Nicosia, Cyprus

## EPV‐0617

### Epstein–barr virus–related neurological disease: Two cases illustrating heterogeneous clinical presentations

#### B. Nitish Gopal ^1^; A. Prabhu^2^

##### ^*1*^ *Department of Neurology, Kastruba Medical College, Manipal Academy of Higher Education, Manipal, Karnataka, India;* ^*2*^ *Department of Neurology, Kastruba Medical College, Manipal Academy of Higher Education, Manipal, Karnataka, India*

## EPV‐0618

### Facial diplegia as the initial manifestation of HIV infection: A case report

#### C. Toilos; K. Ntoskas; I. Dedes; A. Moustafellou; I. Spanou; A. Tsagkaropoulos; E. Kouremenos

##### Department of Neurology, 251 Air Force General Hospital, Athens, Greece

## EPV‐0619

### Froin syndrome as a diagnostic marker of tuberculous cauda equina arachnoiditis in inflammatory polyradiculopathy

#### A. Jehanno ^1^; V. Leclercq^1^; N. Sadeghi^2^; M. Leclercq^1^; M. Bisciglia^1^

##### ^*1*^ *Department of Neurology, HUB Hôpital Erasme, Brussels, Belgium;* ^*2*^ *Department of Radiology, HUB Hôpital Erasme, Brussels, Belgium*

## EPV‐0620

### Fulminant Listeria monocytogenes meningoencephalitis in an immunocompromised patient: A case report

#### E. Molodveanu^1^; D. Rusu^2^; I. Murasan ^1^; T. Kerekes^3^; S. Galbineanu^4^; T. Melicianu^5^; C. Kakucs^3^; C. Falup‐Pecurariu^2^

##### ^*1*^ *Department of Neurology, County Clinic Hospital, Brasov, Romania;* ^*2*^ *Department of Neurology, County Clinic Hospital, Transilvania University, Brasov, Romania;* ^*3*^ *Department of Neurosurgery, County Clinic Hospital, Brasov, Romania;* ^*4*^ *Department of Intensive Care, County Clinic Hospital, Brasov, Romania;* ^*5*^ *Infectious Disease, County Clinic Hospital, Brasov, Romania*

## EPV‐0621

### Herpes simplex encephalitis presenting as super – refractory status epilepticus with retrograde amnesia sequelae in an immunocompetent adult

#### D. Lamela

##### Cebu South Medical Center, Talisay, Philippines

## EPV‐0622

### Intramedullary abscess in a non‐surgical candidate: Is medical management a viable option?

#### A. Orejón Sánchez; A. Sánchez Rodriguez; D. Alonso Vallín; B. Quintana López; P. Díaz Aguirre; C. Castaño Ron; M. Martínez Palicio

##### Neurologia, Hospital Universitario de Cabueñes, Gijon, Asturias

## EPV‐0623

### Isolated oculomotor nerve palsy as an early neurological manifestation of HIV infection

#### I. Abourachida; S. Rachidi; S. Zahlane; M. Chraa; N. Louhab

##### Neurology, Mohammed VI University Hospital, Faculty of Medicine and Pharmacy, Cadi Ayyad University, Marrakech, Morocco

## EPV‐0624

### Keeping the great imitator in mind: A case of syphilitic encephalitis

#### T. Moncada Cordeiro ^1^; C. Rosado Coelho^2^; F. Antunes^2^; I. Menezes Cordeiro^2^

##### ^*1*^ *Neurology Department, Unidade Local de Saúde de São José, Lisbon, Portugal;* ^*2*^ *Neurology Department, Unidade Local de Saúde de São José, Lisbon, Portugal; Neurophysiology Unit, Unidade Local de Saúde de São José, Lisbon, Portugal*

## EPV‐0625

### Management of Nocardia farcinica brain abscess with subdural empyema and septic sinus thrombosis in an elderly immunocompetent patient

#### L. Minkova ^1^; A. Oberson^1^; M. Thurnheer Zürcher^2^; L. Damonti^2^; C. Baumgartner^2^; B. Surial^2^; J. Abu‐Isa^3^; L. Felger^3^; H. Mazyad^3^; L. Spitz^3^; A. Kähler^4^; C. Aubert^4^; D. Bertschi^1^

##### ^*1*^ *Department of Geriatrics, Bern University Hospital, Inselspital, University of Bern, Bern, Switzerland;* ^*2*^ *Department of Infectious Diseases, Bern University Hospital, Inselspital, University of Bern, Switzerland;* ^*3*^ *Department of Neurosurgery, Bern University Hospital, Inselspital, University of Bern, Bern, Switzerland;* ^*4*^ *Department of General Internal Medicine, Inselspital, Bern University Hospital, University of Bern, Bern, Switzerland*

## EPV‐0626

### Meningoencephalitis caused by streptococcus salivarius in a 63‐year‐old patient with no significant medical history

#### K. Ntoskas^1^; C. Toilos ^1^; P. Petrikkos^2^

##### ^*1*^ *Department of Neurology, Hellenic Air Force General Hospital, Athens, Greece;* ^*2*^ *Department of Internal Medicine, Hellenic Air Force General Hospital, Athens, Greece*

## EPV‐0627

### mRNA cytokine profiling in tuberculous meningitis associated infarctions

#### R. Shukla ^1^; J. Klalita^2^; P. Pandey^2^; V. Singh^1^

##### ^*1*^ *Department of Radiodiagnosis, Sanjay Gandhi Post Graduate Institute of Medical Sciences, Lucknow, Uttar Pradesh, India;* ^*2*^ *Department of Neurology, Sanjay Gandhi Post Graduate Institute of Medical Sciences, Lucknow, Uttar Pradesh, India*

## EPV‐0628

### Multiple cerebral space‐occupying lesions: From presumed metastases to the diagnosis of cerebral tuberculosis

#### A. Orejón Sánchez; L. González Fernández; V. Garcia Garcia; B. Quintana López; D. Alonso Vallín

##### Neurologia. Hospital Universitario de Cabueñes, Gijon, Asturias

## EPV‐0629

### Neuroaspergillosis mimicking acute polyradiculoneuritis

#### B. Soare; L. Chirita‐Craciun; B. Popescu

##### Department of Neurology, Colentina Clinical Hospital, Bucharest, Romania

## EPV‐0630

### Atypical neurobrucellosis: Lymphocytic meningoencephalitis with a solitary cystic brain lesion

#### A. Escobar Padilla; K. Alvarez Agoues; D. Ayuso Garcia; N. Andres Marín; N. Diez González; M. Walcker; A. Vinagre Aragon; F. González López; X. Kortajarena Urkola

##### Servicio de Neurologia, Hospital Universitario Donostia – Donostia Ospitalea, Servicio de Enfermedades Infecciosas Hospital Universitario Donostia – Donostia Ospitalea, Donostialdea, Spain

## EPV‐0631

### Neuromelioidosis – A life threatening masquerader

#### A. Pai ^1^; D. Varma^2^; N. Gupta^2^; P. Tirlangi^2^; S. Pv^2^

##### ^*1*^ *Department of Neurology, Kasturba Medical College, Manipal. , Manipal Academy of Higher Education, Manipal, India;* ^*2*^ *Department of Infectious Diseases, Kasturba Medical College, Manipal Academy of Higher Education Manipal, India*

## EPV‐0632

### Neurosyphilis presenting with rapidly progressive dementia and status epilepticus: A case report

#### N. Kahveci Erdemir; A. Çolakoğlu; A. Aksoy Gündoğdu

##### Department of Neurology, Tekirdag Namik Kemal University, Tekirdag, Türkiye

## EPV‐0633

### Non‐convulsive status epilepticus‐misdiagnosis in the Creutzfeldt‐Jakob disease. Clinical cases

#### M. Vasilieva; G. Oglinda‐Catirau; C. Munteanu; M. Junbei; S. Groppa

##### Institute of Emergency Medicine, Clinical Department of Neurology, Epileptology and Internal Disease, Chisinau, Republic of Moldova

## EPV‐0634

### Painful ophthalmoplegia and diplopia: Beyond Tolosa‐Hunt síndrome

#### G. Clementson Delgado; C. Algar Ramírez; G. García Martín; P. Serrano Castro

##### Neurology, Carlos Haya Regional University Hospital, Málaga, Spain

## EPV‐0635

### PD‐L1 immune checkpoint as a potential target for PML immunotherapy: Insights from 2 cases

#### B. Brilot ^1^; A. Silva Gomes^1^; C. Graux^2^; G. Crochet^2^; G. Martin‐Blondel^3^; N. Lambert^4^; F. London^1^

##### ^*1*^ *Department of Neurology, CHU UCL Namur, site Godinne, Yvoir, Belgium;* ^*2*^ *Department of Hematology, CHU UCL Namur, site Godinne, Yvoir, Belgium;* ^*3*^ *Department of Infectious and Tropical Diseases, University Hospital of Toulouse, Toulouse, France;* ^*4*^ *Department of Neurology, University Hospital of Liège, Liège, Belgium*

## EPV‐0636

### Post‐infectious encephalitis associated with coxiella burnetii: A case with aphasia and focal seizures

#### L. Caballero Sánchez; M. Capra; R. Alfonso González; M. Vargas Cobos; A. De Luca; C. Gómez López de San Román; I. Bermejo Casado; D. Cerdán Santacruz; A. Castrillo Sanz; A. Mendoza Rodríguez

##### General Hospital of Segovia

## EPV‐0637

### Post‐infectious upper limb monoradiculopathy following dengue infection: A case report

#### N. Narine; E. Hatim

##### Internal Medicine, Enmore Regional Hospital, Guyana

## EPV‐0638

### Post‐rituximab progressive multifocal leukoencephalopathy treated with pembrolizumab

#### G. Diniz de Pinho ^1^; J. Bonifácio Vítor^1^; A. Calado^1^; D. Coiteiro^2^; P. Vilela^3^; M. Mafra^4^; J. Carda^5^

##### ^*1*^ *Neurology Department, Hospital da Luz, Lisboa, Portugal;* ^*2*^ *Neurosurgery Department, Hospital da Luz, Lisboa, Portugal;* ^*3*^ *Neuroradiology Department, Hospital da Luz, Lisboa, Portugal;* ^*4*^ *Anatomical Pathology Department, Hospital da Luz, Lisboa;* ^*5*^ *Hematology Department, Hospital da Luz, Lisboa*

## EPV‐0639

### Post–SARS‐CoV‐2 acute disseminated encephalomyelitis in a 4‐year‐old child: A case report

#### E. Capestru ^1^; S. Hadjiu^1^; A. Iarovoi^2^; A. Tosciuc^2^; L. Chiosea^2^; T. Covalschi^1^

##### ^*1*^ *State University of Medicine and Pharmacy “Nicolae Testemiţanu”, Pediatric Neurology Clinic of the Department of Pediatrics, Chișinău, Moldova;* ^*2*^ *“Valentin Ignatenco” Municipal Clinical Children's Hospital, Chișinău, Moldova*

## EPV‐0640

### Progressive multifocal leukoencephalopathy in a patient with pancytopenia and pulmonary tuberculosis: A case report

#### Z. Gunes; A. Kula; E. Gursoy; Z. Matur

##### Department of Neurology, Bezmialem Vakıf University Faculty of Medicine, Istanbul, Türkiye

## EPV‐0641

### Progressive primary aphasia with asymmetric temporal lobe atrophy as an initial presentation of neurosyphilis: A case report

#### C. Santos Martín; F. Sierra Hidalgo; F. Azcárate Díaz

##### Neurology, Hospital Universitario Infanta Leonor, Madrid, Spain

## EPV‐0642

### Pseudobulbar palsy due to Herpes Simplex 1 meningoencephalitis with bilateral opercular involvement

#### J. Fins ^1^; L. Silva^1^; A. Surreira^2^; C. Silva^2^; D. Pereira^1^; I. Laranjinha^1^

##### ^*1*^ *Department of Neurology, Hospital of Santo António, Porto, Portugal;* ^*2*^ *Department of Infectious Diseases, Hospital of Santo António, Porto, Portugal*

## EPV‐0643

### Rapidly progressive dementia as a diagnostic challenge: A case of probable Creutzfeldt–Jakob disease

#### I. Murasan ^1^; L. Ungureanu^2^; A. Drujescu^1^; G. Pasat^1^; S. Berzunteanu^1^; R. Margarit^2^; C. Falup‐Pecurariu^2^

##### ^*1*^ *Department of Neurology, County Clinic Hospital, Brasov, Romania;* ^*2*^ *Department of Neurology, County Clinic Hospital, Transilvania University Brasov, Romania*

## EPV‐0644

### Recurrent multifocal infarcts in a heart transplant patient with disseminated phaeohyphomycosis

#### A. Acar ^1^; A. Malati^1^; C. Bullock^2^; K. Gage^2^; O. Sokumbi^3^; S. English^1^

##### ^*1*^ *Department of Neurology, Mayo Clinic, Jacksonville, Florida, USA;* ^*2*^ *Department of Radiology, Mayo Clinic, Jacksonville, Florida, USA;* ^*3*^ *Department of Dermatology, Mayo Clinic, Jacksonville, Florida, USA*

## EPV‐0645

### Survival after primary amebic meningoencephalitis: A rare case of naegleria fowleri infection in a diabetic adult patient

#### S. Mekhchoun; Y. Zakaria; S. Zahlane; K. Balili; M. Chraa; N. Louhab

##### Department of Neurology, Mohammed VI University Hospital, Marrakech, Morocco

## EPV‐0646

### The burden of persisting COVID‐19 associated loss of smell on quality of life, health and mood: Baseline data from the SMELL‐trial

#### N. De Cleene; K. Seppi; B. Heim

##### Department of Neurology, Medical University of Innsbruck (MUI), Austria

## EPV‐0647

### The unseen threat: A two‐month cluster of probable sporadic CJD in Eastern India

#### S. Puri; I. Aleena; D. Chakraborty

##### Department of Neurology, Apollo Multispecialty Hospitals, Kolkata, India

## EPV‐0648

### Tuberculous meningitis with negative microbiological studies: a diagnostic challenge

#### M. Jiménez Alonso; C. Mercado Begara; A. Morillas Pinteño; A. Espinosa Cortés; P. Nieto Palomares

##### Neurology Service, Hospital Universitario de Jaén, Jaén, España

## EPV‐0649

### Varicella zoster virus associated meningoradiculitis in an elderly patient: A case report

#### Y. Tan; S. Goh; Y. Koh; G. Yiin

##### Department of Neurology, Changi General Hospital, Singapore

## EPV‐0650

### Vasculopathy in central nervous system tuberculosis: A retrospective cohort study from Sri Lanka

#### A. Pathirage; V. Velummylum; P. Ruwanpathirana; B. Senanayake

##### Institute of Neurology, National Hospital, Sri Lanka

## EPV‐0651

### When Bell's palsy is not idiopathic: A diagnostic pitfall leading to Ramsay Hunt syndrome with brain encephalitis

#### A. Zurawska ^1,2^; M. Czarniawska^2^; M. Mycko^1,2^

##### ^*1*^ *Department of Neurology and Neurosurgery, University of Warmia and Mazury, Olsztyn, Poland;* ^*2*^ *Department of Neurology and Neurosurgery, University Hospital in Olsztyn, Poland*

## EPV‐0652

### When shingles strikes the brain: A case of VZV vasculopathy with ophthalmoplegia and stroke

#### V. Singh ^1^; D. Arora^1^; P. Sengar^2^; R. Chaurasia^1^; A. Kumar^1^; A. Pathak^1^; V. Mishra^1^; D. Joshi^1^

##### ^*1*^ *Department of Neurology, Institute of Medical Sciences, Banaras Hindu University, Varanasi, Uttar Pradesh;* ^*2*^ *Department of Pathology, Institute of Medical Sciences, Banaras Hindu University, Varanasi, Uttar Pradesh*

## EPV‐0653

### When SSPE begins with blindness: A rare presentation with recurrent visual loss and hemimyoclonus

#### V. Singh ^1^; P. Sengar^2^; R. Chaurasia^1^; A. Pathak^1^; A. Kumar^1^; V. Mishra^1^; D. Joshi^1^

##### ^*1*^ *Department of Neurology, Institute of Medical Sciences, Banaras Hindu University, Varanasi, Uttar Pradesh;* ^*2*^ *Department of Pathology, Institute of Medical Sciences, Banaras Hindu University, Varanasi, Uttar Pradesh*

## Motor neurone diseases

## EPV‐0654

### Care‐related regret and procrastination as determinants of burnout among healthcare professionals managing spinal muscular atrophy

#### L. Carrera‐García^1^; P. Castro ^2^; V. Ejarque^3^; A. León^4^; C. Álvarez^2^; J. Mauriño^2^; A. Nascimento^1^

##### ^*1*^ *Neuromuscular Unit, Department of Neurology, Hospital Sant Joan de Déu, Barcelona, Spain;* ^*2*^ *Medical Department, Roche Farma, Madrid, Spain;* ^*3*^ *Department of Gastroenterology, Hepatology and Nutrition, Hospital San Joan de Déu, Barcelona, Spain;* ^*4*^ *Phoniatrics and Speech Therapy Unit, Hospital Universitari Vall d´Hebron, Barcelona, Spain*

## EPV‐0655

### Clustering and personalized prognostic prediction in patients with amyotrophic lateral sclerosis

#### J. Zhang; S. Ye; L. Chen; X. Liu; D. Fan

##### Department of Neurology, Peking University Third Hospital, Beijing, China

## EPV‐0656

### Contribution of nucleus accumbens and amygdala subnuclei to the emergence of positive and negative behavioural symptoms in ALS

#### G. Scacciatella ^1^; A. Maranzano^2^; A. Cocuzza^3^; V. Patisso^2^; S. Pierro^1^; M. Passaretti^4^; S. Torre^2^; B. Poletti^2^; A. Doretti^2^; C. Morelli^2^; E. Aiello^2^; C. Cinnante^5^; J. Pereira^4^; V. Silani^6^; F. Verde^2^; N. Ticozzi^6^

##### ^*1*^ *Neurology Residency Program, Università degli Studi di Milano, Milan, Italy;* ^*2*^ *Department of Neurology and Laboratory of Neuroscience, IRCCS Istituto Auxologico Italiano, Milan, Italy;* ^*3*^ *IUSS Cognitive Neuroscience (ICoN) Center, Institute for Advanced Studies, Pavia, Italy;* ^*4*^ *Department of Clinical Neuroscience, Karolinska Institutet, Stockholm, Sweden;* ^*5*^ *Department of Radiology and Diagnostic Imaging, IRCCS Istituto Auxologico Italiano, Milan, Italy;* ^*6*^ *Department of Pathophysiology and Transplantation Dino Ferrari Center, Università degli Studi di Milano, Milan, Italy*

## EPV‐0657

### Does genetic background influence serum creatine phosphokinase levels in amyotrophic lateral sclerosis?

#### A. Gharbi; I. Sghaier; A. Mansouri; Y. Abida; A. Atrous; A. Souissi; A. Gargouri; I. Kacem; R. Gouider

##### Neurology Department, LR18SP03 and Clinical Investigation Center Neurosciences and Mental Health – University Hospital Center Razi‐Mannouba, Tunis, Tunisia – Faculty of Medicine of Tunis, Tunis El Manar University, Tunis, Tunisia

## EPV‐0658

### Dropped head syndrome identifies a malignant phenotype in FUS‐associated ALS

#### V. Viric ^1^; S. Peric^1^; A. Marjanovic^2^; A. Palibrk^1^; I. Bozovic^1^; M. Vukojevic^1^; N. Andrejic^1^; V. Ivanovic^1^; I. Basta^1^; Z. Stevic^2^

##### ^*1*^ *Department for neuromuscular disorders, Neurology Clinic, Belgrade, University Clinical Centre of Serbia;* ^*2*^ *Medical Faculty, University of Belgrade, Serbia*

## EPV‐0659

### Early and mild cognitive‐behavioral changes independently predict caregiver burden in ALS: a longitudinal study using the MBNI classification

#### F. Shahrezaei; M. Spisto; L. Aruta; R. Dubbioso

##### Department of Neuroscience and Reproductive and Odontostomatological Sciences, University of Naples Federico II, Naples, Italy

## EPV‐0660

### Home‐based tDCS in amyotrophic lateral sclerosis: Effects on clinical progression and neurodegeneration biomarkers

#### J. Della Toffola; M. Quagliotto; E. Ricci; A. Bratina; P. Manganotti; A. Benussi

##### Neurology Unit, Hospital Care Department of Medicine, Azienda Sanitaria Universitaria Giuliano Isontina, Department of Medical, Surgical and Health Sciences, University of Trieste, Trieste, Italy

## EPV‐0661

### Immune aging connects chronic gastritis and Parkinson's disease through a gastric‐brain network

#### Z. Chen ^1^; Z. Xu^2^; C. Zhang^1^; L. Lei^1^; Z. Li^3^

##### ^*1*^ *Institute of Information on Traditional Chinese Medicine, China Academy of Chinese Medical Sciences, Beijing, China;* ^*2*^ *Dong fang Hospital Beijing University of Chinese Medicine, Beijing, China;* ^*3*^ *The Third Affiliated Hospital of Beijing University of Chinese Medicine, Beijing, China*

## EPV‐0662

### Incidence and prevalence of amyotrophic lateral sclerosis in Curitiba, Brazil

#### O. Hernandez Fustes ^1^; N. Faria^1^; B. Maçaneiro^1^; C. Kamoi Kay^1^; R. Dal‐Pra Ducci Cirino^1^; P. Lorenzoni^1^; P. Rodrigues^1^; L. Fraga^1^; R. Scola^1^; P. Rosignoli^2^

##### ^*1*^ *Universidade Federal do Paraná, Curitiba, Brazil;* ^*2*^ *Secretaria da Saúde do Paraná, Curitiba, Brazil*

## EPV‐0663

### Long‐term respiratory and clinical evaluation in adult SMA type 2 patients treated with risdiplam: A 48‐month observational study

#### S. Loprieno ^1^; F. Torri^1^; G. Iaquinta^1^; M. Gherardi^2^; M. Serradori^2^; L. Carrozzi^3^; G. Siciliano^1^; G. Ricci^1^

##### ^*1*^ *Department of Clinical and Experimental Medicine, University of Pisa, Pisa, Italy;* ^*2*^ *Pulmonology Unit, Cardiothoracic and Vascular Department, University Hospital of Pisa, Pisa, Italy;* ^*3*^ *Department of Surgical, Medical and Molecular Pathology and Critical Care Medicine, University of Pisa Pisa, Italy*

## EPV‐0664

### Motor neuron diseases in latvia: A nationwide epidemiology study

#### V. Krutovs ^1^; A. Grosmane^1^; R. Kažmere^2^; M. Roddate^1^; G. Ķauķe^1^; D. Grosa^2^; S. Šetlere^3^; M. Dīriks^3^; G. Karelis^2^; V. Ķēniņa^1^

##### ^*1*^ *Pauls Stradins Clinical University Hospital, Department of Neurology, Riga, Latvia;* ^*2*^ *Riga East University Hospital, Department of Neurology and Neurosurgery, Riga, Latvia;* ^*3*^ *Children's Clinical University Hospital, Department of Neurology and Neurosurgery, Riga, Latvia*

## EPV‐0665

### Our experience with intrathecal tofersen treatment in patients with SOD1‐associated ALS

#### B. Csendes; Á. Palásti; Z. Nagy; Z. Grosz; K. Surján; M. Molnár

##### Institute of Genomic Medicine and Rare Disorders, Semmelweis University, Budapest, Hungary

## EPV‐0666

### Real‐world treatment with risdiplam in adults with Spinal Muscular Atrophy (SMA): A single center experience

#### H. Durmus; S. Mergen; N. Turapoğlu; A. Cakar; Y. Parman

##### Department of Neurology, Istanbul Faculty of Medicine, Istanbul University, Istanbul, Türkiye

## EPV‐0667

### Results of the molecular genetic study of amyotrophic lateral sclerosis (ALS) in the Republic of Belarus

#### Y. Rushkevich^1^; K. Malhina ^1^; V. Haliyeuskaya^1^; A. Gusina^2^; K. Andrianova^1^; A. Stalybko^2^; A. Vakula^2^

##### ^*1*^ *Republican Scientific and Practical Center of Neurology and Neurosurgery, Minsk, Belarus;* ^*2*^ *Republican Scientific and Practical Center Mother and Child, Minsk, Belarus*

## EPV‐0668

### Risdiplam in adult patients with spinal muscular atrophy: 24‐month follow‐up

#### D. García‐Estévez ^1^; M. Becerra Fernández^2^; N. Suarez Pérez^3^; J. Rodríguez Garrido^4^

##### ^*1*^ *Neurology Service, Complejo Hospitalario Universitario de Ourense, Ourense, Spain;* ^*2*^ *EXPRELA Research Group, CICA – Centro Interdisciplinar de Química e Bioloxía, Departamento de Bioloxía, Facultade de Ciencias, Universidade da Coruña, A Coruña, Spain;* ^*3*^ *Neurology Service, Nursing Department, Complejo Hospitalario Universitario de Ourense, Ourense, Spain;* ^*4*^ *Clinical Laboratory Service, Complejo Hospitalario Universitario de Ourense, Ourense, Spain*

## EPV‐0669

### Rising disability from motor neuron disease across Europe: Mapping long‐term trends in the regional burden

#### S. Wasti^1^; A. Wasti^2^; M. Rahim ^1^

##### ^*1*^ *Department of Neurology, King Edward Medical University, Lahore, Pakistan;* ^*2*^ *Department of Neurology, Continental Medical College, Lahore, Pakistan*

## EPV‐0670

### Safety of memantine in adults with amyotrophic lateral sclerosis: A systematic review

#### L. Hamzeh Hamzeh^1^; E. Seyed^2^; M. Ayman Elgendy ^3^; D. Heib^4^; B. I. Sadek^5^; C. O. S. Mohammed^6^; K. El Refaei^7^; Y. F. Soliman^8^; N. Eldmrdash^9^; M. Shaaban Abdelgalil^10^

##### ^*1*^ *Faculty of Medicine, Caucasus International University, Tbilisi, Georgia;* ^*2*^ *Faculty of Medicine, Al‐Neelain University, Khartoum, Sudan* ^*3*^ *Faculty of Medicine, Misr University for Science and Technology, Giza, Egypt;* ^*4*^ *Faculty of Medicine, Al‐Quds University, Jerusalem, Palestine;* ^*5*^ *Faculty of Medicine, Assiut university, Assiut, Egypt;* ^*6*^ *Faculty of Medicine, Kirkuk University, Kirkuk, Iraq;* ^*7*^ *School of Medicine, Newgiza University (NGU), Giza, Egypt;* ^*8*^ *Faculty of Medicine, Mansoura National University, Dakahlia, Egypt;* ^*9*^ *Faculty of Medicine, Delta University for Science and Technology, Dakahlia, Egypt;* ^*10*^ *Faculty of Medicine, Ain‐Shams University, Cairo, Egypt*

## EPV‐0671

### Sensry nerve involvement in motor neuron disease

#### A. Pietrzak^1^; Ł. Rzepiński ^2,3^

##### ^*1*^ *Department of Neurology, 10th Military Research Hospital and Polyclinic, Bydgoszcz, Poland;* ^*2*^ *Department of Neurology, 10th Military Research Hospital and Polyclinic, Bydgoszcz, Poland;* ^*3*^ *Department of Clinical Medicine, Faculty of Medicine, University of Science and Technology, Bydgoszcz, Poland.*

## EPV‐0672

### Systematic review of fluid biomarkers in Parkinson's disease for tracking progression and early diagnosis

#### L. García Kornhauser; M. Sierra Reyes; S. Pérez Servín; A. Guan Salazar

##### Universidad de Guanajuato, División Ciencias de la salud Licenciatura Médico Cirujano León Guanajuato México, Mexico

## EPV‐0673

### TDP‐43 deficiency‐induced triglyceride catabolism disorder in ALS: Lipid droplets accumulation, insufficient energy supply and neuronal degeneration

#### T. Zhang ^1^; Q. Liao^1^; K. Yan^2^; K. Qiu^1^; J. Zhou^1^; Y. Liu^1^; F. Bi^1^

##### ^*1*^ *The Fifth Affiliated Hospital of Sun Yat‐sen University, Guangzhou, China;* ^*2*^ *The Second Affiliated Hospital of Xinjiang Medical University*, *Ürümqi, China*

## EPV‐0674

### Tracking the individualized longitudinal tempo of ALS through clinical and inflammatory trajectories

#### C. Erkalayci Kerekli; E. Gozke

##### Neurology Department, University of Health Sciences Fatih Sultan Mehmet Training and Research Hospital, Istanbul, Türkiye

## EPV‐0675

### Unraveling the mystery of alien hand syndrome: When your hand has a mind of its own. Abstract

#### K. Moghib^1^; M. Meshref ^2^

##### ^*1*^ *Kasralainy Medical School, Cairo University, Cairo, Egypt;* ^*2*^ *Department of Neurology, Faculty of Medicine, Al‐Azhar University, Cairo, Egypt*

## EPV‐0676

### Wearable sensors detect postural abnormalities in spinal muscular atrophy

#### B. Risi ^1^; N. Ait Allali^2^; E. Franchi^2^; A. Rizzardi^3^; L. Ferullo^2^; F. Caria^2^; R. Bestetti^4^; L. Poli^5^; V. Bozzoni^5^; J. Galli^6^; A. Padovani^7^; A. Pilotto^8^; M. Filosto^9^

##### ^*1*^ *NeMO‐Brescia Clinical Center for Neuromuscular Diseases, Brescia, Italy; Department of Molecular and Translational Medicine, University of Brescia, Brescia, Italy;* ^*2*^ *NeMO‐Brescia Clinical Center for Neuromuscular Diseases, Brescia, Italy;* ^*3*^ *Department of Clinical and Experimental Sciences, University of Brescia, Brescia, Italy; Laboratory of Digital Neurology and Biosensors, University of Brescia, Brescia, Italy;* ^*4*^ *NeMO‐Brescia Clinical Center for Neuromuscular Diseases, Brescia, Italy; Department of Clinical and Experimental Sciences, University of Brescia, Brescia, Italy;* ^*5*^ *Unit of Neurology, ERN EURO‐NMD Center ASST Spedali Civili, Brescia, Italy;* ^*6*^ *Unit of Child and Adolescent Neuropsychiatry, ASST Spedali Civili, Brescia, Italy; Department of Clinical and Experimental Sciences, University of Brescia, Brescia, Italy;* ^*7*^ *Unit of Neurology, ERN EURO‐NMD Center ASST Spedali Civili, Brescia, Italy; Department of Clinical and Experimental Sciences, University of Brescia, Brescia, Italy;* ^*8*^ *Unit of Neurology, ERN EURO‐NMD Center ASST Spedali Civili, Brescia, Italy; Laboratory of Digital Neurology and Biosensors, University of Brescia, Brescia, Italy; Department of Clinical and Experimental Sciences, University of Brescia, Brescia, Italy;* ^*9*^ *NeMO‐Brescia Clinical Center for Neuromuscular Diseases, Brescia, Italy; Unit of Neurology, ERN EURO‐NMD Center ASST Spedali Civili, Brescia, Italy; Department of Clinical and Experimental Sciences, University of Brescia, Brescia, Italy*

## Movement disorders

## EPV‐0677

### A case of neurodevelopmental disorder mitochondrial with abnormal movements and lactic acidosis with or without seizures (nemmlas)

#### H. Kaleagasi ^1^; U. Topbas^2^; M. Komur^3^

##### ^*1*^ *Department of Neurology, Mersin University Medical Faculty, Türkiye;* ^*2*^ *Department of Neurology Toros State Hospital Mersin, Türkiye;* ^*3*^ *Department of Child Neurology, Mersin University Medical Faculty, Türkiye*

## EPV‐0678

### A case of PLA2G6‐associated neurodegeneration

#### A. Gamboa Berastegui; M. Cortes Rubiales; A. Rodriguez Valer; S. Cajaraville Vicente; C. Sifre Peña; A. Santesteban Nedderhoff; A. Rodriguez Lopez; A. Barquin Toca; A. Pinedo Brochado

##### Neurology, H.Galdakao‐Usansolo, Bilbao, Spain

## EPV‐0679

### A novel homozygous PLA2G6 mutation in a patient with young‐adult‐onset dystonia‐parkinsonism

#### G. Genç ^1^; N. Smolina^2^; M. Uğureli^1^; E. Pirdoğan Aydın^3^; D. Selçuk Demirelli^1^; A. Başak^2^

##### ^*1*^ *University of Health Sciences, Department of Neurology, Şişli Etfal Training and Research Hospital, İstanbul, Türkiye;* ^*2*^ *Koç University, School of Medicine, Molecular Biology and Genetics‐ KUTTAM Suna and Inan Kıraç Foundation, Neurodegeneration Research Laboratory, İstanbul, Türkiye;* ^*3*^ *University of Health Sciences, Department of Psychiatry, Şişli Etfal Training and Research Hospital, İstanbul, Türkiye*

## EPV‐0680

### A pilot, cross‐sectional study on sleep quality and anxiety between Parkinson's disease (PD) patients and their caregivers

#### C. Lee; E. Tan; P. Manharlal; G. Chia; X. Choi

##### Department of Neurology, National Neuroscience Institute, Singapore General Hospital, Singapore

## EPV‐0681

### A preventable clinical cascade: Levodopa‐induced polyneuropathy as a contributor to traumatic cervical myelopathy in Parkinson's disease

#### M. Liksunova ^1^; A. Afanasieva^2^; M. Bodnar^2^; T. Slobodin^2^

##### ^*1*^ *Department of Neurology, Bogomolets National Medical University, Kyiv, Ukraine;* ^*2*^ *Department of Neurology, LLC “Dobrobut‐Clinic”, Kyiv, Ukraine*

## EPV‐0682

### Accelerating real‐world evidence generation in Parkinson's disease using the AccessPD registry: Case studies from digital sub‐studies in the UK and US

#### M. Ippolito ^1^; G. Harrison‐Jones^1^; L. Akindele^1^; E. Shelton^2^; A. Noyce^3^

##### ^*1*^ *Bial Pharma UK Ltd, Windsor, UK;* ^*2*^ *Cohort Science, London, UK;* ^*3*^ *Centre for Preventive Neurology, Wolfson Institute of Population Health, Faculty of Medicine and Dentistry, Queen Mary University of London, London, UK*

## EPV‐0683

### Access to essential medicines for Parkinson's disease in armenia: A health system gap analysis

#### K. Petrosyan; A. Grigoryan; N. Hambardzumyan

##### Department of General and Vascular Neurology, Saint Gregory the Illuminator Medical Center, Yerevan, Armenia

## EPV‐0684

### Access to Parkinson's disease treatments in the Middle East: A multi‐national survey of availability, affordability, and utilization patterns

#### M. Salari^1^; S. Soleimani ^2^; S. Mittal^3^; Z. Aldaajani^4^; H. Khalil^5^; M. Etemadifar^6^; M. Gultekin^7^; F. Alfwaress^8^; M. Iqbal Arain^9^

##### ^*1*^ *Men's Health and Reproductive Health Research Center, Shahid Beheshti University of Medical Sciences, Tehran, Iran;* ^*2*^ *Neurology Department, School of Medicine, Shahid Beheshti University of Medical Sciences, Tehran, Iran;* ^*3*^ *Cleveland Clinic, Abu Dhabi, United Arab Emirates;* ^*4*^ *Clinical Neurosciences Department, King Fahad Military Medical Complex, Dhahran, Saudi Arabia;* ^*5*^ *Department of Rehabilitation Sciences, College of Health Sciences, Qatar University, Doha, Qatar;* ^*6*^ *Faculty of Medicine, Isfahan University of Medical Sciences, Isfahan, Iran;* ^*7*^ *Department of Neurology, Erciyes University, Faculty of Medicine 38039, Kayseri, Türkiye;* ^*8*^ *Department of Rehabilitation Sciences, Jordan University of Science and Technology, Irbid, Jordan;* ^*9*^ *College of Health and Human Services, University of North Carolina, Wilmington, NC, USA*

## EPV‐0685

### Adaptive deep brain stimulation in Parkinson's disease: Clinical outcomes, biomarkers and translational challenges—A focused literature review

#### C. Ballester Martinez

##### Unit of Functional Neurosurgery, National Hospital for Neurology and Neurosurgery, UCLH, London, UK

## EPV‐0686

### Adult‐onset idiopathic tic disorder: a rare clinical entity

#### P. Gómez Ramírez; A. Sánchez Gómez; M. El Harmochi Daoud; A. Herrera Ortega; M. Carrillo Carrillo; V. Ros Escolar; M. Corrales Arroyo; J. Vaamonde Gamo

##### Department of Neurology, Ciudad Real General University Hospital, Ciudad Real, Spain

## EPV‐0687

### Adult‐onset vanishing vhite watter disease presenting with dopa‐responsive dystonia

#### L. Bouafia; A. Rekik; A. Mili; K. Jemai; H. Slimene; E. Jarrar; A. Hssine; S. Naija; S. Benamor

##### Neurology Departement, Sahloul University Hospital, Sousse, Tunisia

## EPV‐0688

### Advocacy, care coordination, education & support services: ACCESS HD novel national Huntington's disease community outreach care pathway in Ireland

#### D. Mc Intyre ^1^; O. Russell^3^; O. Hardiman^2^; N. Pender^4^

##### ^*1*^ *St. James's Hospital, Teaching Hospital School of Medicine, Trinity College Dublin, James's St, Saint James, Dublin 8, Ireland;* ^*2*^ *Trinity College Dublin, Trinity Biomedical Sciences Institute, Pearse Street, Dublin 2, Ireland;* ^*3*^ *National Neuroscience Centre, Beaumont Hospital, Beaumont Road, Dublin 9, Ireland;* ^*4*^ *Royal College of Surgeons in Ireland (RCSI), Department of Health Psychology, 123 St Stephen's Green, Dublin 2, Ireland*

## EPV‐0689

### Alpha‐synuclein in neuron‐derived extracellular vesicles in Parkinson's disease

#### B. Elitas Ozmutlu ^1^; I. Koker^2^; M. Abdulsalam^3^; C. Beyret^4^; Z. Taş^5^; M. Çağlayan^4^; B. Derkus^6^; R. Yilmaz^1^

##### ^*1*^ *Ankara University School of Medicine, Department of Neurology, Ankara, Türkiye;* ^*2*^ *Ankara University School of Medicine, Ankara, Türkiye;* ^*3*^ *Ankara University, Institute of Health Sciences, Ankara, Türkiye;* ^*4*^ *Ankara University, Faculty of Pharmacy, Department of Analytical Chemistry, Ankara, Türkiye;* ^*5*^ *Hacettepe University, Faculty of Science, Department of Biology, Ankara, Türkiye;* ^*6*^ *Department of Chemistry, Division of Biochemistry, Faculty of Science, Ankara University, Ankara, Türkiye*

## EPV‐0690

### Amantadine as first‐line treatment in early Huntington's disease: A one‐year observational study

#### L. Cori; G. Palermo; M. Tedeschi; E. Benevento; E. Unti; R. Ceravolo

##### Center for Neurodegenerative Diseases–Parkinson's Disease and Movement Disorders, Unit of Neurology, Department of Clinical and Experimental Medicine, University of Pisa, Pisa, Italy

## EPV‐0691

### Amyloid status as a moderator of the association between physical activity and progression of Parkinson's disease

#### M. Lee ^1^; K. Pak^1^; M. Kim^2^; J. Lee^3^

##### ^*1*^ *Pusan National University Hospital, Yangsan, Republic of Korea;* ^*2*^ *Gyeongsang National University Hospital, Jinju, Republic of Korea;* ^*3*^ *Pusan National University Yangsan Hospital, Yangsan, Republic of Korea*

## EPV‐0692

### Archimedes' spiral‐based analysis using machine learning model for tremor's classification

#### R. Guizani; A. Rekik; M. Abid; A. Mili; S. Laatoui; I. Rekik; S. Ben Amor

##### Department of Neurology, Sahloul University Hospital, Sousse, Tunisia

## EPV‐0693

### Assessment of psychosis in Parkinson disease (PD)

#### V. Tsipropoulou; M. Liakou; I. Dagklis; I. Dagklis; Z. Katsarou; D. Kazis; S. Bostantzopoulou

##### 3rd Department of Neurology, Aristotle University, George Papanikolaou Genreal Hospital, Thessaloniki, Greece

## EPV‐0694

### Association of Restless Leg Syndrome (RLS) with Parkinson's disease, migraine, multiple sclerosis: A systematic review and meta‐analysis

#### Y. Hawas ^1^; E. Moubarak^2^; N. shaban^3^; J. Khasawneh^4^; S. El Khatib^5^; A. abdelbaset^6^; N. Al‐Hawari^7^; A. Dewair^8^; A. Samir^9^; M. Al Diab Al Azzawi^10^

##### ^*1*^ *Faculty of Medicine, Tanta University, Tanta, Egypt;* ^*2*^ *Faculty of Medicine, Cairo University, Cairo, Egypt;* ^*3*^ *Faculty of Medicine for Girls, Al‐Azhar University, Cairo, Egypt;* ^*4*^ *Faculty of Medicine, Yarmouk University, Irbid, Jordan;* ^*5*^ *UCHealth Parkview Medical Center;* ^*6*^ *Al‐azher Faculty of Medicine, New Damietta University;* ^*7*^ *Jordan University of Science and Technology, Irbid, Jordan;* ^*8*^ *Faculty of Medicine, Alexandria University, Alexandria, Egypt;* ^*9*^ *Faculty of Medicine, Al‐Azhar University, Cario, Egypt;* ^*10*^ *Faculty of Medicine, The National Ribat University, Khartoum, Sudan*

## EPV‐0695

### Association of sleep hygiene practice with disturbed sleep in early‐stage Parkinson's Disease

#### C. Lee ^1^; J. Kim^2^; D. Kim^2^; H. Kim^2^; J. Yun^2^

##### ^*1*^ *Department of Neurology, Ewha Womans University Mokdong Hospital, Ewha Womans University College of Medicine, Seoul, Republic of Korea.;* ^*2*^ *Department of Neurology, Ewha Womans University Seoul Hospital, Ewha Womans University College of Medicine, Seoul, Korea*

## EPV‐0696

### Atherogenic lipid indices and disease severity in restless legs syndrome: A cross‐sectional case‐control study

#### A. Aksoy Gündoğdu^1^; D. Kotan ^2^; E. Çiçekli^2^

##### ^*1*^ *Department of Neurology, Tekirdağ Namık Kemal University Faculty of Medicine, Tekirdağ, Türkiye;* ^*2*^ *Department of Neurology, Sakarya University Faculty of Medicine, Sakarya, Türkiye*

## EPV‐0697

### Atypical tremor associated with genes involved in metal metabolism and neurovascular signaling

#### C. Toilos; K. Ntoskas; I. Balatsouka; O. Sideri; V. Deligianni; A. Tsagkaropoulos; E. Kouremenos

##### Department of Neurology, 251 Air Force General Hospital, Athens, Greece

## EPV‐0698

### Baseline clinical characteristics of Parkinson's disease at first presentation to a National Tertiary Referral Center in the Republic of Moldova

#### M. Cebuc ^1^; E. Costru‐Tașnic^2^; I. Teacă^1^; A. Arabadji^1^; L. Rotaru^1^; O. Gavriliuc^1^

##### ^*1*^ *Diomid Gherman Institute of Neurology and Neurosurgery, Chișinău, Republic of Moldova;* ^*2*^ *Nicolae Testemițanu State University of Medicine and Pharmacy, Chișinău, Republic of Moldova*

## EPV‐0699

### Beyond motor control: Neuropsychiatric benefits of botulinum toxin type A in Parkinson's disease: A systematic review and meta‐analysis

#### A. Harb ^1^; A. Mansour^1^; S. Qadri^2^; A. Hamza^3^

##### ^*1*^ *Faculty of Medicine, Al‐Azhar University, Cairo, Egypt;* ^*2*^ *Dow University of Health Sciences, Karachi, Pakistan;* ^*3*^ *University of Pittsburgh, Neuroscience Department Pittsburgh, Pennsylvania*

## EPV‐0700

### Beyond motor symptoms in dystonia: Non‐motor symptoms and retinal OCT features

#### S. Yörük Öner; Y. Dede; S. Emekli; E. Şahin; Ş. Akça Kalem; S. Kalfa; B. Bilgiç; H. Hanağası; B. Samancı

##### Behavioral Neurology and Movement Disorders Unit, Department of Neurology, Istanbul Faculty of Medicine, Istanbul University, Istanbul, Türkiye

## EPV‐0701

### Beyond motor symptoms in Multiple System Atrophy (MSA): Our experience in northern tenerife

#### C. Hernández Javier; V. Pallarés Santos; M. Hernández García; A. Bartolomé Yumar; J. Rojo Aladro

##### Hospital Universitario de Canarias, Spain

## EPV‐0702

### Caregiver burden in people with Parkinson's disease treated with deep brain stimulation: How does it evolve over time?

#### I. Margarido ^1^; C. Pacheco^2^; J. Ramos^3^; C. Chamadoira^4^; M. Rito^4^; A. Oliveira^1^; J. Massano^1^; M. Rosas^1^; M. Pinto^4^; C. Soares^1^

##### ^*1*^ *Department of Neurology, São João University Hospital, Porto, Portugal;* ^*2*^ *Faculty of Medicine of the University of Porto, Porto, Portugal;* ^*3*^ *Diagnostic Neuroradiology Unit, Imaging Department, Gaia‐Espinho Local Health Unit, Vila Nova de Gaia, Portugal;* ^*4*^ *Department of Neurosurgery, São João University Hospital, Porto, Portugal*

## EPV‐0703

### CARNS1 regulates neurohomeostasis in Parkinson's disease via STUB1‐mediated ubiquitination and carnosine axis

#### H. Xu; Q. Ye

##### Department of Neurology, Fujian Medical University Union Hospital, Fuzhou, China

## EPV‐0704

### Changes in tremor, anxiety and depression after MR‐guided focused ultrasound for essential tremor: A prospective study and exploration of predictors

#### A. Shiramba ^1^; S. Lane^3^; L. Andrews^2^; V. Patel^2^; H. Shepherd^1^; D. Damodaran^2^; R. Srinivasaiah^2^; M. Radon^2^; J. Panicker^2^; J. Osman‐Farah^2^; A. Macerollo^4^

##### ^*1*^ *School of Medicine, University of Liverpool, Liverpool, UK;* ^*2*^ *The Walton Centre NHS Foundation Trust for Neurology and Neurosurgery, Liverpool, UK;* ^*3*^ *Institute of Data Health Sciences, University of Liverpool, UK;* ^*4*^ *Institute of Systems, Molecular and Integrative Biology, The University of Liverpool, UK*

## EPV‐0705

### Characteristics of dystonia in Niemann‐Pick disease type C

#### A. Nikolic; V. Marković; M. Ječmenica‐Lukić; A. Tomić‐Pešić; N. Kresojević; N. Dragašević‐Mišković; I. Petrović

##### *Neurology Clinic, University Clinical Center of Serbia,* Belgrade; Serbia

## EPV‐0706

### Characterizing the bradykinesia complex in GBA‐associated Parkinson's disease: A series of three cases

#### M. De Riggi ^1^; D. Birreci^1^; S. Aloisio^1^; S. Grandolfo^1^; L. Angelini^1^; S. Cirinei^1^; G. Paparella^3^; A. Cannavacciuolo^2^; M. Bologna^1^

##### ^*1*^ *Department of Human Neurosciences, Sapienza, University of Rome, Rome, Italy;* ^*2*^ *IRCCS Neuromed, Pozzilli, IS, Italy;* ^*3*^ *3Neurophysiopathology Unit, Department of Translational Biomedicine and Neuroscience (DiBraiN), University of Bari Aldo Moro, Bari, Italy*

## EPV‐0707

### Clinical and genetic characteristics of ANO3‐associated movement disorders in a large Chinese cohort

#### J. Huang ^1^; Y. Li^2^; Z. Wan^2^; L. Li^3^; L. Jin^1^; X. Wan^2^

##### ^*1*^ *Department of Neurology and Neurological Rehabilitation, Yangzhi Rehabilitation Hospital (Shanghai Sunshine Rehabilitation Center), School of Medicine, Tongji University, Shanghai, China;* ^*2*^ *Department of Neurology, Peking Union Medical College Hospital, Chinese Academy of Medical Sciences, Peking Union Medical College, Beijing, China;* ^*3*^ *Neurotoxin Research Center of Key Laboratory of Spine and Spinal Cord Injury Repair and Regeneration of Ministry of Education, Department of Neurology, Tongji Hospital, School of Medicine, Tongji University, Shanghai, China*

## EPV‐0708

### Clinical characterization of the response to Fampridine in SCA27B: Challenges and opportunities

#### I. Margarido; B. Carvalho; R. Soares‐dos‐Reis; C. Soares

##### Department of Neurology, São João University Hospital, Porto, Portugal

## EPV‐0709

### Clinical drug‐drug interaction profile of BIA 28‐6156: Results from three phase I studies

#### T. Fonseca ^1^; A. Ronaghinia^1^; J. Reis^1^; D. Rodrigues^1^; A. Neves^2^; R. Costa^2^; J. Holenz^2^

##### ^*1*^ *BIAL – R&D Investments, S.A., Coronado, Portugal;* ^*2*^ *BIAL – Portela & Co. S.A., Coronado, Portugal*

## EPV‐0710

### Clinical effectiveness and safety of unilateral and staged‐bilateral MR guided focused ultrasound for essential tremor: Systematic literature reviews

#### N. Kaempf ^1^; A. Sanchez Fraga^1^; L. Becla^1^; K. Gant^2^; A. Grinspan^2^; C. Ferrer^1^

##### ^*1*^ *Insightec Europe GmbH, Munich, Germany;* ^*2*^ *Insightec. Inc, Miami, USA*

## EPV‐0711

### Clinical outcomes after dual‐target MR‐guided focused ultrasound in Parkinson's disease: A single‐center experience

#### J. Chen ^1^; M. Lu^2^; C. Chen^3^; C. Tsai^4^

##### ^*1*^ *Neuroscience and Brain Disease Center, CMUH, Taiwan;* ^*2*^ *Neuroscience Laboratory, Department of Neurology, China Medical University Hospital, Taiwan;* ^*3*^ *Department of Radiology, CMUH, Taiwan;* ^*4*^ *School of Medicine, College of Medicine, CMUH, Taiwan*

## EPV‐0712

### Clinical phenotype and early axial involvement in very late‐onset Parkinson's disease: A real‐world cohort study

#### E. Sfikas; A. Simitsi; N. Papagiannakis; I. Alefanti; E. Angelopoulou; A. Alexandratou; I. Alexandratou; L. Stefanis; C. Koros

##### First Department of Neurology, Eginition Hospital, National and Kapodistrian University of Athens, Athens, Greece

## EPV‐0713

### Clinical profile and burden of non‐motor symptoms in Parkinson's disease: A cross‐sectional study

#### M. Cebuc ^1^; O. Grosu^1^; S. Odobescu^1^; I. Moldovanu^1^; V. Vovc^2^; S. Lozovanu^2^; S. Groppa^3^; L. Rotaru^1^

##### ^*1*^ *Functional Neurology Research Unit, Diomid Gherman Institute of Neurology and Neurosurgery, Chișinău, Republic of Moldova;* ^*2*^ *Department of Human Physiology and Biophysics, Nicolae Testemițanu State University of Medicine and Pharmacy, Chișinău, Republic of Moldova;* ^*3*^ *Department of Neurology No. 2, Nicolae Testemițanu State University of Medicine and Pharmacy, Chișinău, Republic of Moldova*

## EPV‐0714

### Clinical, radiological, and genetic characterization of neurodegeneration with Brain Iron Accumulation (NBIA): A case series

#### C. Villar Rodriguez ^1^; D. Villagrán Sancho^1^; I. Lopera Rodríguez^1^; A. Luque Ambrosiani^2^; A. Adarmes Gómez^2^; D. Macías García^2^; F. Carrillo García^2^; L. Muñoz Delgado^2^; E. Ojeda Lepe^2^; S. Jesús Maestre^2^; P. Mir Rivera^2^

##### ^*1*^ *Neurology Department. Virgen del Rocio University Hospital;* ^*2*^ *Movement Disorders Unit, Department of Neurology and Clinical Neurophysiology, Institute of Biomedicine of Seville, Virgen del Rocío University Hospital/CSIC/University of Seville, Seville, Spain.*

## EPV‐0715

### Cognitive decline and motor severity across progressive supranuclear palsy phenotypes

#### A. Hajji ^1^; Y. Abida^2^; A. Mansouri^2^; A. Souissi^2^; A. Atrous^2^; A. Gargouri^2^; I. Kacem^2^; R. Gouider^2^

##### ^*1*^ *Departement of Neurology, LR18SP03, Clinical Investigation Center “Neuroscience and Mental Health”, Razi Hospital, Tunis. Faculty of Medecine of Tunis, University Tunis El Manar, Tunis, Tunisia;* ^*2*^ *Departement of Neurology, LR18SP03, Clinical Investigation Center “Neuroscience and Mental Health”, Razi Hospital, Tunis. Faculty of Medecine of Tunis, University Tunis El Manar, Tunis, Tunisia*

## EPV‐0716

### Cognitive impairment in people with idiopathic dystonia in Bulgaria – A prospective cohort study

#### S. Shtereva ^2^; N. Topalov^2^; S. Shopova^1^; A. Dimitrova^1^; V. Todorov^2^; A. Atanasov^1^; N. Kalaydzhiev^1^; T. Evdenova^1^; D. Gadzhalova^2^; A. Gotsov^1^; I. Milanov^2^; D. Bogdanova^2^

##### ^*1*^ *University Hospital for Active Treatment in Neurology and Psychiatry Sv. Naum, Sofia, Bulgaria;* ^*2*^ *Medical University, Sofia, Bulgaria*

## EPV‐0717

### Cognitive profile of LRRK2‐G2019S Parkinson's disease in a Tunisian cohort

#### A. Agrebaoui; R. Zouari; A. Mousli; B. Marzouki; F. Nabli; Z. Saied; A. Rachdi; D. Ben Mohamed; S. Ben Sassi

##### Department of Neurology, National Institute of Neurology Mongi Ben Hmida, Tunis, Tunisia

## EPV‐0718

### Complications of levodopa–carbidopa intestinal gel therapy: Polyneuropathy, cacosmia, and peritonitis

#### E. Kose

##### *Neurology*, *Bucak Devlet Hastanesi, Burdur, Türkiye*

## EPV‐0719

### Continuous Subcutaneous Levodopa Infusion in Advanced Parkinson's Disease patients with Subthalamic Nucleus Deep Brain Stimulation

#### A. Bernardini ^1^; D. De Monte^2^; G. Ermanis^2^; E. Belgrado^3^; F. Janes^1^; G. Pellitteri^1^; M. Scanni^2^; E. Betto^4^; M. Valente^2^; C. Lettieri^1^

##### ^*1*^ *Clinical Neurology Unit, Department of Head‐Neck and Neurosciences, Azienda Sanitaria Universitaria Friuli Centrale (ASU FC), Udine, Italy;* ^*2*^ *Department of Medicine, University of Udine, Udine, Italy;* ^*3*^ *Neurology Unit, Department of Head‐Neck and Neurosciences, Azienda Sanitaria Universitaria Friuli Centrale (ASU FC), Udine, Italy;* ^*4*^ *Institute of Medical Genetics, Azienda Sanitaria Universitaria Friuli Centrale (ASU FC), Udine, Italy*

## EPV‐0720

### Correlation between tnf‐α levels and psychoemotional disorders in Parkinson's disease

#### S. Jumanazarova; R. Matmurodov

##### Tashkent State Medical University, Tashkent, Uzbekistan

## EPV‐0721

### Could abdominal hernia be an early clue for Parkinson's disease?

#### G. Yildiz; B. Elitas Ozmutlu; R. Yilmaz; M. Akbostancı

##### Department of Neurology, Ankara University Faculty of Medicine, Ankara, Türkiye

## EPV‐0722

### Deep brain stimulation for medically refractory tremor in multiple sclerosis: A single case and review of the literature

#### A. Giordano; R. Matrullo; L. Buttarelli; R. D'Auria; A. Tessitore

##### Department of Advanced Medical and Surgical Sciences, University of Campania “Luigi Vanvitelli”, Napoli, Italy

## EPV‐0723

### Depression and anxiety in Parkinson's disease: A comparison according to motor phenotypes

#### B. Kuz ^1^; M. Yaşa^2^; R. Sonkaya^3^; B. Birbir^2^

##### ^*1*^ *Faculty of Health Sciences, Ordu University, Ordu, Türkiye;* ^*2*^ *Gulhane Faculty of Physiotherapy and Rehabilitation, University of Health Sciences, Türkiye;* ^*3*^ *Neurology Department, Gulhane School of Medicine, University of Health Sciences, Türkiye*

## EPV‐0724

### Depression and quality of life in patients with Parkinson's disease

#### S. Jumanazarova; B. Amonov; R. Matmurodov

##### Tashkent State Medical University, Tashkent, Uzbekistan

## EPV‐0725

### Development of a standardized glossary to improve multidisciplinary and patient‐centered communication in Progressive Supranuclear Palsy (PSP)

#### B. Ghosh^1^; Á. Sánchez‐Ferro^2^; C. Varona ^3^; N. Noel^3^; A. Marcos^3^

##### ^*1*^ *University Hospital Southampton NHS Foundation Trust, Southampton, UK;* ^*2*^ *Hospital Universitario 12 de Octubre, Madrid, Spain;* ^*3*^ *Medical Affairs, Ferrer, Barcelona, Spain*

## EPV‐0726

### Diagnostic revision in Parkinson's disease within five years after diagnosis

#### R. Nasalyk ^2^; S. Shkrobot^1^; K. Duve^1^

##### ^*1*^ *Neurology Department, Horbachevsky Ternopil National Medical University, Ternopil, Ukraine;* ^*2*^ *Ternopil Regional Clinical Psychoneurological Hospital, Ternopil, Ukraine*

## EPV‐0727

### Diagnostic value of MRI Nigrosome‐1 visualization vs DaTSCAN SPECT in Parkinson's disease

#### A. Bystrzyński ^1^; A. Wypych^2^; Z. Serafin^3^; B. Brockhuis^4^; R. Piekarski^1^; W. Sołtan^5^; J. Sławek^1^; M. Burzyńska‐Makuch^2^

##### ^*1*^ *Department of Neurology, Neurodegenerative Diseases and Neuroimmunology, Faculty of Health Sciences, Medical University of Gdańsk, Poland, Neurology & Stroke Department, St. Adalbert Hospital, Copernicus PL Ltd., Gdańsk, Poland;* ^*2*^ *Centre for Modern Interdisciplinary Technologies, Nicolaus Copernicus University in Toruń;* ^*3*^ *Faculty of Medical Sciences, Bydgoszcz University of Science and Technology, Bydgoszcz, Poland;* ^*4*^ *Department of Nuclear Medicine, Medical University of Gdańsk;* ^*5*^ *Neurology&Stroke Department, St. Adalbert Hospital, Copernicus PL Ltd., Gdańsk, Poland*

## EPV‐0728

### Differentiating functional dystonia from organic dystonia – A tertiary care centre experience

#### A. Cherian; D. Kalikavil Puthanveedu

##### Department of Neurology, Sree Chitra Tirunal Institute for Medical Sciences and Technology, Thiruvananthapuram, India

## EPV‐0729

### Digital biomarkers for detecting wearing‐off in Parkinson's disease: A pilot study of the remepy mobile platform

#### A. Amedi ^1^; R. Vakil^2^; R. Rimon^2^; T. Tamir^2^

##### ^*1*^ *The Baruch Ivcher Institute for Brain, Cognition, and Technology, Reichman University, Herzliya, Israel;* ^*2*^ *Remepy Health Ltd., Ramat Gan, Israel*

## EPV‐0730

### Digital narrative medicine in Parkinson's disease: Patient and caregiver stories to inform clinical care

#### P. Pacilio; C. Trisoglio; L. De Carolis; S. Galli; D. Rinaldi

##### Department of Neuroscience, Mental Health and Sensory Organs (NESMOS), Sapienza University of Rome, Rome, Italy

## EPV‐0731

### Domain‐specific associations between pain and non‐motor symptoms in Parkinson's disease

#### S. Landolfo

##### Neurology Unit, San Giacomo Hospital, Monopoli, Italy

## EPV‐0732

### Dopamine dysregulation syndrome induced by dopaminergic therapy in Parkinson's disease: A case report

#### K. Alidreesi ^1^; A. Attar^2^; Y. Aladdin^2^; A. Alsebyani^2^

##### ^*1*^ *College of Medicine, King Saud bin Abdulaziz University for Health Sciences, Jeddah, Kingdom of Saudi Arabia;* ^*2*^ *Neurology Department, King Abdulaziz Medical City, Jeddah, Kingdom of Saudi Arabia*

## EPV‐0733

### Dropped head syndrome after radiotherapy for Hodgkin's lymphoma: Cervical muscles as a potential target for botulinum toxin

#### V. Stride‐González ^1^; P. Martín‐Jiménez^2^; I. Paredes‐Sansinenea;^3^; L. Jiménez‐Roldán^3^; Á. Sánchez‐Ferro^4^; A. Méndez‐Guerrero;^4^; J. Ramírez Sánchez‐Ajofrín;^4^; P. Rábano‐Suárez^4^

##### ^*1*^ *Department of Neurology, Hospital Universitario Doce de Octubre, Madrid, Spain.;* ^*2*^ *Neuromuscular Disorders Unit, Department of Neurology, Hospital Universitario Doce de Octubre, Madrid, Spain.;* ^*3*^ *Department of Neurosurgery, Hospital Universitario Doce de Octubre, Madrid, Spain;* ^*4*^ *Movement Disorders Unit, Department of Neurology, Hospital Universitario Doce de Octubre, Madrid, Spain*

## EPV‐0734

### Dysregulated EEG theta activity suggests impaired synaptic build‐up in levodopa‐induced dyskinesia in Parkinson's disease

#### L. Fiorillo^1^; G. Lombardi^1^; I. Bertaina ^1^; E. Corno^1^; A. Castelnovo^2^; A. Kaelin‐Lang^1^; S. Galati^1^

##### ^*1*^ *Parkinson Disease and Movement Disorder Center, Neurocenter of Southern Switzerland, Ente Ospedaliero Cantonale;* ^*2*^ *Sleep Medicine Unit, Neurocenter of Italian Switzerland, Ente Ospedaliero Cantonale*

## EPV‐0735

### Early real‐world experience with foslevodopa/foscarbidopa infusion in a UK neurology centre

#### K. Varmpompiti; V. Dostal

##### Department of Neurology, Norfolk and Norwich University Hospital, Norwich, UK

## EPV‐0736

### Early‐onset mixed phenotype in a patient with Huntington's Disease and SCA2: A clinico‐pathological case description

#### S. Costa ^1^; F. Almeida^2^; J. Oliveira^3^; A. Sardoeira^4^; M. Pinto^5^; R. Taipa^5^; J. Damásio^1^

##### ^*1*^ *Serviço de Neurologia, Departamento de Neurociências, Centro Hospitalar Universitário do Porto, Porto, Portugal;* ^*2*^ *Serviço de Neurorradiologia, Departamento de Neurociências, Centro Hospitalar Universitário do Porto, Porto, Portugal;* ^*3*^ *CGPP‐IBMC, i3S – Instituto de Investigação e Inovação em Saúde, Universidade do Porto, Porto, Portugal;* ^*4*^ *Serviço de Neurologia, Unidade Local de Saúde Gaia e Espinho, Porto, Portugal;* ^*5*^ *Banco Português de Cérebros, Serviço de Neuropatologia, Departamento de Neurociências, Centro Hospitalar Universitário do Porto, Porto, Portugal*

## EPV‐0737

### Effect of continuous subcutaneous infusion of Foslevodopa/Foscarbidopa on motor fluctuations, dyskinesia, and sleep; subjective vs. PKG watch scores

#### M. Bradley ^1^; C. O'Keeffe^1^; J. Inocentes^1^; E. Donlon^1^; F. Ruggieri^1^; R. Reilly^2^; R. Walsh^1^; T. Lynch^1^; C. Fearon^1^

##### ^*1*^ *Dublin Neurological Institute, Mater Misericordiae University Hospital, Dublin, Ireland;* ^*2*^ *Trinity Centre for Biomedical Engineering, Trinity College Dublin, Dublin, Ireland*

## EPV‐0738

### Effects of Transcranial Pulse Stimulation (TPS) intervention in patients with Parkinson's disease

#### P. Manganotti ^1^; M. Liccari^1^; T. Lombardo^1^; V. Cenacchi^1^; J. Della Toffola^1^; M. Ajcevic^2^; P. Busan^3^

##### ^*1*^ *Clinical Neurology, Department of Medical Sciences University of Trieste Italy;* ^*2*^ *Department of Engineering and Architecture, University of Trieste, Italy;* ^*3*^ *Department of Life Sciences, University of Trieste, Italy*

## EPV‐0739

### Efficacy and safety of cannabinoid‐based therapies in Parkinson's disease: A systematic review and meta‐analysis of randomized controlled trials

#### A. Mansour ^1^; A. Harb^1^; A. Amin^1^; A. Hamza^2^; K. Hamzah^3^; E. Thomas^4^

##### ^*1*^ *Faculty of Medicine, Al‐Azhar University, Cairo, Egypt;* ^*2*^ *University of Pittsburgh, Neuroscience Department Pittsburgh, Pennsylvania;* ^*3*^ *ALkindy College of Medicine/University of Baghdad, Baghdad, Iraq;* ^*4*^ *Faculty of Pharmacy, Alexandria University, Egypt*

## EPV‐0740

### Efficacy and safety of opicapone in early Parkinson's disease: A prospective observational study

#### J. Aguilera Aguilera ^1^; B. Rodriguez Garcia^1^; D. Gómez de la Torre Morales^1^; J. Rodriguez Carrillo^1^; M. Luz Esteve^1^; D. Gonçalves Figueiredo^2^; L. Redondo Robles^1^; G. Carvalho Monteiro^1^

##### ^*1*^ *Salamanca University Hospital*, *Salamanca, Spain*; ^*2*^ *University of Salamanca*, *Salamanca, Spain*

## EPV‐0741

### Efficacy of Citicoline as an add‐on therapy in Parkinson's disease: The CITIPARK TRIAL trial

#### M. Marano ^1^; A. Pilotto^2^; G. Logroscino^3^; A. Stefani^4^; M. D'Amelio^5^; O. Difruscolo^6^; M. Barbagallo^7^; P. Barone^8^; F. Stocchi^9^; P. Palumbo^10^; A. Antonini^11^; L. Ferrannini^12^; M. Trabucchi^13^; A. Padovani^2^

##### ^*1*^ *Unit of Neurology, University Campus Bio‐Medico of Rome, Rome, Italy;* ^*2*^ *Neurology Unit, Department of Clinical and Experimental Sciences, University of Brescia, Brescia, Italy;* ^*3*^ *Center for Neurodegenerative Diseases and the Aging Brain, Pia Fondazione “Card. G. Panico”, Tricase, Italy;* ^*4*^ *Neurology Unit, Department of Systems Medicine, Tor Vergata University of Rome, Italy;* ^*5*^ *Dipartimento Di Biomedicina, Neuroscienze e Diagnostica Avanzata, Università di Palermo, Italy;* ^*6*^ *U.O.C di Neurologia e Stroke Unit, Ospedale Di Venere, Bari, Italy;* ^*7*^ *Geriatric Unit, Department of Internal Medicine and Geriatrics, University of Palermo, Italy;* ^*8*^ *Department of Medicine Surgery and Dentistry, Scuola Medica Salernitana, Neuroscience Section, University of Salerno, Italy;* ^*9*^ *University San Raffaele Roma and IRCCS San Raffaele, Italy;* ^*10*^ *Neurology Unit, N.O.P. – S. Stefano, U.S.L. Toscana Centro, Prato, Italy;* ^*11*^ *Department of Neurosciences, University of Padova, Italy;* ^*12*^ *University of Geneva;* ^*13*^ *University of Brescia, Italy*

## EPV‐0742

### Efficacy of Foslevodopa/Foscarbidopa infusion on gait disturbances and non‐motor symptoms in advanced Parkinson's disease

#### S. Abdelaziz ^2^; A. Leake^3^; M. Guerra Bernardo^3^; L. Kerogoi^3^; A. Shalash^2^; L. Ricciardi^1^; F. Morgante^1^

##### ^*1*^ *City St George's University of London, London, UK;* ^*2*^ *Faculty of Medicine, Ain Shams University– Cairo, Egypt;* ^*3*^ *St George's University Hospital, London, UK*

## EPV‐0743

### Enteric alpha‐synuclein pathology in early‐onset Parkinson's disease and an incidental case in spinocerebellar ataxia type 6

#### C. Shin ^1^; S. Kim^2^; S. Park^2^; J. Kim^1^; J. Lee^3^; S. Chung^4^; J. Kim^5^; T. Ahn^6^; K. Park^7^; J. Shin^8^; H. Lee^9^; S. Kong^9^; Y. Suh^10^; H. Kim^8^; H. Yang^9^; B. Jeon^11^

##### ^*1*^ *Department of Neurology, Seoul National University Bundang Hospital, Seoul National University College of Medicine, Gyeonggi‐do, Republic of Korea;* ^*2*^ *Department of Pathology, College of Medicine, Seoul National University, Seoul, Republic of Korea;* ^*3*^ *Department of Neurology, SMG‐SNU Boramae Medical Center, Seoul National University College of Medicine, Seoul, Republic of Korea;* ^*4*^ *Department of Neurology, Asan Medical Center, Seoul, Republic of Korea;* ^*5*^ *Department of Neurology, Dong‐A University Hospital, Busan, Republic of Korea;* ^*6*^ *Department of Neurology, Kyung Hee University Hospital, Seoul, Republic of Korea;* ^*7*^ *Department of Neurology, GangNeung Asan Hospital, Gangwon‐do, Republic of Korea;* ^*8*^ *Department of Neurology, MRC and Movement Disorder Center, Seoul National University Hospital, Parkinson Study Group, Seoul National University College of Medicine, Seoul, Republic of Korea;* ^*9*^ *Department of Surgery, Cancer Research Institute, Seoul National University College of Medicine, Seoul National University Hospital, Seoul, Republic of Korea;* ^*10*^ *Department of Surgery, Seoul National University Bundang Hospital, Seongnam‐si, Gyeonggi‐do, Republic of Korea;* ^*11*^ *Department of Neurology, Chung‐ang University Health Care System Hyundae Hospital, Namyangju‐si, Gyeonggi‐do, Republic of Korea*

## EPV‐0744

### Epidemiology and genetic landscape of hyperkinetic movement disorders in a Belgian tertiary care centre

#### D. Aktan; C. Mouraux; F. Depierreux

##### GIGA – CRC In Vivo Imaging Unit, Rare Movement Disorders (RMD) Research Group, University of Liege, Liege, Belgium

## EPV‐0745

### Factors of health‐related quality of life in elderly patients with Parkinson's disease

#### M. Miura ^1^; T. Ueno^1^; K. Hiyama^1^; R. Yanagida^1^; I. Kinoshita^1^; R. Haga^1^; A. Arai^1^; M. Tomiyama^2^

##### ^*1*^ *Department of Neurology, Aomori Prefectural Central Hospital, Aomori, Japan;* ^*2*^ *Department of Neurology, Institute of Brain Science, Hirosaki University Graduate School of Medicine, Aomori, Japan*

## EPV‐0746

### Failed and inconclusive clinical trials in Parkinson's disease: A systematic review

#### N. Szejko ^1^; A. Abusrair^2^; K. Smilowska^3^

##### ^*1*^ *Medical University of Warsaw, Sosnowiec, Poland;* ^*2*^ *Neurology Division, Department of Internal Medicine, Qatif Health Network, Sosnowiec, Poland;* ^*3*^ *Department of Neurology, Regional Specialist Hospital of St. Barbara in Sosnowiec, Sosnowiec, Poland*

## EPV‐0747

### Fatigue as a key independent predictor of quality of life in Parkinson's disease

#### S. Matoša ^1^; L. Ivanović Martić^2^; T. Gilman Kuric^1^; E. Strujić^1^; Z. Popović^1^; T. Mirošević Zubonja^1^; S. Tomić^1^

##### ^*1*^ *Department of Neurology, University Hospital Centre Osijek, Osijek, Croatia;* ^*2*^ *Faculty of Medicine, University of J. J. Strossmayer in Osijek, Osijek, Croatia*

## EPV‐0748

### Flow reduction and local tolerability after opicapone use with continuous foslevodopa/foscarbidopa infusion

#### G. Tabar Comellas; N. López Ariztegui; M. Morales Casado

##### Movement Disorders Unit, Hospital Universitario de Toledo, Spain

## EPV‐0749

### Foslevodopa‐foscarbidopa subcutaneous administration as bridging therapy in deep brain stimulation withdrawal syndrome

#### S. De Waele^1^; E. Hendrickx^1^; F. Dijkstra^1^; S. Tan^2^; S. Ligthart^3^; K. Dams^3^; P. Jorens^3^; M. De Praeter^2^; D. Crosiers ^1^

##### ^*1*^ *Department of Neurology, Antwerp University Hospital, Edegem, Belgium, and Faculty of Medicine and Health Sciences, University of Antwerp, Belgium;* ^*2*^ *Department of Neurosurgey, Antwerp University Hospital, Edegem, Belgium and Faculty of Medicine and Health Sciences, University of Antwerp, Belgium;* ^*3*^ *Department of Critical Care, Antwerp University Hospital, Edegem, Belgium, and Faculty of Medicine and Health Sciences, University of Antwerp, Belgium*

## EPV‐0750

### From waiting list to treatment: A “Traffic Light” triage algorithm for Foslevodopa/Foscarbidopa initiation in advanced Parkinson's disease

#### G. Lazzeri ^1^; F. Galbiati^1^; S. Cascino^1^; A. Caniglia^1^; S. Piazza^1^; G. Pezzoli^2^; I. Isaias^1^; S. Bonvegna^1^

##### ^*1*^ *Parkinson Institute of Milan, ASST G. Pini‐CTO, Milan, Italy;* ^*2*^ *Fondazione Pezzoli Per La Malattia Di Parkinson, Milan, Italy*

## EPV‐0751

### Functional movement disorders – A one‐year neuropsychiatric model to prevent chronicity

#### C. Hubsch ^1^; E. Trierweiler^2^

##### ^*1*^ *Service of Neurology, Department of Clinical Neuroscience, Lausanne University Hospital (CHUV) and University of Lausanne (UNIL), Lausanne, Switzerland;* ^*2*^ *Consultation‐Liaison Psychiatry Service, Department of Psychiatry, Lausanne University Hospital (CHUV), Lausanne, Switzerland*

## EPV‐0752

### Gait Instability in Parkinson's disease with motor fluctuations: Composite gait score analysis in clinical and real‐world settings

#### L. Colombo ^1^; A. Pilotto^1^; C. Zatti^3^; A. Rizzardi^2^; C. Hansen^4^; R. Romijnders^4^; W. Maetzler^4^; A. Padovani^1^

##### ^*1*^ *Department of Clinical and Experimental Sciences, Neurology Unit, University of Brescia, Italy;* ^*2*^ *Laboratory of digital Neurology and biosensors, University of Brescia, Italy;* ^*3*^ *Department of continuity of care and frailty, Neurology Unit, ASST Spedali Civili of Brescia, Brescia, Italy;* ^*4*^ *Department of Neurology, Christian‐Albrechts‐University of Kiel, Kiel, Germany*

## EPV‐0753

### Genotype–phenotype–biomarker integration in Parkinson's disease and parkinson‐plus syndromes: A prognostic matrix approach

#### D. Akramova; G. Rakhimbaeva; T. Bobomuratov; N. Vakhabova; U. Shamsieva; B. Ibodov

##### Tashkent State Medical University, Tashkent, Uzbekistan

## EPV‐0754

### Glioblastoma following cell therapy in a patient with Parkinson's disease: A cautionary tale

#### M. Martinez Seijas; A. Lopez Prado; A. Lagüela Alonso; V. Anciones Martin; M. Callejo Seguela; N. Marcos Fernandez; L. Pastor Martin; J. Mallada Abellan; F. Angoso Berrocal; B. Tijero Merino; J. Gomez Esteban; M. Ruiz Lopez

##### Neurology, Cruces Hospital, Barakaldo, Spain

## EPV‐0755

### Gut dysbiosis and inflammation in Parkinson's disease: An integrated microbiome–biomarker assessment

#### N. Mansurova

##### Tashkent State Medical University, Tashkent, Uzbekistan

## EPV‐0756

### Gut microbiota dysbiosis and alpha‐synuclein pathogenesis in Parkinson's disease: Exploring the gut‐brain axis and fecal microbiota transplantation

#### G. Frías Reyes ^1^; E. González Vilchis^2^; J. Morán Peraza^3^; L. Adalid Peralta^4^

##### ^*1*^ *División Académica Multidisciplinaria de Comalcalco DAMC‐UJAT, México;* ^*2*^ *Facultad de Medicina, Universidad Nacional Autónoma de México (UNAM), Coyoacán, Ciudad de México 04510, México;* ^*3*^ *Instituto Nacional de Ciencias Médicas y Nutrición “Salvador Zubirán” Vasco de Quiroga #15, Col. Belisario Domínguez Secc 16, Alcaldía Tlalpan, C.P. 14080, Ciudad de México, México;* ^*4*^ *Laboratorio de Reprogramación Celular, Instituto de Fisiología, Universidad Nacional Autónoma de México (UNAM), en el Instituto Nacional de Neurología y Neurocirugía, Insurgentes Sur 3877, La Fama, Ciudad de México, México*

## EPV‐0757

### “Hot Cross Bun Sign” revealing multiple system atrophy: A case report

#### S. Moudden ^1^; S. Rachidi^2^; O. Cherkaoui Ghazouani^1^

##### ^*1*^ *Neurology Department, Military Hospital of Avicenna, Marrakech, Morocco;* ^*2*^ *Neurology department, University Hospital Center of Marrakech, Marrakech, Morocco*

## EPV‐0758

### If you look, you find: a novel CSF1R gene variant as a possible cause of parkinsonism

#### M. Luz Esteve; M. Ravelo León; D. Gómez de la Torre Morales; J. Rodríguez Carrillo; J. Aguilera Aguilera; I. Díaz Díaz; L. Abad Reguera; A. Hernández González; L. Redondo Robles; J. Velázquez Pérez; F. González Terriza; M. Alañá García; G. Carvalho Monteiro

##### Neurology Department, Complejo Asistencial Universitario de Salamanca, Salamanca, Spain

## EPV‐0759

### Imaging characteristics of ANO10‐related Ataxia

#### O. Tamaš ^1^; S. Pisano^2^; S. Basaia^2^; F. Agosta^2^; A. Milovanović^1^; N. Mazalica^1^; M. Kovačević^1^; M. Filippi^2^; V. Kostić^1^; N. Dragaševic‐Mišković^1^

##### ^*1*^ *Neurology Clinic, Faculty of Medicine, University of Belgrade, Belgrade, Serbia;* ^*2*^ *Vita‐Salute San Raffaele University, Milan, Italy*

## EPV‐0760

### Immunosuppression and Parkinson's disease risk: Unveiling the link – A systematic review of the literature and meta‐analysis

#### J. Nowak ^1^; K. Porębska^2^; A. Kowalska^1^; A. Antoniak^3^; A. Friedman^2^; D. Koziorowski^2^; M. Figura^2^

##### ^*1*^ *Doctoral School of the Medical University of Warsaw, Warsaw, Poland;* ^*2*^ *Department of Neurology, Faculty of Health Sciences, Warsaw, Poland;* ^*3*^ *Student Scientific Group, Department of Neurology, Faculty of Health Sciences, Warsaw, Poland*

## EPV‐0761

### iMOVA: A scalable multimodal prototype for objective movement disorder assessment

#### I. Platt ^1^; K. Sampson^1^; M. Coll I Omana^1^; M. Sartori^1^; N. Guilbeault^2^; A. Almeida^2^; Lopez^2^; J. Frazão^2^; I. Varela^1^; A. Sadnicka^1^

##### ^*1*^ *Department of Clinical and Movement Neurosciences, University College London, London, UK;* ^*2*^ *NeuroGEARS, Sainsbury Wellcome Centre, London, UK*

## EPV‐0762

### Impact of a 3‐month ketogenic diet on sleep quality, daytime sleepiness, and body composition in patients with Parkinson's disease

#### A. Pittino ^1^; F. Filippi^1^; C. Prezza^1^; G. Ermanis^1^; A. Nilo^2^; F. Janes^2^; M. Valente^1^

##### ^*1*^ *Department of Medicine (DMED), University of Udine;* ^*2*^ *Clinical Neurology Unit, Udine University Hospital, Piazzale Santa Maria Della Misericordia, Udine, Italy*

## EPV‐0763

### Improving useability and safety for Parkinson's patients – Use of the ENFit extension tube for delivery of LECIG infusion

#### T. Thomsen; L. Olsen; M. Javidi; N. Karottki; B. Biering‐Sørensen

##### Movement Disorder Clinic, Department of Neurology, Rigshospitalet, Glostrup, Denmark

## EPV‐0764

### Impulse control disorders in Parkinson's disease: associations with dopaminergic burden and dosing patterns

#### E. Cherif; A. Rekik; A. Mili; K. Jemai; E. Jarrar; H. Slimen; S. Naija; A. Hassine; S. Ben Amor ^1^

##### Neurology Department, Sahloul Hospital, Sousse, Tunisia

## EPV‐0765

### Inflammatory processes and the impact of MAO‐B inhibitors in early‐stage idiopathic Parkinson's disease

#### U. Yapıcı; N. Karagöz Sakallı; H. Atakli

##### Neurology, Bakırköy Prof. Dr. Mazhar Osman Training and Research Hospital for Psychiatry, Neurology and Neurosurgery, İstanbul, Türkiye

## EPV‐0766

### Interlocking pentagons task and visuospatial deficit in parkinsonian syndrome: A quantitative approach

#### G. Donzuso ^1^; F. Cilia^1^; E. Ferrante^2^; C. D'Agate^2^; G. Fazzina^1^; C. Cicero^1^; T. Claudio^1^; M. Giovanni^1^; N. Alessandra^1^; M. Zappia^1^

##### ^*1*^ *Department of Medical, Surgical Sciences and Advanced Technologies “GF Ingrassia”, University of Catania, Catania, Italy;* ^*2*^ *AOU Policlinico‐San Marco, Clinica Neurologica, Catania, Italy*

## EPV‐0767

### International multicentre survey of driving and road traffic accidents in Parkinson's disease: The Drive‐PD study

#### K. Poplawska‐domaszewicz ^1^; I. Boura^2^; A. Kurta‐Nowicka^1^; K. Naskręt‐Gasik^1^; S. Haridas^3^; M. Kramberger^4^; V. Metta^3^; K. Wu^5^; M. Chakravarty^6^; C. Falup‐Pecurariu^7^; H. Hussain^3^; A. Nalarketti^3^; T. Masagnay^3^; C. Sangilan^3^; S. Landolfo^7^; C. Santoro^7^; P. Odin^8^; R. van Coller^9^; F. Amod^10^; L. Vlok^11^; S. Chopra^3^; M. Astitha^2^; C. Spanaki;^2^; D. Urso^7^; V. Leta^12^; A. Sauerbier^13^; W. Jost^14^; H. Dafsari^13^; K. Ray Chaudhuri^3^

##### ^*1*^ *Department of Neurology, Poznan University of Medical Sciences, Poznan, Poland;* ^*2*^ *School of Medicine, University of Crete, Heraklion, Crete, Greece;* ^*3*^ *King's College Hospital London Dubai, Dubai, United Arab Emirates;* ^*4*^ *Department of Neurology, University Medical Center Ljubljana, Ljubljana, Slovenia;* ^*5*^ *Parkinson Foundation Centre of Excellence, King's College Hospital NHS Foundation Trust, London, UK;* ^*6*^ *King's College Hospital NHS Foundation Trust, London, UK;* ^*7*^ *Center for Neurodegenerative Diseases and the Aging Brain, Department of Clinical Research in Neurology, University of Bari ‘Aldo Moro’, “Pia Fondazione Cardinale G. Panico”, Tricase, Lecce, Italy;* ^*8*^ *Department of Neurology, Lund University Hospital;* ^*2*^ *21 85 Lund, Sweden;* ^*9*^ *Department of Neurology, Faculty of Health Sciences, University of Pretoria, Pretoria, South Africa;* ^*10*^ *Department of Neurology, University of KwaZulu‐Natal, Durban, South Africa;* ^*11*^ *Department of Neurology, Stellenbosch University, Cape Town, South Africa;* ^*12*^ *Parkinson and Movement Disorders Unit, Department of Clinical Neurosciences, Fondazione IRCCS Istituto Neurologico Carlo Besta, Milan, Italy;* ^*13*^ *Faculty of Medicineand, Department of Neurology, University Hospital Cologne, University of Cologne, Cologne, Germany;* ^*14*^ *Parkinson‐Klinik Ortenau GmbH & Co KG, Kreuzbergstr, Wolfach, Germany*

## EPV‐0768

### Investigating spontaneous smiling in Parkinson's disease through the Whistle–Smile reflex

#### M. De Riggi ^1^; A. Martini^1^; G. Paparella^3^; S. Aloisio^1^; L. Angelini^2^; A. Grandolfo^1^; D. Birreci^1^; D. Costa^1^; L. Marsili^4^; M. Bologna^1^

##### ^*1*^ *Department of Human Neurosciences, Sapienza University of Rome, Italy;* ^*2*^ *IRCCS Neuromed Pozzilli (IS), Italy;* ^*3*^ *Neurophysiopathology Unit, Department of Translational Biomedicine and Neuroscience (DiBraiN), University of Bari Aldo Moro, Bari, Italy;* ^*4*^ *James J. and Joan A. Gardner Center for Parkinson's disease and Movement disorders, Department of Neurology, University of Cincinnati, Cincinnati, OH, USA*

## EPV‐0769

### Is there a relationship between sleep disturbances and bradykinesia features in essential tremor?

#### G. Paparella ^1^; A. Martini^2^; A. Grandolfo^2^; M. Panfili^2^; L. Angelini^3^; M. De Riggi^2^; S. Aloisio^2^; D. Birreci^2^; A. Maraone^2^; F. Bersani^4^; M. Bologna^2^

##### ^*1*^ *Neurophysiopathology Unit, Department of Translational Biomedicine and Neuroscience (DiBraiN), University of Bari Aldo Moro, Bari, Italy;* ^*2*^ *Department of Human Neurosciences, Sapienza, University of Rome, Rome, Italy;* ^*3*^ *IRCCS Neuromed, Pozzilli, IS, Italy;* ^*4*^ *Department of Medico‐Surgical Sciences and Biotechnologies, Sapienza University of Rome, Rome, Italy*

## EPV‐0770

### Ketotic hyperglycemia–related generalized chorea: A rare and atypical presentation

#### M. Vargas Cobos; A. De Luca; R. Alfonso González; M. Capra; C. Gómez López de San Román; L. Caballero Sánchez; I. Bermejo Casado; D. Cerdán Santacruz; A. Castrillo Sanz; A. Mendoza Rodríguez

##### Neurology department, Segovia University General Hospital, Segovia, Spain

## EPV‐0771

### Levodopa‐sparing drugs persistence in advanced Parkinson's disease: a retrospective cohort study on device‐aided therapies

#### M. Molina‐Haro ^1^; I. del Pino‐Diaz^3^; A. Morales‐Lahoz^3^; A. Camero‐Piñatel^1^; J. Romero‐Fábrega^2^; C. Madrid‐Navarro^2^; A. Mínguez‐Castellanos^2^; J. Gutiérrez‐García^3^; F. Escamilla‐Sevilla^2^

##### ^*1*^ *Neurology Service, Hospital Universitario Virgen de las Nieves, Granada, Spain;* ^*2*^ *Neurology Service, Hospital Universitario Virgen de las Nieves, Instituto de Investigación Biosanitaria (ibs.GRANADA), Granada, Spain;* ^*3*^ *Neurology Service. Hospital Universitario Clínico San Cecilio, Granada, Spain*

## EPV‐0772

### Local vascular mimics of asymmetrical limb tremor: A two‐case report

#### J. Cancela ^1^; J. Alves^1^; D. Reis Carneiro^1^; D. Reis Carneiro^2^

##### ^*1*^ *Neurology Department, Coimbra Local Health Unit, Portugal;* ^*2*^ *Faculty of Medicine, University of Coimbra, Portugal*

## EPV‐0773

### Longitudinal assessment of cognitive function in essential tremor

#### F. Novellino ^1^; C. Calomino^1^; R. Nisticò^1^; M. Bianco^1^; M. Bonacci^1^; V. Prigitano^1^; M. Caligiuri^1^; A. Quattrone^1^; M. Salsone^2^; A. Quattrone^1^

##### ^*1*^ *Neuroscience Research Center, Department of Medical and Surgical Science, Magna Grecia University, Catanzaro, Italy;* ^*2*^ *Vita‐Salute San Raffaele University, Milan, Italy*

## EPV‐0774

### Longitudinal changes and clinical utility of the neurological pupillary index in de novo Parkinson's disease

#### J. Lee ^1^; S. You^2^

##### ^*1*^ *Department of Neurology, Dongsan Hospital, Keimyung University School of Medicine, Daegu, Republic of Korea;* ^*2*^ *Department of Neurology, Seoul Medical Center, Seoul, Republic of Korea*

## EPV‐0775

### Long‐term 4‐aminopyridine efficacy in SCA27B: Up to 18‐month case series

#### Á. López Prado; M. Martínez Seijas; M. Callejo Seguela; N. Marcos Fernández; A. Lagüela Alonso; V. Anciones Martín; A. Rodríguez‐Antigüedad Zarrantz; I. Rouco Axpe

##### Neurology Department, Cruces University Hospital, Bilbao, Spain

## EPV‐0776

### Longterm efficacy and safety of levodopa/entacapone/carbidopa intestinal gel in patients with advanced Parkinson's disease in Slovakia

#### M. Minar ^1^; I. Straka^1^; Z. Andre^1^; M. Grofik^2^; J. Necpal^3^; V. Han^4^; M. Ockova^5^

##### ^*1*^ *Faculty of Medicine, Comenius University Bratislava, Slovakia;* ^*2*^ *Jessenius Faculty of Medicine Martin, Comenius University Bratislava, Slovakia;* ^*3*^ *Neurology Department, Hospital Zvolen, Slovakia;* ^*4*^ *Faculty of Medicine, Pavol Jozef Safarik University Košice, Slovakia;* ^*5*^ *Neurology Department, Slovak Medical University and University Hospital Bratislava, Slovakia*

## EPV‐0777

### Lowering the occlusion pressure detection setting of the CRONO® LECIG pump can have a positive impact on device useability and safety

#### B. Biering‐Sørensen^1^; L. Olsen^2^; M. Javidi^3^; T. Thomsen^1^; N. Holm^4^; N. Karottki ^2^

##### ^*1*^ *Movement Disorder and Pain Research Centre, Department of Neurology, Rigshospitalet, Copenhagen, Denmark;* ^*2*^ *Movement Disorder Clinic, Department of Neurology, Rigshospitalet, Copenhagen, Denmark;* ^*3*^ *Department of Public Health, Faculty of Health and Medical Sciences, University of Copenhagen, Denmark;* ^*4*^ *Innoventa Medica, Birkeroed, Denmark*

## EPV‐0778

### Magnetic resonance imaging‐based analysis of morphometric changes in grey matter in patients with Parkinson's disease and depression

#### J. Betz ^1^; T. Meindl^1^; T. Mantel^1^; J. Kirschke^2^; B. Haslinger^1^

##### ^*1*^ *Department of Neurology, Klinikum rechts der Isar, TUM School of Medicine and Health, Technical University of Munich, Munich, Germany;* ^*2*^ *Department of Diagnostic and Interventional Neuroradiology, Klinikum rechts der Isar, TUM School of Medicine and Health, Technical University of Munich, Munich, Germany*

## EPV‐0779

### Abstract withdrawn

## EPV‐0780

### Mirror paresis of the contralateral limb following botulinum toxin injection

#### A. Câmara ^1^; A. Araújo^2^; C. Figueira^2^; T. Aguiar^1^; P. Pita Lobo^1^

##### ^*1*^ *Department of Neurology, Hospital Central do Funchal, Funchal, Portugal;* ^*2*^ *Department of Neuroradiology, Hospital Central do Funchal, Funchal, Portugal*

## EPV‐0781

### Mortality trends in Stroke and paralytic syndrome among older adults in the United States: A 24‐year retrospective analysis

#### Z. Ul Abedin ^1^; M. Khan^2^; E. Umair^3^; A. Iftikhar^1^

##### ^*1*^ *King Edward Medical University*, *Lahore; Pakistan;* ^*2*^ *CMH Lahore Medical College*, *Lahore; Pakistan*; ^*3*^ *Nishtar Medical University, Multan*, *Pakistan*

## EPV‐0782

### Motor phenotype of familial Parkinson's disease: A Tunisian cohort study

#### A. Mousli; R. Zouari; S. Zakaria; F. Nabli; A. Rachdi; D. Ben Mohamed; S. Ben Sassi

##### Department of Neurology, National Institute of Neurology Mongi Ben Hamida, Tunis, Tunisia

## EPV‐0783

### Multimodal mixed‐reality assessment for automated prediction of Movement Disorder Society clinical ratings

#### D. Hemmerling ^1^; M. Wójcik‐Pędziwiatr^2^; B. Krawczyk^3^; W. Szecowska^1^; M. Rudzinska‐Bar^2^

##### ^*1*^ *AGH University of Cracow, Cracow, Poland; Samsung R&D Institute, Poland;* ^*2*^ *Department of Neurology, Frycz Modrzewski Cracow University, Cracow, Poland;* ^*3*^ *AGH University of Cracow, Cracow, Poland*

## EPV‐0784

### Neuroinflammatory and axonal biomarkers as decision‐support tools in Parkinson's disease and atypical Parkinsonism

#### D. Akramova ^1^; G. Rakhimbaeva^1^; T. Bobomuratov^1^; N. Vakhabova^1^; D. Sobirova^1^; S. Khamdamov^2^

##### ^*1*^ *Tashkent State Medical University, Tashkent*, *Uzbekistan*; ^*2*^ *Tashkent State Economic University¸ Tashkent*, *Uzbekistan*

## EPV‐0785

### Neuropsychiatric outcomes after STN‐DBS versus stereotactic lesioning in Parkinson's disease

#### A. Ivanov ^1^; E. Khabarova^2^; S. Kim^1^; J. Rzaev^1^

##### ^*1*^ *Neurosurgeon, Department of Functional Neurosurgery, Federal Neurosurgical Center, Novosibirsk, Russian Federation;* ^*2*^ *Neurologist, Department of Functional Neurosurgery, Federal Neurosurgical Center, Novosibirsk, Russian Federation*

## EPV‐0786

### nOH ratio and risk of falls in patients with Parkinson disease

#### N. Centno Pique; J. Camacho Velasquez; P. Bermell Campos; M. Bea Sintes; V. Adell Ortega; E. Franquet Gomez

##### Department of Neurology, Consorci Sanitari Alt‐Penedes Garraf, Barcelona, Spain

## EPV‐0787

### Non‐motor subtypes in Parkinson's disease and atypical parkinsonian syndromes: A cluster‐based longitudinal study from the PROATYP cohort

#### M. Makrygianni ^1^; D. Potamianou^1^; T. Pipikos^1^; L. Florentin^2^; A. Sarafi^1^; E. Papadogeorgaki^1^; C. Mourmouris^1^; M. Xilouri^3^; M. Stamelou^1^

##### ^*1*^ *Hygeia Hospital, Athens, Greece;* ^*2*^ *AlphaLab, Center of Molecular Biology and Cytogenetics, Athens, Greece;* ^*3*^ *Biomedical Research Foundation Academy of Athens (Bioacademy), Athens, Greece*

## EPV‐0788

### Non‐motor symptoms in Parkinson's disease are under‐recognised in routine care: Evidence from an Uzbek cohort

#### D. Pulatova; F. Saidvaliev

##### Department of Neurology, Tashkent State Medical University, Tashkent, Uzbekistan

## EPV‐0789

### Nothing alike: A PSP‐like case

#### A. Pires; H. Rodrigues; M. Rego; R. Rodrigues

##### Department of Neurology, Entre Douro e Vouga Local Health Unit, Santa Maria da Feira, Portugal

## EPV‐0790

### OPAL‐PD: A longitudinal observational study assessing health‐related quality of life after opicapone initiation in Parkinson's disease

#### M. Ippolito ^1^; G. Harrison‐Jones^1^; L. Akindele^1^; E. Shelton^2^; A. Noyce^3^

##### ^*1*^ *Bial Pharma UK Ltd, Windsor, UK;* ^*2*^ *Cohort Science, London, UK;* ^*3*^ *Centre for Preventive Neurology, Wolfson Institute of Population Health, Faculty of Medicine and Dentistry, Queen Mary University of London, London, UK*

## EPV‐0791

### Optimising DBS for dystonia using local field potentials: LFP‐DYT protocol and anticipated early cohort data

#### D. Ledingham ^1^; R. Mills^1^; M. Gibbs^1^; M. Maynes^2^; A. Pal^2^; R. Iredale^2^; V. Foster^2^; S. Ong^1^; S. Sathyanarayana^2^; A. Jenkins^1^; C. Nicholson^1^; M. Hussain^1^; M. Baker^3^; N. Pavese^2^

##### ^*1*^ *Department of Neurosciences, Newcastle Upont Tyne NHS Foundation Trust, Newcastle Upon Tyne, UK;* ^*2*^ *Clinical Ageing Research Unit, Newcastle University, Newcastle Upon Tyne, UK;* ^*3*^ *Translational and Clinical Research Institute, Newcastle University, Newcastle Upon Tyne, UK*

## EPV‐0792

### Orofacial drinking tremor: A case series and literature review

#### S. Cirinei ^1^; D. Birreci^1^; L. Angelini^2^; S. Aloisio^1^; A. Grandolfo^1^; A. Martini^1^; M. De Riggi^1^; M. Bologna^1^

##### ^*1*^ *Department of Human Neurosciences, Sapienza University of Rome, Rome, Italy;* ^*2*^ *IRCCS Neuromed, Pozzilli (IS), Italy*

## EPV‐0793

### Outcome of subthalamic stimulation in tremor‐dominant Parkinson's disease

#### A. Papp ^1,2^; A. Babakhani^2,3,4^; L. Halász^5^; L. Erőss^2^; G. Miklós^5^; G. Fekete^5^; L. Bognár^6^; A. Szilágyi^7^; G. Tamás^1^

##### ^*1*^ *Department of Neurology, Semmelweis University, Budapest, Hungary;* ^*2*^ *János Szentágothai Doctoral School of Neurosciences, Semmelweis University, Budapest, Hungary;* ^*3*^ *Athinoula A. Martinos Center for Biomedical Imaging, Massachusetts General Hospital Boston, US;* ^*4*^ *Department of Neurology, Massachusetts General Hospital, Boston, US;* ^*5*^ *Department of Neurosurgery and Neurointervention, Semmelweis University, Budapest, Hungary;* ^*6*^ *Department of Neurosurgery, University of Debrecen, Debrecen, Hungary;* ^*7*^ *Department of Emergency Medicine, Semmelweis University, Budapest, Hungary*

## EPV‐0794

### Overlapping etiologies in hpca‐related dystonic syndrome

#### F. Musso ^1^; M. Petracca^2^; D. Genovese^1^; A. Bentivoglio^1^

##### ^*1*^ *Department of Neurology, Università Cattolica del Sacro Cuore, Rome, Italy;* ^*2*^ *Neurology Unit, Fondazione Policlinico Universitario Agostino Gemelli IRCCS, Rome, Italy*

## EPV‐0795

### Pain in Parkinson's disease

#### S. Gjata; I. Zekja; J. Kruja

##### Department of Neurology, Mother Theresa University Hospital Center, Tirana, Albania

## EPV‐0796

### Painful Legs and Moving Toes Syndrome: When treatable factors make the difference

#### L. Francisco ^1^; J. Santos^2^; A. Peralta^3^

##### ^*1*^ *Neurology Department, ULS Alto Minho, Viana do Castelo, Portugal;* ^*2*^ *Neurology Department, ULS Estuário do Tejo, Vila Franca de Xira, Portugal;* ^*3*^ *Laboratory of EEG/Sleep, Department of Neurosciences and Mental Health, Hospital de Santa Maria, Lisboa*

## EPV‐0797

### Pallidal bursting correlates with dystonia severity

#### A. Al Habbal ^1^; S. Falciglia^2^; N. Pozzi^1^; C. Matthies^3^; J. Volkmann^1^; C. Palmisano^1^; A. Mazzoni^2^; P. Capetian^1^; I. Isaias^1^

##### ^*1*^ *Department of Neurology, University Hospital of Wuerzburg and Julius Maximilian University of Wuerzburg, Wuerzburg, Germany;* ^*2*^ *The BioRobotics Institute and Department of Excellence in Robotics and AI, Scuola Superiore Sant’Anna, Pontedera, Italy;* ^*3*^ *Department of Neurosurgery, University Hospital Wuerzburg and Julius Maximilian University of Wuerzburg, Wuerzburg, Germany*

## EPV‐0798

### Paradoxical tremor amplification and impaired arithmetic performance under cognitive load in functional tremor

#### M. Cibula; N. Prezelj; R. Berlot; M. Kojović

##### Department of Neurology, University Medical Centre Ljubljana, Ljubljana, Slovenia

## EPV‐0799

### Parkinson's Disease (PD) and predictors of Driving Cessation: A 7‐year longitudinal prospective study

#### P. Stamatelos ^1^; I. Beratis^2^; P. Hatzaki^1^; A. Economou^3^; N. Andronas^1^; D. Pavlou^4^; S. Fragkiadaki^1^; D. Kontaxopoulou^1^; A. Bonakis^5^; L. Stefanis^1^; G. Yannis^6^; S. Papageorgiou^1^

##### ^*1*^ *1st Department of Neurology, Medical School, National and Kapodistrian University of Athens, Eginition Hospital, Athens, Greece;* ^*2*^ *Psychology Department, The American College of Greece, Deree, Athens, Greece;* ^*3*^ *Department of Psychology, National and Kapodistrian University of Athens, Athens, Greece;* ^*4*^ *School of Topography and Geoinformatics, University of West Attica, Athens, Greece;* ^*5*^ *2nd Department of Neurology, Medical School, National and Kapodistrian University of Athens, Attikon Hospital, Athens, Greece;* ^*6*^ *Department of Transportation Planning and Engineering, School of Civil Engineering, National Technical University of Athens, Athens, Greece*

## EPV‐0800

### Patient adherence to botulinum toxin therapy in the context of dystonia epidemiology (real‐world data from western Moscow)

#### A. Smirnov ^1^; O. Tsemka^1^; Bezrukova^1^; E. Poneveshskaya^2^

##### ^*1*^ *N.I. Pirogov Russian National Research Medical University, Moscow, Russian Federation;* ^*2*^ *Zhadkevich Moscow City Clinical Hospital, Moscow, Russian Federation*

## EPV‐0801

### Patient satisfaction with genetic counseling in GBA1‐associated Parkinson's disease

#### H. Sari; B. Çelik; M. Akbostancı; R. Yilmaz

##### Department of Neurology, Ankara University Faculty of Medicine, Ankara, Türkiye

## EPV‐0802

### Patient voices driving innovation in drug delivery: How user insights guide improvements to the Crono® LECIG pump

#### V. Correa‐Gallego ^1^; N. Smith^1^; C. Canè^2^; S. Papaveri^2^

##### ^*1*^ *Britannia Pharmaceuticals Limited, Green Park, Reading, UK;* ^*2*^ *CANÈ S.p.A. Medical Technology, Cascine Vica, Italy*

## EPV‐0803

### Peripheral lipid and inflammatory markers in Parkinson's disease: Clinical associations of ApoA1 and CRP

#### A. Mishra; S. Rout; A. Verma; R. Shukla

##### All India Institute of Medical Sciences, Raebareli, Uttar Pradesh, India

## EPV‐0804

### PGC‐1α small molecule activator attenuates dopaminergic degeneration in Parkinson's disease via regulating mitochondrial homeostasis

#### J. Kaur; S. Naqvi

##### 1Department of Pharmacology and Toxicology, National Institute of Pharmaceutical Education and Research (NIPER‐R), Lucknow (UP), India

## EPV‐0805

### Population characteristics and motor fluctuation detection in early Parkinson's disease using a wearable sensor

#### J. Rodriguez Carrillo; M. Ravelo Leon; D. Gomez de la Torre; I. Diaz Diaz; M. Luz Esteve; L. Abad Reguera; A. Hernandez Gonzalez; l. Redondo Robles; G. Carvalho Monteiro; J. Aguilera Aguilera

##### Salamanca University Hospital Complex, Salamanca, Spain

## EPV‐0806

### Posterior tibial nerve stimulation in the management of urinary symptoms in idiopathic Parkinson's disease: The National Hospital of Pikine experience

#### F. Niakam Mbouleup ^1,2^; N. Mundih^1,2^; M. Ngoule^1,2^; A. Ngassaki^1,2^; S. Sow^1,2^; A. Diop^3^; J. Kahwagi^3^; C. Kwama^1,2^; M. Dia^1,2^; S. Jouonang^1,2^; C. Bangweni^1,2^; M. Bonkoulou^1,2^; A. Faye^1,2^; C. Samba^1,2^; S. Kamali^1,2^; M. Nidiaye^1,2^; M. Fall^1,3^

##### ^*1*^ *Cheikh Anta Diop University, Dakar, Senegal;* ^*2*^ *Fann National University Hospital, Dakar, Senegal;* ^*3*^ *Pikine National Hospital Center, Dakar, Senegal*

## EPV‐0807

### Precision disease stratification in Parkinson's disease: Fairness‐aware imputation and longitudinal subtype discovery

#### M. Hani^1^; F. Ouardirhi ^2^; M. Hamzaoui^3^; S. Mahmoudi^1^; M. Benjelloun^1^

##### ^*1*^ *Department of Computer Engineering and Management, Faculty of Engineering, University of Mons (UMONS), Mons, Belgium;* ^*2*^ *Department of Computer Science and Systems Analysis, ENSIAS, Université Mohammed V de Rabat, Rabat, Morocco;* ^*3*^ *Department of Computer Engineering, ESPRIT, Ariana, Tunisia*

## EPV‐0808

### Predicting MSA andPSP based on clinical and neuropsychological scores by employing machine learning

#### A. Bougea^1^; Y. Degirmenci ^2^; P. Poulos^3^; C. Kalyvas^4^; E. Angelopoulou^1^; P. Zikos^5^

##### ^*1*^ *National and Kapodistrian University of Athens, Greece;* ^*2*^ *Istanbul Health and Technology University, Faculty of Medicine, Department of Neurology, Istanbul, Türkiye;* ^*3*^ *School of Electrical and Computer Engineering, National Technical University of Athens, Athens, Greece;* ^*4*^ *FSP Biometrics, Fortrea, Athens, Greece;* ^*5*^ *Department of Neurology, Air Force General Hospital, Athens, Greece*

## EPV‐0809

### Predictive model of one‐year motor outcomes after bilateral STN deep brain stimulation in Parkinson's disease: An internally validated study

#### M. Tsalta‐Mladenov ^1^; V. Dimitrova^2^; M. Moynov^3^; S. Andonova^1^; Y. Enchev^3^

##### ^*1*^ *Department of Neurology and Neuroscience, Faculty of Medicine, Medical University “Prof. Paraskev Stoyanov”, Varna, Bulgaria;* ^*2*^ *Department of Optometry and Occupational Diseases, Faculty of Public Health, Medical University “Prof. Paraskev Stoyanov”, Varna, Bulgaria;* ^*3*^ *Department of Neurosurgery, Faculty of Medicine, Medical University “Prof. Paraskev Stoyanov”, Varna, Bulgaria*

## EPV‐0810

### Prevalence and clinical features of Jaw Tremor in Parkinson's disease

#### R. Ismayilov ^1^; Y. Değirmenci^2^

##### ^*1*^ *Neurology Clinic, İstinye University, Gaziosmanpaşa Medical Park Hospital, İstanbul, Türkey;* ^*2*^ *Department of Neurology, İstanbul Health and Technology University Medical Faculty, İstanbul, Türkey*

## EPV‐0811

### Prevalence of burning mouth syndrome in Parkinson's disease: A systematic review and meta‐analysis

#### A. Hamad ^1^; I. Alkhawaldeh^2^

##### ^*1*^ *Faculty of Medicine, Menoufia University, Menoufia, Egypt;* ^*2*^ *Faculty of Medicine, Mutah University, Al‐Karak, Jordan*

## EPV‐0812

### Problematic internet use in Parkinson's disease patients: Risk factors and independent predictors

#### B. Radojević ^1^; N. Dragašević‐Mišković^3^; D. Milenković^2^

##### ^*1*^ *Clinical Hospital Center Zvezdara, Department of Neurology, Belgrade, Serbia;* ^*2*^ *Department of Physics and Mathematics, University of Belgrade – Faculty of Pharmacy, Belgrade, Serbia;* ^*3*^ *Clinic of Neurology, University Clinical Centre of Serbia, Faculty of Medicine, University of Belgrade, Serbia*

## EPV‐0813

### Prodromal symptoms in multiple system atrophy and Parkinson's disease: A comparative study

#### I. Menchetti ^1^; J. Pasquini^1^; E. Benevento^1^; G. Palermo^1^; D. Frosini^2^; R. Ceravolo^1^

##### ^*1*^ *Department of Clinical and Experimental Medicine, University of Pisa, Pisa, Italy;* ^*2*^ *Azienda Ospedaliero‐Universitaria Pisana, Pisa, Italy*

## EPV‐0814

### Progressive supranuclear palsy mimics‐a cohort with redeemable features

#### D. Kalikavil Puthanveedu; A. Cherian

##### Department of Neurology, Sree Chitra Tirunal Institute for Medical Sciences and Technology, Thiruvananthapuram, India

## EPV‐0815

### Proteomics – Potential biomarker of iNPH shunting outcome

#### M. Dudzic^1^; A. Kwiecień ^1^; B. Sokół^2^; J. Kalka^2^; A. Lemański^2^

##### ^*1*^ *Department of Neurology, Municipal Hospital in Poznań, Poland;* ^*2*^ *Department of Neurosurgery, Municipal Hospital in Poznań, Poland*

## EPV‐0816

### Quantitative analysis of reflexive and voluntary saccades in Parkinson disease and multiple system atrophy

#### H. Kim ^1^; Y. Bae^2^; J. Yun^2^

##### ^*1*^ *Department of Neurology, Seoul National University Bundang Hospital, Seongnam, Republic of Korea;* ^*2*^ *Department of Neurology, College of Medicine, Ewha Womans University Seoul Hospital, Seoul, Republic of Korea*

## EPV‐0817

### SAFIN‐DREAM: Real‐world effectiveness of safinamide on non‐motor symptoms as first add‐on therapy to levodopa

#### F. Salazar Hernández ^1^; M. Ruiz Perelló^1^; B. Gómez Gozálvez^1^; D. López Segura^1^; A. Savolainen^1^; M. Lorente^1^; A. Sánchez Pedrós^1^; B. Palazón Cabanes^2^; M. Gómez Pacheco^1^; D. Vidal Mena^1^; J. Bermejillo Barrera^1^; A. Báidez Guerrero^1^; M. Cerdán Sánchez^1^

##### ^*1*^ *Neurology Department, Hospital Santa Lucía, Cartagena, España;* ^*2*^ *Neurology Department, Hospital Lorenzo Guirao, Murcia, España*

## EPV‐0818

### Satisfaction with and functional impact of subcutaneous Foslevodopa/Foscarbidopa treatment – User perspective

#### M. Palermo; M. Saianda Duarte; V. Fonseca; J. Neiva Correia; T. Lampreia; J. Vale; R. Simões

##### Neurology Department, Hospital Beatriz Ângelo, Loures, Portugal

## EPV‐0819

### SCA3 mitochondrial phenotype is rescued by an antisense oligonucleotide targeting expanded CAG repeats in Ataxin‐3 pre‐mRNA

#### R. Abeti^1^; S. Nethisinghe^1^; C. Beekman^2^; R. Weij^2^; J. Alwan^1^; C. Gonzalez‐Robles^1^; N. Iqbal^1^; N. Datson^2^; P. Giunti ^1^

##### ^*1*^ *Ataxia Centre, Department of Clinical and Movement Neurosciences, UCL Queen Square Institute of Neurology, Queen Square House, Queen Square, London, UK;* ^*2*^ *VICO Therapeutics; B.V., Leiden, The Netherlands*

## EPV‐0820

### Sebum and cerumen analysis for the identification of biomarkers in Parkinson's disease

#### K. Mazinke ^1^; H. Zhang^2^; P. Jones^3^; L. Sun^2^; A. Rajput^4^; E. Noyes^4^

##### ^*1*^ *College of Medicine, University of Saskatchewan, Saskatoon, Canada;* ^*2*^ *Department of Agriculture and Bioresources, University of Saskatchewan, Saskatoon, Canada;* ^*3*^ *School of Environment and Sustainability, University of Saskatchewan, Saskatoon, Canada;* ^*4*^ *Saskatchewan Health Authority & College of Medicine, University of Saskatchewan, Saskatoon, Canada*

## EPV‐0821

### Sequential use of device‐aided therapies in advanced Parkinson's disease: An individualized strategy

#### M. Morales‐Casado; G. Tabar‐Comellas; D. Rivero‐Rodriguez; N. López‐Ariztegui

##### Movement Disorders Unit, Department of Neurology, Toledo University Hospital, Toledo, Spain

## EPV‐0822

### Sex differences in the severity of non‐motor symptoms in Parkinson's disease: A systematic review and meta‐analysis

#### C. Terravecchia ^1^; C. Cicero^1^; L. Pettinato^2^; S. Tabbì^3^; G. Donzuso^1^; D. Contrafatto^1^; G. Mostile^1^; M. Zappia^1^; A. Nicoletti^1^

##### ^*1*^ *University of Catania, Department “G.F. Ingrassia”, Section of Neurosciences, Catania, Italy;* ^*2*^ *Department of Clinical and Experimental Medicine, University of Catania, Catania, Italy;* ^*3*^ *Department of Neuroscience, Psychology, Drug Research and Child Health, University of Florence, Florence, Italy*

## EPV‐0823

### Simulation of Foslevodopa/Foscarbidopa extra doses to achieve levodopa steady‐state exposures following nighttime infusion rate reduction

#### S. Stodtmann^1^; M. Rosebraugh ^2^

##### ^*1*^ *AbbVie Deutschland GmbH & Co. KG, Ludwigshafen, Germany;* ^*2*^ *AbbVie Inc., North Chicago, USA*

## EPV‐0824

### Skin biomarkers for neurodegenerative disorders

#### A. Kwan Su Huey ^1^; E. Karabulut^2^; R. Parfait Kamaha Kamaha^3^; M. Uzun^4^; T. Maria Nader^5^

##### ^*1*^ *Department of Geriatrics, Manchester University NHS Foundation Trust, Greater Manchester, UK;* ^*2*^ *Department of Emergency Medicine, Vakfikebir State Hospital, Trabzon, Türkiye;* ^*3*^ *School of Pharmacy and Health Sciences, United States International University Africa, Nairobi, Kenya;* ^*4*^ *Department of Medicine, Karadeniz Technical University, Trabzon, Türkiye;* ^*5*^ *School of Pharmacy, Lebanese American University, Jbeil, Lebanon*

## EPV‐0825

### Sleep as a physiological window into cervical dystonia: REM suppression and multidomain PSG abnormalities

#### M. Lamba; S. Roshan; G. Rajan

##### Department of Neurology, Govind Ballabh Pant Institute of Postgraduate Medical Education and Research, New Delhi, India

## EPV‐0826

### Sleep quality in patients – A cross‐sectional audit of patients with and without Deep brain stimulation

#### K. Camilleri

##### Neurosciences Department, Mater Dei Hospital, Msida, Malta

## EPV‐0827

### Sleep quality in patients with Huntington's disease

#### C. Cerejo; G. Hemicker; E. Hametner; A. Stefani; A. Djamshidian‐Tehrani; B. Högl; B. Heim; K. Seppi

##### Department of Neurology, Medical University of Innsbruck, Innsbruck, Austria

## EPV‐0828

### SMaRT‐PD: Developing a scalable and explainable clinical decision support system for Parkinson's disease management

#### P. Onyeachu^1^; S. Kim^1^; K. Katie Bounsall^2^; C. Carroll ^1^; E. Meinert^1^

##### ^*1*^ *Newcastle University, Newcastle upon Tyne, UK;* ^*2*^ *University of Plymouth, Plymouth, UK*

## EPV‐0829

### Smartwatch‐based assessment of tremor and dyskinesia in Parkinson's disease in the Uzbek population

#### D. Pulatova; F. Saidvaliev

##### Department of Neurology, Tashkent State Medical University, Tashkent, Uzbekistan

## EPV‐0830

### Spectrum of genetically confirmed ataxias in the Latvian population: A single‐center experience

#### K. Lazdovska ^2^; A. Baborikina^1^; R. Valante^1^; V. Kenina^2^

##### ^*1*^ *Neurology dpeartment, Pauls Stradiņš Clinical University Hospital, Riga, Latvia;* ^*2*^ *Biology and microbiology department, Rīga Stradiņš University, Riga, Latvia*

## EPV‐0831

### Stroke in Friedreich ataxia: Prevalence and cardioembolic risk beyond CHA2DS2‐VA

#### C. Guedes Vaz ^1^; D. Dinis^1^; S. Costa^1^; J. Martins^2^; M. Gago^3^; E. Lourenço^4^; J. Sequeiros^5^; J. Barros^1^; A. Meireles^6^; P. Rodrigues^6^; J. Damásio^1^

##### ^*1*^ *Serviço de Neurologia, Unidade Local de Saúde de Santo António, Porto, Portugal;* ^*2*^ *Serviço de Neuropediatria, Unidade Local de Saúde de Santo António, Porto, Portugal;* ^*3*^ *Serviço de Neurologia, Unidade Local de Saúde do Alto Ave, Guimarães, Portugal;* ^*4*^ *Serviço de Neurologia, Unidade Local de Saúde de Braga, Braga, Portugal;* ^*5*^ *Instituto de Biologia Molecular e Celular – Universidade do Porto, Porto, Portugal;* ^*6*^ *Serviço de Cardiologia, Unidade Local de Saúde de Santo António, Porto, Portugal*

## EPV‐0832

### Subcutaneous levodopa in advanced Parkinson's disease: Is it feasible beyond tertiary centers and ‘ideal’ patients?

#### D. Cerdán Santacruz; C. Gómez López de San Román; M. Vargas; A. De Luca; R. Alfonso González; M. Capra; I. Bermejo Casado; L. Caballero Sánchez; J. Berrío; G. Suárez Fernández; A. Castrillo Sanz; A. Mendoza Rodríguez

##### General Hospital Complex of Segovia, Segovia, Spain

## EPV‐0833

### Sweet spot for pallidal deep brain stimulation in the dystonias

#### J. Perez‐Sanchez ^1^; B. De La Casa‐Fages^1^; O. Mateo Sierra^2^; C. Miranda Herrero^3^; I. Garrido Morro^4^; E. Luque Buzo^1^; C. Serra Smith^1^; J. Garbizu Vidorreta^2^; T. Navarro Rivera^5^; A. Contreras Chicote^1^; V. Perez del Olmo^1^; A. Bonilla Tena^1^; P. Fernandez^4^; I. Herrera^4^; P. Elvira^4^; A. Chacon^3^; M. Vazquez^3^; J. Guzmán‐De‐Villoria^4^; C. Fernandez Carballal^2^; F. Grandas^1^

##### ^*1*^ *Movement Disorders Unit, CSUR, European Reference Network for Rare Neurological Diseases (ERN RND), Neurology Department, Hospital General Universitario Gregorio Marañón, Madrid, Spain;* ^*2*^ *Neurosurgery department, Hospital General Universitario Gregorio Marañón, Madrid, Spain;* ^*3*^ *Pediatric Neurology Unit, Pediatrics Department, Hospital General Universitario Gregorio Marañón, Madrid, Spain;* ^*4*^ *Radiology Department, Hospital General Universitario Gregorio Marañón, Madrid, Spain;* ^*5*^ *Psychiatry Department, Hospital General Universitario Gregorio Marañón, Madrid, Spain*

## EPV‐0834

### Systematic review of deep brain stimulation effects on non‐motor symptoms in Parkinson's disease: STN versus GPi

#### G. PAnagou ^1^; I. Tyrovolas^1^; P. Tsitsopoulos^2^; G. Tagaris^1^

##### ^*1*^ *Department of Neurology, Georgios Gennimatas General Hospital, Athens, Greece;* ^*2*^ *Department of Neurosurgery, at Hippokration General Hospital in Thessaloniki, Greece*

## EPV‐0835

### Thalamic lesion tremor: Comparing a typical and an atypical case

#### F. Santachiara ^1^; D. Birreci^1^; L. Angelini^1^; A. Grandolfo^1^; M. de Riggi^1^; S. Aloisio^1^; M. Passaretti^1^; M. Bologna^1^; M. Bologna^2^

##### ^*1*^ *Department of Human Neurosciences, Sapienza University of Rome, Rome, Italy;* ^*2*^ *IRCCS Neuromed, Pozzilli (IS), Italy*

## EPV‐0836

### The association of GSTT1 deletion, HindIII C>G PAI‐1, and rs11808092 polymorphisms linked to Parkinson's disease vulnerability in Egyptian cohort

#### H. Elshebawy ^1^; H. El‐Bassyouni^2^

##### ^*1*^ *Neurology Department, Cairo University, Cairo, Egypt;* ^*2*^ *Clinical Genetics Department, Human Genetics and Genome Research Institute, National Research Centre, Cairo, Egypt*

## EPV‐0837

### The burden of Friedreich ataxia in Brazil: Results of an online survey

#### M. Cavalcante França Júnior^1^; J. Schmier^2^; J. Tsai^2^; S. Jayade^2^; R. Verma^2^; A. Drenth^3^; A. Harrington ^3^

##### ^*1*^ *Department of Neurology, School of Medical Sciences, University of Campinas (UNICAMP), Campinas, São Paulo, Brazil;* ^*2*^ *HEOR & Market Access, OPEN Health, New York, USA;* ^*3*^ *Biogen International GmbH, Baar, Switzerland*

## EPV‐0838

### The Burden of Friedreich Ataxia in Europe: Results of an online survey

#### A. Darling^1^; P. Giunti^2^; J. Greenfield^3^; M. Grobe‐Einsler^4^; B. Kearney^5^; M. Rai^6^; F. Saccà^7^; J. Schmier^8^; J. Tsai^8^; S. Jayade^8^; R. Verma^8^; A. Drenth^9^; A. Harrington ^9^

##### ^*1*^ *Neurology Department, Ataxia Reference Center, Neurometabolic Unit, Movement Disorders Unit, Sant Joan de Déu Children's Hospital, Barcelona, Spain;* ^*2*^ *Ataxia Centre, Clinical and Movement Neurosciences, UCL Queen Square Institute of Neurology, University College London, London, UK;* ^*3*^ *Euro‐ataxia, London, UK; Ataxia UK, London, UK;* ^*4*^ *Center for Neurology, Department of Parkinson's Disease, Sleep and Movement Disorders, University Hospital Bonn, Bonn, Germany;* ^*5*^ *Friedreich's Ataxia Research Alliance (FARA Ireland), Dunlavin, Ireland;* ^*6*^ *Friedreich's Ataxia Research Alliance (FARA Europe), The Netherlands;* ^*7*^ *Department of Neurosciences and Reproductive and Odontostomatological Sciences, University “Federico II”, Naples, Italy;* ^*8*^ *OPEN Health, HEOR & Market Access, New York, NY, USA;* ^*9*^ *Biogen International GmbH, Baar, Switzerland*

## EPV‐0839

### The Burden of Friedreich Ataxia in Italy: Results of an online survey

#### F. Saccà^1^; J. Schmier^2^; J. Tsai^2^; S. Jayade^2^; R. Verma^2^; A. Drenth^3^; A. Harrington ^3^

##### ^*1*^ *Department of Neurosciences and Reproductive and Odontostomatological Sciences, University “Federico II”, Naples, Italy;* ^*2*^ *HEOR & Market Access, OPEN Health, New York, USA;* ^*3*^ *Biogen International GmbH, Baar, Switzerland*

## EPV‐0840

### The clinical and etiological spectrum of Hemiballismus: A case series

#### Ö. Çakir Nartsoy; E. Aciman Demirel; A. Başel Pala; K. Işik; M. Açikgöz; U. Çelebi; G. Taşçatan; H. Atasoy

##### Norology Department/Zonguldak Bülent Ecevit University Training and Research Hospital, Türkiye

## EPV‐0841

### The compelling case of Parkinson's disease presenting as pseudo‐orthostatic tremor: Valuable insights from the neurophysiology lab

#### T. Andrade Martins ^1^; C. Alves^1^; P. Pereira^1^; C. Rodrigues^1^; M. Grunho^1,2^

##### ^*1*^ *Serviço de Neurologia do Hospital Garcia de Orta, Unidade Local de Saúde de Almada‐Seixal, Portugal;* ^*2*^ *Egas Moniz Center for Interdisciplinary Research (CiiEM), Egas Moniz School of Health Science, Caparica, Almada, Portugal*

## EPV‐0842

### The identification of a single axis to Archimedes spiral drawing and its correlates in terms of tremor's type and severity

#### A. Marwa; R. Arwa; G. Rihab; M. Ahmed; L. Senda; R. Islem; S. Ben Amor

##### Department of Neurology of Sahloul University Hospital, Sousse, Tunisia

## EPV‐0843

### The impact of Subthalamic Deep Brain Stimulation on socio‐occupational functioning in Parkinson's disease

#### G. Imbalzano; E. Montanaro; C. Ledda; A. Romagnolo; S. Budicin; C. Pandino; R. Tucci; L. Lopiano; M. Zibetti

##### University of Turin Department of Neuroscience “Rita Levi Montalcini”, Turin, Italy

## EPV‐0844

### The influence of anxiety and depression over the sleep disturbancies in Parkinson's disease

#### C. Falup‐Pecurariu ^1^; I. Murasan^2^; V. Monescu^3^; C. Kakucs^4^; S. Diaconu^1^

##### ^*1*^ *Department of Neurology, County Clinic Hospital, Transilvania University Brasov, Romania;* ^*2*^ *Department of Neurology, County Clinic Hospital, Brasov, Romania;* ^*3*^ *Faculty of Mathematics and Computer Science, Transilvania University, Brasov, Romania;* ^*4*^ *Faculty of Medicine, Transilvania University, Brasov, Romania*

## EPV‐0845

### The non‐motor symptom burden as an independent predictor of disease severity in Parkinson's disease: A cross‐sectional study from a Moldovan center

#### A. Arabadji ^1^; M. Gavriliuc^1^; I. Teacă^1^; E. Costru‐Tașnic^2^; M. Cebuc^1^; N. Rîbac^2^; O. Gavriliuc^2^

##### ^*1*^ *Neurology No 1, “Nicolae Testemitanu” State University of Medicine and Pharmacy, Chisinau, Moldova;* ^*2*^ *“Diomid Gherman” Institute of Neurology and Neurosurgery, Chisinau, Moldova*

## EPV‐0846

### The relationship between swallowing function, trunk control, and hand dexterity in patients with Parkinson's disease

#### N. Ünlüer ^1^; Y. Ateş Sari^2^; T. Özkan^3^; Y. Sücüllü Karadağ^4^

##### ^*1*^ *Health Sciences University, Gülhane Faculty of Physiotherapy and Rehabilitation, Ankara, Turkey;* ^*2*^ *Ankara Yıldırım Beyazıt University, Faculty of Health Sciences, Department of Physiotherapy and Rehabilitation, Ankara, Turkey;* ^*3*^ *Giresun University, Vocational School of Health Services, Therapy and Rehabilitation, Ankara, Turkey;* ^*4*^ *Health Science University, Ankara City Hospital, Department of Neurology, Ankara, Turkey*

## EPV‐0847

### The shape of sequence effect in Parkinson's disease

#### A. Grandolfo ^1^; L. Angelini^2^; M. De Riggi^1^; D. Birreci^1^; S. Aloisio^1^; G. Paparella^3^; A. Cannavacciuolo^2^; M. Bologna^1^

##### ^*1*^ *Department of Human Neurosciences, Sapienza University of Rome, Rome, Italy;* ^*2*^ *IRCCS Neuromed Pozzilli (IS), Italy;* ^*3*^ *Department of Neurology, University of Bari Aldo Moro, Bari, Italy*

## EPV‐0848

### Tips and strategies for subthalamic nucleus aDBS programming in Parkinson's disease

#### Á. Camero Piñatel ^1^; M. Molina Haro^1^; I. Del Pino Díaz^2^; C. Madrid Navarro^3^; L. Triguero Cueva^3^; J. Romero Fábrega^3^; M. Pérez Navarro^1^; M. Katati^4^; A. Mínguez Castellanos^3^; F. Escamilla Sevilla^3^

##### ^*1*^ *Neurology Service, Hospital Universitario Virgen de las Nieves, Granada;* ^*2*^ *Neurology Service, Hospital Universitario Clínico San Cecilio, Granada;* ^*3*^ *Neurology Service, Hospital Universitario Virgen de las Nieves, Instituto de Investigación Biomédica ibs.GRANADA, Granada;* ^*4*^ *Servicio de Neurocirugía, Hospital Universitario Virgen de las Nieves, Granada*

## EPV‐0849

### Twelve‐year longitudinal assessment of cognitive and motor function in Parkinson's disease versus healthy controls

#### X. Choi ^1^; D. Heng^1^; S. Ng^2^; H. Lim^2^; E. Lim^1^; P. Manharlal^1^; L. Tan^2^; E. Tan^1^

##### ^*1*^ *Department of Neurology, National Neuroscience Institute (Singapore General Hospital Campus), Singapore;* ^*2*^ *Department of Neurology, National Neuroscience Institute (Tan Tock Seng Hospital Campus), Singapore*

## EPV‐0850

### Ultrasound‐guided botulinum toxin injections for upper limb tremor: A portuguese single‐center case series

#### S. Moreira; A. Costa; G. Carneiro; S. Varanda; S. Lopes

##### Neurology Department, Unidade Local de Saúde Braga, Braga, Portugal

## EPV‐0851

### Unilateral spinal‐generated pseudo‐dystonia due to chronic cervical cord compression: A case report

#### M. Guida; R. De Micco; M. Vomero; O. Ciaramaglia; C. Cabato; F. Ambrosio; M. Cirillo; A. Tessitore

##### Department of Advanced Medical and Surgical Sciences, University of Campania “Luigi Vanvitelli”, Naples, Italy

## EPV‐0852

### Unmet needs in movement disorders: Similarities and differences in European neurologists perspectives on essential tremor and Parkinson's disease

#### A. Sanchez Fraga ^1^; C. Tengelin^1^; N. Kaempf^1^; P. Crivelli^1^; K. Gant^2^; C. Ferrer^1^; A. Grinspan^2^

##### ^*1*^ *Insightec Europe GmbH, Munich, Germany;* ^*2*^ *Insightec. Inc, Miami, USA*

## EPV‐0853

### Unsupervised phenotyping of beta bursts in Percept RC recordings: A machine learning approach to non‐stationary Parkinson's disease biomarkers

#### P. Melo ^1^; E. Carvalho^1^; A. Oliveira^2^; R. Peres^2^; C. Soares^3^; M. Ferreira‐Pinto^4^; P. Aguiar^5^

##### ^*1*^ *Neuroengineering and Computational Neuroscience, i3S – Institute for Research and Innovation in Health*, *Porto, Portugal; Instituto de Ciências Biomédicas Abel Salazar (ICBAS), University of Porto, Porto, Portugal;* ^*2*^ *Neuroengineering and Computational Neuroscience, i3S – Institute for Research and Innovation in Health, Porto, Portugal; Faculty of Engineering (FEUP), University of Porto, Porto, Portugal;* ^*3*^ *Department of Clinical Neurosciences and Mental Health, RISE‐Health, Faculty for Medicine, University of Porto, Porto, Portugal; Department of Neurology, ULS São João, Porto, Portugal;* ^*4*^ *Department of Surgery and Physiology, RISE‐Health, Faculty for Medicine, University of Porto, Porto, Portugal; Department of Neurosurgery, ULS São João, Porto, Portugal;* ^*5*^ *Neuroengineering and Computational Neuroscience, i3S – Institute for Research and Innovation in Health, Porto, Portugal; Faculty of Medicine (FMUP), University of Porto, Porto, Portugal*

## EPV‐0854

### Use of “Kneu Health” app for Parkinson's phenotype stratification to improve patients’ selection for advanced therapies

#### T. Diven ^1^; S. Lane^2^; K. Muhammed^3^; K. Muhammed^4^; L. Agley^3^; R. Ellis^1^; J. Panicker^1^; J. Osman‐Farah^1^; A. Macerollo^1^

##### ^*1*^ *The Walton Centre NHS Foundation Trust for Neurology and Neurosurgery, Liverpool, UK;* ^*2*^ *Institute of Data Health Sciences, University of Liverpool, UK;* ^*3*^ *Kneu Health Company, London, UK;* ^*4*^ *Oxford University Hospitals, London, UK*

## EPV‐0855

### Validation of a smart shoe system for remote gait monitoring in people with Parkinson's disease

#### S. Maddumage Dona ^1^; K. Sullivan^1^; A. Lehn^2^; N. Kalyani^3^; G. Kerr^1^

##### ^*1*^ *Faculty of Health, Queensland University of Technology, Brisbane, Australia;* ^*2*^ *Princess Alexandra Hospital, Brisbane, Australia;* ^*3*^ *School of Health and Medical Sciences, University of Southern Queensland, Toowoomba, Australia*

## EPV‐0856

### Vascular risk factors as modifiers of Parkinson's disease progression

#### E. Basha; O. Taka; E. Ranxha; O. Cibuku; R. Uruci

##### Neuroscience Department, Neurovascular Service, UHC “Mother Theresa”, Tirana, Albania

## EPV‐0857

### Vertebral artery dissection associated with tourette syndrome: Case report

#### J. De Obaldía ^1^; C. Kadosh^1^; J. Naftali^1^; E. Auriel^1^; J. Lukman^3^; G. Raphaeli^2^

##### ^*1*^ *Neurology Department, Rabin Medical Center, Petach Tikva, Israel;* ^*2*^ *Interventional Neuroradiology Unit, Rabin Medical Center, Petach Tikva, Israel;* ^*3*^ *Radiology Department, Rabin Medical Center, Petach Tikva, Israel*

## EPV‐0858

### VPS16‐associated generalized dystonia misdiagnosed as wilson disease: A diagnostic pitfall

#### I. Alexandratou ^1^; C. Koros^1^; K. Kournis^1^; A. Simitsi^1^; I. Alefanti^1^; N. Papagiannakis^1^; M. Kouvli^1^; M. Marouda^1^; Fitokis^1^; A. Alexopoulou^2^; L. Stefanis^1^

##### ^*1*^ *First Department of Neurology of the National and Kapodistrian University of Athens (NKUA) Medical School at Eginition Hospital, Athens, Greece;* ^*2*^ *Hepatology Unit, Ippokrateio General Hospital, Athens, Greece*

## EPV‐0859

### When infection imitates degenerative movement disorders: A diagnostic trap in late neuroborreliosis

#### M. Kubjatkova; S. Mareček; J. Mašková; P. Havránková; H. Brozova

##### Department of Neurology and Center of Clinical Neuroscience, 1st Faculty of Medicine and General University Hospital in Prague, Czech Republic

## MS and related disorders

## EPV‐0860

### Correlations between coroid plex volume, EPVS and MRI markers of MS pathology

#### A. Esposito ^1^; L. Crosaro^1^; L. Mollo^1^; C. Siniscalchi^1^; F. Falco^1^; G. Corsini^1^; M. Aiello^2^; A. De Rosa^3^; D. Ranucci^1^; V. Nicolella^1^; A. Castiello^1^; S. Cocozza^4^; F. Briganti^4^; M. Moccia^5^; R. Iodice^1^; V. Brescia Morra^1^; R. Lanzillo^1^; M. Petracca^6^; A. Carotenuto^1^

##### ^*1*^ *Department of Neurosciences, Reproductive and Odontostomatological Sciences, Federico II University, Naples, Italy;* ^*2*^ *IRCCS SYNLAB SDN, Naples, Italy;* ^*3*^ *Advanced MRI Neuroimaging Centre, Department of Advanced Medical and Surgical Sciences, University of Campania “Luigi Vanvitelli”, Naples, Italy;* ^*4*^ *Unit of Neuroradiology, Department of Advanced Biomedical Sciences, Federico II University of Naples, Naples, Italy;* ^*5*^ *Department of Molecular Medicine and Medical Biotechnology, Federico II University of Naples, Italy;* ^*6*^ *Department of Human Neurosciences, Sapienza University, Rome, Italy*

## EPV‐0861

### “Shrimp Sign”: A peculiar imaging marker with life‐threatening impact

#### G. Medeiros Andrade Figueira; P. Vallegas Soares; R. Custodio Silveira; V. Tavares Carvalho Crelier; A. Seide Cardoso Vidal; M. Nunes Ferreirinha Leite de Castro; F. Tome do Nascimento; F. Faria Andrade Figueira

##### Department of Neurology, Hospital São Francisco na Providência de Deus, Rio de Janeiro, Brazil

## EPV‐0862

### A single‐centre retrospective review of autologous hematopoietic stem cell transplantation for multiple sclerosis in Singapore

#### S. Abu Hassan ^1^; P. Ratnagopal^1^; W. Foong^1^; T. Yeo^1^; K. Tan^1^; W. Hwang^2^; A. Ho^2^; F. Lim^2^; K. Yong^1^

##### ^*1*^ *National Neuroscience Institute, Singapore;* ^*2*^ *Department of Haematology, Singapore General Hospital, Singapore*

## EPV‐0863

### Access to immunomodulatory treatment for patients with multiple sclerosis in the Republic of Moldova: A mixed‐methods study

#### A. Belenciuc ^1^; A. Grumeza^1^; O. Odainic^1^; V. Lisnic^2^

##### ^*1*^ *Department of Neurology, Institute of Neurology and Neurosurgery “Diomid Gherman”, Chișinău, Moldova;* ^*2*^ *Department of Neurology, “Nicolae Testemitanu”, State University of Medicine and Pharmacy, Chișinău, Moldova*

## EPV‐0864

### AI‐driven MRI measures for predicting NEDA failure in multiple sclerosis

#### T. Schaffartziková ^1^; S. Kozakova^1^; N. Brodova^1^; P. Hanzlikova^2^; J. Havelka^3^; P. Kusnierova^4^; J. Vermirovska^1^; Z. Zipsova^1^; D. Vilimek^3^; P. Hradilek^1^; O. Volny^1^; K. Zondra Revendova^1^

##### ^*1*^ *Department of Neurology and Department of Clinical Neurosciences, Faculty of Medicine, University of Ostrava, University Hospital Ostrava, Ostrava, Czech Republic;* ^*2*^ *Department of Radiology;* ^*2*^ *nd Faculty of Medicine, Charles University and Motol University Hospital, Prague, Czech Republic;* ^*3*^ *Department of Radiology, University Hospital Ostrava, Ostrava, Czech Republic;* ^*4*^ *Institute of Laboratory Medicine and Department of Laboratory Medicine, Faculty of Medicine, University Hospital Ostrava, Ostrava, Czech Republic*

## EPV‐0865

### Amantadine for multiple sclerosis‐related fatigue: A systematic review and meta‐analysis

#### A. Mostafa Amin^1^; M. Kashbor^2^; A. Fawzy Alsokery^1^; A. Nady Ibrahim^1^; M. Wagdy^3^; A. ElSayed Awadallah^1^; A. Reda Aboyadak^1^; M. Mahmoud Hassan Meshref ^4^

##### ^*1*^ *Faculty of Medicine, Al‐Azhar University, Cairo, Egypt;* ^*2*^ *Diagnostic Radiology Department, National Cancer Institute, Misrata, Libya;* ^*3*^ *Faculty of Medicine Modern University for Information and Technology, Cairo, Egypt;* ^*4*^ *Department of Neurology, Faculty of Medicine, Al‐Azhar University, Cairo, Egypt*

## EPV‐0866

### Analytical comparability of SIMOA and NULISA platforms for CSF GFAP and NFL in treatment‐naïve multiple sclerosis

#### V. Cristillo ^1^; A. Pilotto^1^; C. Tolassi^2^; I. Girotto^2^; M. Bertoni^2^; T. Merati^1^; A. Rondina^3^; A. Padovani^1^

##### ^*1*^ *Neurology Unit, Department of Clinical and Experimental Sciences, University of Brescia, Italy;* ^*2*^ *Neurobiorepository and Laboratory of Advanced Biological Markers, University of Brescia and ASST Spedali Civili Hospital, Brescia, Italy;* ^*3*^ *Department of Clinical and Experimental Sciences, University of Brescia, Italy*

## EPV‐0867

### Anti‐CD20 treatment induced neutropenia: A retrospective case series

#### S. Missio ^1^; E. Saccomano^1^; S. Lorenzut^2^; A. Nilo^1^; S. Pez^1^; D. Cutuli^2^; D. Cargnelutti^2^; M. Calligaris^3^; M. Valente^1^

##### ^*1*^ *Clinical Neurology, Department of Medicine (DMED), University of Udine, Udine;* ^*2*^ *Neurology Unit, S. Maria della Misericordia University Hospital, Udine;* ^*3*^ *University of Udine, Department of Medicine (DMED), Udine*

## EPV‐0868

### Anticipatory anxiety before follow‐up visits in early‐stage relapsing‐remitting multiple sclerosis: Prevalence, predictors, and clinical implications

#### A. Pérez‐Sempere^1^; E. Garcia Arcelay ^2^; J. Caruncho^3^; A. Candeliere‐Merlicco;^4^; A. Orviz^5^; J. Martín‐Martínez;^6^; R. Piñar^7^; E. Álvarez‐Rodríguez^8^; E. Pacheco^9^; L. Borrega^10^; I. Casanova^11^; A. Caminero^12^; J. Sánchez‐Menoyo^13^; M. Gómez‐Gutiérrez;^14^; O. Carmona^15^; C. Calles^16^; M. Hernández^17^; P. López‐Muñoz^18^; J. Mauriño^2^; I. González‐Suárez^8^

##### ^*1*^ *Department of Neurology, Hospital Universitario General de Alicante, Alicante, Spain;* ^*2*^ *Medical Department, Roche Farma, Madrid, Spain.;* ^*3*^ *AVEMPO (Asociación Viguesa de Esclerosis Múltiple de Pontevedra), Vigo, Spain;* ^*4*^ *Department of Neurology, Hospital Rafael Méndez, Lorca, Spain;* ^*5*^ *Department of Neurology, Hospital Universitario Fundación Jiménez Díaz, Madrid, Spain;* ^*6*^ *Department of Neurology, Hospital Universitario Miguel Servet, Zaragoza, Spain;* ^*7*^ *Department of Neurology, Hospital Universitario Clínico San Cecilio, Granada, Spain;* ^*8*^ *Department of Neurology, Hospital Universitario Álvaro Cunqueiro, Vigo, Spain;* ^*9*^ *Department of Neurology, Hospital Universitario Juan Ramón Jiménez, Huelva, Spain;* ^*10*^ *Department of Neurology, Hospital Universitario Fundación Alcorcón, Madrid, Spain;* ^*11*^ *Department of Neurology, Hospital Universitario de Torrejón, Madrid, Spain;* ^*12*^ *Department of Neurology, Complejo Asistencial de Ávila, Ávila, Spain;* ^*13*^ *Department of neurology, Galdakao‐Usansolo University Hospital, Osakidetza (Basque Health Service), Usansolo, (Bizkaia), Spain Biobizkaia Health Research Institute;* ^*14*^ *Department of Neurology, Hospital San Pedro de Alcántara, Cáceres, Spain;* ^*15*^ *Department of Neurology, Fundació Salut Empordà, Figueres, Spain;* ^*16*^ *Department of Neurology, Hospital Universitari Son Espases, Palma, Spain;* ^*17*^ *Department of Neurology, Hospital Nuestra Señora de la Candelaria, Tenerife, Spain;* ^*18*^ *Department of Neurology, Hospital Llíria Arnau, Llíria, Spain*

## EPV‐0869

### Applicability of NfL and GFAP as prognostic biomarkers in multiple sclerosis: A prospective study

#### P. López‐Grueiro Valcarce; L. Pulido Fraiz; A. García Leal; G. Torres Iglesias; M. Fernández‐Fournier Fernández; B. Chamorro Hernández; L. Otero Ortega; I. Puertas Muñoz

##### Neurology, Hospital Universitario La Paz, Madrid, Spain

## EPV‐0870

### Assessing the effects of induced fatigability on cognition and balance performance in Multiple Sclerosis: A multicenter cross‐sectional study

#### C. Solaro ^1^; R. Cardini^2^; R. Bertoni^2^; I. Carpinella^2^; C. Pegorini^2^; A. Tacchino^3^; E. Grange^3^; V. Guidotti^3^; A. Gallo^4^; T. Budassi^1^; T. Lanfranco^1^; E. Sbragia^1^; G. Brichetto^2^; M. Rovaris^2^; D. Cattaneo^2^; E. Gervasoni^2^

##### ^*1*^ *Neurology Unit Galliera Hospital, Genova, Italy;* ^*2*^ *IRCCS Fondazione Don Carlo Gnocchi, Milan, Italy;* ^*3*^ *NeuroBRITE Research Center, Italian Multiple Sclerosis Foundation (FISM), Italy;* ^*4*^ *AISM Rehabilitation Service of Como, Como, Italy*

## EPV‐0871

### Assessment of pain and quality of life in multiple sclerosis, neuromyelitis optica spectrum diseases, and MOG antibody associated disease

#### F. Balcı ^1^; B. İncirci^2^; S. Gok^2^

##### ^*1*^ *University of Health Sciences, Hamidiye Faculty of Medicine, Neurology, Istanbul, Türkiye;* ^*2*^ *Haseki Education and Research Hospital, Department of Neurology, İstanbul, Türkiye*

## EPV‐0872

### Assessment of tolerability of relapse treatment in people with Multiple Sclerosis using oral or intravenous methylprednisolone

#### J. Almada Silva ^1^; S. Delgado^1^; A. Rêgo^1^; R. Cunha^2^; A. Fontainhas^2^; I. Mendes^3^; M. Santos^1^; L. Leitão^1^

##### ^*1*^ *Neurology Department of Hospital Professor Doutor Fernando Fonseca, EPE, Unidade Local de Saúde Amadora/Sintra, Portugal;* ^*2*^ *General Day Hospital of Hospital Professor Doutor Fernando Fonseca, EPE, Unidade Local de Saúde Amadora/Sintra, Portugal;* ^*3*^ *Pharmacy Department of Hospital Professor Doutor Fernando Fonseca, EPE, Unidade Local de Saúde Amadora/Sintra, Portugal*

## EPV‐0873

### Association of psychological resilience, and quality of life with brain and lesion volumes in patients with early diagnosis of multiple sclerosis

#### A. Filosa^1^; R. Orlandi^1^; F. Bonomi^1^; F. Gobbin^2^; V. Dionisi^3^; S. Poli^3^; M. Rimondini^3^; A. Gajofatto ^1^

##### ^*1*^ *Department of Neuroscience, Biomedicine and Movement Science, Section of Neurology, University of Verona, Italy;* ^*2*^ *Unit of Neurology, Arzignano Hospital, Vicenza, Italy;* ^*3*^ *Department of Neuroscience, Biomedicine and Movement Science, Section of Clinical Psychology, University of Verona, Italy*

## EPV‐0874

### Associations between visual evoked potentials and MRI lesion load in relapsing‐remitting multiple sclerosis

#### N. Morawiec; B. Gębka‐Kępińska; J. Jurkiewicz; D. Gębka; J. Bączyk; M. Adamczyk‐Sowa

##### Department of Neurology, Faculty of Medical Sciences in Zabrze, Medical University of Silesia in Katowice, Zabrze, Poland

## EPV‐0875

### Autologous haematopoietic stem cell transplantation affects leptomeningeal enhancement in multiple sclerosis: A longitudinal study

#### A. Schmid ^1^; A. Mariottini^1^; A. Ginestroni^3^; E. Simonetti^4^; E. Carlesi^3^; F. Di Pasquale^3^; G. Costantini^1^; L. Marchi^1^; A. Repice^1^; S. Chiti^5^; A. Poggesi^1^; E. Fainardi^3^; C. Nozzoli^4^; I. Cutini^4^; L. Massacesi^1^

##### ^*1*^ *Department of Neurosciences, Psychology, Drug Research and Child Health, University of Florence, Florence, Italy;* ^*3*^ *Neuroradiology Unit, Careggi University Hospital, Florence, Italy;* ^*4*^ *Haematology, Careggi University Hospital, Florence, Italy;* ^*5*^ *Health Physics Unit, Careggi University Hospital, Florence, Italy*

## EPV‐0876

### Autologous hematopoietic stem cell transplantation versus alemtuzumab in multiple sclerosis: A systematic review and meta‐analysis

#### N. Kebir ^1^; S. Waleed^2^; Y. Akram EL Sayed^3^; H. Ayman^4^; T. Aziz El‐Ramly^5^; M. E. Ali^6^; E. S. Moubarak^7^; O. Fayez^8^

##### ^*1*^ *Faculty of Medicine, Oran 1 University, Algeria;* ^*2*^ *Alexandria Faculty of Medicine, Alexandria, Egypt;* ^*3*^ *Faculty of Medicine, Benha University, El qalyubia, Egypt;* ^*4*^ *Faculty of Medicine, Al Azhar University, El Qalubiah, Egypt;* ^*5*^ *Faculty of Medicine, Alexandria University, Alexandria, Egypt;* ^*6*^ *Faculty of Medicine, Tanta University, Tanta, Gharbia, Egypt;* ^*7*^ *Faculty of Medicine, Cairo University, Cairo, Egypt;* ^*8*^ *Faculty of Medicine, Al‐Azhar University, Cairo, Egypt*

## EPV‐0877

### Blood–cerebrospinal fluid barrier permeability and disability outcomes in multiple sclerosis: A real‐world cohort study

#### H. Manazoğlu ^2^; T. Ayaz^1^; A. Emekli^1^; T. Gündüz^1^; M. Kürtüncü^1^

##### ^*1*^ *Department of Neurology, Istanbul Faculty of Medicine, Istanbul University, Istanbul, Türkiye;* ^*2*^ *Department of Neurology, Ümraniye Training and Research Hospital, Istanbul, Türkiye*

## EPV‐0878

### Cerebellar lesions and epileptic seizures as unusual manifestations of AQP4‐IgG‐Positive NMOSD

#### H. Bendjemil

##### Medicine Department, Benamor Djilani Hospital, Eloued, Algeria

## EPV‐0879

### Challenges and treatment gap of multiple sclerosis in Sudan: A cross‐sectional study

#### M. Abdalla

##### Faculty of Medicine Ain Shams University, Cairo, Egypt

## EPV‐0880

### Characterizing the natalizumab wearing‐off phenomenon: Real‐world evidence from a clinically stable multiple sclerosis population

#### S. Alizada ^1^; U. Samadzade^2^; S. Cevik^3^; C. Caliskan^2^; S. Ozakbas^2^

##### ^*1*^ *Dokuz Eylul University, Department of Neurology, Turkey;* ^*2*^ *Izmir University of Economics, Neurology Clinic, Turkey;* ^*3*^ *Dokuz Eylul University, Department of Neurosciences, Turkey*

## EPV‐0881

### ChatMS study – Awareness and use of Large Language Models among people with multiple sclerosis: a large Italian survey

#### I. Schiavetti ^1^; L. Lavorgna^2^; R. Fantozzi^3^; S. Di Lemme^3^; F. Marinelli^4^; M. Tartaglia^4^; E. Cocco^5^; J. Frau^5^; L. Lorefice^6^; R. Lanzillo^7^; C. Cordioli^8^; R. Iodice^7^; M. Stromillo^9^; F. Sangalli^10^; M. Di Gregorio^11^; E. Signoriello^12^; S. Bonavita^12^; A. Gallo^2^; M. Pasca^13^; A. Sartori^14^; G. Sibilia^15^; M. Sormani^1^; A. Signori^1^

##### ^*1*^ *Section of Biostatistics, Department of Health Sciences, University of Genoa, Genoa, Italy/IRCCS Ospedale Policlinico San Martino, Genoa, Italy;* ^*2*^ *Department of Advanced Medical and Surgical Sciences, University of Campania Luigi Vanvitelli, Naples, Italy;* ^*3*^ *Department of Neurology, IRCCS Neuromed, Pozzilli (IS), Italy;* ^*4*^ *Neurology Unit‐MS Center, Fabrizio Spaziani Hospital, ASL Frosinone, Frosinone, Italy;* ^*5*^ *Multiple Sclerosis Center, University of Cagliari, Department of Medical Sciences and Public Health, Cagliari, Italy;* ^*6*^ *Multiple Sclerosis Center, Binaghi Hospital, ASL Cagliari, Cagliari, Italy;* ^*7*^ *Department of Neurosciences, Reproductive Sciences and Odontostomatology, University of Naples Federico II, Naples, Italy;* ^*8*^ *ASST Spedali Civili di Brescia, Multiple Sclerosis Center, Montichiari, Italy;* ^*9*^ *Department of Medicine, Surgery and Neuroscience, University of Siena, Siena, Italy;* ^*10*^ *Centro ad Alta Specializzazione per la diagnosi e la cura della Sclerosi Multipla, Ospedale Valduce, Como, Italy;* ^*11*^ *Neurology Unit, University of Salerno, Salerno, Italy;* ^*12*^ *Multiple Sclerosis Center, Second Division of Neurology, University of Campania “Luigi Vanvitelli”, Naples, Italy;* ^*13*^ *Complex Operative Unit of Neurology, “F. Ferrari” Hospital, Casarano;* ^*7*^ *3042 Lecce, Italy;* ^*14*^ *Neurology Unit, Hospital Care Department of Medicine, Azienda Sanitaria Universitaria Giuliano Isontina, Trieste, Italy;* ^*15*^ *Department of Neurology and MS Center, San Paolo Hospital, Naples*

## EPV‐0882

### Chronic pain in multiple sclerosis: Mechanisms, clinical characteristics and treatment strategies

#### P. Gklinos; G. Christodoulou; D. Pournara; E. Evangelopoulos; D. Mitsikostas

##### 1st Department of Neurology, Eginition University Hospital, National and Kapodistrian University of Athens, Athens, Greece

## EPV‐0883

### Cladribine as an effective and safe alternative in the treatment of relapsing–remitting multiple sclerosis

#### C. Martinez‐Follana; I. Saurina; M. Marín; E. Campanya; M. Garcia‐Huguet; A. Anaya; E. Vadillo; A. Gifreu; J. Gutiérrez; A. Boix; G. Alvarez

##### Neurology Department, Dr Josep Trueta Hospital‐Santa Caterina Hospital, Girona, Spain

## EPV‐0884

### Clinical course and multimodal imaging findings in MOG‐antibody‐associated optic neuritis

#### D. Eliseeva ^1^; A. Belkina^2^; N. Venediktova^3^; V. Bryukhov^4^; A. Burmak^5^; A. Shabalina^5^; N. Sheremet^3^; M. Zakharova^1^

##### ^*1*^ *6th Neurological department, Institute of Clinical and Preventive Neurology, Russian Center of Neurology and Neurosciences, Moscow, Russian Federation;* ^*2*^ *Outpatient department, Russian Center of Neurology and Neurosciences, Moscow, Russian Federation;* ^*3*^ *Federal State Scientific Institution M.M.Krasnov Research Institute of Eye Diseases, Moscow, Russian Federation;* ^*4*^ *Radiology Department, Russian Center of Neurology and Neurosciences, Moscow, Russian Federation;* ^*5*^ *Laboratory, Russian Center of Neurology and Neurosciences, Moscow, Russian Federation*

## EPV‐0885

### Clinical phenotype–based evaluation of serum kappa free light chains in relapsing: Remitting multiple sclerosis

#### S. Mert ^1^; H. Ozturk Emre^2^; E. Demir^1^; K. Cetin^1^; I. Acir^3^

##### ^*1*^ *Department of Neurology, Basaksehir Cam and Sakura City Hospital, Istanbul, Türkiye;* ^*2*^ *Department of Medical Biochemistry, Basaksehir Cam and Sakura City Hospital, Istanbul, Türkiye;* ^*3*^ *Department of Neurology, Bakırkoy Dr. Sadi Konuk Training & Research Hospital, Istanbul, Türkiye*

## EPV‐0886

### Clinical significance of neurotrophic and inflammatory CSF biomarkers in multiple sclerosis and neuromyelitis optica spectrum disorder

#### H. Soytürk^1^; Ş. Aydın Türkoğlu^2^; P. Bakkal ^2^; C. Önal^3^; S. Yıldız^2^

##### ^*1*^ *Interdisciplinary PhD Program in Neuroscience, Bolu Abant İzzet Baysal University, Bolu, Türkiye;* ^*2*^ *Department of Neurology, Bolu Abant İzzet Baysal University Faculty of Medicine, Bolu, Türkiye;* ^*3*^ *Department of Molecular Biology and Genetics, Faculty of Science, Zonguldak Bulent Ecevit University, Zonguldak, Türkiye*

## EPV‐0887

### CNS demyelination following adalimumab treatment for hidradenitis suppurativa: A single‐center experience

#### N. Konstantinidou; D. Digkas; D. Parisis; T. Afrantou; N. Grigoriadis; P. Ioannidis

##### 2nd Department of Neurology, AHEPA University Hospital of Thessaloniki, Aristotle University of Thessaloniki, Greece

## EPV‐0888

### Cognition in multiple sclerosis: Determined by age and education rather than sleep quality or insomnia

#### C. Alis; G. Genc; S. Bulut

##### Department of Neurology, Sisli Hamidiye Etfal Training and Research Hospital, Istanbul, Türkiye

## EPV‐0889

### Comparative viral antibody signatures under anti‐CD20 therapy in MS: Ocrelizumab vs ofatumumab

#### N. Morawiec; B. Adamczyk; K. Kubicka‐Bączyk; J. Jurkiewicz; M. Adamczyk‐Sowa

##### Department of Neurology, Faculty of Medical Sciences in Zabrze, Medical University of Silesia in Katowice, Zabrze, Poland

## EPV‐0890

### Comparison of clinical and demographic characteristics of on‐treatment MS patients between two MS center in Denmark and Romania

#### O. Vrînceanu; L. Pontieri; R. Balasa; M. Magyari

##### Doctoral School, “George Emil Palade” University of Medicine, Pharmacy, Science, and Technology of Tar‐gu Mures, Targu Mures, Romania

## EPV‐0891

### Comparison of fatigue and restless legs syndrome severity across NMOSD, MOGAD, and multiple sclerosis

#### A. Baskin; F. Saridas; E. Koc

##### Department of Neurology, Uludağ University Faculty of Medicine, Bursa, Türkiye

## EPV‐0892

### Comparison of STRATIFY JCV and IMMUNOWELL JCV assays for anti‐JCV antibody detection: A multicenter study across Tuscan neurology centers

#### A. Dini ^1^; B. Giovannini^1^; M. Amato^2^; A. Amidei^3^; M. Bartolozzi^4^; B. Calchetti^5^; N. De Stefano^6^; M. Di Cristinzi^7^; M. Falcini^8^; C. Fioretti^9^; M. Giannini^8^; P. Maritato^10^; L. Massacesi^7^; G. Moscato^9^; K. Plewina^11^; E. Portaccio^2^; A. Repice^7^; M. Stromillo^6^; M. Ulivelli^6^; N. Porcino^1^; L. Pasquali^1^

##### ^*1*^ *Department of Clinical and Experimental Medicine, Neurology Unit, University of Pisa, Pisa, Italy;* ^*2*^ *Department of Neurofarba, University of Florence, Florence, Italy;* ^*3*^ *UOS Neurologia Cecina Piombino Elba, USL Toscana nord‐ovest, Cecina, Italy;* ^*4*^ *Neurology Unit, Hospital of Empoli, Empoli, Italy.;* ^*5*^ *Neurology Department, San Donato Hospital, Arezzo, Italy;* ^*6*^ *Department of Medicine, Surgery and Neuroscience, University of Siena, Siena, Italy;* ^*7*^ *Department of Neurology 2 and Tuscan Region Multiple Sclerosis Referral Centre, Careggi University Hospital, Florence, Italy;* ^*8*^ *Neurology Unit, Department of medical specialties, Azienda USL Toscana Centro, Prato, Italy.;* ^*9*^ *Neurology Unit, Livorno Hospital, Livorno, Italy;* ^*10*^ *Unit of Neurology, San Luca Hospital, Lucca, Italy;* ^*11*^ *Ospedale Misericordia, Neurology Unit, Grosseto, Italy. Study Group: Tuscan MS‐JCV Study Group*

## EPV‐0893

### Contribution of thalamus‐cortex network and cortical habituation mechanisms in multiple sclerosis

#### B. Pitzalis

##### Department of Human Neurosciences, Sapienza University of Rome, Rome, Italy

## EPV‐0894

### Cryoneurolyis of the tibial nerve as a treatment for spasticity in patients with multiple sclerosis

#### A. Hughes ^1^; P. Winston^2^; M. Hashemi^2^

##### ^*1*^ *Faculty of Medicine, University of British Columbia, Vancouver, Canada;* ^*2*^ *Vancouver Island Health Authority Department of Physical Medicine and Rehabilitation, Victoria, Canada*

## EPV‐0895

### De‐escalation of high efficacy disease‐modifying therapies in multiple sclerosis: A systemic review and meta‐analysis assessing relapse rate

#### R. Mohamed ^1^; K. Sarhan^2^; M. Shalabi^2^; M. Meshref^3^

##### ^*1*^ *Mansoura Manchester Program for Medical Education, Faculty of Medicine, Mansoura University, Mansoura, Egypt;* ^*2*^ *Faculty of Medicine, Mansoura University, Mansoura, Egypt;* ^*3*^ *Neurology Department, Faculty of Medicine, Alazhar University, Cairo, Egypt*

## EPV‐0896

### Determinants of resilience in Tunisian patients with multiple sclerosis

#### M. Becha; A. Souissi; N. Msadek; A. Gharbi; Y. Abida; A. Atrous; I. Kacem; A. Gargouri; R. Gouider

##### Neurology Department, LR18SP03 and Clinical Investigation Center Neurosciences and Mental Health – University Hospital Center Razi‐ Mannouba, Tunis, Tunisia – Faculty of Medicine of Tunis, Tunis El Manar University, Tunis, Tunisia

## EPV‐0897

### Development of pulmonary sarcoidosis in a patient with multiple sclerosis on sphingosine‐1‐phosphate modulator. A case report

#### M. Jeremić ^1^; M. Janković^1^; N. Momčilović^1^; M. Budimkić^1^; N. Veselinović^1^; O. Tamaš^1^; N. Trboljevac^2^; J. Drulović^1^; Š. Mesaroš^1^

##### ^*1*^ *Neurology Clinic, University Clinical Center of Serbia, Belgrade, Serbia;* ^*2*^ *Pulmonology Clinic, University Clinical Center of Serbia, Belgrade, Serbia*

## EPV‐0898

### Differentially expressed miRNAs in Multiple Sclerosis patients stratified for NEDA status

#### M. Missaglia ^1^; M. Sorosina^1^; A. Giordano^1^; F. Clarelli^1^; K. Misra^1^; E. Mascia^1^; P. Béatrice^3^; L. Roland^3^; M. Rocca^4^; M. Filippi^2^; F. Esposito^1^

##### ^*1*^ *IRCCS San Raffaele Scientific Institute, Laboratory of human genetics of neurological disorders, Milan, Italy;* ^*2*^ *IRCCS San Raffaele Scientific Institute, Neurology and Neurorehabilitation Unit, Milan, Italy;* ^*3*^ *CRC‐SEP, Neurosciences department, CHU Toulouse, France;* ^*4*^ *IRCCS San Raffaele Scientific Institute, Neuroimaging Research Unit, Division of Neuroscience, Milan, Italy*

## EPV‐0899

### Do obesity related hormones play a role in multiple sclerosis?

#### A. Sowa ^1^; K. Sawa^1^; N. Morawiec^1^; B. Adamczyk^1^; M. Rakoca^2^; J. Jurkiewicz^1^; S. Wawrzyniak^2^; M. Adamczyk‐Sowa^1^

##### ^*1*^ *Department of Neurology, Faculty of Medical Sciences in Zabrze, Medical University of Silesia in Katowice, Zabrze, Poland;* ^*2*^ *Department of Neurology;* ^*10*^ *th Military Research Hospital and Polyclinic, Bydgoszcz, Poland*

## EPV‐0900

### Early functional, cognitive, and neuropsychiatric outcomes in newly diagnosed multiple sclerosis: A two‐year longitudinal study

#### D. Cetinkaya Tezer; B. Tasdelen; S. Ocak; I. Gungor Dogan; S. Demir

##### Neurology Department, Sancaktepe Ilhan Varank Sancaktepe Sehit Prof. Dr. Ilhan Varank Training and Research Hospital, Istanbul, Türkiye

## EPV‐0901

### Effect of ofatumumab on brain atrophy and its association with clinical outcomes in relapsing‐remitting multiple sclerosis

#### A. Pogoda‐Wesołowska ^1^; I. Stachura^2^; S. Górka^1^; E. Gągała^2^; J. Brzostowski^2^; P. Szukało^1^; M. Kania‐Pudło^3^; J. Micek^4^; J. Ginter^2^; A. Stępień^1^

##### ^*1*^ *Neurology Clinic, Military Institute of Medicine – National Research Institute, Warsaw, Poland;* ^*2*^ *Faculty of Medicine, University of Warsaw, Warsaw, Poland;* ^*3*^ *Department of Medical Radiology, Military Institute of Medicine – National Research Institute, Warsaw, Poland;* ^*4*^ *Employee of Novartis Poland Sp. z o.o., Warsaw, Poland*

## EPV‐0902

### Effectiveness and safety of ofatumumab treatment in RRMS patients – One site RWE experience study

#### K. Sawa ^1^; A. Sowa^1^; K. Kubicka‐Bączyk^1^; N. Morawiec^1^; J. Bączyk^1^; J. Jurkiewicz^1^; E. Chełmecka^2^; M. Adamczyk‐Sowa^1^

##### ^*1*^ *Department of Neurology, Faculty of Medical Sciences in Zabrze, Medical University of Silesia in Katowice, Zabrze, Poland;* ^*2*^ *Department of Statistics, School of Pharmacy with Division of Laboratory Medicine, Medical University of Silesia, Sosnowiec, Poland*

## EPV‐0903

### Effectiveness of a dance‐based intervention on motor outcomes in young adults with multiple sclerosis: Results of the ESPRIMO study

#### F. Bressan; R. Orlandi; F. Bonomi; S. Poli; V. Dionisi; F. Gobbin; G. Giusto; A. Filosa; D. Rudi; M. Rimondini; A. Gajofatto

##### Department of Neuroscience, Biomedicine, and Movement Sciences, University of Verona, Verona, Italy

## EPV‐0904

### Effects of fingolimod exposure on birth weight and prematurity in Multiple Sclerosis

#### T. Aslan^1^; E. Kaya ^2^; Y. Simsek^3^; A. Ozdogar^4^; S. Ozcelik^5^; C. Caliskan^3^; S. Ozakbas^3^

##### ^*1*^ *Department of Clinical Neurosciences, Cumming School of Medicine, University of Calgary, Alberta, Canada;* ^*2*^ *Tepecik Training and Research Hospital, Izmir, Türkiye;* ^*3*^ *Izmir University of Economics, Medical Point Hospital, Izmir, Türkiye;* ^*4*^ *Department of Physiotherapy and Rehabilitation, Faculty of Health Sciences, Van Yüzüncü Yıl University, Van, Türkiye;* ^*5*^ *Tufts Medical Center, Department of Neurology, MA, USA*

## EPV‐0905

### Effects of oral disease‐modifying therapies on lymphocyte counts in relapsing‐remitting multiple sclerosis: A real‐world experience

#### P. Sėdžius; D. Musneckienė

##### Department of Neurology, Lithuanian University of Health Sciences, Kaunas, Lithuania

## EPV‐0906

### Efficacy, safety, and neurofilaments evaluation in secondary progressive multiple sclerosis patients treated with siponimod

#### F. Baldisseri ^1^; C. Lapucci^2^; C. Bason^2^; T. Vigo^2^; E. Cipriano^1^; G. Boffa^2^; E. Capello^2^; A. Laroni^1^; E. Sbragia^3^; M. Rilla^4^; P. Gazzola^5^; M. Inglese^2^; M. Cellerino^3^

##### ^*1*^ *Department of Neuroscience, Rehabilitation, Ophthalmology, Genetics, Maternal and Child Health (DINOGMI), University of Genoa, Genoa, Italy;* ^*2*^ *IRCCS Ospedale Policlinico San Martino, Genoa, Italy;* ^*3*^ *Neurologia Ospedali Galliera Genoa, Italy;* ^*4*^ *Neurologia ASL 1 Imperia, Italy;* ^*5*^ *Centro Sclerosi Multipla, ASL3 Genovese, Ospedale P.A. Micone, Genova, Italy*

## EPV‐0907

### EPVS in mid brain in multiple sclerosis correlation with cerebellar and brainsteam atrophy and clinical disability

#### L. Corsaro ^1^; A. Esposito^1^; L. Mollo^1^; C. Siniscalchi^1^; G. Corsini^1^; D. Ranucci^1^; A. Castiello^1^; V. Nicolella^1^; R. Iodice^1^; V. Brescia Morra^1^; R. Iodice^1^; M. Petracca^2^; S. Cocozza^3^; M. Aiello^4^; A. Canna^5^; F. Briganti^6^; M. Moccia^7^; R. Lanzillo^1^; A. Carotenuto^1^

##### ^*1*^ *Department of Neurosciences, Reproductive and Odontostomatological Sciences, Federico II University, Naples, Italy;* ^*2*^ *Department of Human Neurosciences, Sapienza University, Rome, Italy;* ^*3*^ *Departments of Advanced Biomedical Sciences and Electrical Engineering and Information Technology, University of Naples Federico II, Naples, Italy;* ^*4*^ *IRCCS SDN, Naples, Italy;* ^*5*^ *Department of Medicine, Surgery and Dentistry “Scuola Medica Salernitana”, University of Salerno, Baronissi Salerno, Italy;* ^*6*^ *Unit of Neuroradiology, Department of Advanced Biomedical Sciences, Federico II University of Naples, Naples, Italy;* ^*7*^ *Department of Molecular Medicine and Medical Biotechnology, Federico II University of Naples, Naples, Italy*

## EPV‐0908

### Erythrocyte morphological alterations in multiple sclerosis assessed by white‐light diffraction phase microscopy

#### A. Ünal ^1^; Ö. Kocahan^2^; B. Altunan^3^; F. Dilek^4^; Ç. Deniz^5^

##### ^*1*^ *Department of Neurology, Faculty of Medicine, Tekirdağ Namık kemal University, Tekirdağ, Türkiye;* ^*2*^ *Department of Physics,Faculty of Arts and Sciences, Tekirdag Namık Kemal University, Tekirdağ, Türkiye;* ^*3*^ *Department of Neurology, Faculty of Medicine, Tekirdağ Namık kemal University, Tekirdağ, Türkiye;* ^*4*^ *Elderly Care Program, Vocational School of Health, Tekirdağ Namık Kemal University, Tekirdağ, Türkiye;* ^*5*^ *Vocational School of Health Services, Department of Neurology, TC Demiroğlu Bilim University, İstanbul, Türkiye*

## EPV‐0909

### Dissociating cognitive and physical fatigue in multiple sclerosis: A differential roles of psychophysiological vs. neurological‐disease related factors

#### M. Heidari^1^; S. Nabavi ^2^; M. Akbarfahimi^1^; G. Taghizade^1^; L. Lajevardi^1^; S. Karimi^2^

##### ^*1*^ *Department of Occupational Therapy, School of Rehabilitation Science, Iran University of Medical Science, Tehran, Iran;* ^*2*^ *Department of Regenerative Biomedicine, Royan Institute for Stem Cell Biology and Technology, Royan, Iran, ACECR, Tehran, Iran*

## EPV‐0910

### Evaluation of undetectable K‐Flc index in suspected multiple sclerosis

#### F. Chiappetta ^1^; I. Addazio^1^; C. Ballerini^1^; M. Betti^1^; T. Biagioli^2^; A. Caporali^1^; E. De Meo^3^; S. Degl'Innocenti^1^; L. Pastò^4^; P. Purchiaroni^4^; V. Penati^1^; F. Romeo^1^; E. Portaccio^1^; M. Amato^1^

##### ^*1*^ *University of Florence, Department of NEUROFARBA, Florence, Italy;* ^*2*^ *Careggi Diagnostic Laboratory, Careggi University Hospital, Florence, Italy;* ^*3*^ *University College London (UCL) Queen Square Institute of Neurology, Queen Square Multiple Sclerosis Centre, Department of Neuroinflammation, NMR Research Unit, London, UK;* ^*4*^ *Careggi University Hospital, Florence, Italy*

## EPV‐0911

### Exploring the effect of cerebrospinal fluid cytokine levels on MRI biomarkers of inflammation and neurodegeneration in multiple sclerosis

#### I. Addazio ^1^; E. De Meo^1^; M. Betti^1^; C. Ballerini^1^; R. Amoriello^2^; O. Maghrebi^2^; C. Ballerini^2^; E. Fainardi^3^; E. Portaccio^1^; M. Amato^1^

##### ^*1*^ *Department of NEUROFARBA, University of Florence, Florence, Italy;* ^*2*^ *Laboratory of Neuroimmunology Department of Experimental and Clinical Medicine University of Florence, Florence, Italy;* ^*3*^ *Neuroradiology Unit, Careggi University Hospital, Florence, Italy*

## EPV‐0912

### Fatigue and depression in multiple sclerosis: A bidirectional relationship

#### A. Alhajmabrouk^1^; H. Derbali ^1^; I. Bedeoui^1^; Y. Mrad^1^; M. Messelmani^1^; M. Mansour^1^; N. Fakih Mrissa^2^; J. Zaoueli^1^

##### ^*1*^ *Neurology Department, The Military Hospital of Tunis, Tunis, Tunisia;* ^*2*^ *Molecular Biology Research Laboratory, The Military Hospital of Tunis, Tunis, Tunisia*

## EPV‐0913

### Fatigue and disability drive sexual dysfunction in multiple sclerosis: a cross‐sectional study in spain using the MSISQ‐15

#### C. Lapeña López^1^; E. Ginés Murcia ^1^; L. Moreno Navarro^1^; Á. Pérez Sempere^1^; G. Pérez Paz^2^; P. Mahiques Ochoa^1^; M. Warnken Miralles^1^; A. Benavent Rojas^1^; L. Montero Pardo^1^; D. López Ros^1^; A. Muñoz Ambit^1^; L. Ruiz‐Escribano Menchén^1^

##### ^*1*^ *Neurology, Hospital General Universitario de Alicante, Spain;* ^*2*^ *Preventive Medicine, Hospital General Universitario de Alicante, Spain*

## EPV‐0914

### Fatigue severity in multiple sclerosis: preliminary results of a cross‐sectional study

#### F. Sarıdaş; F. Hojjati; S. Albak; E. Koç

##### Bursa Uludağ University, Faculty of Medicine, Department of Neurology, Bursa, Türkiye

## EPV‐0915

### Fatigue, depression and anxiety as determinants of physical and mental quality of life in MS types

#### K. Ntoskas^1^; T. Doskas ^2^

##### ^*1*^ *Department of Neurology, 251 Hellenic Air Force General Hospital, Athens, Greece;* ^*2*^ *Department of Neurology, Athens Naval Hospital, Athens, Greece*

## EPV‐0916

### Feasibility and neurological safety of a ketogenic dietary intervention in relapsing‐remitting multiple sclerosis: A pilot study

#### F. Filippi ^1^; S. Lorenzut^2^; R. Garbo^3^; E. Lamon^4^; I. Del Negro^4^; A. Nilo^1^; S. Pez^4^; G. Gigli^4^; M. Valente^4^

##### ^*1*^ *Department of Medicine (DMED), University of Udine, Via Colugna 50, 33100 Udine, Italy;* ^*2*^ *Neurology Unit, Head Keck and Neuroscience Department, Santa Maria della Misericordia University Hospital, Udine, Italy;* ^*3*^ *SC Neurology of Gorizia Monfalcone, Azienda Sanitaria Universitaria Giuliano Isontina (ASUGI), Udine, Italy;* ^*4*^ *Clinical Neurology Unit, Udine University Hospital, Piazzale Santa Maria della Misericordia, Udine, Italy*

## EPV‐0917

### Features of cognitive and neuropsychological disorders in multiple sclerosis

#### M. Nazarova ^1^; R. Matmurodov^1^; S. Nazarov^2^

##### ^*1*^ *Department of Neurology and medical psychology, Tashkent State Medical University, Tashkent, Uzbekistan;* ^*2*^ *Department of Otorhinolaryngology, Tashkent State Medical University, Tashkent, Uzbekistan*

## EPV‐0918

### Frailty in elderly patients with Multiple Sclerosis (MS)

#### V. Lizhdvoy

##### Department of Neurology, 1Moscow Regional Research Clinical Institute N.A. M.F, Vladimirskiy, Moscow, Russian Federation

## EPV‐0919

### From biomarker to a new clinical perspective: Kappa free light chain index and disability progression in multiple sclerosis

#### M. Bruno; G. Romano; G. Lus; S. Bonavita; E. Signoriello

##### Multiple Sclerosis Centre, Second Division of Neurology, University of Campania “Luigi Vanvitelli”, Naples, Italy

## EPV‐0920

### Functional outcomes after immune reconstitution therapy: A multidimensional evaluation of multiple sclerosis patients treated with cladribine

#### S. Ocak; B. Tasdelen; D. Cetinkaya Tezer; I. Gungor Dogan; S. Demir

##### Neurology Department, Sancaktepe Ilhan Varank Sancaktepe Sehit Prof. Dr. Ilhan Varank Training and Research Hospital, Istanbul, Türkiye

## EPV‐0921

### Gastric diffuse large B‐cell lymphoma in a multiple sclerosis patient undergoing treatment with natalizumab: A case‐report and literature review

#### L. de Castro Rocha; H. Felgueiras

##### Serviço de Neurologia, Unidade Local de Saúde de Gaia e Espinho, Portugal

## EPV‐0922

### Gastrointestinal symptoms and health‐related quality of life in relapsing‐remitting multiple sclerosis: A GSRS and SF‐36 based study

#### S. Talapbek kyzy; M. Tardov; N. Sturov; T. Mansur; A. Mikhailov

##### Department of General Medicine, RUDN University, Moscow, Russian Federation

## EPV‐0923

### Hemifacial spasm as the initial manifestation of multiple sclerosis: a diagnostic pitfall

#### H. Hadji; S. Demuth; P. Sunsovan; M. Verin; C. Ciumas Gaumond

##### Neurology Unit, CHU d'Orleans, Orleans, France

## EPV‐0924

### Hidden disabilities in multiple sclerosis: Incontinence and sexual dysfunction

#### M. Seferoğlu ^1^; M. Köktürk^1^; A. Görgül^1^; A. Sıvacı^1^; M. Güzelsoy^2^

##### ^*1*^ *Bursa Yüksek İhtisas Training and Research Hospital, Neurology Clinic, Türkiye;* ^*2*^ *Bursa Yüksek İhtisas Training and Research Hospital, Urology Clinic, Türkiye*

## EPV‐0925

### How will climate change impact severity and incidence of multiple sclerosis?

#### S. Jalal; N. Nantheswaran; D. Neal; H. Rahman

##### Anglia Ruskin University, Cambridge, UK

## EPV‐0926

### Hyper‐reflecting foci in multiple sclerosis: Association with clinical, optical coherence tomography, and laboratory markers

#### A. Castiello ^1^; A. Esposito^1^; V. Nicolella^1^; G. Corsini^1^; L. Corsaro^1^; G. Cennamo^1^; C. Polito^3^; D. Terracciano^3^; D. Ranucci^1^; G. Moiese^1^; C. Costagliola^1^; M. Petracca^4^; R. Lanzillo^1^; M. Moccia^2^; V. Brescia Morra^2^; A. Carotenuto^1^

##### ^*1*^ *Department of Neurosciences, Reproductive Sciences and Odontostomatology, University of Naples Federico II, Naples, Italy;* ^*2*^ *Multiple Sclerosis Clinical Unit, Policlinico Federico II University Hospital, Naples, Italy;* ^*3*^ *Department of Translational Medical Sciences, Federico II University of Naples, Naples, Italy;* ^*4*^ *Department of Human Neurosciences, Sapienza University, Rome, Italy*

## EPV‐0927

### Immunobiological and gender‐specific features of multiple sclerosis in patients of Uzbek ethnicity

#### S. Khudayarova; G. Rakhmatullayeva; A. Kadirova

##### Tashkent State Medical University, Tashkent, Uzbekistan

## EPV‐0928

### Immunoglobulin profiles in multiple sclerosis: A real‐world comparison of ofatumumab and ocrelizumab

#### M. Sredanović; K. Tešija; I. Adamec; T. Gabelić; B. Barun; I. Martinez; M. Krbot Skorić; M. Habek

##### University Hospital Centre Zagreb, Croatia

## EPV‐0929

### Immunological and hormonal shifts in pregnant women with multiple sclerosis and their impact on pregnancy outcomes

#### Y. Parpiyeva; H. Halimova; N. Rashidova; H. Yuldoshev; A. Dall; B. Tolipova

##### ^*1*^ *Tashkent State Medical University, Tashkent, Uzbekistan*

## EPV‐0930

### Impact of Aquaporin‐4 serostatus on disease activity and disability in NMOSD: A five‐year comparative study

#### S. Ozakbas ^1^; E. Kaya^2^; Y. Simsek^1^

##### ^*1*^ *Izmir University of Economics, Medical Point Hospital, Izmır, Türkiye;* ^*2*^ *Department of Neurology, University of Health Science, Tepecik Training and Research Hospital, Izmir, Türkiye*

## EPV‐0931

### Increasing age is associated with lesser decreases in neurofilament levels under disease‐modifying treatments for MS

#### E. Sfakianakis; I. Tzvetanova; T. Lytras; D. Papadopoulos

##### School of Medicine, European University Cyprus, Nicosia, Cyprus

## EPV‐0932

### Investigation of functional capacity, reaction time, and cognitive functions in individuals with multiple sclerosis

#### E. İpek Halatcı ^1^; A. Erkoç^2^; U. Şimşek^3^; N. Ünlüer^4^

##### ^*1*^ *Ankara Yıldırım Beyazıt University, Vocational School of Health Services, Department of Therapy and Rehabilitation, Disabled Care and Rehabilitation Program, Ankara, Türkiye;* ^*2*^ *University of Health Sciences, Gülhane Institute of Health Sciences, Department of Physiotherapy and Rehabilitation, Ankara, Türkiye;* ^*3*^ *Department of Neurology, University of Health Sciences, Gulhane Training and Research Hospital, Ankara, Türkiye;* ^*4*^ *University of Health Sciences, Gülhane Faculty of Physiotherapy and Rehabilitation, Department of Neurological Physiotherapy and Rehabilitation, Ankara, Türkiye*

## EPV‐0933

### Is it safe to stop? A systematic review and meta‐analysis of discontinuation versus continuation of disease‐modifying therapies in multiple sclerosis

#### K. Sarhan ^1^; R. Mohamed^1^; O. Kassar^2^; M. Meshref^3^

##### ^*1*^ *Faculty of Medicine, Mansoura University, Mansoura, Egypt;* ^*2*^ *Faculty of Medicine, Alexandria University, Alexandria, Egypt;* ^*3*^ *Neurology Department, Faculty of Medicine, Al‐Azhar University, Cairo, Egypt*

## EPV‐0934

### Kappa free light chains – A quantitative alternative to oligoclonal bands in multiple sclerosis

#### J. Mićić ^1^; M. Danilović^2^; L. Arsenović^3^; E. Dinčić^2^

##### ^*1*^ *Faculty of Medicine, Military Medical Academy, University of Defence, Belgrade, Serbia;* ^*2*^ *Department of Neurology, Military Medical Academy, University of Defence, Belgrade, Serbia;* ^*3*^ *Institute of Biochemistry, Military Medical Academy, Belgrade, Serbia*

## EPV‐0935

### Kappa‐free light chain in predicting long term MS disability progression

#### A. Emekli^1^; E. Dilsiz^2^; N. Sancar^2^; M. Tezel^2^; H. Karpuzoğlu^3^; A. Aydın^3^; C. Ulusoy^2^; C. Küçükali^2^; R. Türkoğlu^4^; V. Yılmaz^2^; T. Gündüz^1^; E. Tüzün^2^; M. Kürtüncü ^1^

##### ^*1*^ *Department of Neurology, Istanbul Faculty of Medicine, Istanbul University, Istanbul, Türkiye;* ^*2*^ *Aziz Sancar Experimental Medicine and Research Institute, Istanbul University, Istanbul, Türkiye;* ^*3*^ *Department of Biochemistry, Istanbul Faculty of Medicine, Istanbul University, Istanbul, Türkiye;* ^*4*^ *Department of Neurology, Haydarpaşa Numune Training and Research Hospital, University of Health Sciences, Istanbul, Türkiye*

## EPV‐0936

### Ketogenic diet and fatigue in relapsing‐remitting multiple sclerosis: exploring a neuro‐metabolic signal in a pilot study

#### F. Filippi ^1^; S. Lorenzut^2^; R. Garbo^3^; E. Lamon^4^; I. Del Negro^4^; A. Nilo^1^; S. Pez^4^; G. Gigli^4^; M. Valente^4^

##### ^*1*^ *Department of Medicine (DMED), University of Udine, Udine, Italy;* ^*2*^ *Neurology Unit, Head Keck and Neuroscience Department, Santa Maria della Misericordia University Hospital, Udine, Italy;* ^*3*^ *SC Neurology of Gorizia Monfalcone, Azienda Sanitaria Universitaria Giuliano Isontina (ASUGI);* ^*4*^ *Clinical Neurology Unit, Udine University Hospital, Piazzale Santa Maria della Misericordia, Udine, Italy*

## EPV‐0937

### Life‐threatening hepatitis requiring liver transplantation in a patient with multiple sclerosis treated with ocrelizumab

#### M. Mihalache ^1^; A. Teodorescu^1^; M. Anghel^3^; I. Lupescu^1^; A. Dulămea^2^; M. Marian^2^; S. Iacob^3^; D. Anghel^2^

##### ^*1*^ *Department of Neurology, Fundeni Clinical Institute, Bucharest, Romania;* ^*2*^ *Department of Neurology, “Carol Davila” University of Medicine and Pharmacy, Bucharest, Romania;* ^*3*^ *Department of Gastroenterology, Fundeni Clinical Institute, Bucharest, Romania*

## EPV‐0938

### Linking corpus callosum diffusion tensor imaging biomarkers to MSFC and cognitive–motor dual‐task costs in RRMS

#### B. Celenk ^1^; B. Altunan^1^; B. Sunal^2^; A. Unal^1^

##### ^*1*^ *Neurology, Namik Kemal University Hospital, Tekirdag, Türkiye;* ^*2*^ *Radiology, Namik Kemal University Hospital, Tekirdag, Türkiye*

## EPV‐0939

### Management of multiple sclerosis‐related fatigue: An umbrella review of systematic reviews with meta‐analyses

#### Y. Hawas ^1^; M. Abouzid^2^; A. Hamad^3^; M. El‐Moslemani^4^; E. Salama^5^; M. Al Azzawi^6^; D. Ewis^7^; A. Arafa^4^; H. Saeed^1^; A. Alnajjar^8^; A. Elnaga^9^; M. Khalil^1^

##### ^*1*^ *Faculty of Medicine, Tanta University, Tanta, Egypt;* ^*2*^ *Department of Physical Pharmacy and Pharmacokinetics, Poznan University of Medical Sciences, Poznan, Poland;* ^*3*^ *Faculty of Medicine, Menoufia University, Menoufia, Egypt;* ^*4*^ *Faculty of Medicine, Al‐Azhar University, Damietta, Egypt;* ^*5*^ *Faculty of Medicine, Benha University, Benha, Egypt;* ^*6*^ *Faculty of Medicine, The National Ribat University, Khartoum, Sudan;* ^*7*^ *Faculty of Medicine, Beni Suef University, Beni Suef, Egypt;* ^*8*^ *Faculty of Medicine, Al‐Azhar University, Gaza, Palestine;* ^*9*^ *Faculty of Medicine, Mansoura university, Mansoura, Egypt*

## EPV‐0940

### Metabolomic alterations in central and peripheral nervous system demyelinating diseases

#### M. Zido ^1^; Z. Neuerova^2^; E. Cifkova^2^; M. Lisa^2^; I. Stetkarova^1^

##### ^*1*^ *Neurological Department, University Hospital Kralovske Vinohrady and 3rd Faculty of Medicine, Charles University, Prague, Czech Republic;* ^*2*^ *Department of Chemistry, University of Hradec Kralove, Hradec Kralove, Czech Republic*

## EPV‐0941

### Migraine and tension‐type headache in multiple sclerosis: A two‐year, prospective, longitudinal, controlled study

#### P. Gklinos; E. Evangelopoulos; G. Velonakis; D. Mitsikostas

##### 1st Department of Neurology, Eginition University Hospital, National and Kapodistrian University of Athens, Athens, Greece

## EPV‐0942

### Motivations and treatment shifts in older multiple sclerosis patients: Understanding treatment changes after age 50

#### S. Ozakbas ^1^; E. Kaya^2^; C. Caliskan^1^; E. Zengin^1^; Y. Simsek^1^

##### ^*1*^ *Izmir University of Economics, Medical Point Hospital, Izmır, Türkiye;* ^*2*^ *Department of Neurology, University of Health Science, Tepecik Training and Research Hospital, Izmir, Türkiye*

## EPV‐0943

### Motor–cognitive phenotypes in early multiple sclerosis: Insights from a multidimensional assessment

#### M. Betti ^1^; I. Addazio^1^; C. Ballerini^1^; R. Bonacchi^2^; A. Caporali^1^; F. Chiappetta^1^; E. De Meo^1^; F. Gerli^3^; C. Niccolai^3^; G. Pasquini^3^; L. Pastò^1^; V. Penati^1^; E. Fainardi^4^; E. Portaccio^1^; M. Amato^1^

##### ^*1*^ *University of Florence, Department of NEUROFARBA, Florence, Italy;* ^*2*^ *Vita‐Salute San Raffaele University, Milan, Italy;* ^*3*^ *IRCCS Don Carlo Gnocchi Foundation, Florence, Italy;* ^*4*^ *Neuroradiology Unit, Department of Experimental and Clinical Biomedical Sciences “Mario Serio”, University of Florence, Florence, Italy*

## EPV‐0944

### Multiparametric assessment of MS at diagnosis: is kappa free light chain index associated with clinical, radiological, and retinal findings?

#### A. Miscioscia ^1^; D. Landi^1^; G. Brugnoni^1^; C. Nicoletti^1^; G. Mataluni^1^; F. Napoli^1^; C. Dionisi^1^; A. Martucci^2^; M. Cesareo^2^; C. Nucci^2^; F. Di Giuliano^3^; D. Centonze^1^; G. Marfia^1^

##### ^*1*^ *Multiple Sclerosis Clinical and Research Unit, Department of Systems Medicine, University of Rome Tor Vergata, Rome, Italy;* ^*2*^ *Ophthalmology Unit, Department of Experimental Medicine, University of Rome Tor Vergata, Rome, Italy;* ^*3*^ *Neuroradiology Unit, Department of Biomedicine and Prevention, University of Rome Tor Vergata, Rome, Italy*

## EPV‐0945

### Multiple sclerosis phenotype transition patterns

#### R. Aliyev^1^; D. Orujova ^2^

##### ^*1*^ *Department of Neurology, Azerbaijan Medical University, Baku, Azerbaijan;* ^*2*^ *Department of Neurology, Republican Clinical Hospital named after Academician M.Mirgasymov, Baku, Azerbaijan*

## EPV‐0946

### Multiple sclerosis relapse presenting with broken heart syndrome – A case report

#### K. Dimitrova; M. Dimitrova

##### UMHATEM “N. I. Pirogov”, Sofia, Bulgaria

## EPV‐0947

### Multiple sclerosis‐related uveitis and its prognostic value

#### E. Kaya ^1^; U. Samedzade^2^; E. Zengin^2^; S. Ozakbas^2^

##### ^*1*^ *Tepecik Training and Research Hospital, Department of Neurology, Izmir, Türkiye;* ^*2*^ *Izmir Economy University, Department of Neurology, Izmir, Türkiye*

## EPV‐0948

### Nabiximols improves sleep architecture in multiple sclerosis: A prospective polysomnographic study

#### A. Saraceno; F. Fortunato; S. Barone; M. Sturniolo; P. Bruno; I. Martino; E. Brescia; I. Sammarra; P. Valentino; A. Gambardella

##### Institute of Neurology, Department of Medical and Surgical Sciences, University Magna Graecia of Catanzaro, Italy

## EPV‐0949

### Neurofilament light as a biomarker for stratifying treatment intensity in newly diagnosed multiple sclerosis

#### K. Tešija; Ž. Vogrinc; T. Tunjić; T. Gabelić; I. Adamec; B. Barun; I. Martinez; M. Sredanović; M. Krbot Skorić; M. Habek

##### University Hospital Center Zagreb, Croatia

## EPV‐0950

### Objective assessment of fatigue in relapsing–remitting multiple sclerosis

#### N. Kováčiková ^1^; L. Kotúľová^2^; M. Grendár^2^; E. Kurča^1^; Š. Sivák^1^; E. Kantorová^1^

##### ^*1*^ *Department of Neurology, Faculty of Medicine, Comenius University, University Hospital Martin, Martin, Slovakia;* ^*2*^ *Bioinformatics Consulting Center, Jessenius Faculty of Medicine in Martin, Comenius University in Bratislava, Martin, Slovakia*

## EPV‐0951

### Ocrelizumab effect on progression: real‐world data in large cohort of multiple sclerosis patients

#### G. Romano ^1^; M. Fordellone^2^; A. Gallo^3^; A. d'Ambrosio^3^; V. Cimmino^1^; A. Bisecco^3^; D. Mele^1^; D. Archetto^1^; C. Coppola^1^; S. Bonavita^1^; G. Lus^1^; E. Signoriello^1^

##### ^*1*^ *Multiple Sclerosis Center, UOSD Second Neurology, University of Campania “Luigi Vanvitelli”, Napoli, Italy;* ^*2*^ *Medical Statistics Unit, University of Campania “Luigi Vanvitelli”, Naples, Italy;* ^*3*^ *I Division of Neurology and Department of Advanced Medical and Surgical Sciences, University Hospital and University of Campania “Luigi Vanvitelli”, Naples, Italy*

## EPV‐0952

### OCT measures at multiple sclerosis onset and early therapeutic management: A retrospective, real‐world study

#### M. Zidda ^1^; C. Principi^1^; G. Greco^1^; L. Ahmad^2^; F. Masi^1^; C. Redemagni^2^; E. Rigoni^2^; E. Colombo^2^

##### ^*1*^ *Department of Brain and Behavioral Sciences, University of Pavia, Pavia, Italy;* ^*2*^ *Multiple Sclerosis Centre, IRCCS Mondino Foundation, Pavia, Italy*

## EPV‐0953

### Ofatumumab efficacy, safety, adherence, and retention in Cypriot Multiple Sclerosis patients

#### E. Kkolou; M. Pantzaris; K. Kleopa; I. Smoleski; A. Koupparis; A. Adamou; G. Pitsas

##### Clinical Department, The Cyprus Institute of Neurology and Genetics, Nicosia, Cyprus

## EPV‐0954

### Participation and perceived benefits of physical activity in multiple sclerosis over a decade

#### G. Coghe^1^; I. Schiavetti^2^; E. Idini ^1^; A. Contu^1^; E. Cocco^1^; J. Frau^1^

##### ^*1*^ *University of Cagliari, Asl Cagliari Multiple Sclerosis Center Binaghi, Cagliari, Italy;* ^*2*^ *Departement of Health Sciences (Dissal), University of Genoa, Biostatistic Unit, Genova, Italy*

## EPV‐0955

### Parvovirus B19 infection associated with ocrelizumab in a patient with multiple sclerosis: A case report

#### C. Falsetti ^1^; A. Barilaro^2^; G. Masi^1^; A. Poggesi^1^; L. Massacesi^1^; A. Mariottini^1^

##### ^*1*^ *Department of Neurosciences, Psychology, Drug Research and Child Health, University of Florence, Florence, Italy;* ^*2*^ *Emergency Neurology Unit, Careggi University Hospital, Florence, Italy*

## EPV‐0956

### Passive smartphone usage data as a digital biomarker for cognitive impairment in multiple sclerosis

#### L. Montoya Vera ^1^; M. De Brouwer^3^; M. Stojchevska^3^; M. Cambron^2^; S. Van Hoecke^3^; F. Ongenae^3^; G. Laureys^1^

##### ^*1*^ *Department of Head and Skin, Ghent University, Ghent, Belgium;* ^*2*^ *Neurology Department, AZ Sint Jan, Brugge, Belgium;* ^*3*^ *Department of Internet technology and Data science Lab, Ghent University‐imec, Ghent, Belgium*

## EPV‐0957

### Patient‐reported outcomes in Serbian ocrelizumab‐treated relapsing multiple sclerosis patients with advanced disability: The MuSicalE Interim Analysis

#### J. Drulovic ^1^; S. Vojinovic^2^; N. Veselinovic^3^; D. Aleksic^4^; D. Savic^2^; M. Jeremic^3^; S. Mesaros^3^; T. Boskovic Matic^4^; M. Bulatovic^5^; I. Pavlovic^6^; T. Pekmezovic^7^

##### ^*1*^ *Department of Neurology, University Clinical Center of Serbia, Belgrade, Serbia;* ^*2*^ *Department of Neurology, University Clinical Center Nis, Nis; Faculty of Medicine, University of Nis, Nis, Serbia;* ^*3*^ *Department of Neurology, University Clinical Center of Serbia, Belgrade; Faculty of Medicine, University of Belgrade, Belgrade, Serbia;* ^*4*^ *University of Kragujevac, Serbia, Faculty of Medical Sciences, Department of Neurology;* ^*5*^ *Hemofarm A.D., Vrsac, Serbia;* ^*6*^ *Roche d.o.o., Belgrade, Serbia;* ^*7*^ *Institute of Epidemiology, Faculty of Medicine, University of Belgrade, Belgrade, Serbia*

## EPV‐0958

### Patient‐reported wearing‐off symptoms in Ocrelizumab‐treated multiple sclerosis and their impact on quality of life

#### A. Khoudi; A. Souissi; A. Gharbi; A. Mansouri; A. Atrous; Y. Abida; I. Kacem; A. Gargouri; R. Gouider

##### Neurology Department, LR18SP03 and Clinical Investigation Center Neurosciences and Mental Health – University Hospital Center Razi‐ Mannouba, Tunis, Tunisia – Faculty of Medicine of Tunis, Tunis El Manar University, Tunis, Tunisia

## EPV‐0959

### Physical disability predicts multidomain cognitive dysfunction in multiple sclerosis

#### M. González Platas ^1^; M. Pérez Martín^2^

##### ^*1*^ *Hospital General de Fuerteventura. Las palmas de Gran Canarias, Spain;* ^*2*^ *Neuralia Centro de Rehabilitación Neurologica, Santa Cruz de Tenerife, Spain*

## EPV‐0960

### Plasma biomarkers as early indicators of cognitive and motor impairment in Multiple Sclerosis: A prospective observational study

#### B. Giovannini; N. Porcino; A. Dini; F. Bianchi; L. Pasquali

##### Department of Clinical and Experimental Medicine, Neurology Unit, University of Pisa, Pisa, Italy

## EPV‐0961

### Pre‐diagnostic fatigue and fatigue burden at diagnosis in multiple sclerosis

#### N. Porcino; B. Giovannini; A. Dini; F. Bianchi; L. Pasquali

##### University of Pisa, Pisa, Italy

## EPV‐0962

### Predictors of treatment failure following immune reconstitution therapies in relapsing multiple sclerosis: A real‐world cohort

#### A. Gifreu‐Fraixinó; A. Garcia‐Sarreón; A. Pardiñas Ramon; A. Boix‐Lago; J. Gutiérrez Naranjo; C. Coll‐Martínez; A. Miguela‐Benavides; J. Huertas‐Pons; R. Álvarez Morell; J. Gich Fullà; A. Quiroga‐Varela; G. Álvarez‐Bravo

##### Girona Biomedical Research Institute (IDIBGI), Neurodegeneration and Neuroinflammation Research Group, Salt, Spain

## EPV‐0963

### Predominant verbal fluency deficit in multiple sclerosis

#### E. Babych; Y. Solodovnikova

##### Department of Neurology and Neurosurgery, Odesa National Medical University, Odesa, Ukraine

## EPV‐0964

### Pregnancy and clinical outcomes in women with multiple sclerosis: data from the Republic of Moldova

#### A. Belenciuc ^1^; V. Macari^2^; M. Sangheli^2^; V. Lisnic^2^

##### ^*1*^ *Department of Neurology, Institute of Neurology and Neurosurgery “Diomid Gherman”, Chișinău, Republic of Moldova;* ^*2*^ *Department of Neurology, “Nicolae Testemitanu”, State University of Medicine and Pharmacy, Chișinău, Republic of Moldova*

## EPV‐0965

### Preserving professional autonomy in multiple sclerosis: A multimodal management of lower urinary tract dysfunction

#### P. Perdun ^1^; A. Pleșa^1^; A. Pleșa^2^; C. Sîrbu^1^; C. Sîrbu^2^; C. Sîrbu^3^

##### ^*1*^ *“Carol Davila” University of Medicine and Pharmacy, Bucharest, Romania;* ^*2*^ *Neurology Department, “Dr. Carol Davila” Central Military Emergency University Hospital, Bucharest, Romania;* ^*3*^ *Academy of Romanian Scientists, Bucharest, Romania*

## EPV‐0966

### Prevalence and treatment efficacy of trigeminal neuralgia in patients with multiple sclerosis

#### F. Balcı

##### University of Health Sciences, Hamidiye Faculty of Medicine, Neurology, Istanbul, Türkiye

## EPV‐0967

### Prevalence of Epstein–Barr virus infection in inflammatory diseases of the central nervous system

#### M. Silva ^1^; M. Soares^2^; P. Faustino^3^; I. Gomes^3^; F. Ladeira^3^; J. Sequeira^3^; C. Capela^3^; M. Leal Rato^4^

##### ^*1*^ *São José Local Health Unit, Lisbon, Portugal;* ^*2*^ *São José Local Health Unit, Lisbon;Lisbon Academic Clinical Center; Multiple Sclerosis Integrated Care Unit, São José Local Health Unit, Lisbon, Portugal;* ^*3*^ *Lisbon Academic Clinical Center; Multiple Sclerosis Integrated Care Unit, São José Local Health Unit, Lisbon, Portugal;* ^*4*^ *Lisbon Academic Clinical Center; Multiple Sclerosis Integrated Care Unit, São José Local Health Unit, Lisbon, Portugal; Egas Moniz Center for Interdisciplinary Research, Faculty of Medicine, University of Lisbon, Lisbon, Portugal*

## EPV‐0968

### Psychiatric disorders preceding the diagnosis of multiple sclerosis: A case series

#### F. Ben Chihi ^1^; H. Derbali^1^; I. Bedoui^1^; Y. Mrad Dali^1^; M. Messelmani^1^; M. Mansour^1^; N. Fekih Mrissa^2^; J. Zaouali^1^

##### ^*1*^ *Department of Neurology, Military Hospital of Tunis, Tunisia;* ^*2*^ *Molecular Biology Unit (UR17DN06), Laboratory of Hematology, Military Hospital of Tunis, Tunisia*

## EPV‐0969

### Quality of reporting randomized controlled trials in exercise approaches for multiple sclerosis: A CONSORT‐based assessment (up to 2025)

#### D. Onan ^1^; M. Kodounis^2^

##### ^*1*^ *Department of Physiotherapy and Rehabilitation, Faculty of Health Sciences, Yozgat Bozok University, Yozgat, Türkiye;* ^*2*^ *First Neurology Department, Eginitio Hospital, School of Medicine, National & Kapodistrian University of Athens, Athens, Greece*

## EPV‐0970

### Radiologically isolated syndrome: New challenges for prognosis and management

#### D. Korobko ^2^; I. Arkhipov^1^; A. Prokaeva^2^; L. Vasilkiv^3^; A. Tulupov^3^

##### ^*1*^ *Novosibirsk Regional Clinical Hospital, Novosibirsk, Russian Federation;* ^*2*^ *Novosibirsk State Medical Universit of Minzdrav of Russia, Novosibirsk, Russian Federation;* ^*3*^ *International Tomography Center of SB of RAS, Novosibirsk, Russian Federation*

## EPV‐0971

### Real‐world treatment satisfaction in multiple sclerosis: Digital patient reported outcomes comparison between intravenous and subcutaneous anti‐CD20

#### B. Friedrich^1^; J. Steinmetz‐Späh^1^; A. Urbano^1^; N. Joschko^2^; T. Wiemer^2^; L. Masanneck^3^; T. Ziemssen ^4^

##### ^*1*^ *Temedica GmbH, Munich, Germany;* ^*2*^ *Roche Pharma AG, Grenzach‐Wyhlen, Germany;* ^*3*^ *Department of Neurology, Medical Faculty and University Hospital, Heinrich Heine University Düsseldorf;* ^*4*^ *Center of Clinical Neuroscience, Department of Neurology, University Clinic Carl Gustav Carus and Dresden University of Technology, Dresden, Germany*

## EPV‐0972

### Real‐world use of Ublituximab: Patient profile in clinical practice

#### L. García‐Vasco; A. Paz‐Tamayo; I. Gómez‐Estévez; C. Sánchez‐García; J. Quezada‐Sánchez; P. Martín‐Sánchez; C. Oreja‐Guevara

##### Department of Neurology, Hospital Clínico San Carlos, Madrid, Spain

## EPV‐0973

### Retrospective analysis of lymphocyte dynamics in multiple sclerosis patients treated with fingolimod

#### L. Bir; S. Tekin; K. Özdemir, M. Sarılar

##### Neurology, Pamukkale University, Denizli, Türkiye

## EPV‐0974

### Revisiting corpus callosum index data after 20 years is it sensitive enough to predict evolution?

#### G. Medeiros Andrade Figueira; S. Paula Vallegas Soares; S. Raquel Custodio; C. Viviane Tavares Carvalho; V. Alexandra Seide Cardoso; M. Nunes Ferreirinha Leite de Castro; F. Tome do Nascimento; F. Faria Andrade Figueira

##### Dept of Neurology, Hospital São Francisco na Providência de Deus, Rio de Janeiro, Brazil

## EPV‐0975

### Rituximab as a single therapeutic strategy in coexisting takayasu arteritis and multiple sclerosis: A case report

#### F. Oggiano ^1^; M. Modesto^1^; D. Lomonaco^1^; M. Caccamo^1^; P. De Marco^1^; P. Colella^2^; V. De Marco^1^; A. Rini^1^

##### ^*1*^ *Neurological Department, A. Perrino's Hospital, Brindisi, Italy;* ^*2*^ *Neuroradiology Department, A. Perrino's Hospital, Brindisi, Italy*

## EPV‐0976

### Screening chronic fatigue in MS with MFIS: Clinical correlates in a real‐world cohort

#### E. Rraklli ^1^; A. Quka^2^; A. Kuqo^2^; M. Papajani^2^; P. Djamandi^2^; S. Grabova^2^; J. Kruja^2^

##### ^*1*^ *Regional Hospital Center, Berat, Albania;* ^*2*^ *UHC Mother Theresa, Tirane, Albania*

## EPV‐0977

### Sepsis mortality in multiple sclerosis: Trends and disparities using CDC WONDER

#### A. Nasim ^1^; V. Dhaduk^2^; H. Ka‐Hey Mah^3^; S. Rajeshwara^4^; P. Gudipati^5^; A. Paloh^6^; E. Nguyen^7^; A. Nizar Mawti^8^; K. Raval^9^; M. Neway Tekle^10^; M. Francis^11^; J. Sabu Thomas^12^; R. Mathew Francis^13^; A. Salman^14^

##### ^*1*^ *Saba University School of Medicine, the Netherlands;* ^*2*^ *Shantabaa Medical College and General Hospital, Amreli, Gujarat;* ^*3*^ *Saba University School of Medicine, the Netherlands;* ^*4*^ *Shimoga Institute of Medical Sciences, Shimoga, Karnataka;* ^*5*^ *Jawaharlal Nehru Medical College;* ^*6*^ *Rajiv Gandhi University;* ^*7*^ *University of California Irvine;* ^*8*^ *University of Kurdistan;* ^*9*^ *GCS Medical College, Hospital and Research Centre;* ^*10*^ *University of Gondar;* ^*11*^ *St.Johns Medical College;* ^*12*^ *Saint Louis University College for Public Health and Social Justice;* ^*13*^ *Saint Louis University College for Public Health and Social Justice;* ^*14*^ *Dow University of Health Sciences, Karachi, Pakistan*

## EPV‐0978

### Serum calprotectin as a biomarker of inflammatory activity and treatment response in relapsing multiple sclerosis

#### I. Vigiser^1^; H. Kolb^2^; A. Karni^2^; M. Golan^2^; K. Regev ^2^

##### ^*1*^ *Gray Faculty of Medical & Health Sciences, Tel Aviv University, Tel Aviv, Israel;* ^*2*^ *Neuroimmunology and Multiple Sclerosis Unit, Neurology Institute, Tel Aviv Sourasky Medical Center, Tel Aviv, Israel*

## EPV‐0979

### Serum glial fibrillary acidic protein and neurofilament light chain as biomarkers of progression and cognitive decline in multiple sclerosis

#### C. Bava ^1^; F. Scavelli^2^; L. Giorgi^1^; A. Sala^2^; P. Valentino^2^; M. Lo Re^2^; S. Malucchi^2^; A. Di Sapio^2^

##### ^*1*^ *NICO‐Neuroscience Institute Cavalieri Ottolenghi, Orbassano, Italy;* ^*2*^ *University Hospital San Luigi Gonzaga, Department of Neurology and CReSM*, *Sanatorio San Luigi, Italy*

## EPV‐0980

### Serum neurofilament light chains and serum glial fibrillary acidic protein in relation to cognitive performance in multiple sclerosis

#### E. Platos ^1^; B. Helmlinger^2^; R. Demjaha^1^; C. Tafrali^1^; M. Martinez‐Serrat^1^; S. Hechenberger^3^; B. Heschl^2^; S. Wurth^2^; P. Benkert^4^; J. Kuhle^4^; M. Haindl^2^; E. Hofer^5^; A. Damulina^1^; S. Ropele^2^; C. Enzinger^2^; D. Pinter^2^; M. Khalil^2^

##### ^*1*^ *Neurology Biomarker Research Unit, Department of Neurology, Medical University of Graz, Graz, Austria;* ^*2*^ *Department of Neurology, Medical University of Graz, Graz, Austria;* ^*3*^ *Department of Neurology, Medical University of Graz, Graz, Austria, Department of Oncology, Medical University of Graz, Graz, Austria;* ^*4*^ *Multiple Sclerosis Centre and Research Center for Clinical Neuroimmunology and Neuroscience (RC2NB), Departments of Biomedicine and Clinical Research, University Hospital and University of Basel, Basel, Switzerland;* ^*5*^ *Institute for Medical Informatics, Statistics and Documentation, Medical University of Graz, Graz, Austria*

## EPV‐0981

### Sexual dysfunction and related factors in multiple sclerosis patients

#### O. Kopchak; K. Hrynevych

##### Department of Neurology, Psychiatry and Physical Rehabilitation Kyiv Medical University, Kyiv, Ukraine

## EPV‐0982

### Silent rebound: Tumefactive demyelinating lesion following the discontinuation of teriflunomide

#### A. Kale; G. Ertürk Kale; N. Tepe

##### Neurology, Balıkesir University Health Practice and Research Hospital, Balıkesir, Türkiye

## EPV‐0983

### Sleep disorders and quality of life in multiple sclerosis

#### D. HadjKacem ^1^; S. Daoud^1^; F. Kchaou^1^; S. Msaad^2^; N. Farhat^1^; K. Moalla^1^; N. Charfi^1^; N. Bouattour^1^; S. Sakka^1^; S. Kammoun^2^; M. Damak^1^

##### ^*1*^ *Department of Neurology, Habib Bourguiba University Hospital, Sfax, Tunisia;* ^*2*^ *Department of Pulmonology, Hedi Chaker University Hospital, Sfax, Tunisia*

## EPV‐0984

### SmartBioMS: Smartphone application for investigating speech as a passive biomarker to track progression in Multiple Sclerosis

#### J. Šmíd ^1^; V. Illner^1^; T. Tykalová^1^; M. Šubert^1^; M. Novotný^1^; M. Šimek^1^; T. Uher^2^; D. Horáková^2^; J. Motýl^2^; J. Rusz^1^

##### ^*1*^ *Faculty of Electrical Engineering, Czech Technical University in Prague, Czech Republic;* ^*2*^ *Department of Neurology and Centre of Clinical Neuroscience, First Faculty of Medicine, Charles University, Czech Republic*

## EPV‐0985

### Social cognition and cognitive impairment across RRMS and SPMS

#### K. Ntoskas^1^; T. Doskas ^2^

##### ^*1*^ *Department of Neurology, 251 Hellenic Air Force General Hospital, Athens, Greece;* ^*2*^ *Department of Neurology, Athens Naval Hospital, Athens, Greece*

## EPV‐0986

### Social cognition: A significant modifier of Quality of Life in MS

#### K. Ntoskas^1^; T. Doskas ^2^

##### ^*1*^ *Department of Neurology, 251 Hellenic Air Force General Hospital, Athens, Greece;* ^*2*^ *Department of Neurology, Athens Naval Hospital, Athens, Greece*

## EPV‐0987

### Statistical properties of tract density for MS, NMOSD and MOGAD

#### O. Pereverzeva ^1^; A. Mikitchuk^2^; A. Buniak^1^

##### ^*1*^ *Neurological Department, Republican Research and Clinical Center of Neurology and Neurosurgery, Minsk, Belarus;* ^*2*^ *Faculty of Radiophysics and Computer Technologies, Belarusian State University, Minsk, Belarus*

## EPV‐0988

### Statistical properties of tract density in corpus callosum for MS, NMOSD and MOGAD patients

#### O. Pereverzeva ^1^; A. Buniak^1^; A. Mikitchuk^2^

##### ^*1*^ *neurological department, Republican research and clinical center of neurology and neurosurgery, Minsk, Belarus;* ^*2*^ *Faculty of Radiophysics and Computer Technologies, Belarusian State University, Minsk, Belarus*

## EPV‐0989

### Supportive interventions to reduce caregiver burden among family caregivers of Multiple Sclerosis (MS) patients: A systematic review and meta‐analysis

#### A. Putra ^1^; H. Kusuma^2^; D. Zahra^1^; E. Wiyogo^1^; K. Maharani^1^

##### ^*1*^ *Department of Neurology, Faculty of Medicine, Universitas Indonesia, Jakarta, Indonesia;* ^*2*^ *School of Medicine and Health Sciences, Atma Jaya Catholic University of Indonesia, Jakarta, Indonesia*

## EPV‐0990

### The evolving burden of multiple sclerosis and migraine in G20 countries: A geospatial and socioeconomic analysis from 1990 to 2023

#### H. Hu; F. Yang

##### The First Medical Center, Chinese PLA General Hospital, Beijing, China

## EPV‐0991

### The rising role of cyberchondria and ehealth literacy in multiple sclerosis: Psychological reflections of online health information seeking

#### B. Geçer; Z. Koçak Geçer; S. Mungan

##### Neurology Department, Health Sciences University Bilkent City Hospital, Ankara, Türkiye

## EPV‐0992

### Theory of mind and empathy deficits are associated with verbal and executive dysfunction in relapsing–remitting multiple sclerosis

#### R. Turkoglu^1^; M. Tezel ^2^; E. Tuzun^2^; E. Arsoy^1^

##### ^*1*^ *Department of Neurology, Istanbul Haydarpasa Numune Training and Research Hospital, Istanbul, Türkiye;* ^*2*^ *Department of Neuroscience, Aziz Sancar Experimental Medicine Research Institute, Istanbul, Türkiye*

## EPV‐0993

### Therapeutic adherence in multiple sclerosis: The role of patient self‐management behaviors

#### B. Ben Dhaou^1^; K. Moalla^1^; O. Hammami ^1^; W. Amouri^2^; O. Jridi^2^; N. Bouattour^1^; S. Daoud^1^; S. Sakka^1^; M. Damak^1^

##### ^*1*^ *Department of Neurology, Habib Bourguiba Hospital, Sfax, Tunisia;* ^*2*^ *Higher Institute of Nursing Sciences, Sfax, Tunisia*

## EPV‐0994

### Time to disability milestones in NMOSD and MOGAD: A study of a Tunisian cohort

#### O. Ben Othman; R. Zouari; M. Saied; D. Ben Mohamed; A. Rachdi; F. Nabli; S. Ben Sassi

##### National Institute of Neurology, Adult Department, Tunis, Tunisia

## EPV‐0995

### Timing of high‐dose corticosteroids and motor recovery after immune‐mediated myelitis: a Girona neuroimmunology cohort

#### A. García‐Sarreón ^1^; A. Gifreu‐Fraixinó^1^; J. Gutierrez Naranjo^1^; A. Boix Lago^1^; A. Pardiñas Ramon^2^; G. Álvarez‐Bravo^1^

##### ^*1*^ *Multiple Sclerosis and Neuroimmunology Unit, Neurology Department, Dr. Josep Trueta University Hospital and Santa Caterina Hospital, Girona, Spain;* ^*2*^ *Neurodegeneration and Neuroinflammation Research Group,Girona Biomedical Research Institute (IDIBGI), Salt, Spain*

## EPV‐0996

### Transverse myelitis potentially linked to undifferentiated connective tissue disease: Clinical clues from a single case

#### A. Çolakoğlu; N. Kahveci Erdemir; A. Aksoy Gündoğdu

##### Neurology, Tekirdag Namik Kemal University, Tekirdag, Türkiye

## EPV‐0997

### Tuberculous longitudinally extensive transverse myelitis in an elderly patient: A diagnostic and therapeutic challenge

#### M. Ibrahim

##### Hôtel Dieu de France, Université Saint Joseph, Beirut, Lebanon

## EPV‐0998

### Tumefactive multiple sclerosis: A single‐centre experience

#### Y. Kooli ^1^; H. Derbali^1^; K. Chekili^1^; Y. Mrad^1^; I. Bedoui^1^; M. Messelmani^1^; M. Mansour^1^; N. Fekih Mrissa^2^; J. Zaouali^1^

##### ^*1*^ *Department of Neurology, Military Hospital of Tunis, Tunisia;* ^*2*^ *Molecular Biology Unit, Laboratory of Hematology, Military Hospital of Tunis, Tunisia*

## EPV‐0999

### Understanding real‐world engagement with a patient monitoring app: Insights from the Remote Multiple Sclerosis Care in Antwerp (ReMSCA) study

#### J. Derdelinckx ^1^; L. Costers^2^; Y. Vervoort^2^; R. Khan^2^; C. Van Coppenolle^3^; A. De Keersmaecker^4^; M. Breuls^5^; J. Kerstens^6^; N. Fockaert^7^; A. Symons^8^; T. Reynders^4^; W. Van Hecke^2^; B. Willekens^9^

##### ^*1*^ *Department of Neurology, Antwerp University Hospital, Edegem, Belgium and Laboratory of Experimental Hematology (Vaxinfectio), University of Antwerp, Antwerp, Belgium;* ^*2*^ *icometrix, Leuven, Belgium;* ^*3*^ *Janssen‐Cilag NV, a Johnson & Johnson company, Beerse, Belgium;* ^*4*^ *Department of Neurology, Antwerp University Hospital, Edegem, Belgium and Translational Neurosciences, Faculty of Medicine and Health Sciences, Wilrijk, Belgium;* ^*5*^ *Department of Neurology, Antwerp University Hospital, Edegem, Belgium and Department of Neurology, AZ Klina, Brasschaat, Belgium;* ^*6*^ *Department of Neurology, Antwerp University Hospital, Edegem, Belgium;* ^*7*^ *Department of Neurology, AZ Klina, Brasschaat, Belgium;* ^*8*^ *Department of Neurology, ZAS Middelheim and Cadix, Antwerp, Belgium;* ^*9*^ *Department of Neurology, Antwerp University Hospital, Edegem, Belgium; Translational Neurosciences and Laboratory of Experimental Hematology (Vaxinfectio), University of Antwerp, Antwerp, Belgium*

## EPV‐1000

### Unplanned pregnancy during ponesimod therapy in RRMS: Clinical course and outcome

#### G. Masi ^1^; G. Costantini^1^; A. Schmidt^1^; A. Barilaro^2^; L. Massacesi^1^; A. Poggesi^1^; A. Mariottini^1^

##### ^*1*^ *University of Florence, Department of Neurosciences, Psychology, Drug Research and Child Health, Florence, Italy;* ^*2*^ *Careggi University Hospital, Emergency Neurology Unit, Florence, Italy*

## EPV‐1001

### Using AI chatbots as a source of information for patients with multiple sclerosis

#### A. Micanek^1^; B. Adamova ^2^; I. Srotova^2^; M. Rous^3^; M. Vachova^4^; P. Hradilek^5^; D. Horakova^6^; E. Kubala Havrdova^6^

##### ^*1*^ *Grammar School Brno, Vídenska, Brno, Czech Republic;* ^*2*^ *Department of Neurology, University Hospital Brno and Faculty of Medicine , Masaryk University, Brno, Czech Republic;* ^*3*^ *Department of Neurology, Faculty of Medicine and Dentistry, Palacky University and University Hospital, Olomouc, Czech Republic;* ^*4*^ *Department of Neurology, KZ a.s., Hospital Teplice, Teplice, Czech Republic;* ^*5*^ *Department of Neurology, Faculty of Medicine, University of Ostrava, Ostrava, Czech Republic;* ^*6*^ *Department of Neurology, Centre of Clinical Neuroscience, First Faculty of Medicine, Charles University in Prague and General University Hospital, Prague, Czech Republic*

## EPV‐1002

### Video‐oculography markers of subclinical brainstem involvement in multiple sclerosis

#### A. Belyakova‐Bodina; E. Larina; A. Mezenchuk; K. Grammatchikova; A. Abramova; D. Ganin; A. Broutian; M. Zakharova

##### Research Center of Neurology, Moscow, Russia

## EPV‐1003

### Viral antibody profiles in multiple sclerosis and healthy controls: a cross‐sectional study

#### N. Morawiec; B. Adamczyk; N. Niedziela; J. Jurkiewicz; M. Adamczyk‐Sowa

##### Department of Neurology, Faculty of Medical Sciences in Zabrze, Medical University of Silesia in Katowice, Zabrze, Poland

## EPV‐1004

### When treating skin uncovers the brain: A case of multiple sclerosis in a patient on dupilumab for atopic dermatitis

#### M. Bruno; G. Romano; E. Signoriello; G. Lus

##### Multiple Sclerosis Centre, Second Division of Neurology, University of Campania “Luigi Vanvitelli”, Naples, Italy

## Muscle and neuromuscular junction disorder

## EPV‐1005

### A phase 3 study to evaluate the efficacy and safety of Telitacicept in patients with generalized myasthenia gravis: Trial design update

#### K. G. Claeys ^1^; F. Saccà^2^; A. A. Habib^3^; R. J. Nowak^4^; M. Pasnoor^5^; G. I. Wolfe^6^; T. Vu^7^; J. Sokolove^8^; J. F. Howard; Jr^9^

##### ^*1*^ *Department of Neurology, University Hospitals Leuven, and Laboratory for Muscle Diseases and Neuropathies, KU Leuven, Leuven, Belgium;* ^*2*^ *Università degli Studi di Napoli Federico II, Napoli, Italy;* ^*3*^ *Department of Neurology, University of California Irvine, Orange, CA, USA;* ^*4*^ *Department of Neurology, Yale School of Medicine, New Haven, CT, USA;* ^*5*^ *University of Kansas Medical Center, Kansas City, MO, USA;* ^*6*^ *University at Buffalo, Buffalo, NY, USA;* ^*7*^ *University of South Florida Morsani College of Medicine, Tampa, FL, USA;* ^*8*^ *Vor Bio, Boston, MA, USA;* ^*9*^ *University of North Carolina School of Medicine, NC, USA*

## EPV‐1006

### Association of complement deposition with disease severity and target treatment in dysferlinopathy

#### F. Zheng; S. Kang; W. Zhang; H. Lv; J. Deng; Y. Yuan; Z. Wang; M. Yu

##### Department of Neurology, Peking University First Hospital, Beijing, China

## EPV‐1007

### CAPTIVATE trial design: A pivotal phase 3 study of claseprubart in chronic inflammatory demyelinating polyneuropathy

#### L. Querol ^1^; J. Allen^2^; F. Eftimov^3^; S. Peric^4^; T. Dysgaard^5^; Y. Rajabally^6^; T. Harbo^7^; E. Nobile‐Orazio^8^; H. Katzberg^9^; D. Cornblath^10^; J. Katz^11^; J. Sheffield^12^; L. Hickey^12^; B. Beazley^12^; N. Carrillo^12^; R. Lewis^13^

##### ^*1*^ *Hospital de la Santa Creu, Barcelona, Spain;* ^*2*^ *University of Minnesota, Minneapolis, USA;* ^*3*^ *Amsterdam UMC, Amsterdam, The Netherlands;* ^*4*^ *University of Belgrade, Belgrade, Serbia;* ^*5*^ *University of Copenhagen, Copenhagen, Denmark;* ^*6*^ *University of Aston, Birmingham, UK;* ^*7*^ *Aarhus University Hospital, Aarhus, Denmark;* ^*8*^ *Università degli Studi di Milano, Milan, Italy;* ^*9*^ *Institute of Medical Science, Toronto, Canada;* ^*10*^ *Johns Hopkins University School of Medicine;* ^*11*^ *California Pacific Medical Center, San Francisco, California, USA;* ^*12*^ *Dianthus Therapeutics, NY, USA;* ^*13*^ *Cedars Sinai, LA, CA, USA*

## EPV‐1008

### Cardiovascular burden among patients with myasthenia gravis in France: A retrospective study using the french national insurance database

#### C. Blein ^1^; J. Podhorna^1^; A. Echaniz‐Laguna^2^; J. Camdessanché^3^; S. Attarian^4^; M. Ciumas^1^; G. Solé^5^

##### ^*1*^ *argenx BV, Ghent, Belgium;* ^*2*^ *Department of Neurology, APHP, CHU de Bicêtre, INSERM U1195, Paris‐Saclay University, Le Kremlin‐Bicêtre, France;* ^*3*^ *Department of Neurology, Neuromuscular Disease Reference Center, Hôpital Nord, University Hospital of Saint‐Étienne, Euro‐NMD, Saint‐Étienne, France;* ^*4*^ *Reference Center for Neuromuscular Disease and ALS, Timone University Hospital, Aix‐Marseille University, CHU Timone, Filnemus, Euro‐NMD, Marseille, France;* ^*5*^ *Neuromuscular Reference Center AOC, Neurology and Neuromuscular Diseases Department, Pellegrin Hospital, Bordeaux University Hospitals, Bordeaux, France*

## EPV‐1009

### Clinical characteristics, outcomes and comparison of conventional and newer therapies in myasthenia gravis

#### J. Sowmya ^1^; P. Sripadma^2^

##### ^*1*^ *senior Resident, Department of Neurology, Kastruba Medical College, Manipal Academy of Higher Education, Manipal, Karnataka, India;* ^*2*^ *associate Professor, Department of Neurology, Kastruba Medical College, Manipal Academy of Higher Education, Manipal, Karnataka, India*

## EPV‐1010

### Clinical, serological and therapeutic characteristics of immune‐mediated necrotizing myopathy: A case series

#### A. Akrivaki; A. Theodorou; C. Moschovos; M. Papadopoulou; E. Anagnou; S. Mesimvrinos; M. Arvaniti; E. Dimitriadou; G. Papagiannopoulou; S. Salakou; J. Tzartos; G. Tsivgoulis

##### Second Department of Neurology, “Attikon” University Hospital, School of Medicine, National and Kapodistrian University of Athens, Athens, Greece

## EPV‐1011

### Combined central and peripheral demyelination: Incidental findings or a single disease entity?

#### D. Alonso Vallín; A. Orejón Sanchez; B. Quintana Lopez; V. García Gracia; L. Gonzalez Fernández

##### *Hospital Universitario de Cabueñes*, *Spain*

## EPV‐1012

### Comparative efficacy of one cycle of efgartigimod versus standard of care in generalized myasthenia gravis

#### Q. Liu; W. Zhu; Y. Da

##### Department of Neurology, Xuanwu Hospital, Capital Medical University, Beijing, China

## EPV‐1013

### Content validity of the Myasthenia Gravis Impairment Index (MGII) patient‐reported ocular score in patients with ocular myasthenia gravis

#### M. Orlovic^1^; R. Jimenez^2^; W. Huang^2^; M. Mendes^1^; F. Verhammme^1^; F. Gistelinck^1^; J. Randall^3^; S. Hughes^3^; J. Morena^4^; N. Silvestri^2^; I. Lee^5^; C. Barnett‐Tapia^6^; C. Blein ^1^

##### ^*1*^ *argenx BV, Zwijnaarde, Ghent, Belgium;* ^*2*^ *argenx US Inc., Boston, USA;* ^*3*^ *Clinical Outcomes Solutions, Kent, UK;* ^*4*^ *Department of Neurology, Division of Neuromuscular Medicine, Duke University, Durham, USA;* ^*5*^ *Department of Neurology, Eleanor and Lou Gehrig ALS Center, Columbia University, New York, USA;* ^*6*^ *Department of Medicine, Division of Neurology, University of Toronto, Toronto, Canada*

## EPV‐1014

### Diagnostic value of muscle ultrasound as screening tool for congenital myopathies

#### D. Sparasci ^1^; S. Maitz^2^; O. Petrini^3^; C. Gobbi^1^; P. Ripellino^1^

##### ^*1*^ *Department of Neurology, Neurocenter of Southern Switzerland EOC, Lugano, Switzerland;* ^*2*^ *Service of Medical Genetics, Oncologic Institute of Southern Switzerland, EOC, Switzerland, Switzerland;* ^*3*^ *University of Applied Sciences and Arts of Southern Switzerland, Bellinzona, Switzerland*

## EPV‐1015

### Early cerebrospinal fluid cytokine changes after nusinersen loading phase in adult spinal muscular atrophy

#### B. Gębka‐ Kępińska; D. Gębka; J. Majchrzak; M. Cwynar; J. Jurkiewicz; B. Adamczyk; N. Morawiec; M. Lis; N. Niedziela; J. Bączyk; M. Adamczyk‐Sowa

##### Department of Neurology, Faculty of Medical Sciences in Zabrze Medical University of Silesia Katowice, Poland

## EPV‐1016

### Eculizumab enables rapid remission and successful embryo transfer in refractory generalized myasthenia gravis: A case report

#### Y. Deng

##### Department of Neurology, The Second Affiliated Hospital of Zhejiang University School of Medicine, Hangzhou, China

## EPV‐1017

### Effect of nipocalimab on sustained myasthenia gravis control during infections: Post‐hoc analysis of the vivacity‐MG3 study

#### C. Antozzi ^1^; G. Rodrigues^2^; M. Fitzgibbon^3^; R. Edwards^4^; I. Turkoz^5^; M. Kutch^5^; S. Ramchandren^3^; G. Hayat^6^

##### ^*1*^ *Immunotherapy and Apheresis Departmental Unit, Neuroimmunology and Muscle Pathology Unit, Fondazione IRCCS Istituto Neurologico C. Besta, Milan, Italy;* ^*2*^ *Johnson & Johnson, Madrid, Spain;* ^*3*^ *Medical Affairs, Johnson & Johnson, Raritan, NJ, USA;* ^*4*^ *Medical Affairs, Johnson & Johnson, Spring House, PA, USA;* ^*5*^ *Medical Affairs, Johnson & Johnson, Titusville, NJ, USA;* ^*6*^ *Department of Neurology, Saint Louis University School of Medicine, Saint Louis, MO, USA*

## EPV‐1018

### Effectiveness and tolerability of rozanolixizumab in generalized myasthenia gravis: A single‐center retrospective analysis of 18 cases

#### T. Chiba; G. Watanabe; A. Ishikawa; A. Tamagake; K. Tsukita; Y. Suzuki

##### Department of Neurology, National Hospital Organization Sendai Medical Center, Sendai, Japan

## EPV‐1019

### Efficacy and safety of eculizumab versus intravenous immunoglobulin in the acute exacerbation of myasthenia gravis

#### Y. Deng; L. Dong; L. Hu; Z. Wu; L. Yang; C. Zhang; H. Luo; X. Zhang; X. Yan; S. Wang; X. Yang; Q. Jiang

##### Myopathy Department, The First Affiliated Hospital of Guangzhou University of Chinese Medicine, Guangzhou, China

## EPV‐1020

### Abstract withdrawn

## EPV‐1021

### Efficacy and safety of efgartigimod versus intravenous immunoglobulin in moderate to severe generalized myasthenia gravis

#### H. Liu; Y. Wang; N. Xie; Q. Liu; X. Wen; W. Zhu; L. Di; Y. Da

##### Department of Neurology, Xuanwu Hospital, Capital Medical University, Beijing, China

## EPV‐1022

### Efficacy of nipocalimab in adult patients with none‐to‐mild ocular manifestations of generalized myasthenia gravis in Phase 3 VIVACITY‐MG3

#### K. Claeys ^1^; K. Gandhi^2^; I. Turkoz^3^; M. Kutch^4^; M. Fitzgibbon^5^; S. Ramchandren^3^

##### ^*1*^ *Department of Neurology, University Hospitals Leuven, and Laboratory for Muscle Diseases and Neuropathies, KU Leuven, Leuven, Belgium;* ^*2*^ *Medical Affairs, Johnson & Johnson, Horsham, PA, USA;* ^*3*^ *Medical Affairs, Johnson & Johnson, Titusville, NJ, USA;* ^*4*^ *Cytel, Inc, MA, USA;* ^*5*^ *Medical Affairs, Johnson & Johnson, Raritan, NJ, USA*

## EPV‐1023

### Emergency use of eculizumab in impending and manifest myasthenic crisis: A retrospective case series

#### Y. Huang; Q. Xu

##### Department of Neurology, Shenzhen People's Hospital (The Second Clinical Medical College, Jinan University, The First Affiliated Hospital, Southern University of Science and Technology), Shenzhen, China

## EPV‐1024

### Evaluation of decrement patterns in distal and proximal muscles using repetitive nerve stimulation in myasthenia gravis

#### Z. Gunes; Z. Matur

##### Department of Neurology, Bezmialem Vakıf University Faculty of Medicine, Istanbul, Türkiye

## EPV‐1025

### Experiences with Myotonic Dystrophy Type 1 (DM1) and treatment with Del‐desiran: Interviews with participants in MARINA‐OLE™ and their caregivers

#### A. Davidson; K. Gallagher; S. Vaziri; C. Army; B. Knisely; M. Fowler

##### Avidity Biosciences, Inc., San Diego, USA

## EPV‐1026

### Generalized but not ocular weakness predicts quality of life in myasthenia gravis: A factor‐analytic and regression study

#### E. Strujic ^1^; D. Rutnik Fot^1^; S. Matosa^1^; Z. Popovic^1^; J. Rimac^2^; S. Misevic^2^; B. Kovac^2^; T. Mirosevic Zubonja^2^; S. Juric^1^; S. Tomic^1^; T. Gilman Kuric^1^

##### ^*1*^ *University Hospital Center Osijek, Department of Neurology, Faculty of Medicine Osijek, Josip Juraj Strossmayer University of Osijek, Croatia;* ^*2*^ *University Hospital Center Osijek, Department of Neurology, Croatia*

## EPV‐1027

### Head drop in myasthenia gravis – How does it influence patients’ quality of life and acceptable symptoms state (PASS)?

#### D. Orzechowski; E. Sobieszczuk; P. Szczudlik; A. Kostera‐Pruszczyk

##### Department of Neurology, Medical University of Warsaw, ERN EURO NMD, Warsaw, Poland

## EPV‐1028

### Hypokalaemic periodic paralysis: Don't miss thyrotoxicosis

#### A. Elsaddig

##### Neurology Department, Sheikh Tahnoon Bin Mohammed Medical City, Al Ain, United Arab Emirates

## EPV‐1029

### Impact of efgartigimod treatment on patients with generalized myasthenia gravis with distinct antibody profiles in a single‐center study

#### A. Onaka; G. Watanabe; M. Suzuki; G. Akao; A. Ishikawa; A. Tamagake; K. Tsukita; Y. Suzuki

##### Department of Neurology, National Hospital Organization Sendai Medical Center, Sendai, Japan

## EPV‐1030

### Impact of high‐dose methylprednisolone pulse therapy for ocular myasthenia gravis in a single‐center study

#### N. Matsuo; G. Watanabe; A. Ishikawa; A. Tamagake; K. Tsukita; Y. Suzuki

##### Department of Neurology, National Hospital Organization Sendai Medical Center, Sendai, Japan

## EPV‐1031

### Impact of published single‐fiber EMG reference values on diagnostic classification in myasthenia gravis

#### W. Zhong ^1^; A. Pogosean^2^; A. Punga^2^; B. Roy^1^

##### ^*1*^ *Department of Neurology, Yale New Haven Hospital, New Haven, USA;* ^*2*^ *Department of Medical Sciences, Uppsala University and Clinical Neurophysiology, Uppsala University Hospital, Uppsala, Sweden*

## EPV‐1032

### Indirect comparison of nipocalimab safety versus FcRn blockers, C5 complement inhibitors, rituximab and IVIg in generalized myasthenia gravis

#### S. Jacob ^1^; B. Hutton^2^; S. Van Sanden^3^; T. Denee^4^; W. Karmous^5^; J. Diels^3^; K. Gandhi^6^; M. Hashim^3^; N. Gilhus^7^

##### ^*1*^ *University Hospitals Birmingham NHS Foundation Trust, Birmingham, UK; Institute of Immunology and Immunotherapy, University of Birmingham, Birmingham, UK;* ^*2*^ *Ottawa Hospital Research Institute, Ottawa, Canada;* ^*3*^ *Johnson & Johnson, Beerse, Belgium;* ^*4*^ *Johnson & Johnson, Breda, Netherlands;* ^*5*^ *Johnson & Johnson, Issy les Moulineaux, France;* ^*6*^ *Johnson & Johnson, Horsham, PA, USA;* ^*7*^ *Department of Clinical Medicine, University of Bergen, Bergen, Norway; Department of Neurology, Haukeland University Hospital, Bergen, Norway*

## EPV‐1033

### Late‐onset multiple Acyl‐CoA dehydrogenase deficiency: Clinical features, diagnostic challenges, and the role of oxidative stress in pathophysiology

#### D. Zoppi ^1^; A. Russo^1^; F. Vallefuoco^1^; M. De Maria^1^; G. Esposito^2^; T. Fioretti^2^; V. Maiolo^2^; F. Santorelli^3^; R. Iodice^1^; S. Tozza^1^; R. Dubbioso^1^; F. Manganelli^1^; L. Ruggiero^1^

##### ^*1*^ *Department of Neurosciences, Reproductive and Odontostomatological Sciences, University of Naples Federico II, Naples, Italy;* ^*2*^ *CEINGE‐Biotecnologie Avanzate Franco Salvatore, Naples, Italy;* ^*3*^ *Molecular Medicine, IRCCS Fondazione Stella Maris, Pisa, Italy*

## EPV‐1034

### Long‐term clinical and functional outcomes in 5q spinal muscular atrophy of nusinersen‐treated patients (AME‐NUSILONG)

#### M. Meza Cano^2^; V. Lopez Diaz ^1^

##### ^*1*^ *Tecnológico de Monterrey, Monterrey, Nuevo León México;* ^*2*^ *Hospital Christus Muguerza Alta Especialidad, Monterrey, Nuevo Leon, Mexico*

## EPV‐1035

### Long‐term user assessment of ME&MGTM, an app tracking generalized Myasthenia Gravis manifestations

#### C. Barnett‐Tapia^1^; S. Lehnerer^2^; K. Drareni^3^; N. Pesic‐Heuvrard^3^; D. Ravindra^3^; N. Sellami^3^; S. Zinaï^3^; B. Dutta^4^; B. Yungher^4^; L. Carment ^3^; J. Howard Jr^5^

##### ^*1*^ *Ellen and Martin Prosserman Centre for Neuromuscular Diseases, Division of Neurology, Department of Medicine, University Health Network, University of Toronto, Toronto, Ontario, Canada;* ^*2*^ *Charité – Universitätsmedizin Berlin, corporate member of Freie Universität Berlin and Humboldt‐Universität zu Berlin, Department of Neurology with Experimental Neurology, Berlin, Germany, Paris, France;* ^*4*^ *Alexion, AstraZeneca Rare Disease, Boston, MA, USA;* ^*5*^ *University of North Carolina, Department of Neurology, Chapel Hill, USA*

## EPV‐1036

### ME&MGTM digital biomarkers to monitor generalized Myasthenia Gravis symptoms: DOMYA, a validation study

#### E. Berling^1^; I. Ebong^2^; C. Tard^3^; P. Cintas^4^; G. Guliani^5^; E. Lagrange^6^; L. Malatesta^7^; M. Michaud^8^; A. Nadaj‐Pakleza^9^; D. Ostrovskiy^10^; Y. Péréon^11^; M. Pulley^12^; S. Raja^13^; T. Ragole^14^; S. Sacconi^15^; S. Takacs^16^; C. Gorin^17^; A. Balique‐Laplanche^17^; N. Sellami^17^; S. Zinaï^17^; B. Yungher^18^; B. Dutta^18^; L. Carment ^17^; C. Scheiner^19^; P. Laforêt^20^

##### ^*1*^ *APHP, Service de Neurologie, Hôpital Raymond Poincaré, Centre de Référence Nord‐Est‐Ile‐de‐France, FHU PHENIX, Garches, France;* ^*2*^ *Department of Neurology, University of Kentucky College of Medicine, Lexington, USA;* ^*3*^ *U1172, Centre de Référence des Maladies Neuromusculaires Nord/Est/Île‐de‐France, Service de Neurologie A, CHU de Lille, Lille, France;* ^*4*^ *Service de Neurologie, Centre de Référence des Maladies Neuromusculaires, Toulouse University Hospital, Toulouse, France;* ^*5*^ *HealthPartners Institute, Bloomington, Department of Neurology, University of Minnesota, Minneapolis, MN HealthPartners Institute Neuroscience Research Center, St. Paul. Regions Hospital, St. Paul, USA;* ^*6*^ *Department of Neurology, Grenoble University Hospital, Grenoble, France;* ^*7*^ *Department of Neurology, Vanderbilt University Medical Center, Nashville, USA;* ^*8*^ *Centre de Référence des Maladies Neuromusculaires Nord‐Est‐Ile de France, Department of Neurology, University Hospital Centre, Nancy, France;* ^*9*^ *Centre de Référence des Maladies Neuromusculaires Nord‐Est‐Ile de France, Department of Neurology, University Hospital Centre, Strasbourg, France;* ^*10*^ *Department of Neurology, Hofstra University, North Shore, Mount Sinai School of Medicine, Neurological Associates of Long Island, New Hyde Park, USA;* ^*11*^ *CHU Nantes, Reference Centre for Neuromuscular Disorders AOC, FILNEMUS, Euro‐NMD, Hôtel‐Dieu, Nantes, France;* ^*12*^ *College of Medicine‐Jacksonville, Department of Neurology, University of Florida, Jacksonville, USA;* ^*13*^ *Department of Neurology, Duke University Medical Center, Durham, North Carolina, USA;* ^*14*^ *Department of Neurology, University of Colorado Anschutz Medical Campus, Aurora, Colorado, USA;* ^*15*^ *Referral Centre for Neuromuscular Diseases, CHU Nice, France;* ^*16*^ *Department of Neurology, Indiana University School of Medicine, Indianapolis, USA;* ^*17*^ *Ad Scientiam, Paris, France;* ^*18*^ *Alexion, AstraZeneca Rare Disease, Boston, USA;* ^*19*^ *University of Tennessee Medical Center, Knoxville, USA;* ^*20*^ *Nord‐Est/Ile‐de‐France Neuromuscular Reference Center, UMR 1179, Neurology Department, Raymond‐Poincaré Hospital, Garches, FHU PHENIX, UVSQ Paris‐Saclay University, France*

## EPV‐1037

### Metabolic myopathy due to carnitine palmitoyltransferase II (CPT II) deficiency: A case report

#### M. González Gómez; E. Gismera Fontes; F. Sánchez García; L. Cabral Esquea; M. González Sánchez

##### Department of Neurology, Guadalajara University Hospital, Guadalajara, Spain

## EPV‐1038

### Mild clinical presentation of anti‐SRP immune‐mediated necrotizing myopathy: A case report

#### Y. Kim

##### Department of Neurology, Hallym University Sacred Heart Hospital, Hallym University College of Medicine, Anyang, Republic of Korea

## EPV‐1039

### Muscle anoctaminopathies: Clinical and genetic spectrum in a case series

#### N. Marcos; A. Jauregi; A. González; M. Callejo; A. Lagüela; V. Anciones; Á. López; M. Martínez; L. Pastor; J. Mellada; F. Angoso; A. Rodríguez‐Antigüedad

##### Neurology Department, Cruces University Hospital, Barakaldo, Spain

## EPV‐1040

### Myasthenia gravis and pregnancy: A retrospective analysis from a large tertiary care centre in Russia

#### P. Isabekova; T. Alekseeva

##### Department of Neurology, Almazov National Medical Research Centre, Saint‐Petersburg, Russian Federation

## EPV‐1041

### Neurophysiological assessment of the hypoglossal nerve in oculopharyngeal muscular dystrophy

#### T. Andrade Martins ^1^; S. Palma^1^; A. Oliveira^2^; C. Alves^1^; C. Santos Silva^1,3^; P. Pereira^1^

##### ^*1*^ *Serviço de Neurologia do Hospital Garcia de Orta, Unidade Local de Saúde de Almada‐Seixal, Almada, Portugal;* ^*2*^ *Serviço de Neurologia, Unidade Local de Saúde do Estuário do Tejo, Vila Franca de Xira, Portugal;* ^*3*^ *Faculty of Medicine, Institute of Molecular Medicine, University of Lisbon, Lisbon, Portugal*

## EPV‐1042

### Ocular‐onset myasthenia gravis in a real‐world Italian referral center: Clinical characteristics and risk of generalization

#### G. Camera; P. Paone; E. Agazzi; M. Sgarzi

##### Papa Giovanni XXIII Hospital, Bergamo, Italy

## EPV‐1043

### Passive and active digital biomarkers for comprehensive monitoring of generalized myasthenia Gravis: Design of the HARMONY 360 study

#### S. Lehnerer^1^; R. Iorio^2^; F. de Rivera Garrido^3^; M. Dimachkie^4^; L. Gerischer^5^; S. Bieuvelet^6^; L. Carment ^6^; B. Landrieu^6^; N. Pesic‐Heuvrard^6^; N. Sellami^6^; S. Zinaï^6^; E. Järvinen^7^; J. Vissing^8^; P. Laforêt^9^

##### ^*1*^ *Charité – Universitätsmedizin Berlin, corporate member of Freie Universität Berlin and Humboldt‐Universität zu Berlin, Department of Neurology with Experimental Neurology, Berlin, Germany;* ^*2*^ *Department of Neuroscience, Università Cattolica del Sacro Cuore, Rome, Italy; Neurology Unit, Fondazione Policlinico Universitario A. Gemelli IRCCS, Rome, Italy; Neurology Unit, Fondazione Policlinico Universitario A. Gemelli IRCCS, Rome, Italy;* ^*3*^ *Reference Unit of Hereditary Ataxias and Paraplegias, Department of Neurology, IdiPAZ, Hospital Universitario La Paz, Madrid, Spain;* ^*4*^ *Department of Neurology, University of Kansas Medical Center, Kansas City, KS, USA;* ^*5*^ *Department of Neurology With Experimental Neurology, Charité‐Universitätsmedizin Berlin, Corporate Member of Freie Universität Berlin and Humboldt‐Universität zu Berlin, Berlin, Germany; Neuroscience Clinical Research Center, Charité‐Universitätsmedizin Be;* ^*6*^ *Ad Scientiam, Paris, France;* ^*7*^ *Merck OY, Espoo Finland, an affiliate of Merck KGaA, Darmstadt, Germany;* ^*8*^ *Department of Neurology, Copenhagen Neuromuscular Center, Rigshospitalet, University of Copenhagen, Denmark;* ^*9*^ *Nord‐Est/Ile‐de‐France Neuromuscular Reference Center, UMR 1179, Neurology Department, Raymond‐Poincaré Hospital, Garches, FHU PHENIX, UVSQ Paris‐Saclay University, France*

## EPV‐1044

### Persistent muscle damage after statin discontinuation: A retrospective series of 78 patients submitted to muscle biopsy

#### Y. Pamblanco^1^; N. Muelas^2^; R. Sivera ^2^; I. Azorín^2^; P. Martí^2^; R. Vilchez^2^; L. Gómez^2^; E. Millet^3^; J. Vílchez^2^

##### ^*1*^ *Neurology Department, Hospital Universitari Francesc de Borja, Gandia. Spain;* ^*2*^ *Neuromuscular Diseases Unit, Neurology Department, Hospital Universitari I Politècnic La Fe, Neuromuscular and Ataxias Research Group, Instituto de Investigación Sanitaria La Fe, Valencia, Spain;* ^*3*^ *Neurophysiology Department, Hospital Politècnic i Universitari La Fe, Valencia, Spain*

## EPV‐1045

### Phenotype, serum biomarkers, and quantitative muscle MRI in patients with mitochondrial myopathy due to single large‐scale mtDNA deletions

#### S. García‐Bellido Ruiz ^1^; P. Martín Jiménez^1^; L. Bermejo Guerrero^1^; L. Ochoa Sánchez^5^; M. Navarro Riquelme^5^; R. Garrido Moraga^5^; A. González Quintana^5^; A. Hernández Lain^2^; A. Bermejo Moriñigo^3^; V. González Méndez^3^; M. Martín Casanueva^4^; A. Blazquez Encinar^5^; C. Domínguez González^5^

##### ^*1*^ *Neurology Department; 12 de Octubre University Hospital, Madrid, Spain;* ^*2*^ *Pathology Department; 12 de Octubre University Hospital, Madrid, Spain;* ^*3*^ *Radiology Department; 12 de Octubre University Hospital, Madrid, Spain;* ^*4*^ *Clinical Genetics Department; 12 de Octubre University Hospital, Madrid, Spain;* ^*5*^ *12 de Octubre Research Institute (imas12), Madrid, Spain*

## EPV‐1046

### Portuguese Myasthenia gravis epidemiological and clinical study: Preliminary data

#### E. Santos ^1^; A. Matos^2^; S. Cruz^3^; M. Pinto^4^; I. Sa Pereira^4^; L. Braz^4^; C. Campos^5^; P. Pereira^6^; T. Aguiar^7^; M. Pereira^7^; C. Santos^8^; A. Veiga^9^; R. Tojal^10^; H. Costa^11^; T. Santos^12^; G. Bonifacio^13^; C. Borbinha^14^; F. Bernardo^15^; J. Forteza^16^; J. Machado^17^; H. Nzwalo^18^; I. Carvalho^2^; I. Ferreira^1^; S. Bernardo^3^

##### ^*1*^ *Neurology Department, Santo António University Hospital, Porto, Portugal;* ^*2*^ *Neurology Department, Coimbra University Hospital, Coimbra, Portugal;* ^*3*^ *Neurology Department, Amadora‐Sintra Hospital, Amadora, Portugal;* ^*4*^ *Neurology Department, Sao Joao University Hospital, Porto, Portugal;* ^*5*^ *Neurology Department, Santa Maria University Hospital, Lisboa, Portugal;* ^*6*^ *Neurology Department, Garcia de Orta Hospital, Almada, Portugal;* ^*7*^ *Neurology Department, SESARAM, Funchal, Portugal;* ^*8*^ *Neurology Department, Entre Douro e Vouga Hospital, Feira, Portugal;* ^*9*^ *Neurology Department, Trans‐os‐Montes Alto Douro Hospital, Vila Real, Portugal;* ^*10*^ *Neurology Department, Cascais Hospital, Cascais, Portugal;* ^*11*^ *Neurology Department, Gaia Espinho Hospital, Gaia, Portugal;* ^*12*^ *Neurology Department, Viseu Dao Lafoes Hospital, Viseu, Portugal;* ^*13*^ *Neurology Department, Arrabida Hospital, Faro, Portugal;* ^*14*^ *Neurology Department, Evora Hospital, Evora, Portugal;* ^*15*^ *Neurology Hospital, Algarve Hospital, Faro, Portugal;* ^*16*^ *Neurology Department, Guarda Hospital, Guarda, Portugal;* ^*17*^ *Neurology Department, Matosinhos Hospital, Matosinhos, Portugal;* ^*18*^ *Neurology Department, Litoral Alentejano Hospital, Santiago Cacem, Portugal*

## EPV‐1047

### Possible impact of very late onset disease on clinical presentation and myasthenic crisis in myasthenia gravis

#### N. Merten Athayde; L. Albuquerque Brito; E. Braga Oliveira; J. Soares Baima; M. Rolim Mendes de Alencar; H. Capistrano Freitas; C. Leite Rodrigues

##### Neuromuscular Unit of Neurology Department of the Hospital Geral de Fortaleza (HGF), Ceará, Brazil

## EPV‐1048

### Potential mechanistic advantages of aC1s targeting in myasthenia gravis: Upstream versus downstream complement blockade

#### S. Shelly ^1^; M. Lalla^2^; Y. Zhao^2^; L. Rehaume^2^; J. Cross^2^; T. Vu^3^

##### ^*1*^ *Rambam Medical Center, Haifa, Israel;* ^*2*^ *Dianthus Therapeutics, NY, USA;* ^*3*^ *University of South Florida, Tampa, USA*

## EPV‐1049

### Predictors of conversion of myasthenic crisis from impending myasthenic crisis

#### R. Singh; S. Joy; J. Parihar; A. Das; A. Elavarasi; A. Gupta; A. Aggarwal; A. Pandit; V. Vy; D. Vibha; A. Garg; R. Bhatia; A. Srivastava; M. Tripathi

##### All India Institute of Medical Sciences, Delhi, India

## EPV‐1050

### Pregnancy enhanced tracking with neonatal and infant assessment (PETUNIA): Design of a safety study for nipocalimab in generalized myasthenia gravis

#### N. Goyal^1^; C. Robinson^2^; C. Schneider‐Gold^3^; D. Clements^4^; M. Fitzgibbon^5^; I. Turkoz^6^; L. Gentner^6^; T. Adewole^6^; L. Schwartz^5^; M. Jacobson^6^; M. Van Horn ^4^

##### ^*1*^ *Stanford University School of Medicine, Stanford, USA;* ^*2*^ *Novant Health East Cooper, Mount Pleasant, USA;* ^*3*^ *Ruhr‐University of Bochum, Bochum, Germany;* ^*4*^ *Johnson & Johnson, Horsham, USA;* ^*5*^ *Johnson & Johnson, Raritan, USA;* ^*6*^ *Johnson & Johnson, Titusville, USA*

## EPV‐1051

### Quality of life outcomes in triple‐seronegative and MuSK antibody‐positive myasthenia gravis patients: Evidence from MyRealWorld‐MG

#### M. Orlovic^1^; C. Qi^2^; C. Blein^1^; D. Masschaele ^1^; L. Van de Veire^3^; N. Tollenaar^3^; S. Dewilde^3^; A. Meisel^4^; H. Murai^5^

##### ^*1*^ *argenx BV, Ghent, Belgium;* ^*2*^ *argenx US Inc., Boston, USA;* ^*3*^ *Services in Health Economics (SHE) BV, Brussels, Belgium;* ^*4*^ *Department of Neurology and Experimental Neurology, Charité – Universitätsmedizin Berlin, Berlin, Germany;* ^*5*^ *Department of Neurology, International University of Health and Welfare, Narita, Japan*

## EPV‐1052

### Real word data on alglucosidase – Cipaglucosidase/miglustat switching in LOPD: an Italian multiCenter experience

#### O. Musumeci ^1^; I. Arena^1^; C. Sancricca^2^; S. Servidei^2^; A. Sechi^3^; B. Risi^4^; M. Filosto^4^; L. Vercelli^5^; T. Mongini^5^; S. Fecarotta^6^; G. Parenti^6^; L. Ruggiero^7^; G. Crescimanno^8^; A. Toscano^1^

##### ^*1*^ *Department of Clinical and Experimental Medicine, University of Messina, Italy;* ^*2*^ *Neurophysiopathology Unit, Fondazione Policlinico Universitario A. Gemelli IRCCS, Rome, Italy;* ^*3*^ *Regional Coordinating Center for Rare Diseases, University Hospital of Udine, Udine, Italy;* ^*4*^ *NeMO‐Brescia Clinical Center for Neuromuscular Diseases, Brescia, Italy;* ^*5*^ *Neuromuscular Unit, Department of Neurosciences “Rita Levi Montalcini”, University of Turin, Turin, Italy;* ^*6*^ *Department of Translational Medical Sciences, Federico II University, Naples, Italy;* ^*7*^ *Department of Neurosciences, Reproductive and Odontostomatological Sciences, University of Naples Federico II, Naples;* ^*8*^ *Department of Biomedicine, Neuroscience, and Advanced Diagnostic (BIND), University of Palermo, Italy*

## EPV‐1053

### Real‐world analysis of healthcare resource utilization, costs, and work loss in patients in the united states initiating therapy for myasthenia gravis

#### L. Lal^1^; L. Miller‐Wilson ^1^; N. Princic^2^; C. Lew^2^; Y. Edwards^1^; N. Streicher^3^

##### ^*1*^ *Immunovant, Inc., New York, NY, USA;* ^*2*^ *Merative, Ann Arbor, MI, USA;* ^*3*^ *MedStar Georgetown University Hospital, Washington, USA*

## EPV‐1054

### Real‐world evidence on the timing of immunosuppressive therapy and post‐thymectomy outcomes in myasthenia gravis: A multicenter retrospective study

#### Y. Shi ^1^; H. Zhang^1^; M. Fan^1^; G. Qi^2^; Y. Cui^3^

##### ^*1*^ *Department of Neurology, Tianjin Medical University General Hospital, Tianjin, China;* ^*2*^ *Center of Treatment of Myasthenia Gravis, People's Hospital of Shijiazhuang, Shijiazhuang, China;* ^*3*^ *Department of Neurology, China National Clinical Research Center for Neurological Diseases, Beijing Tiantan Hospital, Capital Medical University, Beijing, China*

## EPV‐1055

### Relationship between peripheral nervous system damage and fatigue in immune checkpoint inhibitors

#### E. Akbas^1^; A. Yildirim ^2^; F. Atalah^3^; M. Besiroglu^4^; M. Gumus^4^

##### ^*1*^ *Mardin Training and Research Hospital, Istanbul, Turkey;* ^*2*^ *İstanbul Medeniyet University Göztepe Training and Research Hospital Neurology Department, Istanbul, Turkey;* ^*3*^ *Istanbul Prof. Dr. Cemil Tascioglu City Hospital, Istanbul, Turkey;* ^*4*^ *İstanbul Medeniyet University Göztepe Training and Research Hospital Oncology Department, Istanbul, Turkey*

## EPV‐1056

### Rethinking myasthenia gravis care through brain health lens: Systematic review on socio‐economic burden, patient pathways, digital tools adoption

#### G. Turchetti ^1^; V. Quoidbach^2^; S. Cannizzo^1^; A. Archer^3^; L. Allard^4^; W. Atzori^5^; A. Béhin^6^; J. Bruijnes^7^; G. Caldo^8^; M. Crean^8^; S. Dickson^9^; M. Filosto^10^; M. Friconneau^3^; A. Kole^11^; A. Kostera‐Pruszczyk^12^; M. Leonardi^13^; S. Łukomska^14^; M. Maestri^15^; L. Maggi^16^; A. Marcassoli^13^; A. Meisel^17^; D. Rothardt^18^; T. Ruck^19^; S. Sacconi^20^; L. Strani^21^; M. Uccheddu^22^; L. Wiehe^23^

##### ^*1*^ *Institute of Management, Scuola Superiore Sant'Anna, Pisa, Italy;* ^*2*^ *European Brain Council, Brussels, Belgium; Student, Warwick Medical School, University of Warwick, UK, Belgium;* ^*3*^ *Groupe d’intérêt Myasthénie de l’AFM Téléthon, France;* ^*4*^ *EU Myasthenia Gravis Association (EuMGA) and ABMM, Belgium;* ^*5*^ *Head of Patient Advocacy for EUCAN and International & Expanded Markets Alexion AstraZenecaRare Diseases, Sweden;* ^*6*^ *Institut de Myologie, Service de Neuro‐Myologie, AP‐HP, GH Pitié Salpêtrière, Paris, France;* ^*7*^ *Department of Neurology, Maastricht University Medical Centre, Maastricht, Netherlands;* ^*8*^ *European Academy of Neurology, Austria;* ^*9*^ *European Brain Council, Brussels, Belgium | Institute ofNeuroscience and Physiology, Sahlgrenska Academy, University of Gothenburg, Gothenburg, Sweden;* ^*10*^ *Department of Clinical and Experimental Sciences, University of Brescia, Brescia, Italy;* ^*11*^ *Global Patient Engagement Lead MG UCB Pharma, Belgium;* ^*12*^ *Warszawski Uniwersytet Medyczny, Warsaw, Poland;* ^*13*^ *Neurology, Public Health, Disability Unit & Coma Research Centre, Fondazione IRCCS Istituto Neurologico C.Besta, Milano, Italy;* ^*14*^ *Myasthenia Gravis Association – Face to Face, Poland;* ^*15*^ *Azienda Ospedaliero Universitaria Pisana, Pisa, Italy;* ^*16*^ *Fondazione IRCCS Istituto Neurologico Carlo Besta, Milan, Italy;* ^*17*^ *Department of Neurology with Experimental Neurology, Center forStroke Research Berlin, Integrated Myasthenia gravis Center, Charité‐Universitätsmedizin Berlin, Germany;* ^*18*^ *DMG, MG Association, Germany;* ^*19*^ *Ruhr University Bochum, Department of Neurology, BG University Hospital Bergmannsheil, Bochum, Germany;* ^*20*^ *Centre Hospitalier Universitaire de Nice, Nice, France;* ^*21*^ *Patient Advocacy Director Italy Alexion AstraZeneca Rare Diseases, Italy;* ^*22*^ *European Myasthenia Gravis Association (EuMGA), Associazione Italiana Miastenia, Italy;* ^*23*^ *Global Patient Advocacy Director, Rare Disease Lead Alexion AstraZeneca Rare Diseases, Spain*

## EPV‐1057

### Retrospective analysis of the association between extrathymic neoplasms and Myasthenia Gravis in a patient cohort

#### E. Martínez Campos ^1^; L. Torné Hernandez^1^; I. Pagola Lorz^1^; E. Vicente Cemborain^2^; I. Jericó Pascual^1^

##### ^*1*^ *Neurology, Hospital Universitario de Navarra, Pamplona, España;* ^*2*^ *Instituto de Salud Pública y Laboral de Navarra, Pamplona, España*

## EPV‐1058

### Semi‐quantitative analyses of muscle magnetic resonance imaging for pattern recognition in early idiopathic inflammatory myopathies

#### Y. Leven ^1^; S. Kim^2^; A. Sanner^2^; F. Merzou^3^; S. Groppa^3^; C. Nelke^4^; A. Othman^2^; A. Deistung^5^; B. Troppa^5^; A. Mensch^6^; S. Meuth^1^; T. Ruck^4^; M. Pawlitzki^1^

##### ^*1*^ *Department of Neurology, University Hospital Duesseldorf, Germany;* ^*2*^ *Department of Neuroradiology, University Medical Center Mainz, Germany;* ^*3*^ *Department of Neurology, University Hospital Saarland, Germany;* ^*4*^ *Department of Neurology, BG University Hospital Bergmannsheil, Bochum, Germany;* ^*5*^ *Department of Radiology, University Medicine Halle, Germany;* ^*6*^ *Department of Neurology, University Medicine Halle, Germany*

## EPV‐1059

### Short‐term comparison of efgartigimod vs. eculizumab in patients with myasthenia gravis: a dual‐center real‐world study

#### M. Fan ^1^; H. Zhang^1^; Y. Shi^1^; Y. Cui^2^; P. Tang^1^; H. Xue^1^; Y. Shao^1^; L. Yang^1^; H. Wang^2^

##### ^*1*^ *Department of Neurology, Tianjin Medical University General Hospital, Tianjin, China;* ^*2*^ *China National Clinical Research Center for Neurological Diseases, Beijing Tiantan Hospital, Capital Medical University, China*

## EPV‐1060

### Sleep disturbances in pediatric duchenne muscular dystrophy: Insights from the sleep disturbance scale for children

#### S. Lima ^1^; J. Cardoso^2^; M. Santos^3^; L. Morais^3^; R. Amorim^3^; V. Oliveira^4^; C. Garrido^3^

##### ^*1*^ *Neurology Department, Unidade Local de Saúde de Trás‐os‐Montes e Alto Douro, Vila Real, Portugal;* ^*2*^ *Pediatric Neurology Department, Centro Materno Infantil do Norte, Unidade Local de Saúde de Santo António, Porto, Portugal;* ^*3*^ *Multidisciplinary Pediatric Neuromuscular Diseases Team, Centro Materno Infantil do Norte, Unidade Local de Saúde de Santo António, European Reference Network for Rare Neuromuscular Diseases (EURO‐NMD) Center, Porto, Portugal;* ^*4*^ *Pediatric Pneumology Department, Centro Materno Infantil do Norte, Unidade Local de Saúde de Santo António, Porto, Portugal*

## EPV‐1061

### Sleep quality, depression, and functional status in adult patients with spinal muscular atrophy

#### S. Demirtas ^1^; G. Sunter^1^; B. Turkoglu^1^; S. Bulut^2^

##### ^*1*^ *Department of Neurology, Marmara University Pendik Training and Research Hospital, Istanbul, Türkiye;* ^*2*^ *Department of Emergency Medicine, Marmara University Faculty of Medicine, Istanbul, Türkiye*

## EPV‐1062

### Spectrum of orthopedic complications in children with autosomal recessive limb‐girdle muscular dystrophy: A tunisian cohort study

#### K. Chekili ^1^; H. Klaa^1^; T. Ben Younes^1^; Z. Miladi^1^; S. Bouchoucha^2^; A. Zioudi^1^; M. Jamoussi^1^; H. Benrhouma^1^; I. Ben Youssef Turki^1^; I. Kraoua^1^

##### ^*1*^ *LR18SP04 and Department of Pediatric Neurology, National Institute of Neurology Mongi Ben Hmida, Tunis, Tunisia;* ^*2*^ *LR18SP04 and Department of orthopedic of the Children's Hospital Bechir Hamza, Tunis, Tunisia*

## EPV‐1063

### Successful treatment of Glycogenosis Type V (McArdle's disease) with high‐dose vitamin B6

#### J. Schaefer ^1^; A. Saak^2^

##### ^*1*^ *Department of Neurology Uniklinikum Dresden, Dresden, Germany;* ^*2*^ *Department of Neurology, Kepler Universitätsklinikum Linz, Linz, Austria*

## EPV‐1064

### Sustained clinical benefit with limited retreatment: Real‐world experience with efgartigimod in achr‐positive generalized myasthenia gravis

#### G. D'Alvano; F. D'Anna; D. Marigliano; F. Capuozzo; R. Sansone; V. Todisco; A. Tessitore; A. Bisecco

##### Department of Advanced Medical and Surgical Sciences – University of Campania “Luigi Vanvitelli”, Caserta, Italy

## EPV‐1065

### Switching from alglucosidade alfa to avalglucosidase: Real‐world data from a single‐center experience

#### O. Musumeci; I. Arena; C. Ubergo; S. Drago; A. Toscano

##### Department of Clinical and experimental Medicine, University of Messina, Italy

## EPV‐1066

### The role of the QMG score in determining the therapeutic approach in patients with myasthenia gravis – a prospective clinical study

#### K. Dimitrova; M. Dimitrova

##### UMHATEM “N. I. Pirogov”, Sofia, Bulgaria

## EPV‐1067

### The role of vital capacity (VC) in myasthenia gravis (MG) patients without clinical signs (CS) of respiratory dysfunction (RD)

#### V. Haliyeuskaya; Y. Rushkevich; K. Malhina

##### Republican Scientific and Practical Center of Neurology and Neurosurgery, Minsk, Belarus

## EPV‐1068

### The story behind a foot drop: When EMG requires a second reading

#### D. Alonso Vallín; C. Benlloch López; R. Sivera Mascaró; J. Vázquez Costa; M. Sevilla Mantecón; N. Muelas Gómez

##### Hospital Universitari i Politècnic La Fe, Valencia, Spain

## EPV‐1069

### The true chameleon: A single center experience on Congenital Myasthenic Syndromes

#### J. Duarte ^1^; M. Brum^2^; L. Medeiros^3^

##### ^*1*^ *Neurology Department, São José Local Health Unit, Lisbon, Portugal;* ^*2*^ *Neurology Department, São José Local Health Unit, Lisbon, Portugal; CNS – Campus Neurológico, Lisbon, Portugal; Neurophysiology Unit, São José Local Health Unit, Lisbon, Portugal;* ^*3*^ *Neurophysiology Unit, São José Local Health Unit, Lisbon, Portugal*

## EPV‐1070

### Treatment with anti‐FcRn and C5‐inhibitors in drug‐resistant gMG‐real world data

#### E. Sobieszczuk; P. Szczudlik; B. Kierdaszuk; A. Kostera‐Pruszczyk

##### Department of Neurology, Medical University of Warsaw, ERN EURO NMD, Warsaw, Poland

## EPV‐1071

### Variable phenotypic expression associated with a recurrent ANO5 variant: A clinical, MRI and muscle biopsy case series

#### A. Alungulese ^1^; I. Catalina Alvarez^1^; J. Suarez Gonzalez^2^; F. Arias^3^; R. Garcia Uzquiano^4^; I. Millán Arredondo^5^; L. Del Pino tejado^1^; M. Esteban Rodriguez^6^

##### ^*1*^ *Department of Neurology, Gregorio Marañón University Hospital, Madrid, Spain;* ^*2*^ *Genomics Unit, Gregorio Marañón University Hospital, Madrid, Spain;* ^*3*^ *Department of Pathology, Gregorio Marañón University Hospital, Madrid, Spain;* ^*4*^ *Department of Pediatrics, Gregorio Marañón University Hospital, Madrid, Spain;* ^*5*^ *Department of Radiology, Gregorio Marañón University Hospital, Madrid, Spain;* ^*6*^ *Department of Pathology, La Paz University Hospital, Madrid, Spain*

## EPV‐1072

### Wearable‐based activity markers in Myasthenia Gravis

#### P. Epping ^1^; N. Huntemann^1^; U. Ceylan^2^; S. Faissner^2^; T. Ruck^3^; S. Meuth^1^; M. Pawlitzki^1^; L. Masanneck^1^

##### ^*1*^ *Department of Neurology, Medical Faculty and University Hospital Düsseldorf, Düsseldorf, Germany;* ^*2*^ *Department of Neurology, Katholisches Klinikum Bochum, Bochum, Germany;* ^*3*^ *Department of Neurology, BG University Hospital Bergmannsheil, Ruhr‐University Bochum, Bochum, Germany*

## EPV‐1073

### Where do patients with myasthenia gravis die? A retrospective study

#### S. Wasti ^1^; A. Wasti^2^

##### ^*1*^ *Department of Neurology, King Edward Medical University, Lahore, Pakistan;* ^*2*^ *Department of Neurology, Continental Medical College, Lahore, Pakistan*

## Neurocritical care

## EPV‐1074

### A study of serum fibrinogen level in acute ischemic stroke patients in critical care unit

#### D. Heeramani^2^; P. Anshu^1^; K. Bhoi^1^; P. Agnihotri^2^; D. Gupta ^1^

##### ^*1*^ *Departement of General Medicine Sri Balaji Institute of Medical Sciences Raipur, India;* ^*2*^ *Departement of Critical Care Medicine Sri Balaji Institute of Medical Sciences Raipur, India*

## EPV‐1075

### Beyond seizure detection: ICU based cEEG‐guided management of NORSE targeting Ictal–Interictal Continuum and BIRDs – A case series

#### S. Agarwal; D. Khandelwal; A. Vyas

##### Department of Neurology, Sawai Man Singh Hospital, Jaipur, India

## EPV‐1076

### Cerebral edema in ICU patients: Etiological spectrum and mortality predictors

#### S. Biswas ^1^; S. Vasireddy^2^; Y. Srivastava^1^; S. Arora^3^; J. Rajamani^4^; A. Shankar^5^; K. Jindal^6^

##### ^*1*^ *Department of Internal Medicine, Ivano‐Frankivsk National Medical University, Ivano‐Frankivsk, Ukraine;* ^*2*^ *Department of Neurology, NMC Specialty Hospital, Abu Dhabi, United Arab Emirates;* ^*3*^ *Department of Iinternal Medicine, University of Debrecen, Hungary;* ^*4*^ *Department of Iinternal Medicine, Medical College, Caucasus University, Tbilisi, Georgia;* ^*5*^ *Department of Iinternal Medicine, Humanitas University, Milan, Italy;* ^*6*^ *Department of Iinternal Medicine, Government Medical College, Patiala*

## EPV‐1077

### Cerebral EEG activity response to mannitol in acute ischemic malignant stroke

#### R. Urdea ^1^; V. Tiu^2^; A. Daneasa^2^; V. Frunza^1^; G. Bazar^1^; I. Manea^3^; D. Taralunga^3^; G. Neagu^3^; C. Panea^2^

##### ^*1*^ *Neurology Department, Elias Emergency University Hospital, Bucharest, Romania;* ^*2*^ *Neurology Department, Carol Davila University of Medicine and Pharmacy, Bucharest, Romania;* ^*3*^ *Applied Electronics and Information Engineering Department, National University of Science and Technologies Politehnica, Bucharest, Romania*

## EPV‐1078

### Comparative outcomes of toxic versus metabolic encephalopathy in critically Ill adults: A MIMIC‐IV analysis

#### S. Biswas^1^; Y. Srivastava ^1^; S. Vasireddy^2^; S. Arora^3^; J. Rajamani^4^; K. Jindal^5^; R. Falodia^6^; A. Shankar^7^

##### ^*1*^ *Department of Internal Medicine, Ivano‐Frankivsk National Medical University, Ivano‐Frankivsk, Ukraine;* ^*2*^ *Department of Neurology, NMC Specialty Hospital, Abu Dhabi, United Arab Emirates;* ^*3*^ *Department of Internal Medicine, University of Debrecen, Hungary;* ^*4*^ *Department of Internal Medicine Medical College, Caucasus University, Tbilisi, Georgia;* ^*5*^ *Department of Internal Medicine Government Medical College, Patiala, India;* ^*6*^ *Department of Internal Medicine, All India Institute of Medical Sciences, Jodhpur, India;* ^*7*^ *Department of Internal Medicine, Humanitas University, Milan, Italy*

## EPV‐1079

### Delayed anoxic leukoencephalopathy: When recovery isn't the end

#### M. Yousaf; M. Hussain; F. Nauman; L. Tablieh

##### *Virginia Tech Carilion School of Medicine*, *Roanoke; Virginia*

## EPV‐1080

### Determinants of mortality and functional outcomes in neurological intensive care unit patients

#### N. Kahveci Erdemir ^1^; A. Çolakoğlu Çil^1^; A. Aksoy Gündoğdu^1^; İ. Yıldırım^2^

##### ^*1*^ *Department of Neurology, Tekirdag Namik Kemal University, Tekirdag, Türkiye;* ^*2*^ *Department of Anesthesiology and Reanimation, Tekirdag Namik Kemal University, Tekirdag, Türkiye*

## EPV‐1081

### Evaluation of nasogastric tube placement via X‐ray in neurological intensive care patients

#### S. Sayyigit Tekin; H. Gultekin; M. Cevik; M. Aluclu

##### Dicle University Medical School Department of Neurology Diyarbakir Türkiye

## EPV‐1082

### Improving diagnostic accuracy and rehabilitation outcomes in transient ischemic attack and ischemic stroke using the NIRSIT device

#### B. Bakhriev^1^; S. Rakhmonkulov ^2^; D. Khidoyatova^3^

##### ^*1*^ *Neurology, Child Neurology and Medical Genetics Department, Tashkent State Medical University, Uzbekistan;* ^*2*^ *Neurology, Child Neurology and Medical Genetics Department, Tashkent State Medical University, Uzbekistan;* ^*3*^ *Neurology and Traditional Medicine.Tashkent State Medical University, Uzbekistan*

## EPV‐1083

### Intrathecal dexamethasone for FIRES: Therapeutic experience and review of current evidence

#### W. Foong; S. Abu Hassan; Z. Tan; K. Yong; Y. Tan

##### Department of Neurology, National Neuroscience Institute, Singapore

## EPV‐1084

### Non‐convulsive status epilepticus in the neurological intensive care unit: Electro‐clinical outcome predictors

#### V. Radisic ^1^; A. Ristic^1^; M. Ercegovac^1^; B. Ralic^2^; D. Jovanovic^1^; I. Berisavac^1^

##### ^*1*^ *Neurology Clinic University Clinical Center of Serbica, Belgrade, Serbia;* ^*2*^ *Clinical Hospital Center Zvezdara, Belgrade, Serbia*

## EPV‐1085

### Procalcitonin and complications in non‐aneurysmal subarachnoid hemorrhage: A single‐centre cohort study

#### E. Ranxha; O. Çibuku; E. Basha; O. Taka

##### Neuro vascular Service, University Hospital “Mother Teresa” Tirane, Albania

## EPV‐1086

### Prognostic value of routinely assessed cerebrospinal fluid parameters in spontaneous subarachnoid hemorrhage

#### K. Berek; A. Berek; P. Kindl; F. Di Pauli; A. Schiefecker; B. Pfausler; F. Deisenhammer; R. Beer; R. Helbok; V. Rass; H. Hegen

##### Department of Neurology, Medical University of Innsbruck, Innsbruck, Austria

## EPV‐1087

### Validation of entrustable professional activities assessment for neurocritical care advanced practice providers via simulation

#### E. Jung

##### Chonnam National University Hospital, Gwangju, Republic of Korea

## Neuroepidemiology

## EPV‐1088

### Analysis of cerebrovascular disease incidence and seasonal trends: A 10‐year nationwide study in Georgia (2014–2023)

#### G. Matchavariani ^1^; F. Parsayan^2^; D. Tabatadze^1^; M. Kvezereli^1^; G. Chakhava^3^; I. Rukhadze^1^

##### ^*1*^ *Caucasus Medical Centre, Tbilisi, Georgia;* ^*2*^ *Tbilisi State University, Tbilisi, Georgia;* ^*3*^ *Georgian Association of Medical Specialties, Tbilisi, Georgia*

## EPV‐1089

### Association of occupational factors and environmental tobacco smoke with risk of Alzheimer's disease

#### L. Yang

##### Zhejiang Hospital, No. 12, Lingyin Road, Hangzhou, China

## EPV‐1090

### Case‐mix, referral drivers, and admissions in a rapid‐access neurology clinic: A 12‐month evaluation from a middle‐income setting

#### M. Grecco ^1^; M. De Sampaio^1^; S. Caporale^1^; G. Povedano^1^; J. Pozo Putalivo^2^

##### ^*1*^ *Complejo Médico Policial Churruca Visca, Buenos Aires, Argentina;* ^*2*^ *RedSalud Santiago, Santiago de Chile, Chile*

## EPV‐1091

### Clinical and etiological predictors of stroke severity in young adults with ischemic stroke

#### I. Khiyel ^1^; H. Guetteche^1^; L. Rouabah^1^; B. Fekraoui^2^

##### ^*1*^ *Molecular and Cellular Biology Laboratory, University of Brothers Mentouri Constantine 1, Constantine, Algeria;* ^*2*^ *Department of Neurology, University of Salah Boubnider Constantine 3, Constantine, Algeria*

## EPV‐1092

### Comparative epidemiology of optic neuritis before and after the COVID‐19 pandemic: A comprehensive literature review

#### C. Topala ^1^; C. Ferri^2^; N. Merli^2^; V. Lisnic^1^; M. Pugliatti^2^

##### ^*1*^ *IMSP “Diomid Gherman” Institute of Neurology and Neurosurgery, “Nicolae Testemitanu” State University of Medicine and Pharmacy, Chisinau, Republic of Moldova;* ^*2*^ *Department of Neuroscience and Rehabilitation, University of Ferrara, Ferrara, Italy*

## EPV‐1093

### Diagnostic value of nerve growth factor in cognitive impairment in generalized epilepsy

#### R. Fayziyeva

##### Department of Neurology, Tashkent State Medical University, Tashkent, Uzbekistan

## EPV‐1094

### Estimating the prevalence of mild behavioral impairment in a population‐based greek cohort: Findings from the HELIAD study

#### E. Angelopoulou ^1^; S. Charisis^2^; P. Sakka^3^; M. Kosmidis^4^; M. Yannakoulia^5^; E. Dardiotis^6^; G. Hadjigeorgiou^7^; A. Hatzimanolis^8^; S. Papageorgiou^1^; N. Scarmeas^1^

##### ^*1*^ *1st Neurology Department, Aiginition University Hospital, National and Kapodistrian University of Athens, Greece;* ^*2*^ *Glenn Biggs Institute for Alzheimer's and Neurodegenerative Diseases, UT Health San Antonio, TX, USA;* ^*3*^ *Athens Association of Alzheimer's Disease and Related Disorders, Marousi, Greece;* ^*4*^ *Lab of Cognitive Neuroscience, School of Psychology, Aristotle University of Thessaloniki, Greece;* ^*5*^ *Department of Nutrition and Dietetics, Harokopio University, Athens, Greece;* ^*6*^ *Department of Neurology, University Hospital of Larissa, Faculty of Medicine, School of Health Sciences, University of Thessaly, Greece;* ^*7*^ *Department of Neurology, Medical School, University of Cyprus, Nicosia;* ^*8*^ *Department of Psychiatry, National and Kapodistrian University of Athens Medical School, Aiginition Hospital, Greece*

## EPV‐1095

### Impact of war‐related stress on multiple sclerosis relapse in iranian patients: Lessons from a recent war in the Middle East

#### F. Hojjatipour ^1^; M. Salari^2^

##### ^*1*^ *Shahid Beheshti University of Medical Sciences, Tehran, Iran;* ^*2*^ *Shahid Beheshti University of Medical Sciences, Tehran, Iran*

## EPV‐1096

### Incidence, prevalence, and disability associated with neurological disorders in Jordan between 1990 and 2023: An analysis based on the GBD Study 2023

#### M. Al‐Wardat; M. Etoom

##### *Department of Rehabilitation Sciences, Jordan University of Science and Technology*, *Irbid, Jordan*

## EPV‐1097

### Lifestyle and comorbidities for cerebrovascular risk in adults 40–65, Eastern Poland

#### D. Jakubowicz‐Lachowska; M. Bargielski; M. Wierciński; M. Bazyleiwcz; E. Szyłak; M. Chorąży; J. Kochanowicz; A. Kułakowska

##### Department of Neurology, Medical University of Bialystok, Poland

## EPV‐1098

### Long‐term trends in idiopathic epilepsy in India: Evidence from the global burden of disease study, 1990–2023

#### A. Sharma ^1^; P. Kola^2^; A. Paruchuri^2^

##### ^*1*^ *Government Medical College, Omandurar, Chennai, India;* ^*2*^ *All India Institute of Medical Sciences, New Delhi, India*

## EPV‐1099

### Paternal perinatal depression in Pakistan: A mixed‐methods systematic review

#### S. Wasti^1^; A. Wasti^2^; M. Abdullah ^1^; A. Mustafa^1^

##### ^*1*^ *Department of Neurology, King Edward Medical University, Lahore, Pakistan;* ^*2*^ *Department of Neurology, Continental Medical College, Lahore, Pakistan*

## EPV‐1100

### Phenotype distribution and differential disease burden in multiple sclerosis: Real‐world data from the apolo study

#### C. Oreja‐Guevara ^1^; I. González‐Suarez^2^; S. Eichau Madueño^3^; S. Otero Romero^4^; M. Calvo Díez^5^; C. Engroba Teijeiro^6^; A. Casado Gómez^7^; P. Castro Albarrán^7^; J. Meca‐Lallana^8^

##### ^*1*^ *Neurology Department, Hospital Universitario Clínico San Carlos, Madrid, Spain;* ^*2*^ *Neurology Department, Hospital Álvaro Cunqueiro, Vigo, Spain;* ^*3*^ *Neurology Department, Hospital Universitario Virgen Macarena, Seville, Spain;* ^*4*^ *Preventive Medicine and Epidemiology Department, Hospital Universitario Vall d'Hebron, Barcelona, Spain;* ^*5*^ *Health Economics & Pricing Business, Sanofi, Barcelona, Spain;* ^*6*^ *Market Access, Sanofi, Barcelona, Spain;* ^*7*^ *Pharmacoeconomics & Outcomes Research Iberia (PORIB), Madrid, Spain;* ^*8*^ *Neurology Department, Hospital Universitario Clínico Virgen de la Arrixaca, Murcia, Spain*

## EPV‐1101

### Post‐COVID changes in thyroid cancer incidence: Possible neuroendocrine and ACE2‐mediated mechanisms

#### Z. Khalimova; B. Kamildjanova; M. Omiljonov; I. Khakimov

##### Republican Specialized Scientific and Practical Medical Center of Endocrinology named after Acad. E.Kh. Turakulov, Tashkent, Uzbekistan

## EPV‐1102

### Serum S100B protein levels in neurodegenerative diseases

#### J. Tana ^1^; A. Mitre^2^; I. Zekja^1^; J. Kruja^1^

##### ^*1*^ *Neurology Department, UHC Mother Teresa, Tirana;* ^*2*^ *Intermedica Laboratory, Tirana*

## EPV‐1103

### Sex differences in ischemic stroke among young adults: A North African cohort study

#### I. Khiyel ^1^; H. Gutteche^1^; L. Rouabah^1^; B. Fekraoui^2^

##### ^*1*^ *Molecular and Cellular Biology Laboratory, University of Brothers Mentouri Constantine 1, Constantine, Algeria;* ^*2*^ *Department of Neurology, University of Salah Boubnider Constantine 3, Constantine, Algeria*

## EPV‐1104

### Sex‐specific trends in multiple sclerosis burden in India, 1990–2023: A GBD 2023 analysis

#### A. Sharma ^1^; P. Kola^2^; A. Paruchuri^2^

##### ^*1*^ *Government Medical College, Omandurar, Chennai, India;* ^*2*^ *All India Institute of Medical Sciences, New Delhi, India*

## EPV‐1105

### Sociodemographic and clinical profile of an outpatient population with functional neurological disorder over a decade

#### B. Fasching; G. Hennig; C. Mueller; B. Ludwig

##### Department of Neurology, Medical University of Vienna, Vienna, Austria

## EPV‐1106

### Superficial siderosis of the central nervous system: An underdiagnosed cause of hearing loss and progressive ataxia

#### I. Saurina Navarro ^1^; C. Martínez Follana^1^; M. Marín Rubio^1^; E. Campanya Marti^1^; M. García Huguet^1^; B. Alemany Perna^2^; D. López Domínguez^2^

##### ^*1*^ *Department of Neurology, Hospital Doctor Josep Trueta, Girona, Spain;* ^*2*^ *Ataxia Unit – Hospital Doctor Josep Trueta, Girona/Hospital Santa Caterina, Salt, Spain*

## EPV‐1107

### The impact of psychological stress on epileptic seizure frequency during wartime: A study following the “Swords of Iron” War in October 2023

#### A. Eilam; M. Arar; Y. Balash; V. Samogalsky; N. Khmeliov

##### Neurology Department, Kaplan Medical Center, Rechovot, Israel

## Neurogenetics

## EPV‐1108

### A 62‐year‐old woman with hyperorality and apathy drug resistant, A new variant of CHMP2B mutation

#### A. Sangiorgio; C. Manco; D. Righi; S. Bianchi; N. De Stefano; D. Plantone

##### Department of Medicine, Surgery and Neuroscience, University of Siena, Italy

## EPV‐1109

### A novel mutation in the RNF216 gene associated with gordon holmes syndrome: Two Turkish sibling cases

#### Z. Baykan Biyikli; C. Boz

##### Karadeniz Technical University, Department of Neurology, Trabzon, Türkiye

## EPV‐1110

### A recurrent pathogenic MTM1 variant manifesting as hereditary spastic paraplegia (HSP) expands the phenotypic spectrum of X‐linked myotubular myopathy

#### C. Koniari^1^; Z. Kontogeorgiou^1^; N. Ragazos^1^; C. Kartanou^1^; E. Anagnostou^2^; G. Pepe^3^; G. Nasioulas^3^; G. Karadima^1^; G. Koutsis ^1^

##### ^*1*^ *Neurogenetics Unit, 1st Department of Neurology, National and Kapodistrian University of Athens, Eginitio Hospital, Athens, Greece;* ^*2*^ *1st Department of Neurology, National and Kapodistrian University of Athens, Eginitio Hospital, Athens, Greece;* ^*3*^ *Genekor Medical S.A., Athens, Greece*

## EPV‐1111

### Accelerated brain aging and anatomical substrates in spinocerellar ataxia type 3

#### G. Shirui

##### Department of Neurology and Institute of Neurology of First Affiliated Hospital, Institute of Neuroscience, and Fujian Key Laboratory of Molecular Neurology, Fujian Medical University, Fuzhou, China

## EPV‐1112

### Adult‐onset congenital myasthenic syndrome: Genetic variability and clinical phenotype

#### A. Sousa

##### Unidade Local de Saúde de Aveiro, Portugal

## EPV‐1113

### Application of genetic tests in clinical practice: challenges in dealing with atypical phenotypes in neurodegenerative diseases

#### M. Anselmi ^1^; E. Monfrini^2^; A. Di Fonzo^2^; R. Di Giacopo^3^

##### ^*1*^ *Interdepartmental Centre of Medical Science, University of Trento, Trento, Italy;* ^*2*^ *Neurology Unit, Foundation IRCCS Ca' Granda Ospedale Maggiore Policlinico, Milan, Italy;* ^*3*^ *Neurology Unit, Santa Maria del Carmine Hospital, Rovereto, Italy*

## EPV‐1114

### Association study between polymorphisms of the PTH gene and the morphology of the Sella Turcica

#### C. Nagai ^1^; J. Armando Brancher^2^; M. Gonçalves Fauth^1^; E. Calvano Küchler^3^

##### ^*1*^ *Department of Anatomy, Federal University of Paraná, Curitiba, Paraná, Brazil;* ^*2*^ *Postgraduate Program in Cell and Molecular Biology, Federal University of Paraná, Curitiba, Paraná, Brazil;* ^*3*^ *Department of Orthodontics, University of Bonn, Medical Faculty, Bonn, Germany*

## EPV‐1115

### Atypical presentation of spinocerebellar Ataxia 34 associated with a Novel Elovl4 mutation

#### F. Usta; S. Usta; M. Mercan; V. Yayla

##### Neurology Clinics, University of Health Sciences, Dr. Sadi Konuk Training and Research Hospital, Istanbul Türkiye

## EPV‐1116

### Autosomal dominant cerebellar ataxia in siblings with a shared PDYN frameshift variant

#### R. Rodrigues ^1^; M. Rosário^1^; V. Carvalho^1^; L. Correia Guedes^2^

##### ^*1*^ *Neurology, Department of Neurosciences and Mental Health, Unidade Local de Saúde de Santa Maria, Lisbon, Portugal;* ^*2*^ *Centro de Estudos Egas Moniz, Clínica Universitária de Neurologia, Faculty of Medicine, University of Lisbon, Lisbon, Portugal*

## EPV‐1117

### Beyond ALS genes: Identification of causative variants associated with neurodegenerative disorders in Greek ALS patients

#### C. Kartanou^1^; V. Eliades^1^; Z. Kontogeorgiou^1^; N. Ragazos^1^; T. Loupis^2^; D. Vrachnos^2^; I. Spyropoulou^1^; M. Petraki^1^; C. Koniari^1^; S. Aristeidou^3^; E. Koropouli^3^; A. Daponte^3^; V. Zouvelou^3^; M. Rentzos^3^; E. Kapaki^3^; M. Panas^1^; P. Makrythanasis^4^; L. Stefanis^3^; G. Koutsis ^1^; G. Karadima^1^

##### ^*1*^ *Neurogenetics Unit, 1st Department of Neurology, National and Kapodistrian University of Athens, Eginitio Hospital, Athens, Greece;* ^*2*^ *Hematology Research Lab, Clinical, Experimental and Translational Research Center, Biomedical Research Foundation, Academy of Athens, Athens, Greece;* ^*3*^ *1st Department of Neurology, National and Kapodistrian University of Athens, Eginitio Hospital, Athens, Greece;* ^*4*^ *Laboratory of Medical Genetics, Medical School, National and Kapodistrian University of Athens, St. Sophia's Children's Hospital, Athens, Greece*

## EPV‐1118

### CADASIL atypical motor presentation

#### M. Seco ^1^; F. Assis Jacinto^1^; A. André^2^; A. Ferreira^3^; P. Salgado^4^; S. Duarte^4^

##### ^*1*^ *Neurology Department, Hospital Pedro Hispano, Portugal;* ^*2*^ *Neurology Department, Faro Hospital, Faro, Portugal; Neurology Department, Horta Hospital, Portugal;* ^*3*^ *Neurology Department, Portuguese Institute of Oncology of Porto*, *Porto, Portugal; Neurology Department, Horta Hospital, Portugal;* ^*4*^ *Neurology Department, Hospital Pedro Hispano; Neurology Department, Horta Hospital, Portugal*

## EPV‐1119

### Challenges in diagnosing pediatric neurodegeneration with brain iron accumulation: A case report

#### A. Condrea ^1^; D. Secu^2^; N. Balica^2^; I. Istratuc^1^; S. Hadjiu^3^; V. Sacara^2^

##### ^*1*^ *Pediatrics Clinic, Mother and Child Institute, Chisinau, Republic of Moldova;* ^*2*^ *Human Molecular Genetics Laboratory, Mother and Child Institute, Chisinau, Republic of Moldova;* ^*3*^ *Nicolae Testemițanu State University of Medicine and Pharmacy, Chisinau, Republic of Moldova*

## EPV‐1120

### Clinical and genetic characterization of a Portuguese cohort with familial multiple cavernous malformations

#### T. Morais ^1^; C. Silva^2^; A. Santos^3^; P. Vicente^4^; M. Almeida^3^; I. Santana^4^; J. Durães^4^

##### ^*1*^ *Neurology Department, Divino Espírito Santo Hospital, Ponta Delgada, Azores, Portugal;* ^*2*^ *Neurology Department, Local Health Unit of Leiria, Leiria, Portugal;* ^*3*^ *Center for Neuroscience and Cell Biology, University of Coimbra, Coimbra, Portugal;* ^*4*^ *Neurology Department, Local Health Unit of Coimbra, Coimbra, Portugal*

## EPV‐1121

### Decoding Parkinson's genetics: Causal mutations, risk factors, and clinical phenotypes

#### G. Frías Reyes ^1^; E. González Vilchis^2^; L. Adalid Peralta^3^

##### ^*1*^ *Multidisciplinary Academic Division of Comalcalco DAMC‐UJAT, Comalcalco, Mexico;* ^*2*^ *Faculty of Medicine, National Autonomous University of Mexico (UNAM), Ciudad de México, México;* ^*3*^ *Laboratorio de Reprogramación Celular, Instituto de Fisiología, National Autonomous University of Mexico (UNAM), en el Instituto Nacional de Neurología y Neurocirugía, Ciudad de México, México*

## EPV‐1122

### Defining a rare mtDNA phenotype: MELAS‐like presentation and status epilepticus linked to MT‐TT m.15933G>A

#### S. Chowdhury; M. Tiet

##### Neurology, University Hospitals Leicester NHS Foundation Trust, Derby, UK

## EPV‐1123

### Determination of genotype‐phenotype characteristics in 194 cases with hereditary ataxia

#### M. Gultekin

##### *Erciyes University Faculty of Medicine, Department of Neurology, Kayseri*, *Türkiye*

## EPV‐1124

### Early‐onset parkinsonism associated with intermediate CAG/CAA expansion in the TBP gene

#### G. Soares ^1^; S. Moreira^1^; J. Oliveira^2^

##### ^*1*^ *Department of Neurology, Hospital Pedro Hispano, Matosinhos, Portugal;* ^*2*^ *Centre for Predictive and Preventive Genetics, IBMC – Instituto de Biologia Molecular e Celular, University of Porto, Porto, Portugal*

## EPV‐1125

### Epileptic and developmental encephalopathy associated with wdr45 mutation

#### I. Istratuc ^2^; C. Calcii^1^; A. Condrea^1^; E. Capestru^1^; I. Calistru^3^; M. Sprincean^3^; S. Hadjiu^1^

##### ^*1*^ *Nicolae Testemitanu State University of Medicine and Pharmacy, Chisinau, Republic of Moldova;* ^*2*^ *PHMI Mother and Child Institute, Chisinau, Republic of Moldova;* ^*3*^ *Laboratory of Neurobiology and Medical Genetics, Nicolae Testemițanu State University of Medicine and Pharmacy, Chișinău, Republic of Moldova*

## EPV‐1126

### Evaluation of inflammatory and neurotrophic gene expression profiles in the epileptogenesis process

#### H. Soytürk^1^; P. Bakkal ^2^; T. Bakkal^3^; Ü. Kılıç^4^; Ş. Aydın Türkoğlu^2^

##### ^*1*^ *Interdisciplinary PhD Program in Neuroscience, Bolu Abant İzzet Baysal University, Bolu, Türkiye;* ^*2*^ *Department of Neurology, Bolu Abant İzzet Baysal University Faculty of Medicine, Bolu, Türkiye;* ^*3*^ *Department of Neurology, Izzet Baysal State Hospital, Bolu, Türkiye;* ^*4*^ *First and Emergency Aid Program, Vocational School of Health Services, Duzce University, Duzce, Türkiye*

## EPV‐1127

### Exploring the clinical spectrum of LRSAM1‐related disorder: A Greek case series

#### C. Koniari^1^; Z. Kontogeorgiou^1^; C. Tzembetzis^1^; C. Kartanou^1^; E. Anagnostou^2^; V. Mouratoglou^3^; P. Kokotis^2^; V. Zouvelou^2^; N. Fakas^3^; Z. Lygerou^4^; M. Rentzos^2^; L. Stefanis^2^; G. Karadima^1^; G. Koutsis ^1^

##### ^*1*^ *Neurogenetics Unit, 1st Department of Neurology, National and Kapodistrian University of Athens, Eginitio Hospital, Athens, Greece;* ^*2*^ *1st Department of Neurology, National and Kapodistrian University of Athens, Eginitio Hospital, Athens, Greece;* ^*3*^ *Department of Neurology; 401 General Military Hospital of Athens, Athens, Greece;* ^*4*^ *Molecular Genetics Unit, Department of General Biology, School of Medicine, University of Patras, Patras, Greece*

## EPV‐1128

### Fabry's disease: Rare symptom in a rare disease

#### S. Bourokba; N. Toubal; M. Louanchi

##### Department of Neurology CHU Annaba, Annaba, Algeria

## EPV‐1129

### Fragile X–associated tremor/ataxia syndrome (FXTAS) with mosaicism in genetic testing and different clinical course in two female siblings

#### E. Zeis; P. Kalligerou; A. Daskalaki; C. Koros; V. Konstantinidis; N. Skarmeas; S. Papageorgiou

##### 1st Department of Neurology,Aiginiteio University Hospital, Athens, Greece

## EPV‐1130

### Genetic evaluation of hereditary ataxia patients in Türkiye using targeted next‐generation sequencing: The impact of consanguinity

#### E. Soker ^1^; D. Yorulmaz^1^; D. Agirbasli^2^; A. Kalayci^2^

##### ^*1*^ *Istanbul University‐Cerrahpasa, Cerrahpasa Faculty of Medicine, Istanbul, Türkiye;* ^*2*^ *Department of Medical Genetics, Istanbul University‐Cerrahpasa, Cerrahpasa Faculty of Medicine, Istanbul, Türkiye*

## EPV‐1131

### Genetic risk factors in neurodegenerative parkinsonism

#### T. Strnadová ^1^; K. Menšíková^1^; K. Kolaříková^2^; R. Vodička^2^; P. Kaňovský^1^

##### ^*1*^ *Department of Neurology and Clinical Neuroscience Center, Faculty of Medicine and Dentistry, Palacky University, Olomouc, Czech Republic;* ^*2*^ *Department of Clinical and Molecular Genetics, Faculty of Medicine and Dentistry, Palacky University, Olomouc, Czech Republic*

## EPV‐1132

### Genetic spectrum and phenotypic heterogeneity in Parkinson's disease patients undergoing whole‐exome sequencing in a tertiary referral center

#### A. Alexandratou ^1^; G. Vatselas^2^; R. Antonellou^1^; A. Simitsi^1^; N. Papagiannakis^1^; C. Koros^1^; E. Sfikas^1^; I. Alefanti^1^; M. Maniati^2^; K. Lourentzos^3^; A. Bonakis^3^; P. Makrythanasis^4^; L. Stefanis^1^

##### ^*1*^ *First Dept of Neurology, NKUA Medical School, Athens, Greece;* ^*2*^ *BRFAA, Greece;* ^*3*^ *Second Dept of Neurology, NKUA Medical School, Athens, Greece;* ^*4*^ *Laboratory of Medical Genetics, NKUA Medical School, Athens, Greece*

## EPV‐1133

### Hemiplegic migraine with epileptic seizures and reversible cortical abnormalities in a male adolescent: A case report

#### A. Baz Cárdenas

##### Barbara Gutierrez Ruano, Hospital Central de la Defensa Gómez Ulla, Madrid, Spain

## EPV‐1134

### Hypercapnic respiratory failure in familial cavernous cavernoma: Neurogenetic diagnostic criteria and significance of lesion progression – A rare case

#### T. Balraj Singh ^1^; C. Chan^1^; K. Teo^2^

##### ^*1*^ *Department of Medicine, National University Hospital, Singapore;* ^*2*^ *Division of Neurology, Department of Medicine, National University Hospital, Singapore*

## EPV‐1135

### Identification of genetic modifiers in adult Moyamoya disease using whole‐exome sequencing

#### J. Kim ^1^; J. Jeon^2^

##### ^*1*^ *Department of Neurosurgery, Seoul National University College of Medicine, Seoul, Republic of Korea;* ^*2*^ *Department of Neurosurgery, Hallym University College of Medicine, Chuncheon‐si, Gangwon‐do, Republic of Korea*

## EPV‐1136

### Inflammation‐related genetic variants and copy number changes in Alzheimer's disease in a Saudi cohort

#### L. Sorour ^1^; M. Alkhateeb^3^; N. Qadi^1^; M. Al Rajeh^2^; F. El Bitar^1^

##### ^*1*^ *King Faisal Specialist Hospital and Research Center (KFSHRC), Riyadh, Kingdom Saudi Arabia;* ^*2*^ *Al Habib Medical Center, Riyadh, Kingdom Saudi Arabia;* ^*3*^ *Princess Nourah bint Abdulrahman University (PNU), Riyadh, Kingdom Saudi Arabia*

## EPV‐1137

### Late presentation of megalencephalic leukoencephalopathy with subcortical cysts (MLC) in a female patient with ataxia, dystonia and seizure

#### I. Sarac ^1^; F. Borovecki^1^; H. Sarac^2^

##### ^*1*^ *University of Zagreb, Croatia;* ^*2*^ *University Hospital Center Zagreb, Croatia*

## EPV‐1138

### Machine learning applications in Huntington's disease genomics: A systematic review

#### G. Uzoechina; T. Osajiuba

##### Faculty of Clinical Sciences, University of Nigeria Teaching Hospital, Enugu, Nigeria

## EPV‐1139

### Measuring functional abilities and assistance level in niemann‐pick disease type C, GM2 gangliosidoses, and ataxia

#### B. Zanrucha ^1^; M. Patterson^1^; J. Kerthi^1^; J. Raymond^1^; A. Hatcher^1^; M. Grosso^1^; J. Caines^2^; S. Rounds^2^

##### ^*1*^ *IntraBio Inc., Austin, Texas, USA;* ^*2*^ *Curant Health LLC., Smyrna, Georgia, USA*

## EPV‐1140

### Mitochondrial encephalomyopathy, lactic acidosis, and stroke‐like episodes: A case series

#### M. Majoul; Y. Memmich; D. Ben Mohamed; R. Zouari; A. Rachdi; Z. Saied; F. Nebli; S. Ben Sassi

##### Department of Neurology, National Institute Mongi Ben Hmida of Neurology, Tunis, Tunisia

## EPV‐1141

### Molecular diagnosis of trinucleotide repeat expansion disorders in Singapore (1996–2024)

#### A. Raheja ^1^; K. Narasimhalu^1^; H. Law^2^

##### ^*1*^ *National Neuroscience Institute (SGH Campus), Singapore;* ^*2*^ *KK Women's and Children's Hospital, Singapore*

## EPV‐1142

### Neuromuscular manifestations in patients with lipodystrophy

#### N. Varga ^1^; Z. Grosz^1^; A. Takacs^2^; P. Reismann^3^; A. Jobbagy^4^; V. Molnar^1^; M. Molnar^1^

##### ^*1*^ *Institute of Genomic Medicine and Rare Disorders, Semmelweis University, Budapest, Hungary;* ^*2*^ *Department of Ophthalmology, Semmelweis University, Budapest, Hungary;* ^*3*^ *Department of Internal Medicine and Oncology, Semmelweis University, Budapest, Hungary;* ^*4*^ *Department of Dermatology, Dermatooncology and Venerology, Semmelweis University, Budapest, Hungary*

## EPV‐1143

### Peak expiratory flow causally affects brain neuronal activity: A Mendelian randomization study

#### Y. Mao; R. Wu

##### Department of Radiology, Second Affiliated Hospital, Shantou University Medical College, Shantou, Guangdong, China

## EPV‐1144

### Persistent sleep disturbance in youth: A clue to Wilson disease

#### V. Mouratoglou ^1^; N. Papageorgiou^1^; M. Thomas^1^; I. Vamvakaris^1^; G. Pepe^3^; G. Nasioulas^3^; A. Simitsi^2^; C. Koniari^1^; L. Stefanis^2^; N. Fakas^1^

##### ^*1*^ *Neurology Department; 401 General Military Hospital of Athens, Athens, Greece;* ^*2*^ *1st Department of Neurology, National and Kapodistrian University of Athens, Eginitio Hospital, Athens, Greece;* ^*3*^ *Genekor Medical S.A, Athens, Greece*

## EPV‐1145

### Progressive external ophthalmoplegia due to a POLG gene mutation: A case report

#### C. Segura Sanz

##### Department of Neurology, University Hospital Virgen de la Victoria, Málaga

## EPV‐1146

### Progressive spastic paraparesis misdiagnosed as cerebral palsy: a case of ARSACS

#### M. Nezval ^1^; T. Dorňák^1^; L. Satke^1^; M. Nevrlý^1^; L. Osičková^2^; P. Kaňovský^1^

##### ^*1*^ *Department of Neurology and Clinical Neuroscience Center, Faculty of Medicine and Dentistry, Palacky University, Olomouc, Czech Republic;* ^*2*^ *Department of Clinical and Molecular Genetics, Faculty of Medicine and Dentistry, Palacky University, Olomouc, Czech Republic*

## EPV‐1147

### Pseudodemyelinating MRI changes in neurofibromatosis type 1: Clinical significance of UBO/FASI

#### K. Kovalchuk; T. Nehrych; M. Shorobura; N. Nehrych; N. Bozhenko; Y. Matviyenko

##### Department of Neurology, Danylo Halytsky Lviv National Medical University, Lviv, Ukraine

## EPV‐1148

### Rare case of neuromuscular oculoauditory syndrome: Genetic challenges

#### A. Frontistis ^1^; S. Kalampokini^1^; D. Goulis^2^; G. Pepe^3^; S. Koukoula^4^; M. Moschou^1^; M. Arnaoutoglou^1^; V. Papaliagkas^5^; V. Kimiskidis^1^

##### ^*1*^ *First Department of Neurology, AHEPA University Hospital, Aristotle University of Thessaloniki;* ^*2*^ *Unit of Reproductive Endocrinology, First Department of Obstetrics‐Gynaecology, Aristotle University of Thessaloniki;* ^*3*^ *Genekor Medical S.A., Athens, Greece;* ^*4*^ *Vitreoretinal Department, Ophthalmica Eye Institute, Thessaloniki, Greece;* ^*5*^ *Department of Biomedical Sciences, School of Health Sciences, International Hellenic University, Thessaloniki, Greece*

## EPV‐1149

### RORA‐Related IDDECA with cerebellar atrophy: Marked clinical response to valproate in an adult patient

#### V. Busco ^1^; G. Di Lazzaro^2^; A. Cimmino^1^; M. Petracca^2^; D. Genovese^2^; A. De Biase^2^; F. Musso^1^; A. Calarco^1^; P. Calabresi^2^; A. Bentivoglio^2^

##### ^*1*^ *Catholic University of the Sacred Heart, Rome, Italy;* ^*2*^ *Neurology Unit, Fondazione Policlinico Universitario Agostino Gemelli IRCCS, Rome, Italy*

## EPV‐1150

### Transcranial alternating current stimulation for treatment of multiple system atrophy cerebellar type: A randomized controlled trial

#### G. Shirui

##### Department of Neurology and Institute of Neurology of First Affiliated Hospital, Institute of Neuroscience, and Fujian Key Laboratory of Molecular Neurology, Fujian Medical University, Fuzhou, China

## EPV‐1151

### VPS16‐dystonia linked pathogenic variant in Parkinson's disease: Expanding the phenotype?

#### M. Ostrožovičová ^1^; T. Lorincova^1^; R. Jech^2^; M. Zech^3^; M. Skorvanek^1^

##### ^*1*^ *Department of Neurology, P.J. Safarik University, and University Hospital of L. Pasteur, Kosice, Slovakia; Laboratory of Clinical Neurosciences, University Science Park MEDIPARK, P. J. Safarik University, Kosice, Slovakia;* ^*2*^ *Department of Neurology and Center of Clinical Neuroscience, First Faculty of Medicine Charles University and General University Hospital in Prague, Prague, Czech Republic;* ^*3*^ *Institute of Human Genetics, School of Medicine and Health, Technical University of Munich, Munich, Germany. Institute of Neurogenomics, Helmholtz Zentrum München, Institute for Advanced Study, Technical University of Munich, Munich, Germany*

## Neuroimaging

## EPV‐1152

### AI in healthcare: The potential of ChatGPT as an assisting tool in diagnosing stroke

#### N. Abdelmaksoud^1^; H. Hussien ^2^; M. Mamdouh^3^; Y. Fathy^4^; N. Eldmrdash^5^; A. Gamal^6^; H. Elsharkawy^7^; M. Hossam^8^

##### ^*1*^ *Neurology Department, Misr University for Science and Technology, Giza, Egypt;* ^*2*^ *5th year student, Faculty of Medicine, Mansoura University, Mansoura, Egypt;* ^*3*^ *5th year student, Faculty of Medicine, Sohag University, Sohag, Egypt;* ^*4*^ *3rd year student, Faculty of Medicine, Mansoura National University, Mansoura, Egypt;* ^*5*^ *5th year student, Faculty of Medicine, Delta University, Mansoura, Egypt;* ^*6*^ *Medical intern, Sohag University Hospital, Sohag, Egypt;* ^*7*^ *Physical Therapy intern, Mansoura University Hospital, Kafr‐Elshiekh, Egypt;* ^*8*^ *5th year student Faculty of Physical Therapy, October 6th University, Giza, Egypt*

## EPV‐1153

### Beyond the classical triad: Cerebral and spinal cord involvement in Kearns‐Sayre syndrome

#### A. Correia ^1^; D. Cruz^1^; C. Cunha^2^; A. Antunes^1^; L. Albuquerque^1^; V. Carvalho^1^

##### ^*1*^ *Neurology Department, Department of Neurosciences and Mental Health, Santa Maria Hospital, Lisbon, Portugal;* ^*2*^ *Neuroradiology Department, Department of Neurosciences and Mental Health, Santa Maria Hospital, Lisbon, Portugal*

## EPV‐1154

### Changes in brain morphometry in post COVID‐19 patients: An exploratory study

#### S. Panda ^1^; R. Naik^1^; S. Tiwari^2^; G. Bohra^3^; P. Khera^2^

##### ^*1*^ *Department of Neurology All India Institute of Medical Sciences, Jodhpur, India;* ^*2*^ *Department of Diagnostic and Interventional Radiology All India Institute of Medical Sciences, Jodhpur, India;* ^*3*^ *Department of Medicine All India Institute of Medical Sciences, Jodhpur, India*

## EPV‐1155

### CHANTER syndrome: An emerging diagnosis in toxic encephalopathy

#### I. Córdoba Bueno; M. Mesa Hernández; M. Jimenez Arenas; P. Blanco Ramírez; M. García Falcón; J. Gómez Moreno; M. Molina López; A. Parejo Olivera; A. Roa Montero

##### Department of Neurology, Badajoz University Hospital, Badajoz, Spain

## EPV‐1156

### Characterizing the cellular distribution of iron in Parkinson's disease from MRI data

#### A. Oliveira^2^; Q. Raynaud^1^; M. Castro Jiménez^3^; C. Hubsch ^3^; E. Accolla^4^; J. Bally^3^; A. Lutti^1^

##### ^*1*^ *Laboratory for Research in Neuroimaging, Department of Clinical Neuroscience, Lausanne University Hospital and University of Lausanne, Lausanne, Switzerland;* ^*2*^ *Department of Radiology, Lausanne University Hospital (CHUV), Lausanne, Switzerland;* ^*3*^ *Service of Neurology, Department of Clinical Neuroscience, Lausanne University Hospital (CHUV) and University of Lausanne (UNIL), Lausanne, Switzerland;* ^*4*^ *Service de Neurology, HFR – Hôpital Cantonal Fribourg, Fribourg, Switzerland*

## EPV‐1157

### Clinical and radiological features of autoimmune encephalitis: A monocentric study from Tunisia

#### O. Sakli; R. Zouari; Z. Saied; A. Rachdi; D. Ben Mohamed; F. Nabli; L. Bouafia; S. Ben Sassi

##### Department of Neurology, Mongi Ben Hamida Hospital, Tunis, Tunisia

## EPV‐1158

### Clinical utility of DaTscan in detecting neurodegenerative causes of Parkinsonism

#### A. Teo ^1^; K. Teo^1^; S. Ng^1^; C. Tan^2^

##### ^*1*^ *Division of Neurology, Department of Medicine, National University Hospital, Singapore;* ^*2*^ *Division of Neurology, Department of Medicine, Alexandra Hospital, Singapore*

## EPV‐1159

### Correlation between FDG and amyloid‐beta PET uptake in patients with neurodegenerative diseases

#### M. Bonacci; M. Caligiuri; C. Calomino; R. Nisticò; M. Bianco; V. Prigitano; A. Quattrone; A. Quattrone; F. Novellino

##### Neuroscience Research Center, University “Magna Graecia”, Catanzaro, Italy; Department of Medical and Surgical Sciences, University “Magna Graecia”, Catanzaro, Italy

## EPV‐1160

### Deep learning–based synthesis of [11C]PiB MicroPET images from [18F]FDF in an Alzheimer's disease animal model

#### M. Becerril

##### Universidad Nacional Autónoma de México, Mexico

## EPV‐1161

### Diencephalo‐mesencephalic Junction Dysplasia Type B: Diagnostic challenges and clinico‐imaging correlations

#### M. Ursoi ^1^; M. Sangheli^1^; G. Corcea^2^

##### ^*1*^ *University of Medicine and Pharmacy “Nicolae Testemiţanu”, Chișinău, Republic of Moldova;* ^*2*^ *Institute of Neurology and Neurosurgery “Diomid Gherman”, Chișinău, Republic of Moldova*

## EPV‐1162

### Differentiating neurovascular conflict in classical trigeminal neuralgia: A 15‐year study on the use of multispiral computed tomographic angiography

#### O. Zykova; E. Balyazina; A. Afanasyeva

##### Rostov State Medical University, Rostov‐on‐Don, Russian Federation

## EPV‐1163

### Distinct cerebral perfusion patterns in drug‐induced parkinsonism versus Parkinson's disease: A dual‐phase 18F‐FP‐CIT PET analysis

#### E. Jung

##### Chonnam National University Hospital, Gwangju, Republic of Korea

## EPV‐1164

### Enlarged perivascular spaces alongside white matter hyperintensities predict poor MoCA outcomes among elderly individuals

#### M. Abdi ^1^; K. Hassaninia^1^; M. Almasi‐Dooghaee^2^; F. Akhoundi^2^

##### ^*1*^ *Medicine Faculty, Iran University of Medical Sciences, Tehran, Iran;* ^*2*^ *Neurology department, Iran University of Medical sciences, Tehran, Iran*

## EPV‐1165

### Evaluation of the correlation of neuromelanin loss in the Substantia Nigra on MRI

#### S. Aydeniz; S. Özkaynak; K. Karaali

##### Neurology, Akdeniz University/Antalya, Türkiye

## EPV‐1166

### Fahr disease diagnosed in an elderly patient: A puzzling clinical case

#### A. Quka ^1^; I. Zekja^1^; E. Ranxha^2^; A. Hoxha^1^; A. Kuqo^1^

##### ^*1*^ *Department of Neurology, UHC Mother Teresa, Tirana, Albania;* ^*2*^ *Stroke Unit, UHC Mother Teresa, Tirana, Albania*

## EPV‐1167

### FDG‐PET in Creutzfeldt‐Jakob disease presenting as corticobasal syndrome

#### A. Vătămănelu ^1^; I. Ion^1^; B. Chambert^2^; G. Taieb^1^; G. Castelnovo^1^; D. Renard^1^

##### ^*1*^ *Department of Neurology, CHU Nîmes, Univ. Montpellier, France;* ^*2*^ *Department of Nuclear Medicine, CHU Nîmes, Univ. Montpellier, France*

## EPV‐1168

### Hypertrophic olivary degeneration secondary to venous infarction: a previously unreported presentation

#### M. Bonnin Bravo; M. García Ruiz; E. López Valdés; M. Álvarez Gutiérrez; L. Domínguez Jiménez; Á. Lafuente Almadén; J. Melgar Jara; P. Mayo Rodríguez

##### Neurology Department, Hospital Clínico San Carlos, Madrid, Spain

## EPV‐1169

### Hypomagnesemia‐associated cerebellar syndrome: A reversible cause of acute ataxia

#### M. Filipa Graça; A. Ferreira; M. Calejo; S. Duarte

##### Neurology Department, Unidade Local de Saúde Matosinhos, Matosinhos, Portugal

## EPV‐1170

### Improving diagnostic sensitivity for temporal lobe epilepsy by using deep learning‐based MRI reconstruction

#### D. Jung

##### Ajou University School of Medicine, Suwon, Republic of Korea

## EPV‐1171

### Inflammatory cerebral amyloid angiopathy: Report of two clinical cases

#### M. Ruiz Perelló; F. Salazar Hernandez; B. Gómez González; D. Lopez Segura; A. Maija Savolainenhg; M. Lorente; A. Sancho; A. Gomez Esteban; D. Vidal Mena; M. Lopez Lopez; E. Fages Caravaca; M. Ortega Ortega; M. Ruiz Perelló

##### Department of Neurology, Santa Lucía University Hospital, Cartagena, Spain

## EPV‐1172

### Intermittent diplopia revealing an aneurysmal persistent trigeminal artery

#### A. Kalfat ^1^; I. Dkhil^2^; S. Jelassi^2^; A. Ben Lakhal^2^; N. Hammami^2^; S. Nagi^2^

##### ^*1*^ *LR18SP04 and Pediatric Neurology Department, National Mongi Ben Hmida Institute of Neurology, Tunis, Tunisia;* ^*2*^ *Neuroradiology Department, National Mongi Ben Hmida Institute of Neurology, Tunis, Tunisia*

## EPV‐1173

### Intravenous thrombolysis in wake‐up and unknown‐onset ischemic stroke selected by CT‐based imaging: A systematic review and meta‐analysis

#### H. Atwan

##### Faculty of Medicine, Assiut University, Assiut, Egypt

## EPV‐1174

### Intravenous thrombolysis in wake‐up and unknown‐onset ischemic stroke selected by MRI‐based imaging: A systematic review and meta‐analysis

#### H. Atwan

##### Faculty of Medicine, Assiut University, Assiut, Egypt

## EPV‐1175

### Longitudinal neuroimaging evidence of cognitive changes in essential tremor

#### C. Calomino; A. Quattrone; M. Bianco; R. Nisticò; M. Bonacci; V. Prigitano; F. Novellino

##### Neuroscience Research Center, Department of Medical and Surgical Sciences, Magna Graecia University of Catanzaro, Italy

## EPV‐1176

### Neuroimaging spectrum of central nervous system tuberculosis – A retrospective cohort study

#### A. Pathirage; V. Velummylum; P. Ruwanpathirana; B. Senanayake

##### Institute of Neurology, National Hospital, Sri Lanka

## EPV‐1177

### Not everything is as it seems: Pseudo–midbrain atrophy on neuroimaging

#### E. Gismera Fontes; M. González Sánchez; F. Sánchez García; M. González Gómez; L. Cabral Esquea

##### Neurology Department, University Hospital Guadalajara, Guadalajara, Spain

## EPV‐1178

### Persistent trigeminal artery causing transient oculomotor nerve palsy: A neuroimaging case

#### O. Cibuku ^1^; V. Thimjo^1^; E. Xhukellari^1^; A. Rroji^2^; E. Ranxha^1^

##### ^*1*^ *Neuro vascular Service, University Hospital “Mother Teresa” Tirane, Albania;* ^*2*^ *Neuro radiology Department, University Hospital “Mother Teresa” Tirane, Albania*

## EPV‐1179

### Preserved global topology with frontoparietal control–centered cross‐network coupling alterations after carbon monoxide poisoning

#### Y. Li ^1^; T. Wang^2^

##### ^*1*^ *Lanzhou University, Lanzhou, China;* ^*2*^ *The First Hospital of Lanzhou University, Lanzhou, China*

## EPV‐1180

### pyogenic cerebellar abscesses leading to ataxia and intractable hiccups

#### A. Elsaddig

##### Neurology Department, Sheikh Tahnoon Bin Mohammed Medical City, Al Ain, United Arab Emirates

## EPV‐1181

### Radscale score and clinical response to tap‐test in patients with normal pressure hydrocephalus

#### J. Camacho Velasquez; B. Lobo Minuve; E. Franquet Gomez; M. Bea Sintes; V. Adell Ortega; P. Bermell Campos

##### Department Of Neurology. Consorci Sanitari Alt‐Penedes‐Garraf. Barcelona, Spain

## EPV‐1182

### Reversible splenial lesion syndrome after fentanyl exposure: Expanding the etiological spectrum

#### P. Nieto Palomares^1^; M. El Harmochi Doud^2^; P. Gómez Ramírez ^2^; A. García Maruenda^3^; I. Martín Sobrino^4^; L. Quirós Illán^6^; A. Sánchez Gómez^2^; A. Herrera Ortega^2^

##### ^*1*^ *Neurology, Neurotraumatological Hospital of Jaén, Jaén, Spain;* ^*2*^ *Neurology, University General Hospital of Ciudad Real, Ciudad Real, Spain;* ^*4*^ *Neurology, Algeciras Hospital, Algeciras, Spain;* ^*5*^ *Neurology, Clinic Hospital, Barcelona, Spain;* ^*6*^ *Neurology, University Infanta Leonor Hospital, Madrid, Spain*

## EPV‐1183

### Safety and feasibility of non‐contrast follow‐up magnetic resonance imaging in clinically stable multiple sclerosis patients

#### J. García Granado ^1^; E. Santana Suárez^2^; M. Pérez García^3^; J. de la Nuez González^1^; M. Pérez Vieitez^1^; M. Quintana García^2^; V. Serrano Romero‐De Ávila^2^; Á. Hernández Pérez^2^; M. Genescà Gómez^2^; A. Peñate Medina^2^; R. García García^2^; V. González Pérez^2^; S. Dorta Gámez^2^; L. Ramos Gil^2^; F. Cabrera Naranjo^1^; A. González Hernández^1^

##### ^*1*^ *Neurology Department, Hospital Universitario de Gran Canaria Doctor Negrín, Las Palmas de Gran Canaria, Spain;* ^*2*^ *Radiology Department, Complejo Hospitalario Universitario Insular Materno Infantil, Las Palmas de Gran Canaria, Spain;* ^*3*^ *Primary Care, Las Palmas de Gran Canaria, Spain*

## EPV‐1184

### Sensory‐onset multiple sclerosis with posterior column lesions: Multivariate predictors of long‐term disability

#### Y. Koubci

##### Mohamed Seghir Nekkache Hospital, Algeria

## EPV‐1185

### Simulation‐assisted trans‐interpeduncular approach for resection of a midline mesencephalic cavernous malformation

#### K. Watanabe^1^; S. Madireddy ^2^; K. Ishikawa^1^; K. Tomoto^1^; T. Saito^1^; Y. Murayama^1^

##### ^*1*^ *Department of Neurosurgery, The Jikei University School of Medicine, Tokyo, Japan;* ^*2*^ *Department of Neurology, The University of Tokyo School of Medicine, Tokyo, Japan*

## EPV‐1186

### Subclavian steal due to fibromuscular dysplasia: A rare neurovascular challenge

#### D. Nicula; A. Pavel; A. Chesnoiu; L. Marinescu; S. Stefan; A. Ribigan; F. Antochi; C. Tiu

##### Neurology, University Emergency Hospital Bucharest, Bucharest, Romania

## EPV‐1187

### Subtentorial Subdural Empyema: A case report

#### I. Bermejo Casado; M. Vargas Cobos; A. De Luca; R. Alfonso González; M. Capra; C. Gómez López de San Román; L. Caballero Sánchez; D. Cerdán Santacruz; A. Castrillo Sanz; A. Mendoza Rodriguez

##### Neurology, Hospital General Universitario de Segovia, Segovia, Spain

## EPV‐1188

### The bloomy rind sign: The first case of ovarian cancer‐associated leptomeningeal carcinomatosis with bloomy rind sign

#### K. Petrosyan ^1^; A. Maralyan^2^; S. Voskanyan^1^

##### ^*1*^ *Department of general and vascular neurology, Saint Gregory the Illuminator Medical Center, Yerevan, Armenia;* ^*2*^ *Diagnostic Department, MRI Service, Saint Gregory the Illuminator Medical Center, Yerevan, Armenia*

## EPV‐1189

### The Medusa's spasm

#### J. Rodrigues Barros ^1^; A. Araújo e Silva^1^; M. Pereira^2^; E. Caldeira^3^; J. Cordeiro Matos^4^; R. Mendes Franco^2^; J. Gonçalves^1^; H. Mota Dória^1^; T. Aguiar^2^

##### ^*1*^ *Neuroradiology Department, Hospital Central do Funchal, SESARAM, EPERAM, Funchal, Portugal;* ^*2*^ *Neurology Department, Hospital Central do Funchal, SESARAM, EPERAM, Funchal, Portugal;* ^*3*^ *Internal Medicine Department, Hospital Central do Funchal, SESARAM, EPERAM, Funchal, Portugal;* ^*4*^ *Physical Medicine and Rehabilitation Department, Hospital Central do Funchal, SESARAM, EPERAM, Funchal, Portugal*

## EPV‐1190

### The use of dual‐energy computed tomography in ischemic stroke patients after endovascular thrombectomy

#### M. Tsalta‐Mladenov ^1^; V. Dimitrova‐Kirilova^2^; T. Drenski^1^; M. Leonidova^3^; M. Kondakova^3^; E. Kalchev^3^; G. Todorov^3^; C. Bachvarov^3^

##### ^*1*^ *Department of Neurology and Neuroscience, Faculty of Medicine, Medical University “Prof. Paraskev Stoyanov”, Varna, Bulgaria;* ^*2*^ *Department of Optometry and Occupational Diseases, Faculty of Public Health, Medical University “Prof. Paraskev Stoyanov”, Varna, Bulgaria;* ^*3*^ *Department of Imaging Diagnostics, Interventional Radiology, Faculty of Medicine, Medical University of Varna, Bulgaria*

## EPV‐1191

### Two cases of diplopia caused by arteriovenous fistulas and a short literature review

#### K. Kournis ^1^; E. Strataki^1^; N. Arkoudis^2^; G. Velonakis^2^; V. Zouvelou^1^

##### ^*1*^ *First Department of Neurology, Medical School, National and Kapodistrian University of Athens, Athens, Greece (ERN EURO‐NMD);* ^*2*^ *Research Unit of Radiology;* ^*2*^ *nd Department of Radiology, Medical School, National and Kapodistrian University of Athens, Athens, Greece*

## EPV‐1192

### When brain calcifications meet cancer: Glioblastoma in Fahr's disease

#### I. Vasilache ^1^; V. Stefanescu^1^; D. Stefanescu^2^; R. Toma^3^; A. Dulamea^4^

##### ^*1*^ *Neurology department, Fundeni Clinical Institute, Bucharest, Romania;* ^*2*^ *Neurology department, Bucharest Oncological Institute, Bucharest, Romania;* ^*3*^ *Radiotherapy Department, Bucharest Oncological Institute, Bucharest, Romania;* ^*4*^ *Carol Davila University of Medicine and Pharmacy, Bucharest, Romania*

## Neuroimmunology

## EPV‐1193

### A qualitative study of factors affecting multiple sclerosis management

#### K. Madineni; M. Hernandez; T. Moro; T. Johnson; F. Sierra_Morales

##### Department of Neurological Sciences, RUSH Multiple Sclerosis Center, RUSH University Medical Center, Chicago, USA

## EPV‐1194

### A real‐life experience with eculizumab in neuromyelitis optica spectrum disorders patients

#### J. Li; M. Su

##### Department of Neurology, Xiangya Hospital, Central South University, Changsha, China

## EPV‐1195

### A triple challenge in anti‐GAD65 encephalitis: Diagnostic mimicry, therapeutic refractoriness, and the herpes simplex virus conundrum

#### B. Chelaru; S. Petrescu; C. Gheti; M. Martoiu; C. Panea

##### Neurology, Elias Emergency University Hospital, Bucharest, Romania

## EPV‐1196

### Acute encephalopathy with leptomeningeal‐predominant amyloid‐β‐related angiitis: A diagnostic blind spot in clinicoradiologic criteria

#### R. Gupta ^1^; T. Canento Sanchez^4^; M. George^3^

##### ^*1*^ *Department of Perioperative Medicine, Division of Surgical Services Logan and Beaudesert Health Service, Queensland, Australia;* ^*3*^ *Department of Psychogeriatrics,West Moreton Health, Ipswich Hospital, Ipswich, Queensland, Australia;* ^*4*^ *Department of Neurology, Princess Alexandra Hospital, Woolloongabba, Queensland, Australia*

## EPV‐1197

### Acute presentation of anti‐IgLON5 encephalitis with response to immunotherapy

#### M. Chondrogiorgi ^1^; A. Haski^2^; A. Koursaris^2^; D. Chatzistefanidis^1^; S. Konitsiotis^1^

##### ^*1*^ *Department of Neurology, Medical School, University of Ioannina, Ioannina, Greece;* ^*2*^ *Department of Neurology, University Hospital of Ioannina, Ioannina, Greece*

## EPV‐1198

### Anti‐cyclic citrullinated peptide in cerebrospinal fluid as a diagnostic marker of aseptic meningitis in rheumatoid arthritis: Two case reports

#### M. El Harmochi Daoud; M. Muñoz Pasadas; P. Gómez Ramírez ^1^; A. Sánchez Gómez; A. Herrera Ortega; M. Carrillo Carrillo; V. Ros Escolar; A. Hernández Gónzalez; M. Usero Ruíz; M. Corrales Arroyo; J. Vaamonde Gamo

##### Hospital General Universitario Ciudad Real, Spain

## EPV‐1199

### Anti‐IgLON5 encephalitis: A diagnostic challenge

#### H. El Mouhajir Mohamed; G. Torres Sanchez; S. Rodrigo Herrero

##### Neurology Service, Juan Ramón Jiménez Hospital, Huelva, Spain

## EPV‐1200

### Anti‐SOX1 autoantibody positivity in an 11‐year‐old child after COVID‐19: A case report

#### G. Kurt Bayır^1^; S. Aydın^2^; G. Öz Tuncer^3^; H. Kınık Kaya^4^; A. Aksoy ^5^

##### ^*1*^ *Division of Pediatric Neurology, Department of Pediatrics, Faculty of Medicine, Ondokuz Mayıs University, Samsun/Türkiye;* ^*2*^ *Division of Pediatric Neurology, Department of Pediatrics, Faculty of Medicine, Ondokuz Mayıs University, Samsun/Türkiye;* ^*3*^ *Division of Pediatric Neurology, Department of Pediatrics, Faculty of Medicine, Ondokuz Mayıs University, Samsun/Türkiye;* ^*4*^ *Divisions of Pediatric Critical Care and Ondokuz Mayıs University School of Medicine, Samsun/Türkiye;* ^*5*^ *Division of Pediatric Neurology, Department of Pediatrics, Faculty of Medicine, Ondokuz Mayıs University, Samsun/Türkiye*

## EPV‐1201

### Apathy is a frequent symptom but does not correlate with sNfl and sGFAP in multiple sclerosis

#### F. Massaro^1^; T. Zaccone^2^; C. Tambussi^3^; A. Mingione^1^; C. Monzani^1^; K. Piscopo^4^; A. Corona^1^; S. Mesiti^1^; M. Guidetti^3^; E. Vegni^4^; S. Marceglia^3^; T. Bocci^5^; S. Oliveri^5^; A. Priori^5^; F. Martinelli Boneschi ^6^

##### ^*1*^ *Laboratory of Precision Medicine of Neurological Diseases, Aldo Ravelli Center for Neurotechnology and Experimental Brain Therapeutics, Department of Health Sciences, University of Milan, Milan, Italy;* ^*2*^ *Clinical Neurology Unit, ASST Santi Paolo e Carlo, Milan, Italy;* ^*3*^ *Aldo Ravelli Center for Neurotechnology and Experimental Brain Therapeutics, Department of Health Sciences, University of Milan, Milan, Italy;* ^*4*^ *Unit of Clinical Psychology, Department of Mental Health and Dependencies, Santi Paolo and Carlo Hospitals, Department of Health Sciences, University of Milan, Milan, Italy;* ^*5*^ *Aldo Ravelli Center for Neurotechnology and Experimental Brain Therapeutics, Department of Health Sciences, University of Milan, Clinical Neurology Unit, ASST Santi Paolo e Carlo, Milan, Italy;* ^*6*^ *Laboratory of Precision Medicine of Neurological Diseases, Aldo Ravelli Center for Neurotechnology and Experimental Brain Therapeutics, Department of Health Sciences, University of Milan, Clinical Neurology Unit, ASST Santi Paolo e Carlo, Milan, Italy*

## EPV‐1202

### Association between EDSS and brain metrics during peri‐pregnancy in women with multiple sclerosis

#### M. Sönmez ^1^; S. Koç^2^; K. Buyukturkoglu^3^; L. Davis^3^; D. Arslan Mehdiyev^1^; M. Terzi^4^; C. Tozlu^5^

##### ^*1*^ *Department of Neurology, Ankara Etlik City Hospital, Ankara, Türkiye;* ^*2*^ *Department of Neuroscience, Ondokuz Mayıs University Institute of Graduate Studies, Samsun, Türkiye;* ^*3*^ *The Center for Translational and Computational Neuroimmunology, Columbia University Irving Medical Center, New York, NY, USA;* ^*4*^ *Department of Neurology, Ondokuz Mayıs University Faculty of Medicine, Samsun, Türkiye;* ^*5*^ *Department of Radiology, Weill Cornell Medical Center, New York, NY, USA*

## EPV‐1203

### Association between surgical waiting time and prognosis after thymectomy in non‐thymomatous MG patients: A multicenter real‐world study

#### L. Yang ^1^; H. Zhang^2^; Y. Shi^2^; Y. Cui^3^; X. Zhang^4^; X. Zhang^4^; Y. Xue^4^; Y. Geng^4^; X. Lian^5^; P. Liu^4^; M. Fan^2^; G. Qi^4^

##### ^*1*^ *Songjiang Hospital Affiliated to Shanghai Jiao Tong University School of Medicine, Department of Neurology, Shanghai, China;* ^*2*^ *Department of Neurology, Tianjin Medical University General Hospital, Tianjin, China;* ^*3*^ *China National Clinical Research Center for Neurological Diseases, Beijing Tiantan Hospital, Capital Medical University, Beijing, China;* ^*4*^ *Shijiazhuang People's Hospital, Center of Myasthenia Gravis, Shijiazhuang, Hebei, China;* ^*5*^ *Changzhou First Hospital, Department of Neurology, Changzhou, Jiangsu, China*

## EPV‐1204

### Attention‐deficit/hyperactivity disorder and subsequent risk of multiple sclerosis: A population‐based retrospective case‐control study

#### Y. Eshel^1^; D. Hadar^2^; H. Cohen^2^; N. Cohen^2^; S. Bloch^1^; D. Golan ^1^

##### ^*1*^ *Multiple Sclerosis and Neuroimmunology Center, Department of Neurology, Lady Davis Carmel Medical Center, Haifa, Israel, Rappaport Faculty of Medicine, Technion‐Israel Institute of Technology, Haifa, Israel;* ^*2*^ *Research Authority, Lady Davis Carmel Medical Center, Haifa, Israel*

## EPV‐1205

### Autoimmune brainstem encephalitis: A cohort study and topic modelling using artificial intelligence based BERTopic model on PubMed abstracts

#### M. Jin ^1^; J. Kerstens^1^; R. van Steenhoven^1^; E. Erdag Turgeon^1^; M. Nagtzaam^1^; M. van Duijn^1^; S. Veenbergen^2^; J. de Vries^1^; M. Titulaer^1^

##### ^*1*^ *Neurology department of Erasmus Medical Center, Rotterdam, The Netherlands;* ^*2*^ *Department of Immunology, Laboratory Medical Immunology, Erasmus Medical Center, Rotterdam, the Netherlands*

## EPV‐1206

### Autoimmune limbic encephalitis additional onset features

#### T. Santana; A. Morgadinho; I. Rosário Marques

##### Department of Neurology, Local Health Unit of Almada‐Seixal, Almada, Portugal

## EPV‐1207

### Beyond IgG1: Predominance of IgG2 and IgG3 subclasses in low‐positive MOG‐IgG profiles and its diagnostic implications

#### A. Vural; M. Mamipour; M. Yousefi

##### Koç University Research Center for Translational Medicine, İstanbul, Türkiye

## EPV‐1208

### Beyond neurology – The multidisciplinary journey of patients with multiple sclerosis: Portrait of a Portuguese cohort

#### Â. Seke

##### *Neurology Department, Neuroscience Division, Local Health Unit of Santo António, Porto, Portugal;* ^*2*^ *ICBAS – Institute of Biomedical Sciences Abel Salazar, University of Porto, Porto, Portugal;* ^*3*^ *Neurophysiology Department, Neuroscience Division, Local Healt, Porto, Portugal*

## EPV‐1209

### Beyond Stiff Person Syndrome: A case of Progressive Encephalomyelitis with Rigidity and Myoclonus (PERM) with status epilepticus and stroke mimicry

#### E. Garcés Becerril; D. Ginarte Milanés; Á. Antón Conejos; S. García Rubio; E. Bellosta Diago; R. Caldu Agud

##### Neurology, Hospital Clínico Universitario Lozano Blesa, Zaragoza, Spain

## EPV‐1210

### Cerebrospinal fluid spectrum in Weston‐Hurst acute hemorrhagic leukoencephalitis: A four‐case series

#### H. Iranmanesh ^1^; G. Naeije^2^

##### ^*1*^ *Neurology, CHU Brugmann, Brussels, Belgium;* ^*2*^ *Neurology, Hôpital Erasme, Brussels, Belgium*

## EPV‐1211

### Characterization of sleep disturbances in patients with demyelinating diseases: A comparative study of MOGAD, NMOSD, and multiple sclerosis

#### M. Lorenzotti ^1^; R. Orlandi^2^; A. Gajofatto^3^

##### ^*1*^ *Neurology, Verona university Hospital, Verona, Italia;* ^*2*^ *Neurology, Verona university Hospital, Verona, Italia;* ^*3*^ *Neurology, Verona University Hospital, Verona, Italia*

## EPV‐1212

### Clinical and immunological spectrum of Central Nervous System Demyelinating Diseases in Sri Lanka: A retrospective observational study

#### A. Wanniarachchi; P. Wijethunga; P. Ruwanpathirana; B. Senanayake

##### Institute of Neurology, National Hospital of Sri Lanka, Colombo, Sri Lanka

## EPV‐1213

### Clinical and radiological response to Pembrolizumab in a newly diagnosed HIV‐associated progressive multifocal leukoencephalopathy: A case report

#### A. Nawaz

##### Central Park Teaching Hospital, Lahore, Kahna Nau, Lahore, Pakistan

## EPV‐1214

### Clinical characteristics, treatment and outcomes in chronic meningitis in neurosarcoidosis: A retrospective cohort study

#### K. Mahabali; W. den Broeder; W. Westendorp; D. van de Beek; M. Brouwer

##### Department of Neurology, Amsterdam UMC, Amsterdam, The Netherlands

## EPV‐1215

### Clinical outcome of AQP4‐IgG seropositive neuromyelitis optica spectrum disorders in Hong Kong Chinese

#### K. Chan

##### Department of Medicine, LKS Faculty of Medicine, The University of Hong Kong

## EPV‐1216

### Clinical profile of patients with myasthenia gravis in China: A nationwide prospective cohort study

#### T. Chang ^1^; H. Wang^2^; G. Qi^3^; X. Ma^4^; L. Bai^4^; Y. Wang^4^; D. Yuan^4^; Q. Guo^4^; X. Meng^5^; F. Shi^5^

##### ^*1*^ *Department of Neurology, Tangdu Hospital, the Fourth Military Medical University, Xi'an, Shaanxi, China;* ^*2*^ *Department of Neurology, Beijing Tiantan Hospital, Capital Medical University, Beijing, China;* ^*3*^ *Treatment Center of Myasthenia Gravis, People's Hospital of Shijiazhuang, Hebei Medical University, Shijiazhuang, China;* ^*4*^ *AstraZeneca Global R&D (China) Co., Ltd., Shanghai, China;* ^*5*^ *Department of Neurology, China National Clinical Research Center for Neurological Diseases, Beijing Tiantan Hospital, Capital Medical University, Beijing, China*

## EPV‐1217

### Clinical recognition pathways in autoimmune GFAP astrocytopathy: Insights from three cases

#### S. Yamamoto; G. Watanabe; G. Akao; A. Ishikawa; A. Tamagake; K. Tsukita; Y. Suzuki

##### Department of Neurology, National Hospital Organization Sendai Medical Center, Sendai, Japan

## EPV‐1218

### Clinical spectrum and serological features of neuromyelitis optica spectrum disorder in a Sri Lankan cohort

#### A. Wanniarachchi; P. Wijethunga; P. Ruwanpathirana; B. Senanayake

##### Institute of Neurology, National Hospital of Sri Lanka, Colombo, Sri Lanka

## EPV‐1219

### Cognitive impairment in rheumatoid arthritis: Association with disease activity and inflammation

#### M. Abzalova; M. Yakubova; N. Abdurakhmanova

##### Tashkent State Medical University, Tashkent, Uzbekistan

## EPV‐1220

### Comparability of serum neurofilament light chain measurements by SIMOA and ECLIA in stroke and multiple sclerosis patients

#### A. Konitsioti ^1^; F. Schweitzer^2^; W. Johannis^2^; G. Fink^1^; M. Schroeter^1^; F. Steffen^3^; S. Bittner^3^; C. Warnke^4^

##### ^*1*^ *Department of Neurology, University of Cologne, Faculty of Medicine and University Hospital Cologne, Cologne, Germany;* ^*2*^ *Institute for Clinical Chemistry, Faculty of Medicine and University Hospital Cologne, Cologne, Germany;* ^*3*^ *Department of Neurology, Focus Program Translational Neuroscience (FTN) and Immunotherapy (FZI), Rhine Main Neuroscience Network (rmn(2)), University Medical Center of the Johannes Gutenberg University Mainz, Mainz, Germany;* ^*4*^ *Department of Neurology, University Hospital Marburg, Germany*

## EPV‐1221

### Comparison of patients with familial and sporadic multiple sclerosis – selected risk factors of the disease – Neurological Clinic in Zabrze, Poland

#### K. Kubicka‐Bączyk^1^; J. Bączyk^1^; J. Jurkiewicz^1^; P. Choręza^2^; E. Chełmecka^2^; M. Adamczyk‐Sowa ^1^

##### ^*1*^ *Department of Neurology, Faculty of Medical Sciences in Zabrze, Medical University of Silesia in Katowice, Poland;* ^*2*^ *Department of Medical Statistics Faculty of Pharmaceutical Sciences in Sosnowiec Medical University of Silesia in Katowice, Poland*

## EPV‐1222

### Comparison of symptom frequency and heritability in patients with familial and sporadic multiple sclerosis. Neurological Clinic in Zabrze, Poland

#### K. Kubicka‐Bączyk^1^; J. Bączyk^1^; J. Jurkiewicz^1^; P. Choręza^2^; N. Morawiec^1^; E. Chełmecka^2^; M. Adamczyk‐Sowa ^1^

##### ^*1*^ *Department of Neurology, Faculty of Medical Sciences in Zabrze, Medical University of Silesia in Katowice, Poland;* ^*2*^ *Department of Medical Statistics Faculty of Pharmaceutical Sciences in Sosnowiec Medical University of Silesia, Poland*

## EPV‐1223

### CSF oligoclonal bands are not a useful biomarker for predicting early reactivation after discontinuation of first‐line multiple sclerosis therapies

#### K. van Tulder ^1^; N. Jafari^2^; C. Corsten^1^; R. Klein Kranenbarg^3^; M. van Luijn^4^; I. Smets^1^; B. Wokke^1^; J. Smolders^5^

##### ^*1*^ *Department of Neurology, MS center ErasMS, Erasmus MC, University Medical Center, Rotterdam, the Netherlands;* ^*2*^ *Department of Neurology, Amphia Hospital, Breda, the Netherlands;* ^*3*^ *Department of Neurology, MS center ErasMS, Erasmus MC, University Medical Center, Rotterdam, the Netherlands; Department of Neurology, Albert Schweitzer Hospital, Dordrecht, the Netherlands;* ^*4*^ *Department of Immunology, MS Center ErasMS, Erasmus MC, University Medical Center, Rotterdam, the Netherlands;* ^*5*^ *Department of Neurology, Department of Immunology, MS center ErasMS, Erasmus MC, University Medical Center, Rotterdam, the Netherlands; Neuroimmunology Research Group, Netherlands Institute for Neuroscience, Amsterdam, the Netherlands*

## EPV‐1224

### Eculizumab for refractory thymoma‐associated myasthenia gravis with dual impending myasthenic crisis and cholinergic crisis: A new hope?

#### S. Liu ^1^; R. Xu^2^; G. Huang^2^; C. Tang^2^; Q. Sheng^2^

##### ^*1*^ *Department of Neurology, Jiangxi Provincial People's Hospital, Clinical College of Nanchang Medical College, First Affiliated Hospital of Nanchang Medical College, Xiangya Hospital of Central South University Jiangxi Hospital, National Regional Medical Ce;* ^*2*^ *Department of Neurology, Jiangxi Provincial People's Hospital, Clinical College of Nanchang Medical College, First Affiliated Hospital of Nanchang Medical College, Xiangya Hospital of Central South University Jiangxi Hospital, National Regional Medical Ce*

## EPV‐1225

### Efficacy and safety of eculizumab in refractory spontaneous 3M syndrome associated with malignancy

#### J. Guo; L. Chen; M. Li; J. Liu; Q. Wang; L. Chen

##### Department of Neurology, The Second Hospital & Clinical Medical School, Lanzhou University, Lanzhou, China

## EPV‐1226

### Efficacy and safety of ozanimod in multiple sclerosis: A systematic review and meta‐analysis

#### A. Hasanin ^1^; H. Khelifa^2^

##### ^*1*^ *Faculty Of Medicine, University of Tripoli, Libya;* ^*2*^ *University of Oran 1 Ahmed Ben Bella, Algeria*

## EPV‐1227

### Features of humoral immunity in children and adolescents with migraine

#### D. Alidjanova; R. Kadirova

##### Tashkent State Medical University, Tashkent, Uzbekistan

## EPV‐1228

### From childhood to adulthood: Phenotypic differentiation and clinical evolution in Myelin oligodendrocyte glycoprotein antibody‐associated disease

#### S. Costa ^1^; F. Barbosa^1^; D. Duarte^2^; J. Lopes^1^; D. Costa^1^; R. Samões^1^; S. Figueiroa^3^; E. Santos^1^

##### ^*1*^ *Serviço de Neurologia, Departamento de Neurociências, Unidade Local de Saúde de Santo António, Porto, Portugal;* ^*2*^ *Serviço de Neurorradiologia, Departamento de Neurociências, Unidade Local de Saúde, Porto, Portugal;* ^*3*^ *Serviço de Neuropediatria, Centro Materno Infantil do Norte, Unidade Local de Saúde de Santo António, Porto, Portugal*

## EPV‐1229

### GFAP astrocytopathy associated with Epstein–Barr virus infection: A case report

#### V. Tseriotis; F. Lioliou; P. Papadopoulos; C. Vogiatzi; D. Thomas; S. Koukou

##### Department of Neurology, Agios Pavlos General Hospital of Thessaloniki, Thessaloniki, Greece

## EPV‐1230

### Glutamate decarboxylase antibody‐associated neurological disorders: Clinical features, immunotherapy, and short‐term outcomes in a russian cohort

#### E. Chekanova; E. Nuzhnyi; E. Fedotova; E. Shalimanova; E. Golovneva; M. Zakharova

##### Russian Center of Neurology and Neurosciences, Moscow, Russian Federation

## EPV‐1231

### Immune‐related adverse events due to immune checkpoint inhibitors: A single‐centre case series

#### A. Favero ^1^; A. Sartori^2^; G. Mazzon^2^; M. Tomaselli^2^; A. Bratina^2^; A. Bosco^2^; P. Manganotti^1^; A. Benussi^1^

##### ^*1*^ *Neurology Unit, School of Neurology, Department of Medical, Surgical and Health Sciences, University of Trieste, Trieste, Italy;* ^*2*^ *Neurology Unit, Cattinara Hospital, Azienda Sanitaria Universitaria Giuliano Isontina, Trieste, Italy*

## EPV‐1232

### Impact of prior infection and vaccination on immune‐mediated neurological syndromes: A tertiary hospital experience

#### P. López‐Grueiro Valcarce; J. Granja López; P. Condo Cabrera; S. Arés Ballesteros; S. Sánchez Velasco; L. Lacruz Ballester

##### Neurology, Hospital Universitario La Paz, Madrid, Spain

## EPV‐1233

### Inborn errors of immunity as a novel etiology for neuroinfectious and neuroinflammatory disorders

#### F. Safavi

##### NIAID, National Institute of Health, USA

## EPV‐1234

### Increased vulnerability to autoimmune CNS disorders after post‐COVID herpes zoster: A global real‐world analysis

#### Y. Cheng ^1^; C. Lu^1^; J. Wang^2^; Y. Hou^1^; K. Lu^3^

##### ^*1*^ *School of Medicine, College of Medicine, Fu Jen Catholic University, New Taipei City, Taiwan;* ^*2*^ *Department of Research, Taipei Tzu Chi Hospital, Buddhist Tzu Chi Medical Foundation, New Taipei City, Taiwan.;* ^*3*^ *Division of Nephrology, Department of Medicine, Taipei Tzu Chi Hospital, Buddhist Tzu Chi Medical Foundation, New Taipei City, Taiwan*

## EPV‐1235

### Kappa and Lambda Index as diagnostic and prognostic biomarkers in autoimmune neurological disorders

#### E. Virgilio ^1^; M. Morra^2^; A. Maglione^1^; R. Rosso^1^; F. Masuzzo^2^; M. Matta^2^; S. Rolla^1^; A. Sala^3^; A. Di Sapio^3^; M. Clerico^1^

##### ^*1*^ *Department of Clinical and Biological Sciences, University of Turin, Turin, Italy;* ^*2*^ *Patologie Neurologiche e Specialistiche Unit, San Luigi Gonzaga Hospital, Orbassano, Italy;* ^*3*^ *SCDO Neurology Unit, San Luigi Gonzaga Hospital, Orbassano, Italy*

## EPV‐1236

### Kappa Free light chain index in CSF: A biomarker beyond multiple sclerosis

#### F. Assis Jacinto; F. Correia; S. França; R. Rocha; D. Fitas

##### Neurology Department, Hospital Pedro Hispano, Matosinhos, Portugal

## EPV‐1237

### Longitudinal [¹^8^F]FDG‐PET changes map onto distinct cholinergic/dopaminergic and serotonergic territories in autoimmune encephalitis

#### D. Cerne ^1^; G. Benvenuto^2^; S. Raffa^3^; A. Panza^2^; C. Barone^2^; A. Lechiara^4^; E. Mobilia^4^; G. Rebella^3^; P. Mattioli^2^; B. Orso^2^; E. Micalizzi^3^; D. Arnaldi^2^; F. Villani^2^; L. Roccatagliata^5^; G. Pesce^4^; M. Pardini^2^; S. Morbelli^6^; A. Uccelli^3^; L. Benedetti^1^; F. Massa^2^

##### ^*1*^ *Department of Neurology, Sant'Andrea Hospital, La Spezia, Italy;* ^*2*^ *Department of Neuroscience, Rehabilitation, Ophthalmology, Genetics, Maternal and Child Health, University of Genoa, Genoa, Italy;* ^*3*^ *RCCS Ospedale Policlinico San Martino, Genoa, Italy;* ^*4*^ *Autoimmunology Laboratory, IRCCS Ospedale Policlinico San Martino, Genoa, Italy;* ^*5*^ *Department of Health Sciences (DISSAL), University of Genoa, Genoa, Italy;* ^*6*^ *Nuclear Medicine Unit, Azienda Ospedaliero‐Universitaria Città della Salute e della Scienza di Torino, Turin, Italy*

## EPV‐1238

### Longitudinal study on apathy and social cognition in early relapsing‐remitting multiple sclerosis

#### G. Diniz de Pinho ^1^; C. Chester^2^; D. Silva^2^; I. Brás Marques^1^

##### ^*1*^ *Neurology Department, Hospital da Luz, Lisboa, Portugal;* ^*2*^ *Neuropsychology Department, Hospital da Luz, Lisboa, Portugal*

## EPV‐1239

### Long‐term clinical and functional burden of autoimmune encephalitis: A targeted literature review

#### C. Buffel^1^; X. Zhao^1^; M. De Belder^2^; C. López de Silanes de Miguel^2^; S. Ellor^3^; S. Schmidt^2^; F. De Ruyck ^2^

##### ^*1*^ *ISMS, Zoersel, Belgium;* ^*2*^ *argenx, Ghent, BBelgium,* ^*3*^ *arargenx, Boston, USA*

## EPV‐1240

### Mapping of neuromyelitis optica spectrum disorders in Belgium: Interim results

#### A. Lefter ^1^; D. Dive^2^; V. Van Pesch^3^; B. Van Wijmeersch^4^; F. London^5^; M. DHaeseleer^6^; M. Vokaer^7^; E. Lommers^2^; S. Dauby^2^; C. Ernon^2^; S. Laurent^8^; K. Delmotte^9^; V. Deprez^10^; V. Tota^11^; J. Bouziotis^12^; J. Derdelinckx^13^; T. Reynders^1^; B. Willekens^1^

##### ^*1*^ *Translational Sciences Research Group, Faculty of Medicine and Health Sciences, University of Antwerp, Wilrijk, Belgium; Department of Neurology, Antwerp University Hospital, University of Antwerp, Edegem, Belgium;* ^*2*^ *Department of Neurology, Centre Hospitalier Universitaire de Liège, Liège, Belgium;* ^*3*^ *Department of Neurology, Cliniques Universitaires Saint‐Luc, Université Catholique de Louvain, Brussels, Belgium;* ^*4*^ *University MS Center, Noorderhart, Rehabilitation and MS, Pelt, Belgium;* ^*5*^ *Department of Neurology, CHU UCL Namur site Godinne, Université Catholique de Louvain, Yvoir, Belgium;* ^*6*^ *Department of Neurology, University Hospital Brussels, Brussels, Belgium; National Multiple Sclerosis Center Melsbroek, Melsbroek, Belgium;* ^*7*^ *Department of Neurology, MS Clinic, Delta Hospital, CHIREC group, Brussels, Belgium;* ^*8*^ *National Multiple Sclerosis Center Melsbroek, Melsbroek, Belgium; Neurology, CHU Saint‐Pierre, Université libre de Bruxelles, Brussels, Belgium;* ^*9*^ *Jessa Hospital, Hasselt, Belgium;* ^*10*^ *Department of Neurology, AZ Rivierenland Hospital, Rumst, Belgium;* ^*11*^ *Department of Neurology, Centres Hospitaliers Universitaires HELORA, Mons, Belgium;* ^*12*^ *Clinical Trial Center, CRC Antwerp, Antwerp University Hospital, University of Antwerp, Edegem, Belgium;* ^*13*^ *Department of Neurology, Antwerp University Hospital, University of Antwerp, Edegem, Belgium; University of Antwerp, Laboratory of Experimental Hematology, Vaccine and Infectious Disease Institute (Vaxinfectio), Antwerp, Belgium*

## EPV‐1241

### Mapping the clinical trajectory of MOGAD

#### S. Gomes ^1^; A. Costa^1^; M. Gomes^2^; A. Santos^1^; F. Sousa^1^; J. Pinto^1^; J. Cerqueira^1^

##### ^*1*^ *Neurology Department, Unidade Local de Saúde de Braga, Braga, Portugal;* ^*2*^ *Neuroradiology Department, Unidade Local de Saúde de Braga, Braga, Portugal*

## EPV‐1242

### Mitochondrial–immune overlap in leber hereditary optic neuropathy: A case report and lessons learned

#### W. Eltantawi

##### International Medical Center, Jeddah, Saudi Arabia

## EPV‐1243

### Modulation of AMPA‐receptors through combination of medium‐chain triglycerides and perampanel to control autoimmune status epilepticus: Case report

#### L. Stadler^1^; L. Schappe^1^; P. Lochner^1^; L. Bonifer^1^; M. Fousse^1^; S. Meuth^2^; S. Groppa^1^; Y. Winter ^1^

##### ^*1*^ *Department of Neurology, Saarland University Medical Center, University of Saarland, Homburg, Germany;* ^*2*^ *Department of Neurology, University Hospital Münster, Münster, Germany*

## EPV‐1244

### Multiple sclerosis and autoimmune comorbidities

#### S. Costa ^1^; Â. Mpovo^1^; J. Gonçalves^2^; J. Lopes^1^; D. Costa^1^; A. Sousa^3^; R. Samões^1^; E. Santos^1^

##### ^*1*^ *Serviço de Neurologia, Departamento de Neurociências, Unidade Local de Saúde de Santo António, Porto, Portugal;* ^*2*^ *ICBAS – Instituto de Ciências Biomédicas Abel Salazar, Universidade do Porto, Porto, Portugal;* ^*3*^ *Serviço de Neurofisiologia, Departamento de Neurociências, Unidade Local de Saúde, Porto, Portugal*

## EPV‐1245

### Natalizumab: a drug to avoid in multiple sclerosis patients with atypical lesions? – A case report

#### I. Cwajgenberg; J. Tuler; G. Meyas; R. Mancini; I. Rangel; S. Mermelstein; F. Schmidt; D. Terrana

##### Department of Neurology, Pedro Ernesto University Hospital, Rio de Janeiro, Brazil

## EPV‐1246

### Neurological chronic toxicity induced by immune checkpoint inhibitors: A case of encephalitis, optic neuropathy and polyneuropathy

#### F. Capuozzo ^1^; G. D'Alvano^1^; C. Cesaro^2^; M. Cirillo^1^; A. Tessitore^1^; E. Martinelli^2^; A. Bisecco^1^

##### ^*1*^ *Department of Advanced Medical and Surgical Sciences – University of Campania “Luigi Vanvitelli”, Italy;* ^*2*^ *Department of Precision Medicine – University of Campania “Luigi Vanvitelli”, Italy*

## EPV‐1247

### Neurological complications of immune checkpoint inhibitors therapy in patients with malignant melanoma

#### I. Štětkářová ^1^; M. Židó^1^; E. Ehler^2^

##### ^*1*^ *Department of Neurology, Third Faculty of Medicine, Charles University and University Hospital Kralovske Vinohrady, Prague, Czech Republic;* ^*2*^ *Department of Neurology, Faculty of Health Studies, Pardubice University and Pardubice Regional Hospital, Czech Republic*

## EPV‐1248

### Neurological manifestations in rheumatoid arthritis: prevalence and inflammatory correlates

#### M. Abzalova; M. Yakubova; N. Abdurakhmanova

##### Tashkent State Medical University, Tashkent, Uzbekistan

## EPV‐1249

### Neurosarcoidosis mimicking a high‐grade brain tumor: A diagnostic challenge

#### D. Machado Sampedro ^1^; J. Sosa Luis^1^; J. Martinez Barrio^2^; J. Garbizu Vidorreta^3^; Y. Fernandez Bullido^2^; G. Lafuente Gómez^1^

##### ^*1*^ *Neurología, Hospital General Universitario Gregorio Marañón, Madrid, Spain;* ^*2*^ *Reumatología, Hospital General Universitario Gregorio Marañón, Madrid, Spain;* ^*3*^ *Neurocirugía, Hospital General Universitario Gregorio Marañón, Madrid, Spain*

## EPV‐1250

### Ofatumumab in refractory anti‐GABABR encephalitis with active infection

#### J. Wang

##### Department of Neurology, Chongqing University Affiliated Sanxia Hospital, Chongqing, China

## EPV‐1251

### Optimization of treatment of autonomic disorders and vegetative dysfunction in patients with rheumatoid arthritis

#### S. Kalandarova; S. Kuranbayeva; S. Rustambekova; A. Amriddinov

##### Tashkent State Medical University, Tashkent, Uzbekistan

## EPV‐1252

### Outcomes within 5 years following different immunosuppressive treatment protocols in cerebral amyloid angiopathy‐related inflammation: A case series

#### C. Morgan ^1^; N. Gupta^1^; A. Siegel^2^; A. Nevares^3^; F. Sweeney^1^; C. Roy‐Hewitson^1^

##### ^*1*^ *Neurology, University of Vermont Medical Center, Burlington, USA;* ^*2*^ *Larner College of Medicine, University of Vermont, Burlington, USA;* ^*3*^ *Rheumatology, University of Vermont Medical Center, Burlington, USA*

## EPV‐1253

### Overlap between anti‐NMDAR encephalitis and multiple sclerosis: A rare neuroimmunological overlap

#### C. Conde Velasco; J. Arzalluz Luque; I. Ruiz Salcedo; A. Rincón Valencia; S. Eichau Madueño

##### Department of Neurology, Hospital Virgen Macarena, Sevilla, Spain

## EPV‐1254

### Overlap syndrome of myositis, myocarditis, and myasthenia gravis secondary to immune checkpoint inhibitors (ICI): A tertiary‐center case series

#### C. Conde Velasco; Á. Laviana Marín; A. Rincón Valencia; I. Ruiz Salcedo; Y. Morgado Linares; E. Martínez Fernández

##### Department of Neurology. Hospital Virgen Macarena. Sevilla. Spain

## EPV‐1255

### Pediatric MOGAD atypical fulminant or insidious presentations: monophasic or relapsing conditions?

#### S. Garcia‐Tarodo ^1^; A. Bordessoule^2^; C. Habre^3^; J. Fluss^1^; K. Deiva^4^

##### ^*1*^ *Neuropediatric Unit, Department of Child, Adolescent and Women, Geneva University Hospitals, Switzerland;* ^*2*^ *Pediatric intensive care, Department of Child, Adolescent and Women, Geneva University Hospitals, Switzerland;* ^*3*^ *Pediatric Radiology Unit, Department of Diagnostic, Geneva University Hospitals, Switzerland;* ^*4*^ *Pediatric Neurology department, Paris Saclay University Hospitals, Le Kremlin Bicêtre, Paris, France*

## EPV‐1256

### Posterior cortical atrophy caused by antiphospholipid syndrome Identified by the “railroad track” sign on brain PET

#### A. Aksamit

##### Mayo Clinic, Rochester MN, USA

## EPV‐1257

### Pre‐diagnostic disease trajectories preceding central nervous system inflammation: A UK Biobank Study

#### D. Kook ^1^; H. Kim^2^; K. Kim^3^; Y. Park^2^; W. Kim^1^; B. Choi^1^

##### ^*1*^ *Department of Neurology, Gangnam Severance Hospital, Yonsei University College of Medicine, Seoul, Republic of Korea;* ^*2*^ *Department of Biomedical Systems Informatics, Yonsei University College of Medicine, Seoul, Republic of Korea;* ^*3*^ *Departent of Neurology, Yonsei University College of Medicine, Seoul, Republic of Korea*

## EPV‐1258

### Prognostic significance of thyroid autoantibodies in MOGAD and NMOSD: A biomarker for disease activity

#### T. Fidan Çolak; S. Şen; M. Terzi

##### Department of Neurology, Faculty of Medicine, Ondokuz Mayıs University, Samsun, Türkiye

## EPV‐1259

### Progressive Hemifacial Atrophy (Parry‐Romberg Syndrome): Long‐term clinical course with traumatic onset and late stabilization

#### N. Bozhenko; M. Bozhenko

##### Neurology Department, Danylo Halytsky Lviv National Medical University, Lviv, Ukraine

## EPV‐1260

### Real‐life study of multiple sclerosis patients under treatment with cladribine

#### A. Costa; S. Gomes; S. Moreira; J. Ferreira Pinto; A. Santos; F. Sousa; J. Cerqueira

##### Neurology Department – Local Health Unit Braga, Braga, Portugal

## EPV‐1261

### Recurrent aseptic meningitis associated with nivolumab: A rare immune‐related neurological adverse event

#### L. Caballero Sánchez; M. Vargas Cobos; A. De Luca; R. Alfonso González; M. Capra; C. Gómez López de San Román; I. Bermejo Casado; D. Cerdán Santacruz; A. Castrillo Sanz; A. Mendoza Rodríguez

##### General Hospital of Segovia, Segovia, Spain

## EPV‐1262

### Reduction of immune cell subsets and autoantibodies by cladribine in generalised myasthenia gravis peripheral blood mononuclear cell‐based assays

#### Y. Wu^1^; E. Järvinen ^2^; D. Jack^3^; T. Derfuss^4^; C. Sun^1^

##### ^*1*^ *EMD Serono Research & Development Institute, Inc., Billerica, MA, USA (an affiliate of Merck KGaA);* ^*2*^ *Merck OY, Espoo, Finland (an affiliate of Merck KGaA);* ^*3*^ *Merck Serono Ltd., Feltham, UK (an affiliate of Merck KGaA);* ^*4*^ *Department of Biomedicine, University of Basel, Switzerland*

## EPV‐1263

### Role of anti‐HBs antibody titers in HBV reactivation risk stratification in pOBI patients with multiple sclerosis treated with anti‐CD20 therapy

#### L. Corsaro ^1^; A. Esposito^1^; A. Buonomo^2^; F. Cuccurullo^2^; G. Viceconte^2^; V. Fotticchia^2^; E. Scivicco^1^; R. Scotto^2^; I. Gentile^2^; A. Carotenuto^1^; M. Moccia^3^; V. Brescia Morra^1^; R. Lanzillo^1^

##### ^*1*^ *Department of Neuroscience, Reproductive Sciences and Odontostomatology, Multiple Sclerosis Clinical Care and Research Centre, Federico II University, Naples;* ^*2*^ *Department of Clinical Medicine and Surgery, Section of Infectious Diseases, University of Naples Federico II, Naples, Italy;* ^*3*^ *Department of Molecular Medicine and Medical Biotechnology, Federico II University of Naples, Naples, Italy*

## EPV‐1264

### Safety of anti‐CD20 monoclonal antibodies in multiple sclerosis: A network meta‐analysis of infection‐specific outcomes

#### S. Biswas^1^; S. Vasireddy ^2^; Y. Srivastava^1^; S. Arora^3^; J. Rajamani^4^; K. Jindal^5^; A. Jayakumar^6^

##### ^*1*^ *Department of Internal Medicine, Ivano‐Frankivsk National Medical University, Ivano‐Frankivsk, Ukraine;* ^*2*^ *Department of Neurology, NMC Specialty Hospital, Abu Dhabi, United Arab Emirates;* ^*3*^ *Department of Internal Medicine, University of Debrecen, Hungary;* ^*4*^ *Department of Internal Medicine Medical College, Caucasus University, Tbilisi, Georgia;* ^*5*^ *Department of Internal Medicine Government Medical College, Patiala;* ^*6*^ *Department of General Medicine, Sri Ramachandra Medical College, Chennai, India*

## EPV‐1265

### Seizure semiology and EEG patterns across subtypes of Autoimmune Epilepsy: Insights for early diagnosis

#### O. Sakli; R. Zouari; Z. Saied; A. Rachdi; D. Ben Mohamed; F. Nabli; L. Bouafia; S. Ben Sassi

##### Department of Neurology, Mongi Ben Hamida University Hospital, Tunis, Tunisia

## EPV‐1266

### Steroids or blindness: When optic neuritis does not evolve as expected

#### M. Luz Esteve ^1^; D. Gómez de la Torre Morales^1^; M. Ravelo León^1^; J. Aguilera Aguilera^1^; J. Rodríguez Carrillo^1^; I. Díaz Díaz^1^; A. Hernández González^1^; L. Abad Reguera^1^; B. Rodríguez García^1^; P. Serafim Reis^2^; M. Polo Martín^1^

##### ^*1*^ *Neurology Department, Complejo Asistencial Universitario de Salamanca, Salamanca, Spain;* ^*2*^ *Ophthalmology Department, Complejo Asistencial Universitario de Salamanca, Salamanca, Spain*

## EPV‐1267

### Susac's silent storm: how high clinical suspicion leads to a masquerading diagnosis. Case report

#### Á. Antón Conejos; D. Arcila Salazar; E. Noroña Vascónez; M. González Rodas; M. Martínez Montoyo; B. Del Moral Sahuquillo; E. Bellosta Diago

##### Neurology, Hospital Clínico Universitario Lozano Blesa, Zaragoza, Spain

## EPV‐1268

### Targeting ICOS/ICOS‐L signaling ameliorates disease in an autoimmune mouse model of neuromyelitis optica spectrum disorders

#### K. Chan; L. Yick; H. Xue

##### Department of Medicine, School of Clinical Medicine, LKS Faculty of Medicine, The University of Hong Kong, Hong Kong, China

## EPV‐1269

### The Gut–Brain–Immune axis in neurodegeneration: Diet as a molecular modulator

#### S. Kulkarni

##### Department of Medicine, Georgian National University SEU, Tbilisi, Georgia

## EPV‐1270

### The prevalence and nature of neurocognitive deficits in MS patients – Implications for routine inpatient assessment

#### M. Lis; M. Schneider; J. Molenda; M. Machej; R. Szymaniak; M. Huber; P. Maj; J. Jurkiewicz; M. Adamczyk‐Sowa

##### Department of Neurology, Faculty of Medical Sciences in Zabrze, Medical University of Silesia, Katowice, Poland

## EPV‐1271

### Timing matters: Study of the impact of chrononutrition on multiple sclerosis–related fatigue

#### N. Phillips^1^; V. Ajdacic‐Gross^2^; J. Rebeaud^3^; G. Thévoz^3^; S. Laquinto^4^; C. Baum^5^; C. Kamm^6^; J. Kuhle^7^; I. Rapold^2^; P. Roth^8^; C. Zecca^9^; V. von Wyl^4^; S. Eussen^10^; N. Steinemann^2^; T. Collet^1^; C. Pot ^3^

##### ^*1*^ *Service of Endocrinology, Diabetology and Metabolism, Department of Medicine, Geneva University Hospitals, Geneva, Switzerland;* ^*2*^ *Swiss MS Registry, Epidemiology, Biostatistics and Prevention Institute, University of Zürich, Zürich, Switzerland;* ^*3*^ *Laboratories of Neuroimmunology, Center for Research in Neuroscience and Service of Neurology, Department of Clinical Neurosciences, Lausanne University Hospital and University of Lausanne, Lausanne, Switzerland;* ^*4*^ *Department of Epidemiology, Epidemiology, Biostatistics and Prevention Institute, University of Zürich, Zürich, Switzerland;* ^*5*^ *Rehabilitation Clinic Zihlschlacht, Zihlschlacht, Switzerland;* ^*6*^ *Neurology and Neurorehabilitation Center, Lucerne Cantonal Hospital, Lucerne, Switzerland;* ^*7*^ *Departments of Biomedicine and Clinical Research, Multiple Sclerosis Centre and Research Center for Clinical Neuroimmunology and Neuroscience (RC2NB), University Hospital and University of Basel, Basel, Switzerland; Department of Neurology, University Hos;* ^*8*^ *Department of Neurology, University Hospital Zürich and University of Zürich, Zürich, Switzerland;* ^*9*^ *Department of Neurology, Neurocenter of Southern Switzerland, Ospedale Regionale di Lugano, EOC, Lugano, Switzerland; Faculty of Biomedical Sciences, Università della Svizzera Italiana, Lugano, Switzerland;* ^*10*^ *Department of Epidemiology; Cardiovascular Research Institute Maastricht (CARIM); and Care and Public Health Research Institute (CAPHRI), Maastricht University, Maastricht, The Netherlands*

## EPV‐1272

### Transcriptomic analysis of B cells treated with evobrutinib in a mouse model of neuromyelitis optica spectrum disorders

#### H. Xue; L. Yick; K. Chan

##### Department of Medicine, School of Clinical Medicine, LKS Faculty of Medicine, The University of Hong Kong, Hong Kong, China

## EPV‐1273

### Trial in progress – Impact of continuous assessment of generalized Myasthenia Gravis using a mobile application: myMGverse APP observational study

#### A. Vesperinas ^1^; R. Álvarez^2^; T. Garcia^3^; P. Carbonell^4^; D. Rodriguez^5^; E. Cortés^1^

##### ^*1*^ *Hospital de la Santa Creu i Sant Pau, Barcelona, Spain;* ^*2*^ *Hospital Universitario Ramón y Cajal , Madrid, Spain;* ^*3*^ *Complejo Hospitalario Universitario de Santiago, A Coruña, Spain;* ^*4*^ *Virgen de la Victoria, Málaga, Spain;* ^*5*^ *argenx, Ghent, Belgium*

## EPV‐1274

### Two cases of cerebral amyloid angiopathy‐related inflammation following immune checkpoint inhibitor treatment

#### N. Gupta ^1^; C. Morgan^1^; A. Siegel^2^; A. Nevares^3^; F. Sweeney^1^; C. Roy‐Hewitson^1^

##### ^*1*^ *Neurology, University of Vermont Medical Center, Burlington, USA;* ^*2*^ *Larner College of Medicine, University of Vermont, Burlington, USA;* ^*3*^ *Rheumatology, University of Vermont Medical Center, Burlington, USA*

## EPV‐1275

### Ublituximab for relapsing forms of MS: Interim results on real‐world treatment satisfaction, quality of life and tolerability from the BRILL study

#### S. Faissner ^1^; A. Salmen^1^; T. Ziemssen^2^; S. Pfeuffer^3^; C. Man^4^; B. Kallmann^5^; Y. Yalachkov^6^; G. Nelles^7^; C. Mayer^8^; P. Thilmann^9^; G. Reifschneider^10^; H. Miskin^11^; A. Bendarraz^12^; D. Lüftenegger^13^; A. Schmitt^13^

##### ^*1*^ *Department of Neurology, Ruhr‐University Bochum, St. Josef‐Hospital, Bochum, Germany;* ^*2*^ *Center of Clinical Neuroscience, Neurological Clinic, University Hospital Carl Gustav Carus Dresden, Dresden, Germany;* ^*3*^ *Neurological Clinic of the University Hospital Gießen and Marburg GmbH, Gießen, Germany;* ^*4*^ *neuropraxis HX, Höxter, Germany;* ^*5*^ *Multiple Sclerosis Center Bamberg, Bamberg, Germany;* ^*6*^ *Goethe University Frankfurt, University Medicine, Department of Neurology, Frankfurt am Main, Germany;* ^*7*^ *NeuroMed Campus, Köln, Germany;* ^*8*^ *Neurological Practice at Kaiserplatz, Frankfurt, Germany;* ^*9*^ *Neuroplus Group Practice for Neurology, Mannheim, Germany;* ^*10*^ *Neuro‐Centrum‐Science GmbH, Erbach, Germany;* ^*11*^ *TG Therapeutics, Morrisville, USA;* ^*12*^ *Neuraxpharm Pharmaceuticals, Barcelona, Spain;* ^*13*^ *Neuraxpharm Arzneimittel GmbH, Langenfeld, Germany*

## EPV‐1276

### Unilateral mydriasis and severe ataxia: Anti‐Hu paraneoplastic encephalomyelitis as a herald of small‐cell lung cancer

#### M. González Campos; G. Torres Sánchez; H. El Mouhahjir Mohamed; A. Ortega Ruiz; A. Tapia Barrantes; J. Mederos

##### Neurology, Juan Ramón Jiménez Hospital, Huelva, Spain

## EPV‐1277

### Very late‐onset AQP4‐positive neuromyelitis optica associated with autoimmune hemolytic anemia and thrombocytopenia: a case report

#### M. Favale; C. Esilio; A. d'Ambrosio; A. Bisecco; R. Docimo; A. Tessitore; A. Gallo

##### I Division of Neurology and Department of Advanced Medical and Surgical Sciences, University Hospital and University of Campania “Luigi Vanvitelli”, Naples, Italy

## EPV‐1278

### Vestibular tests and virtual reality–based evaluation of otolith organs in MS: Associations with brainstem lesions and serum GFAP levels

#### B. Ozdemir^1^; Z. Kaya ^2^; I. Oz^3^; M. Kilinc^2^

##### ^*1*^ *Department of Otolaryngology, Division of Audiology, Baskent University Medical School, Ankara, Türkiye;* ^*2*^ *Department of Neurology, Baskent University Medical School, Ankara, Türkiye;* ^*3*^ *Department of Otolaryngology, Baskent University Medical School, Ankara, Türkiye*

## EPV‐1279

### Viral seroprevalence and risk of reactivation in MS patients treated with ocrelizumab: A real‐world cohort study

#### E. Zengin ^1^; C. Calıskan^2^; Y. Aras^2^; S. Ozakbas^3^

##### ^*1*^ *Izmir University of Economics Medical Point Hospital, Departmant of Neurology, Izmir, Türkiye;* ^*2*^ *Izmir University of Economics Medical Point Hospital, Izmir, Türkiye;* ^*3*^ *Izmir University of Economics Medical Point Hospital, Departmant of Neurology, MS Research Association, Izmir, Türkiye*

## EPV‐1280

### Vitaccess Real MG Registry: baseline characteristics and patient‐reported outcomes scores from an international myasthenia gravis patient registry

#### F. Amini ^1^; E. Bagshaw^1^; A. Fellows^1^; M. Larkin^1^; C. Petitjean^1^; K. Gwathmey^2^; Z. Mahuwala^3^; F. Sacca^4^; A. Habib^5^; S. Jacob^6^

##### ^*1*^ *Vitaccess Limited, London, UK;* ^*2*^ *Department of Neurology, Virginia Commonwealth University, Richmond, VA, USA;* ^*3*^ *Department of Neurology, University of Kentucky, Lexington, KY, USA;* ^*4*^ *University of Naples, Naples, Italy;* ^*5*^ *UCI Health, Orange, CA, USA;* ^*6*^ *Institute of Immunology and Immunotherapy, University of Birmingham, Birmingham, UK*

## EPV‐1281

### When immunotherapy unlocks the door to autoimmunity: A case report of MOGAD triggered by Pembrolizumab in a patient with Gray Zone Lymphoma

#### M. Rima ^1^; C. Di Carmine^2^; A. D'Andreagiovanni^3^; E. Pennese^4^; M. De Angelis^2^; S. Sensi^1^

##### ^*1*^ *Department of Neuroscience, Imaging and Clinical Science, University “G. D'Annunzio” of Chieti‐Pescara, Italy;* ^*2*^ *Emergency Department‐ Emergency Neurology and Stroke Unit – “S. Spirito” Hospital, Pescara, Italy;* ^*3*^ *Neurophysiopathology and Neurodegenerative Disorders Unit – “S. Spirito” Hospital, Pescara, Italy;* ^*4*^ *Department of Haematogy‐Oncology – “S. Spirito” Hospital, Pescara, Italy*

## EPV‐1282

### When miller fisher strikes twice: A rare case of recurrent miller fisher syndrome

#### A. Tsimpiktsioglou; M. Ioakeimidis; T. Doskas

##### Neurology Department, Athens Naval Hospital, Athens, Greece

## EPV‐1283

### When the eyes dance and the gait fails: A case of anti‐GFAP associated opsoclonus‐myoclonus‐ataxia

#### A. Barbosa; A. Correia; M. Carvalho; F. Dourado Sotero; A. Antunes; L. Albuquerque

##### Neurology Department, Department of Neurosciences and Mental Health, Santa Maria Hospital, Lisbon, Portugal

## Neuroinformatics

## EPV‐1284

### Artificial intelligence in neurology: Preliminary results of a nationwide survey among Spanish neurologists

#### A. De Luca; M. Vargas Cobos; R. algonzo González; C. Gómez López de San Román; L. Caballero Sánchez; I. Bermejo Casado; D. Cerdán Santacruz; J. Eguizabal Aguado; A. Castrillo Sanz; A. Mendoza Rodriguez; M. Capra

##### Neurology Service, General Hospital of Segovia, Segovia, Spain

## EPV‐1285

### Beyond craniotomy: Endovascular approach to brain‐computer interface

#### A. Masonis ^1^; Ž. Chomanskis^2^

##### ^*1*^ *Vilnius University, Faculty of Medicine, Vilnius, Lithuania;* ^*2*^ *Vilnius Republican University Hospital, Vilnius, Lithuania*

## EPV‐1286

### Clinical characteristics and outcomes of acute stroke patients based on RES‐Q data in Uzbekistan

#### M. Komiljonova

##### Tashkent State Medical University, Tashkent, Uzbekistan

## EPV‐1287

### Data sharing in neurology: An analysis of the data availability statements in the European Journal of Neurology 2024–2025

#### U. Karadkar ^1^; W. Struhal^2^

##### ^*1*^ *School of Information, The University of Texas at Austin, Austin, USA;* ^*2*^ *Department of Neurology, University Hospital Tulln, Tulln, Austria – NOE LGA, Karl Landsteiner University, Austria*

## EPV‐1288

### Leveraging single‐cell and spatial omics for brain tumor insights to improve therapeutic strategies

#### S. Roy^1^; M. Nassor^2^; F. Zahin^3^; S. Chaudhry ^4^; K. K. Odei^5^; P. Nkrumah‐Boateng^6^; A. Wireko^7^

##### ^*1*^ *Hull York Medical School, University of York, York, UK;* ^*2*^ *School of Medicine, University of Sunderland, Sunderland, UK;* ^*3*^ *Medical School, The University of Buckingham, Buckingham, UK;* ^*4*^ *Medical School, The University of Edinburgh, Edinburgh, UK;* ^*5*^ *Independent Researcher, Toronto, Canada;* ^*6*^ *University of Ghana Medical School, Accra, Ghana;* ^*7*^ *Icormed Research Collaborative, Berlin, Germany*

## EPV‐1289

### Lipid induced energetic lability in serotonin and dopamine transporter assembly could be resisted by truncation isoforms

#### T. Karagöl; A. Karagöl

##### Istanbul University, Istanbul Faculty of Medicine, Istanbul, Türkiye

## EPV‐1290

### Network‐based identification of strategic research opportunities in neurologic disorders: A bibliometric and graph analytics approach

#### I. Filchenko^1^; N. Cihoric ^2^; A. Shaheen^3^; F. Dennstädt^2^

##### ^*1*^ *Department of Neurology, Inselspital, University Hospital and University of Bern, Bern, Switzerland;* ^*2*^ *Department of Radiation Oncology, Inselspital, University Hospital and University of Bern, Bern, Switzerland;* ^*3*^ *Alexandria Faculty of Medicine, Alexandria, Egypt*

## EPV‐1291

### Research frontiers and gaps across 3,343 neurological diseases: A systematic bibliometric analysis of the PubMed database

#### I. Filchenko^1^; N. Cihoric ^2^; A. Shaheen^3^; F. Dennstädt^2^

##### ^*1*^ *Department of Neurology, Inselspital, University, Switzerland Hospital and University of Bern, Switzerland;* ^*2*^ *Department of Radiation Oncology, Inselspital, University Hospital and University of Bern, Switzerland;* ^*3*^ *Alexandria Faculty of Medicine, Alexandria, Egypt*

## EPV‐1292

### Reverse‐QTY engineering converts soluble glutamate transporter isoforms into high‐affinity inhibitors

#### A. Karagöl; T. Karagöl

##### Istanbul University, Istanbul Faculty of Medicine, Istanbul, Türkiye

## EPV‐1293

### Validation of brain fog scale in Farsi

#### F. Hojjatipour

##### Neurology, Shahid Beheshti University of Medical Sciences, Sydney, Australia

## Neurological manifestation of systemic diseases

## EPV‐1294

### Acute bilateral visual loss as the initial manifestation of Wernicke encephalopathy: A rare case report

#### J. Kim; H. Kim; G. Nam; S. Cho

##### Neurology, Hallym University Dongtan Sacred Heart Hospital, Hwaseong, Republic of Korea

## EPV‐1295

### Acute confusional syndrome as the initial presentation of hemophagocytic syndrome secondary to high‐grade lymphoma

#### B. Quintana López; A. O. Sanchez; D. Alonso Vallin; V. García Gracia; C. Castaño Ron; P. Diaz Aguirre; M. Cabezas Arias

##### Neurology, University Hospital of Cabueñes, Gijón, Spain

## EPV‐1296

### an unusual cause of axonal polyradiculoneuropathy: Metastatic infiltration from nasopharyngeal carcinoma

#### D. Cerdán Santacruz; M. Vargas Cobos; A. De Luca; R. Alfonso González; M. Capra; C. Gómez López de San Román; I. Bermejo Casado; L. Caballero Sánchez; A. Castrillo Sanz; G. Suárez Fernández; A. Mendoza Rodríguez

##### Universitary General Hospital of Segovia, Segovia, Spain

## EPV‐1297

### Anti‐Hu mediated small‐fiber neuropathy: A rare manifestation of small‐cell neuroendocrine prostate cancer

#### G. Fernandes; L. Ache

##### Neurology, Hospital Moinhos de Vento, Porto Alegre, Brazil

## EPV‐1298

### Anti‐PL‐7 (Anti‐Threonyl‐tRNA Synthetase) positive myopathies: Two distinct clinical cases

#### A. Başel Pala; K. Işik; Ö. Çakir Nartsoy; G. Taşçatan; E. Aciman Demirel; M. Açikgöz; U. Çelebi; H. Atasoy

##### Department of Neurology, Zonguldak Bülent Ecevit University Faculty of Medicine, Zonguldak, Türkiye

## EPV‐1299

### Anti‐seizure and anti‐epileptic effects of angiotensin receptor blockers in patients after acute stroke in observational studies

#### Z. Dr. Kiss; B. Fülöp; Z. Hermann; B. Jassoy; A. Koszta; K. Kovács‐Kusza; K. Magyari‐Zuberecz; E. Pordány‐Bagoly; K. Radó; D. Dani; G. Szegleti; V. Varga; Z. Szupera

##### Neurology and Stroke Department, DBC‐Szent Imre Teaching Hospital, Budapest, Hungary

## EPV‐1300

### Anxiety and depression in overt and subclinical hypothyroidism: Single‐centre observational study

#### B. Vosikov; S. Magzumova; K. Kuchkarov; M. Rajapov

##### Department of Psychiatry and Narcology, Tashkent State Medical University, Tashkent, Uzbekistan

## EPV‐1301

### Atypical neurological presentation of autoimmune polyglandular syndrome type 2: A case report

#### N. Singh i Kaur; L. Rosés Hernansáez; G. Rumaldo Gutti; C. Guerrero Castaño

##### Neurology, Hospital Universitari Germans Trias i Pujol, Badalona, Spain

## EPV‐1302

### Bedtime vs morning antihypertensive dosing: cardiovascular and neurovascular outcomes — A systematic review & trial sequential analysis

#### M. Pandit ^1^; M. Ali^2^; FNU. Sawaira^3^; A. Ali^4^; A. Jamal^4^; S. Khan^4^; M. Gul^5^; J. Afridi^6^; A. Khan^4^; M. Yasir^7^; A. Zahid^8^; M. Shah^4^; J. Ikram^8^

##### ^*1*^ *Faculty of Medicine, Medical University of Plovdiv, Plovdiv, Bulgaria;* ^*2*^ *Department of Molecular Medicine, University of Pavia, Italy;* ^*3*^ *Khyber Girls Medical College, Peshawar, Pakistan;* ^*4*^ *Khyber Medical College, Peshawar, Pakistan;* ^*5*^ *The Wright Center for Graduate Medical Education, Scranton, PA, USA;* ^*6*^ *St. Lukes Hospital, St. Louis, MO, USA;* ^*7*^ *Bacha Khan Medical College, Mardan, Pakistan;* ^*8*^ *The Cleveland Clinic Foundation, Cleveland, Ohio, USA*

## EPV‐1303

### Blind to the diagnosis: Marchiafava‐Bignami disease

#### L. Heap ^1^; I. Coyle‐Gilchrist^1^; J. Saada^2^; M. Cao^1^

##### ^*1*^ *Neurology, Norfolk and Norwich University Hospital, Norfolk, UK;* ^*2*^ *Radiology, Norfolk and Norwich University Hospital, Norfolk, UK*

## EPV‐1304

### Central nervous system demyelination in primary Sjögren's syndrome: Clinical, imaging and antibody findings in seven patients

#### K. Chekili^1^; H. Derbali^1^; Y. Kooli ^1^; Y. Mrad^1^; I. Bedoui^1^; M. Messelmani^1^; M. Mansour^1^; N. Fekih Mrissa^2^; J. Zaouali^1^

##### ^*1*^ *Department of Neurology, Military Hospital of Tunis, Tunisia;* ^*2*^ *Molecular Biology Unit (UR17DN06), Laboratory of Hematology, Military Hospital of Tunis, Tunisia*

## EPV‐1305

### Cognitive and neurological manifestations in rheumatoid arthritis: Clinical and inflammatory correlates

#### M. Abzalova; M. Yakubova; N. Abdurakhmanova; Z. Urakova

##### Tashkent State Medical University, Tashkent, Uzbekistan

## EPV‐1306

### Cognitive impairment as an initial manifestation of diffuse large B‐cell lymphoma: A case report

#### P. Dodu; Y. López‐Moreno; P. Cabezudo‐García; I. Rodriguez‐Lavado; V. Castro‐Sánchez

##### Neurology Department, Regional Universitary Hospital of Málaga, Málaga, Spain

## EPV‐1307

### Fluctuating encephalopathy and vomiting in adolescence as a presentation of late onset ornithine transcarbamylase deficiency

#### L. García‐Granados ^1^; M. de Miguel‐Escribano^2^; J. Andrada‐Moreno^1^; I. Lopera‐Rodríguez^1^; J. Ciriero‐Macías^1^; A. Luque‐Ambrosiani^1^; J. Román‐Rueda^1^; A. Palomino‐García^1^; M. Pérez‐Quintana^2^

##### ^*1*^ *Department of Neurology, Hospital Universitario Virgen del Rocío, Sevilla, Spain;* ^*2*^ *Autoimmune and Rare Diseases Unit, Department of Internal Medicine, Hospital Universitario Virgen del Rocío, Sevilla, Spain*

## EPV‐1308

### From lung to brain: Invasive aspergillosis by *Aspergillus nidulans* in lung transplant recipient and stroke mimic case

#### J. Celis ^1^; G. Roncancio^2^; D. Celis^3^; S. Urrego^4^; F. Campo^5^; O. Munoz^6^; J. Botero^7^; N. Uribe^7^; A. Londono^7^; R. Zapata^8^; C. Montoya^8^; C. Garcia^2^

##### ^*1*^ *Neurology Service Clinica Cardio Vid/Universidad Pontificia Bolivariana, Medellin, Colombia;* ^*2*^ *Infectology Service Clinica Cardio Vid/Univesrsidad Pontificia Bolivariana, Medellin, Colombia;* ^*3*^ *Neumology Service Clinica Cardio Vid/Universidad Pontificia Bolivariana/Universidad Ces Medellin, Colombia;* ^*4*^ *Neurology Service Clinica Cardio Vid/Universidad De Antioquia Medellin, Colombia;* ^*5*^ *Neumology Service Clinica Cardiovid/Clinica Universitaria Bolivariana Medellin, Colombia;* ^*6*^ *Neumology Service Clinica Cardio Vid/Unioversidad Ces Medellin, Colombia;* ^*7*^ *Neumologuy Service Clinica Cardio Vid Medellin, Colombia;* ^*8*^ *Thoracic Surgery Clinica Cardio Vid Medellin, Colombia*

## EPV‐1309

### Hypertrophic pachymeningitis associated with Polyarteritis Nodosa: An unusual cause of headache and visual disorders

#### J. Huang ^1^; R. Guo^1^; Y. Sun^1^; Z. Jia^1^; L. Gao^2^; B. Li^1^

##### ^*1*^ *Department of Neurology, The Second Hospital of Hebei Medical University, Shijiazhuang, China; Key Laboratory of Neurology (Hebei Medical University), Ministry of Education, China; Key Laboratory of Neurology of Hebei Province, Shijiazhuang, China;* ^*2*^ *Department of Rheumatology and Immunology, The Second Hospital of Hebei Medical University, Shijiazhuang, China*

## EPV‐1310

### Intracerebral hemorrhage as the initial presentation of severe undiagnosed immune thrombocytopenic purpura

#### R. Mesa Martínez; J. Tejada García; L. García Tuñón Villaluenga

##### Neurology, Complejo Asistencial Universitario de León (CAULE), León, Spain

## EPV‐1311

### Invisible myelopathy: Lupus myelitis overlapping with anti‐AQP4 NMOSD

#### J. Marín Hinojosa; V. Delgado Gil; V. Fernández; J. Ruiz García; L. Rivas Ramos; M. Nieva; E. Morales García

##### Neurology, Virgen de la Victoria University Hospital, Málaga, Spain

## EPV‐1312

### Lithium: From stabilizer to disruptor

#### R. Alfonso González; M. Vargas Cobos; A. C. De Luca; M. Capra; C. Gómez López de San Román; L. Caballero Sánchez; I. Bermejo Casado; J. Eguizábal Aguado; D. Cerdán Santacruz; A. Castrillo Sanz; A. Mendoza Rodríguez

##### Service of Neurology, Universitary Hospital of Segovia, Segovia, Spain

## EPV‐1313

### MELAS presenting as Anton syndrome: Beyond diagnostic challenges

#### S. Marinho Pinto ^1^; J. Menezes Barbosa^1^; C. Semedo^2^; J. Pereira Alves^1^

##### ^*1*^ *Neurology Department, ULS da Arrábida, Setúbal, Portugal;* ^*2*^ *Neurology Department, ULS de São José, Lisbon, Portugal*

## EPV‐1314

### Misdiagnosed schizoaffective disorder revealing Fahr's syndrome secondary to hypoparathyroidism: A case report

#### A. Afanasieva; M. Liksunova; M. Bodnar; T. Slobodin

##### Department of Neurology, LLC “Dobrobut‐Clinic”, Kyiv, Ukraine

## EPV‐1315

### Misfortunes never come singly – Neurological involvement in hematologic malignancy

#### M. Docan ^1^; A. Hanganu^1^; A. Dulamea^2^

##### ^*1*^ *Fundeni Clinical Institute, București, Romania;* ^*2*^ *Carol Davila University of Medicine and Pharmacy, Bucharest, Romania*

## EPV‐1316

### Neuropsychological burden, stigma and social participation in autoimmune vitiligo: A cross‐sectional study

#### M. Rajapov; K. Kuchkarov; B. Vosikov; N. Yadgarova

##### Department of Psychiatry and Narcology, Tashkent State Medical University, Tashkent, Uzbekistan

## EPV‐1317

### New‐onset generalized seizure as the initial manifestation of Neuropsychiatric SLE (NPSLE)

#### R. Usman ^1^; M. Firdus^2^

##### ^*1*^ *Dr. Akbar Niazi Teaching Hospital, Islamabad, Pakistan;* ^*2*^ *Fauji Foundation Hospital Rawalpindi, Pakistan*

## EPV‐1318

### Numb chin syndrome: A silent warning sign

#### F. Sánchez García; M. González Gómez; E. Gismera Fontes; L. Cabral Esquea; A. Mena Bravo

##### Neurology, University Hospital of Guadalajara, Guadalajara, Spain

## EPV‐1319

### Oligoclonal bands in diabetic lumbosacral radiculoplexus neuropathy: An unusual presentation of Bruns–Garland syndrome

#### M. Alvarez‐Gutierrez; P. Mayo‐Rodríguez; A. Lafuente‐Almaden; J. Melgar‐Jara; L. Dominguez‐Jimenez; M. Bonnin‐Bravo; E. Lopez‐Valdes; M. Garcia‐Ruiz

##### Neurology Department, Hospital Clinico San Carlos, Madrid, Spain

## EPV‐1320

### Rapidly progressive cognitive decline as an elderly onset of neurological symptoms in an 80‐year‐old woman with MELAS

#### J. Melgar Jara; M. García Ruiz; E. López‐Valdez; A. Lafuente‐Almaden; L. Dominguez Jimenez; M. Bonin Bravo; M. Alvarez Gutierrez; P. Mayo Rodriguez

##### Neurology Department, Hospital Clinico San Carlos, Madrid, Spain

## EPV‐1321

### Recurrent Wernicke‐Korsakoff syndrome of non‐alcoholic etiology: A case report

#### J. Ciriero Macías ^1^; I. Lopera Rodríguez; J. Andrada Moreno; L. García Granados; L. Fernández Espigares; J. Román Rueda; A. Luque Ambrosiani

##### Neurology/Neurophysiology, Hospital Universitario Virgen del Rocío, Seville, Spain

## EPV‐1322

### Rising mortality trends related to type 2 diabetes mellitus and cardiac arrest among american adults: A 21‐year national analysis

#### M. Ali ^1^; M. Pandit^2^; E. Saghir^3^; B. Ali^3^; M. Hashmi^4^; N. Shoaib^4^; H. Mateen^4^; M. Saghir^4^; A. Abdullah^4^; M. Ahsan^4^; N. Bhatt^5^

##### ^*1*^ *Department of Molecular Medicine, University of Pavia, Pavia, Italy;* ^*2*^ *Faculty of Medicine, Medical University of Plovdiv, Plovdiv, Bulgaria;* ^*3*^ *Department of Medicine, Dow University of Health Sciences, Karachi, Pakistan;* ^*4*^ *Department of Medicine, Jinnah Sindh Medical University, Karachi, Pakistan;* ^*5*^ *Department of Internal Medicine, HCA Houston Healthcare Clear Lake, Texas, USA*

## EPV‐1323

### Role of bowel wash in recovery from hepatic encephalopathy

#### V. Nandmer

##### Gandhi Medical College Hamidia Hospital Bhopal, Bhopal, Madhya Pradesh

## EPV‐1324

### Spectrum of non‐psychotic neuropsychiatric syndromes in women with alimentary obesity: A clinical study

#### N. Talipova; M. Kevorkova; N. Yadgarova

##### Department of Psychiatry and Narcology, Tashkent State Medical University, Tashkent, Uzbekistan

## EPV‐1325

### The neuroprotective effect of dual GIP/GLP1‐RA agonists: A scoping review of preclinical and clinical evidence

#### Y. Hawas ^1^; M. Abouzid^2^; A. Gadelmawla^3^; D. Ewis^4^; A. Khatatbeh^5^; Y. Negida^6^; A. Negida^7^

##### ^*1*^ *Faculty of Medicine, Tanta University, Tanta, Egypt;* ^*2*^ *Department of Physical Pharmacy and Pharmacokinetics, Poznan University of Medical Sciences, Poznan, Poland;* ^*3*^ *Faculty of Medicine, Menoufia University, Menoufia, Egypt;* ^*4*^ *Faculty of Medicine, Beni Suef University, Beni Suef, Egypt;* ^*5*^ *Al‐Hussein medical center, Jordanian Royal Medical Services, Amman, Jordan;* ^*6*^ *Faculty of Medicine, Zagazig University, Egypt;* ^*7*^ *Department of Neurology, Virginia Commonwealth University, Richmond, Virginia, USA*

## EPV‐1326

### Thirty‐year trends in late and late latent syphilis in the United States: National, sex‐specific, and regional patterns

#### S. Wasti^1^; A. Wasti^2^; M. Rahim ^1^

##### ^*1*^ *Department of Neurology, King Edward Medical University, Lahore, Pakistan;* ^*2*^ *Department of Neurology, Continental Medical College, Lahore, Pakistan*

## EPV‐1327

### Transverse myelitis revealing primary Sjögren's syndrome in elderly patients: A case series

#### F. Ben Chihi ^1^; H. Derbali^1^; I. Bedoui^1^; Y. Mrad Dali^1^; M. Messelmani^1^; M. Mansour^1^; N. Fekih Mrissa^2^; J. Zaouali^1^

##### ^*1*^ *Department of Neurology, Military Hospital of Tunis, Tunisia;* ^*2*^ *Molecular Biology Unit (UR17DN06), Laboratory of Hematology, Military Hospital of Tunis, Tunisia*

## EPV‐1328

### Treosulfan vs Busulfan for HSCT: A meta‐analysis of key outcomes

#### M. Ali^1^; M. Pandit ^2^; M. Khalid^3^; FNU. Sawaira^4^; F. Khan^5^; U. Rehman^6^; A. Bisola^7^; A. Khan^8^; M. Kiwan^9^; A. Akhtar^10^; A. Ali^11^; A. Janjua^12^; S. Aijaz^13^; T. Patel^14^

##### ^*1*^ *Department of Molecular Medicine, University of Pavia, Pavia, Italy;* ^*2*^ *Faculty of Medicine, Medical University of Plovdiv, Plovdiv, Bulgaria;* ^*3*^ *Department of Surgery, Karachi Medical and Dental College, Karachi, Pakistan;* ^*4*^ *Khyber Girls Medical College, Peshawar, Pakistan;* ^*5*^ *Ayub Medical College, Abbottabad, Khyber Pakhtunkhwa, Pakistan;* ^*6*^ *Khyber Medical College, Peshawar, Pakistan;* ^*7*^ *Faculty of Medicine and Pharmacy, Cadi Ayyad University, Marrakech, Morocco;* ^*8*^ *Department of Medicine, Saidu Medical College, Swat, KPK, Pakistan;* ^*9*^ *Gomal Medical College, Dera Ismail Khan, KPK, Pakistan;* ^*10*^ *Ziauddin Medical College, Ziauddin University, Karachi, Pakistan;* ^*11*^ *Khyber Teaching Hospital, Peshawar, Pakistan;* ^*12*^ *King Edward Medical University, Lahore, Pakistan;* ^*13*^ *United Medical and Dental College, Karachi, Pakistan;* ^*14*^ *American University of Antigua College of Medicine, Osbourn, Antigua and Barbuda*

## EPV‐1329

### When water becomes neurotoxic: Acute hyponatremia causing seizure in chronic polydipsia

#### M. Stefanache ^1^; A. Iacob^1^; I. Radu^1^; C. Sîrbu^2^

##### ^*1*^ *Department of Neurology, Central Military Emergency University Hospital “Dr. Carol Davila”, Bucharest, Romania;* ^*2*^ *Clinical Neuroscience Department, Carol Davila University of Medicine and Pharmacy, Bucharest, Romania*

## EPV‐1330

### Indian classical music and the brain: Neurological insights into rhythm, emotion, and cognition

#### S. Dubey

##### Department of Medicine, Jawaharlal Nehru Medical College, Wardha, India

## Neurology and arts

## EPV‐1331

### Neurology on canvas

#### J. Marín Hinojosa ^1^; J. Cazorla Quintero^2^

##### ^*1*^ *Neurology, Virgen de la Victoria University Hospital, Málaga, Spain;* ^*2*^ *Psychiatry. Virgen de la Victoria University Hospital, Málaga, Spain*

## EPV‐1332

### What happens if it's not dementia?

#### R. Surti ^1^; A. Shaiju^2^

##### ^*1*^ *Medical Student, University of Leeds, Leeds, UK;* ^*2*^ *Medical Student, University of Leeds, Leeds, UK*

## Neuro‐oncology

## EPV‐1333

### A reversible mimic of dementia with Lewy bodies: Anti‐LGI1 encephalitis

#### I. Alcobendas; B. Lucio Ceballos; J. Botía Barberá; A. Martínez; A. Contreras Chicote; J. García Domínguez; G. Lafuente Gómez

##### Neurology, Hospital General Universitario Gregorio Marañón, Madrid, Spain

## EPV‐1334

### An integrated framework for epileptogenicity, diagnostic evaluation and clinical management of pediatric low‐grade epilepsy‐associated tumors

#### A. Katramadou; D. Kanakis

##### Laboratory of Pathology, Basic Sciences,University of Nicosia Medical School, Nicosia,Cyprus

## EPV‐1335

### Assessment of cognitive functions in glial brain tumors

#### D. Berdibayeva; N. Ryskeldiyev; A. Kabykenova; A. Moldabekov

##### National Centre for Neurosurgery, Astana, Kazakhstan

## EPV‐1336

### Autoimmune GFAP astrocytopathy encephalomyelitis and ovarian teratoma: A case report and etiological differential diagnosis

#### A. Rodríguez Herrera; S. López Anguita; F. Valenzuela Rojas; J. Rodríguez Quinchanegua; A. Baz Cárdenas; V. Torres do Rego

##### Neurology, Hospital Central de la Defensa, Madrid, Spain

## EPV‐1337

### Bloomy rind sign as a diagnostic clue in IDH‐wild‐type brainstem glioma

#### I. Saurina Navarro ^1^; C. Martínez Follana^1^; M. Marín Rubio^1^; E. Campanya Marti^1^; M. García Huguet^1^; A. Gifreu Fraixinó^2^; J. Gutiérrez Naranjo^2^; A. Boix Lago^2^; G. Alvarez Bravo^2^

##### ^*1*^ *Department of Neurology, Hospital Doctor Josep Trueta, Girona, Spain;* ^*2*^ *Territorial Neuroimmunology and Multiple Sclerosis Unit of Girona, Hospital Doctor Josep Trueta, Girona – Hospital Santa Caterina, Salt, Spain*

## EPV‐1338

### Bridging the gap: Evaluating FLASH radiotherapy in the context of glioblastoma

#### E. De Santis ^1^; M. Scalera^2^; F. Di Martino^3^; A. Flori^4^; A. Cavalieri^5^; M. Celentano^6^; F. Paiar^7^; N. Giannini^7^; S. Capaccioli^5^; F. Raimondi^1^; E. Vannini^2^; M. Costa^2^

##### ^*1*^ *Bio@SNS Lab, Scuola Normale Superiore, Pisa, Italy;* ^*2*^ *Neuroscience Institute, CNR, Pisa, Italy;* ^*3*^ *Azienda ospedaliero‐universitaria pisana, U.O. Fisica Sanitaria, Pisa, Italy;* ^*4*^ *“G. Monasterio” Foundation – Regione Toscana, Italy;* ^*5*^ *University of Pisa, Center for instrument Sharing of the University of Pisa (CISUP), Pisa, Italy;* ^*6*^ *National Institute of Nuclear Physics, Section of Pisa, Italy;* ^*7*^ *University of Pisa, Department of Translational Research and New Technologies in Medicine and Surgery, Pisa, Italy*

## EPV‐1339

### Calcifications in extra‐axial metastases from prostate adenocarcinoma: A case report

#### A. De Luca; M. Vargas Cobos; R. Alfonzo González; M. Capra; C. Gómez López de San Román; L. Caballero Sánchez; I. Bermejo Casado; D. Cerdán Santacruz; A. Castrillo Sanz; A. Mendoza Rodriguez

##### Neurology Service, General Hospital of Segovia, Segovia, Spain

## EPV‐1340

### CAR‐T–Related myelopathy after anti‐BCMA therapy: The first reported case in plasma cell leukemia

#### I. Alcobendas Liern; B. Lucio Ceballos; J. Botía Barberá; R. Leal Hidalgo; G. Lafuente Gómez

##### Neurology, Hospital General Universitario Gregorio Marañón, Madrid, Spain

## EPV‐1341

### Cavernous sinus squamous cell carcinoma masquerading as sinusitis: a diagnostic challenge

#### A. Quka ^1^; I. Zekja^1^; D. Disha^1^; A. Xhumari^2^; J. Kruja^1^

##### ^*1*^ *Department of Neurology, UHC Mother Teresa, Tirana, Albania;* ^*2*^ *Department of Neurosurgery, UHC Mother Teresa, Tirana, Albania*

## EPV‐1342

### Cerebral venous thrombosis associated with immune checkpoint inhibitor therapy in malignant melanoma: A case report

#### R. Chiujdea ^1^; T. Dragan^1^; N. Enache^1^; B. Ciocanescu^1^; I. Murasan^1^; C. Falup‐Pecurariu^2^

##### ^*1*^ *Department of Neurology, County Clinic Hospital, Brasov, Romania;* ^*2*^ *Department of Neurology, County Clinic Hospital, Transilvania University Brasov, Romania*

## EPV‐1343

### Demyelination of the central nervous system CNS associated with cancer immunotherapy

#### V. Lizhdvoy

##### Department of Neurology, Moscow Regional Research Clinical Institute n.a. M,F, Vladimirskiy, Moscow, Russian Federation

## EPV‐1344

### Diagnosis of brain tumors in pediatric emergency department‐single center experience

#### S. Popovic ^1^; R. Kravljanac^1^; M. Milić^2^; A. Dimić^1^; S. Ostojić^1^; G. Kovačević^1^; B. Vučetić‐Tadić^1^; J. Bedjik^1^; V. Oparnica^1^

##### ^*1*^ *Department of neurology, Institute for Mother and Child Healthcare of Serbia, Faculty of Medicine University of Belgrade, Beograd, Serbia;* ^*2*^ *Department of Neurosurgery, University Clinical Center of Serbia, Faculty of Medicine University of Belgrade, Beograd, Serbia*

## EPV‐1345

### Effects of memantine on temozolomide‐induced apoptotic signaling in glioma cells

#### B. Erbaş ^1^; M. Cevik^2^; B. Ozgur^3^; G. Demircan^2^

##### ^*1*^ *Department of Pharmacology, Demiroglu Bilim University Faculty of Medicine, Istanbul, Türkiye;* ^*2*^ *Department of Medical Biology and Genetics, Demiroglu Bilim University Faculty of Medicine, Istanbul, Türkiye;* ^*3*^ *Demiroğlu Bilim University, School of Health Services, Istanbul, Türkiye*

## EPV‐1346

### Inter‐perforator microdebulking: A vascular‐based approach to clinoidal meningioma resection

#### K. Watanabe^1^; S. Madireddy ^2^; Y. Murayama^1^; K. Ishikawa^1^

##### ^*1*^ *The Jikei University School of Medicine, Department of Neurosurgery, Tokyo, Japan;* ^*2*^ *The University of Tokyo School of Medicine, Department of Neurology, Tokyo, Japan*

## EPV‐1347

### Intravascular lymphoma mimicking a longitudinally extensive transverse myelitis: A case series and review of the literature

#### A. Vargas‐Verdaguer ^1^; K. Matute^2^; M. Ruiz‐Vives^3^; C. del Valle‐Vargas^1^; C. Vázquez^1^; E. Hernández^4^; C. Bracke^5^; A. Romero^5^; A. Riveros^6^; B. Xicoy^7^; C. Carrato^2^; I. Aldecoa^8^; M. Ortega^9^; R. Esgueva^10^; Y. Blanco^3^; J. Cabrera‐Maqueda^3^; S. Presas^1^; C. Izquierdo^1^; A. González‐Garcia^1^; C. Guerrero^1^

##### ^*1*^ *Department of Neurology, Hospital Universitari Germans Trias i Pujol. Badalona, Spain.;* ^*2*^ *Department of Pathology, Hospital Universitari Germans Trias i Pujol, Badalona, Spain;* ^*3*^ *Neuroimmunology and Multiple Sclerosis Unit, Laboratory of Advanced Imaging in Neuroimmunological Diseases, Hospital Clinic of Barcelona, IDIBAPS, and Universitat de Barcelona, Spain;* ^*4*^ *Department of Radiology, Hospital Universitari Germans Trias i Pujol, Badalona, Spain;* ^*5*^ *Department of Infectious Diseases, Hospital Universitari Germans Trias i Pujol, Badalona, Spain;* ^*6*^ *Department of Reumathology, Hospital Universitari Germans Trias i Pujol, Badalona, Spain;* ^*7*^ *Department of Hemathology, Hospital Universitari Germans Trias i Pujol, Badalona, Spain;* ^*8*^ *Department of Pathology, Biomedical Diagnostic Center, Hospital Clinic of Barcelona, University of Barcelona and Neurological Tissue Bank, Biobanc‐Hospital Clínic‐FRCB/IDIBAPS, Barcelona, Spain;* ^*9*^ *Institut de Medicina Legal i Ciències Forenses de Catalunya (IMLCFC), Spain;* ^*10*^ *Institut de Medicina Legal i Ciències Forenses de Catalunya (IMLCFC) and Banc de Sang i Teixits (BSC), Spain*

## EPV‐1348

### Isolated inflammatory leptomeningitis after CAR‐T therapy: ICANS or TIAN? An atypical case repor

#### W. Jakmajian ^1^; R. Ursu^1^; N. Cainic^1^; A. Velly^2^; C. Thieblemont^2^; A. Carpentier^1^; C. Barsan^1^; S. Cuzzubo^1^; C. Cristinelli^2^

##### ^*1*^ *Neurology, Saint Louis Hospital, Paris, France;* ^*2*^ *Hemato‐oncology, Saint‐Louis Hospital, Paris, France*

## EPV‐1349

### Longitudinally extensive spinal cord lesion mimicking autoimmune myelitis: A case of primary spinal glioblastoma

#### S. Stasinou; Z. Gritsopoulou; A. Tsimpiktsioglou; K. Aslanidou; G. Nakkas; M. Ioakeimidis; T. Doskas

##### Neurology Clinic, Athens Naval Hospital, Athens, Greece

## EPV‐1350

### Mild Encephalitis/Encephalopathy with a Reversible Splenial Lesion (MERS) in acute myeloid leukemia during pregnancy: A case report

#### S. Rachidi ^1^; I. Abourachida; Y. Zakaria; M. Chraa; N. Louhab; S. Zahlan

##### Neurology Department, Mohammed VI University Hospital, Faculty of Medicine and Pharmacy, Marrakech, Morocco

## EPV‐1351

### Myeloradiculitis associated with cancer: A diagnostic challenge

#### M. Costa ^1^; D. Matos^1^; R. Lobato^1^; M. Rodrigues^2^; D. Ferreira^1^

##### ^*1*^ *Department of Neurology, Hospital de Santa Luzia, ULSAM, Viana do Castelo, Portugal;* ^*2*^ *Department of Neuroradiology, ULS Gaia/Espinho, Portugal*

## EPV‐1352

### Myositis–myocarditis syndrome secondary to Immune Checkpoint Inhibitor drugs and experience with Abatacept–Ruxolitinib in a tertiary hospital

#### S. García‐Bellido Ruiz; D. Pérez Rangel; G. Velilla Alonso; S. Muñiz Castrillo

##### Neurology Department, 12 de Octubre University Hospital, Madrid, Spain

## EPV‐1353

### Neurological complications in hematological malignancies: A retrospective national cohort analysis

#### N. Chakhoian; A. Ter‐Grigoryan; M. Melik‐Andreasyan; L. Sahakyan; H. Grigoryan

##### Yeolyan Hematology and Oncology Center, Yerevan, Armenia

## EPV‐1354

### Outcome prediction in skull‐base neurosurgery: A preliminary analysis

#### F. Casaccio ^1^; P. Ferroli^2^; M. Broggi^2^; M. Moretti^2^; M. Leonardi^3^; E. Soldini^3^; G. Camarda^3^; G. Anzano^2^; G. Baroni^1^

##### ^*1*^ *Department of Electronics, Information and Bioengineering, Politecnico di Milano, Milan, Italy;* ^*2*^ *Department of Neurosurgery, Forndazione IRCCS Istituto Neurologico Carlo Besta, Milan, Italy;* ^*3*^ *Neurology, Public Health and Disability Unit, Fondazione IRCCS Istituto Neurologico Carlo Besta, Milan, Italy*

## EPV‐1355

### Paraneoplastic sensory ganglionopathy unveiling bronchogenic carcinoma in a heavy smoker

#### G. Louzada de Souza

##### Department of Neurology, Federal University of Triângulo Mineiro, Uberaba, Brazil

## EPV‐1356

### Person in the Barrel Syndrome following immune checkpoint inhibitor therapy: Bilateral brachial plexopathy as a form of peripheral neurotoxicity

#### R. Olaizola Diaz ^1^; A. Bonilla Tena^2^; I. Lera Ramirez^2^; O. Uriz Bacaicoa^4^; R. Leal Hidalgo^1^; I. Catalina^2^; A. Alungulense^2^; L. del Pino Tejado^1^; N. Ramirez Merino^3^; G. Lafuente Gomez^2^

##### ^*1*^ *Neurology, HM Madrid Hospital, Madrid, Spain;* ^*2*^ *Neurology, Gregorio Marañon Hospital, Madrid, Spain;* ^*3*^ *Oncology, Gregorio Marañon Hospital, Madrid, Spain;* ^*4*^ *Neurology, Rey Juan Carlos Hospital, Madrid, Spain*

## EPV‐1357

### Preoperative functional, sociodemographic, and psychological factors as predictors of postoperative outcomes in brain tumor surgery

#### M. Leonardi ^1^; E. Soldini^1^; G. Camarda^1^; G. Anzano^2^; F. Di Meco^2^; M. Moretti^2^; M. Broggi^2^; P. Ferroli^2^

##### ^*1*^ *Neurology, Public Health and Disability Unit. Fondazione IRCCS Istituto Neurologico Carlo Besta, Milan, Italy;* ^*2*^ *Department of Neurosurgery, Fondazione IRCCS Istituto Neurologico Carlo Besta, Milan, Italy*

## EPV‐1358

### Presentation and types of childhood cancer at the Muhimbili National Hospital (MNH), 2023 retrospective observational study

#### M. Arga ^1^; R. Laiti^2^; Y. Hammor^3^; G. Abd El Raheem^1^

##### ^*1*^ *University of Medical Sciences and Technology, Sudan;* ^*2*^ *Muhimbili National Hospital, Tanzania;* ^*3*^ *St Vincent's University Hospital, Dublin, Ireland*

## EPV‐1359

### Prophylactic antiepileptics in glioma: Do they prevent first seizures? A systematic review

#### H. Patel ^1^; R. Mishra^2^; P. Chouksey^3^; L. Salazar^4^; A. Agarwal^5^

##### ^*1*^ *Department of Neurosurgery, All India Institute of Medical Sciences, Bhopal, India;* ^*2*^ *Department of Neurosurgery, All India Institute of Medical Sciences, Bhopal, India;* ^*3*^ *Department of Neurosurgery, All India Institute of Medical Sciences, Bhopal, India;* ^*4*^ *AV Healthcare Innovators, LLC; Madison, WI, USA;* ^*5*^ *Department of Neurosurgery, All India Institute of Medical Sciences, Bhopal, India*

## EPV‐1360

### Rapidly progressive cerebellar syndrome secondary to primary peritoneal carcinoma with positive CV‐2/CRMP5 antibodies: A novel association

#### M. Serrano Alarcon; L. Guirao Guillén; E. Huertas Muñoz; L. Centeno Pons; P. Mayo Rodríguez

##### Neurology Department, Hospital Clínico San Carlos, Madrid, Spain

## EPV‐1361

### Real‐world distribution, diagnostic delay, and management of primary brain tumors in a tertiary neurology center: Insights from a patient‐level cohort

#### F. Daddoveri ^1^; E. Schirinzi^1^; M. Cantarella^2^; N. Montemurro^3^; D. Di Carlo^3^; V. Ortenzi^4^; G. Fanelli^4^; A. Nugumanova^1^; A. Naccarato^4^; G. Masi^5^; F. Acerbi^3^; G. Siciliano^1^

##### ^*1*^ *Neurological Clinic, Department of Clinical and Experimental Medicine, University of Pisa, Italy;* ^*2*^ *Radiotherapy Unit, Azienda Ospedaliero‐Universitaria Pisana (AOU Pisa), Pisa, Italy;* ^*3*^ *Neurosurgery Unit, Azienda Ospedaliero‐Universitaria Pisana (AOU Pisa), Pisa, Italy;* ^*4*^ *Pathology Unit, Azienda Ospedaliero‐Universitaria Pisana (AOU Pisa), Pisa, Italy;* ^*5*^ *Oncology Unit, Azienda Ospedaliero‐Universitaria Pisana (AOU Pisa), Pisa, Italy*

## EPV‐1362

### Survival, response, and safety outcomes of oncolytic viral therapy in pediatric brain tumors: A meta‐analysis

#### P. Pitchan Velammal^1^; G. Srinivasan ^2^

##### ^*1*^ *Neurology, The University of Tennessee Health Science Center‐Memphis, TN, USA;* ^*2*^ *Stanley Medical College, Chennai, India*

## EPV‐1363

### Sustained visual recovery with the MEKi Trametinib in an adult patient with sporadic optic pathway glioma harboring a rare BRAF mutation

#### A. Di Stefano ^1^; F. Villanacci^1^; A. Pisano^1^; L. Testaverde^2^; A. Rallo^3^; V. Giudice^3^; G. Benini^3^; V. Zucchi^4^; R. Della Monica^5^; L. Chiariotti^5^; G. Ferri^6^; I. Giovannoni^7^; E. Sartori^8^; V. Barresi^9^; M. Eoli^10^; M. Touat^11^; S. Cupini^8^; O. Santonocito^1^

##### ^*1*^ *Department of Neurosurgery, Azienda USL Toscana Nord‐ovest, Livorno Hospital, Livorno, Italy;* ^*2*^ *Department of Neuroradiology, Azienda USL Toscana Nord‐ovest, Livorno Hospital, Livorno, Italy;* ^*3*^ *Department of Ophtalmology, Azienda USL Toscana Nord‐ovest, Livorno Hospital, Livorno, Italy;* ^*4*^ *Department of Anatomic Pathology, Azienda USL Toscana Nord‐ovest, Livorno Hospital, Livorno, Italy;* ^*5*^ *CEINGE, Federico II University, Napoli, Italy;* ^*6*^ *Fondazione Pisana per la Scienza, San Giuliano Terme, Italy;* ^*7*^ *Pathology Unit, Bambino Gesù Children's Hospital, IRCCS, Roma, Italy;* ^*8*^ *Department of Oncology, Azienda USL Toscana Nord‐ovest, Livorno Hospital, Livorno, Italy;* ^*9*^ *Neuropathology Unit, Istituto IRCCS C. Besta, Milano, Italy;* ^*10*^ *Neurooncology Unit, Istituto IRCCS C. Besta, Milano, Italy;* ^*11*^ *Department of Neuro‐oncology, Sorbonne University, Inserm, CNRS, UMRS 1127, Paris Brain Institute, AP‐HP Sorbonne University, La Pitié Salpêtrière – Charles Foix Hospital Group, Paris, France*

## EPV‐1364

### The growing burden of central nervous system cancers across central asian states

#### S. Wasti^1^; A. Wasti^2^; M. Rahim ^1^

##### ^*1*^ *Department of Neurology, King Edward Medical University, Lahore, Pakistan;* ^*2*^ *Department of Neurology, Continental Medical College, Lahore, Pakistan*

## EPV‐1365

### The role of genetic mutations in determining immunotherapy response in meningioma patients: A prospective study with MRI monitoring

#### Alya

##### Medicine, Tangerang, Indonesia

## EPV‐1366

### To assess the prevalence of anxiety and depressive disorders in patients with breast cancer, as well as the possibilities for their early detection

#### G. Ernayeva; Z. Ibodullayev; S. Qaraxanova; N. Maxamatjanova

##### Neurology and Medical Psychology Department, Tashkent State Medical University, Tashkent, Uzbekistan

## EPV‐1367

### To evaluate the effectiveness of cognitive‐behavioral therapy in reducing anxiety and depressive symptoms in patients with breast cancer

#### G. Ernayeva; Z. Ibodullayev; N. Maxamatjanova; S. Qaraxanova

##### Neurology and medical psychology department, Tashkent state medical university, Tashkent, Uzbekistan

## EPV‐1368

### Unexpected but not unmanageable: A rare CNS manifestation of Chronic Lymphocytic Leukemia

#### B. Machado Antunes ^1^; F. Dourado Sotero^1^; A. Patrícia Antunes^1^; D. Alves^2^; L. Albuquerque^1^

##### ^*1*^ *Neurology Service, Neurosciences and Mental Health Department, Santa Maria Hospital, Santa Maria Local Health Unit, Lisbon, Portugal;* ^*2*^ *Hematology and Bone Marrow Transplantation Service, Santa Maria Hospital, Santa Maria Local Health Unit, Lisbon, Portugal*

## EPV‐1369

### Unexpectedly high incidence and clinical presentation patterns of glioblastoma: A population‐based study from a defined health area

#### F. Salazar Hernández; M. Ruiz Perelló; B. Gómez Gozálvez; D. López Segura; A. Maija Savolainen; A. Sancho Pedrós; M. Lorente Hernández; D. Vidal Mena; J. Sánchez Villalobos; J. García Carmona; M. Ortega Ortega

##### Neurology Department, Hospital Santa Lucía, Cartagena, Spain

## Neuro‐ophthalmology/ neuro‐otology

## EPV‐1370

### Altered sensory integration in vestibular migraine: Effects of visual input, cervical proprioception and mastoid vibration on postural responses

#### H. Ozdemir ^1^; E. Cortese^2^; P. Upadhyay^2^; I. Valnarov‐Boulter^2^; M. Bancroft^2^; D. Kaski^2^

##### ^*1*^ *Neurology Department, Ege University, Izmir, Türkiye;* ^*2*^ *SENSE Research Unit, Department of Clinical and Movement Neurosciences, Institute of Neurology, University College London, London, UK*

## EPV‐1371

### A disease in the differential diagnosis of orbitopathy: IgG4‐related disease

#### S. Tekin; L. Bir

##### Pamukkale University, School of Medicine, Denizli/Türkiye

## EPV‐1372

### A study to evaluate the risk of osteoporosis and osteopenia in patients with benign paroxysmal positional vertigo

#### T. Makeeva; I. Strelnikova

##### Pain managment center, “Olimp Zdorovya” Medical Center, Voronezh, Russian Federation

## EPV‐1373

### Analysis of catch‐up saccades during video head impulse testing in vestibular migraine

#### H. Ozdemir; M. Yavuz; S. Ibis; F. Gokcay; N. Celebisoy

##### Ege University Medical School, Department of Neurology, İzmir, Turkey

## EPV‐1374

### Anti‐IL‐6 receptor antibody ameliorates impaired visual function in AQP4 peptide‐immunized mice

#### Y. Katsura; Y. Nakatake‐Furuie; S. Miyake; K. Serizawa

##### Product Research Department, Medical Affairs Division, Chugai Pharmaceutical Co., Ltd., Japan

## EPV‐1375

### Artificial intelligence–based assessment of optical coherence tomography in optic nerve disorders: A comparative study of large language models

#### R. Shukla; A. Shukla; A. Mishra

##### All India Institute of Medical Sciences, Raebareli, Uttar Pradesh, India

## EPV‐1376

### Atypical visual involvement in Miller–Fisher syndrome: Case report

#### K. Reyes González ^1^; E. Sarmiento Lizarraga^1^; F. Morales Ramírez^2^; E. Carreón Bautista^2^; E. Otero Cerdeira^2^; L. Ordoñez Boschetti^2^; G. Albert Meza^2^; C. Ramírez Buenrostro^2^

##### ^*1*^ *Internal Medicine, Hospital Español, Ciudad de México, México;* ^*2*^ *Neurology, Hospital Español, Ciudad de México, México*

## EPV‐1377

### Audio and vestibular symptoms in Post Traumatic Stress Disorder

#### A. Di Stadio ^1^; M. Ralli^2^; D. Kaski^3^

##### ^*1*^ *Link University, Rome, Italy;* ^*2*^ *Unicamillus‐International Medical University, Rome, Italy;* ^*3*^ *UCL Queen Square Neurology, London, UK*

## EPV‐1378

### Bilateral abducens nerve palsy following Bexsero (meningococcal group B vaccine) administration – Case report

#### J. Pavlickova

##### Department of Neurology, 1st Faculty of Medicine and General University Hospital in Prague, Czech Republic

## EPV‐1379

### Central retinal artery occlusion – The tip of the iceberg of cerebrovascular disease

#### A. Cheșnoiu‐Dobrota ^1^; S. Stefan^1^; F. Antochi^1^; A. Ribigan^2^; B. Dorobat^1^; D. Nicula^1^; A. Pavel^1^

##### ^*1*^ *University Emergency Hospital, Bucharest, Romania;* ^*2*^ *Carol Davila University of Medicine and Pharmacy, Bucharest, Romania*

## EPV‐1380

### CGRP‐based treatment approaches for vestibular migraine: Assessing therapeutic outcomes

#### V. Pawar

##### Tarabichi Vertigo and Balance Center, Dubai, UAE

## EPV‐1381

### Clinic‐ophthalmological characteristics of migraine with aura

#### S. Nazarkulova ^1^; L. Khamraeva^1^; U. Nabiev^2^

##### ^*1*^ *Department of Ophthalmology, pediatric ophthalmology, Tashkent State Medical University, Tashkent, Uzbekistan;* ^*2*^ *Department of Neurology and Medical Psychology, Tashkent State Medical University, Tashkent, Uzbekistan*

## EPV‐1382

### Diagnostic value of bedside eye movement examination in movement disorders

#### E. Bittencurt Thomaz de Assis ^1^; P. Martínez Ruiz^2^; C. Acosta Gudiel^3^; H. Özdemir^4^; D. Kaski^5^

##### ^*1*^ *Immanuel Kant Baltic Federal University, Russia;* ^*2*^ *University of Sevilla, Seville, Spain;* ^*3*^ *The Autonomous University of Madrid, Madrid, Spain;* ^*4*^ *Ege University, Bornova, Türkiye;* ^*5*^ *University College London, London, UK*

## EPV‐1383

### Does lumbar puncture cause changes in magnetic resonance imaging findings in patients diagnosed with pseudotumour cerebri?

#### N. Kale; E. Alper Peksöz; S. Omerhoca; S. Erdogan

##### Health Sciences University, Bagcılar Training and Research Hospital, Department of Neurology

## EPV‐1384

### Dorsal midbrain syndrome due to complicated posterior reversible encephalopathy syndrome

#### K. Choi ^1^; S. Choi^1^; J. Choi^2^; H. Kim^2^

##### ^*1*^ *Department of Neurology1, Pusan National University Hospital, Pusan National University School of Medicine, Biomedical Research Institute, Busan, Republic of Korea;* ^*2*^ *Department of Neurology, Pusan National University Yangsan Hospital, Pusan National University School of Medicine, Research Institute for Convergence of Biomedical Science and Technology, Yangsan, Republic of Korea*

## EPV‐1385

### Euthyroid thyroid eye disease with negative thyroid antibodies

#### D. Elitas Ozmutlu; P. Togay Işıkay

##### Department of Neurology, Ankara University School of Medicine, Ankara, Türkiye

## EPV‐1386

### Evaluation of ocular higher‐order aberrations in patients with migraine: A comparative study

#### R. Shukla ^1^; A. Mishra^2^; P. Garg^1^; A. Shukla^1^

##### ^*1*^ *Department of Ophthalmology, All India Institute of Medical Sciences, Raebareli, Uttar Pradesh, India;* ^*2*^ *Department of Neurology, All India Institute of Medical Sciences, Raebareli, Uttar Pradesh, India*

## EPV‐1387

### Features of vestibular disorders in patients with sensorineural hearing loss

#### K. Shylo ^1^; O. Dubenko^2^

##### ^*1*^ *Graduate Student of Department of Neurology and Child Neurology of Kharkiv National Medical University of the Ministry of Health of Ukraine, Kharkiv, Ukraine;* ^*2*^ *Doctor of Medical Sciences, Professor of Department of Neurology and Child Neurology of Kharkiv National Medical University of the Ministry of Health of Ukraine; 4, Nauky Ave., Kharkiv, Ukraine;* ^*6*^ *1022*

## EPV‐1388

### Global burden of hearing loss in people with otitis media: A systematic review and meta‐analysis

#### S. Wasti^1^; M. Khan^1^; M. Muddassar ^1^; A. Wasti^2^; M. Azfar^1^; F. Awan^1^

##### ^*1*^ *Department of Neurology, King Edward Medical University, Lahore, Pakistan;* ^*2*^ *Department of Neurology, Continental Medical College, Lahore, Pakistan*

## EPV‐1389

### Identification of blood‐based inflammatory biomarkers for vestibular neuritis using a proximity extension assay

#### J. Choi ^1^; H. Kim^1^; S. Choi^1^; Y. Lee^1^; S. Choi^2^; K. Choi^2^; S. Lee^3^; M. Kim^2^

##### ^*1*^ *Department of Neurology, Pusan National University School of Medicine, Research Institute for Convergence of Biomedical Science and Technology, Pusan National University Yangsan Hospital, Yangsan, Republic of Korea;* ^*2*^ *Department of Neurology, Pusan National University Hospital, Pusan National University School of Medicine and Biomedical Research Institute, Busan, Republic of Korea;* ^*3*^ *Department of Neurology, Chonnam National University Hospital, Chonnam National University Medical School, Gwangju, Republic of Korea*

## EPV‐1390

### Intellectual disability in episodic ataxia type 2

#### H. Kim ^1^; S. Kim^2^; S. Lee^3^; J. Kim^3^; S. Na^4^; J. Choi^5^; J. Kim^6^

##### ^*1*^ *Biomedical Research Institute, Seoul National University Bundang Hospital, Seongnam, Republic of Korea;* ^*2*^ *Department of Neurology, Inha University Hospital, Inha University College of Medicine, Incheon, Republic of Korea;* ^*3*^ *Department of Neurology, Chonnam National University Medical School, Gwangju, Republic of Korea;* ^*4*^ *Department of Neurology, Incheon St. Mary's Hospital, the Catholic University of Korea, Incheon, Republic of Korea;* ^*5*^ *Department of Neurology, Pusan National University Yangsan Hospital, Yangsan, Republic of Korea;* ^*6*^ *Department of Neurology, College of Medicine, Seoul National University, Seoul, Republic of Korea*

## EPV‐1391

### Is there any prognostic factor for benign intracranial hypertension: Clinical, MRI, and OCT findings

#### I. Cetindamar ^1^; M. Altinbas^2^; E. Toplutas^1^; O. Arici Duz^1^; F. Senturk^2^

##### ^*1*^ *Department of Neurology, Istanbul Medipol University, Istanbul, Türkiye;* ^*2*^ *Department of Ophthalmology, Istanbul Medipol University, Istanbul, Türkiye*

## EPV‐1392

### Marked improvement of infantile nystagmus with memantine: A video‐documented case

#### F. Bingöl

##### *Düzce Atatürk Devlet Hastanesi*, *Düzce; Türkiye*

## EPV‐1393

### Motorist's vestibular disorientation syndrome: Clinical profile of 24 patients with proposed diagnostic criteria

#### V. Pawar

##### Tarabichi Vertigo and Balance Center, Dubai, UAE

## EPV‐1394

### Multimodal assessment of visual pathway involvement in multiple sclerosis without optic neuritis: correlating OCT metrics with neurological parameters

#### A. Khozyaeva^1^; N. Semenova^1^; E. Poneveshskaya ^2^; V. Gosteva^2^; A. Kukushkina^2^; M. Davydovskaya^2^; E. Lysogorskaya^2^

##### ^*1*^ *Lomonosov Moscow State University, Moscow, Russian Federation;* ^*2*^ *Zhadkevich Moscow City Clinical Hospital, Moscow, Russian Federation*

## EPV‐1395

### Ophthalmological predictors of idiopathic intracranial hypertension in patients with chronic migraine

#### A. Kramarenko; I. Maryenko

##### Republican Research and Clinical Center of Neurology and Neurosurgery, Minsk, Belarus

## EPV‐1396

### Orbital myositis as a rare manifestation of varicella zoster virus reactivation: A case report

#### A. Sánchez Rodríguez; A. García Rúa; M. Álvarez Álvarez; M. Martinez Palicio; A. Orejón Sánchez

##### Department of Neurology, Cabueñes University Hospital, Gijón, Spain

## EPV‐1397

### Possibilities for improving postural control in a virtual reality environment in patients with multiple sclerosis

#### I. Maryenko; M. Mozheiko; H. Kleban

##### State Institution “Republican Scientific and Practical Centre of Neurology and Neurosurgery”, Minsk, Belarus

## EPV‐1398

### Posterior Ischemic Optic Neuropathy After Herpes Zoster Opthalmicus

#### F. Uysal Tan ^1^; D. Erimhan Cevik^1^; S. Uke Uzun^2^; E. Erol^1^

##### ^*1*^ *Etjik City Hospital, Neurology, Ankara, Türkiye;* ^*2*^ *Etjik City Hospital, Ophthalmology, Ankara, Türkiye*

## EPV‐1399

### Predictors of long‐term disability in multiple sclerosis following optic neuritis

#### N. Lakri

##### Mohamed Seghir Nekache Hospital, Algiers, Algeria

## EPV‐1400

### Quality of life in visual snow syndrome

#### E. Sokolov ^1^; D. Romanov^1^; N. Vaschenko^2^; A. Sergeev^1^

##### ^*1*^ *I.M. Sechenov First Moscow State Medical University of the Ministry of Health of the Russian Federation (Sechenov University), Moscow, Russian Federation;* ^*2*^ *University Headache Clinic, Moscow, Russian Federation*

## EPV‐1401

### Quantitative analysis of smooth pursuit and saccadic eye movements in multiple sclerosis

#### P. Skacik; E. Kantorova; E. Kurca

##### Neurology Clinic UNM and JLF UK Martin, Slovakia

## EPV‐1402

### Safety and efficacy of high‐dose tradipitant for motion sickness: A systematic review and meta‐analysis of randomised controlled trials

#### M. Haq ^1^; M. Shahid^2^

##### ^*1*^ *Allama Iqbal Medical College, Lahore, Pakistan;* ^*2*^ *King Edward Medical University, Lahore, Pakistan*

## EPV‐1403

### The relationship between binaural auditory integration at the brainstem level and cognition in Parkinson's disease

#### A. Chumak ^1^; O. Alenikova^1^; M. Dymkovskaya^1^; A. Mikitchuk^2^

##### ^*1*^ *Neurology Department, Republican Research and Clinical Center of Neurology and Neurosurgery, Minsk, Belarus;* ^*2*^ *Department of Quantum Radiophysics and Optoelectronics Belarusian State University, Minsk, Belarus*

## EPV‐1404

### Two cases of micropsia after an ischemic stroke

#### K. Kang

##### Department of Neurology, Nowon Eulji Medical Center, Eulji University School of Medicine, Seoul, Republic of Korea

## EPV‐1405

### Validation study of oculometric biomarkers in parkinson's disease

#### I. Patry ^1^; B. Dalhøi^1^; S. Larsen^2^; E. Kerty^3^; S. Dalbro^3^

##### ^*1*^ *Neurology, Bulbitech AS, Trondheim, Noway;* ^*2*^ *Statistics, Meddoc AS, Skjetten, Norway;* ^*3*^ *Neurology, Oslo University Hospital, Oslo, Norway*

## EPV‐1406

### Velocity‐dependent horizontal optokinetic nystagmus dysfunction in Multiple Sclerosis: A possible oculomotor distinctive feature

#### M. Castela Ferreira; J. Lourenço Rosa; M. Silva

##### Neurology Department, Unidade Local de Saúde São José, Lisbon, Portugal

## EPV‐1407

### Vestibular Function Tests in patients with Spinocerebellar Ataxia Type 3 (SCA3) and Cerebellar Ataxia Neuropathy Vestibular Areflexia (CANVAS)

#### Z. Calic ^1^; A. Dayball^2^; D. Herrero^3^; N. Young^2^; S. Rosengren^2^; A. Bradshaw^2^; K. Kumar^4^; M. Welgampola^2^

##### ^*1*^ *Department of Neurophysiology, Liverpool Hospital, Australia;* ^*2*^ *Institute of Clinical Neurosciences, Royal Prince Alfred Hospital, Camperdown, Australia;* ^*3*^ *Faculty of Medicine of Clinica Alemana, Universidad del Desarrollo, Santiago de Chile, Chile;* ^*4*^ *Concord Repatriation General Hospital, Department of Neurology, Concord, Australia*

## EPV‐1408

### Visualization aspects of endolymphatic hydrops in patients with recurrent cochleovestibular disorders

#### I. Maryenko ^1^; A. Poddubny^2^

##### ^*1*^ *Republican Research and Clinical Center of Neurology and Neurosurgery, Minsk, Belarus;* ^*2*^ *Republican Center for Research and Practice in Otolaryngology, Minsk, Belarus*

## Neuropathies

## EPV‐1409

### A rare mimicker of CIDP: A case of POEMS syndrome presenting with progressive polyneuropathy

#### I. Karabas; C. Koseoglu Toksoy; I. Tasdelen; B. Akbagra Kaya

##### Department of Neurology, University of Health Sciences, Erenkoy Training and Research Hospital for Psychiatric and Neurological Diseases, Istanbul, Türkiye

## EPV‐1410

### Acute demyelinating polyneuropathy associated with diabetic amyotrophy: a complex clinical presentation in an elderly patient

#### B. Quintana López; D. Alonso Vallin; A. O. Sanchez; V. García Gracia; P. Diaz Aguirre; C. Castaño Ron; J. González Fernández

##### Neurology, University Hospital of Cabueñes, Gijón, Spain

## EPV‐1411

### Associations between short‐chain fatty acid profiles and sensory diabetic polyneuropathy in patients with type 2 diabetes and NAFLD: A pilot study

#### T. Atoev ^1^; Y. Khalimov^2^; G. Shodikulova^1^; G. Rodionov^3^; A. Bystrova^2^; V. Kuzmina^2^; G. Negmatova^1^

##### ^*1*^ *Samarkand State Medical University*, *Samarkand, Uzbekistan*; ^*2*^ *First Saint Petersburg State Medical University named after I.P.Pavlov*, *Saint Petersburg, Russia*; ^*3*^ *All‐Russian Centre for Emergency and Radiation Medicine named after A.M. Nikiforov*, *Saint Petersburg, Russia*

## EPV‐1412

### Burden of illness for patients with multifocal motor neuropathy: A quantitative survey of individuals from the United States

#### K. Gable^1^; K. Beadon^2^; S. Barrera‐Sierra^3^; C. Arvin‐Berod^3^; L. Jarman^3^; P. Nisbet^4^; L. Butler^5^; J. Wood ^3^

##### ^*1*^ *Duke University Medical Center, Durham, USA;* ^*2*^ *University of British Columbia, Vancouver, Canada;* ^*3*^ *argenx, Ghent, Belgium;* ^*4*^ *One Research LLC, Mt. Pleasant, USA;* ^*5*^ *GBS/CIDP Foundation International, Conshohocken, USA*

## EPV‐1413

### Clinical and electrophysiological correlates of Guillain–Barré syndrome in a tertiary care cohort

#### H. Jatic; N. Mahmutbegovic; N. Masic; E. Hasanbegovic; S. Drnda; S. Sabanagic‐Hajric; E. Mehmedika‐Suljic

##### Neurology Clinic, Clinical Center of Sarajevo University, Sarajevo, Bosnia and Herzegovina

## EPV‐1414

### Clinical characteristics and determinants of cognitive decline in patients with Parkinson's disease

#### W. Yiwen

##### Department of Neurology of Jiangbei Campus, The First Affiliated Hospital of Army Medical University, Chongqing, China

## EPV‐1415

### Clinical spectrum and treatment outcomes in Anti‐MAG neuropathy: A real‐world case series

#### A. Daponte ^1^; D. Tzavella^1^; M. Brinia^1^; E. Koropouli^1^; V. Gkotzamanis^1^; E. Koumasopoulos^1^; G. Zorbas^1^; G. Armenis^1^; E. Anagnostou^1^; P. Kokotis^1^; T. Zambelis^2^; V. Zouvelou^1^; M. Rentzos^1^

##### ^*1*^ *1st Department of Neurology, Eginitio Hospital, Medical School, National and Kapodistrian University of Athens, Athens, Greece;* ^*2*^ *Henry Dunant Hospital, Athens, Greece*

## EPV‐1416

### Cognitive dysfunction and peripheral neuropathy in a conflict‐affected diabetic population: A cross‐sectional study from Sudan

#### S. Mokhtar ^1^; I. Hussein^1^; H. Fadhl^2^; Y. Fadlelmoula^3^; I. Eltaeib^1^; M. Abdulrahman^4^

##### ^*1*^ *University of Gadarif, Gadarif, Sudan;* ^*2*^ *Aden University, Aden, Yemen;* ^*3*^ *Gazira University, Wad Madani, Sudan;* ^*4*^ *Alzaiem Alazhari University, Khartoum, Sudan*

## EPV‐1417

### Differentiating multifocal motor neuropathy and multifocal variant of CIDP: A multiparametric diagnostic approach

#### K. Stepanchenko ^1^; I. Pitenko^2^

##### ^*1*^ *Kharkiv National Medical University, Kharkiv, Ukraine*; ^*2*^ *Kharkiv City Clinical Multidisciplinary Hospital № 25*, *Kharkiv, Ukraine*

## EPV‐1418

### Eosinophilic granulomatosis with polyangiitis presenting as acute polyneuropathy mimicking Guillain‐Barre syndrome

#### R. Guizani; S. Naija; K. Jemai; A. Hassine; S. Ben Amor

##### Department of Neurology, Sahloul University Hospital, Sousse, Tunisia

## EPV‐1419

### Evolution of genetic testing in hereditary neuropathies: Experience of a centre

#### S. Palma ^1^; J. da Silva Cardoso^2^; P. Rayko^2^; D. Pinto^3^; C. Ionel^1^; T. Geraldes^1^; F. Antunes^4^; J. Monteiro^5^; M. Rodrigues^1^; C. Santos Silva^1,6^; J. Oliveira^3^; P. Pereira^1^

##### ^*1*^ *Department of Neurology, Unidade Local de Saúde Almada‐Seixal, Almada, Portugal;* ^*2*^ *Department of Genetics, Unidade Local de Saúde de São João, Porto, Portugal;* ^*3*^ *Centre for Predictive and Preventive Genetics, Institute for Molecular and Cell Biology, Porto, Portugal;* ^*4*^ *Department of Neurology, Unidade Local de Saúde de São José, Lisbon, Portugal;* ^*5*^ *Department of Paediatric Neurology, Unidade Local de Saúde Almada‐Seixal, Almada, Portugal;* ^*6*^ *Faculty of Medicine, Institute of Molecular Medicine, University of Lisbon, Lisbon, Portugal*

## EPV‐1420

### Genetic and neurophysiological insights into PIGG‐associated disorders

#### A. Vlasenko; D. Subbotin; A. Marakhonov; A. Murtazina

##### Research Centre for Medical Genetics, Moscow, Russian Federation

## EPV‐1421

### Guillain‐Barré syndrome in complex clinical settings — Diagnostic and therapeutic importance of nerve conduction studies

#### D. Sharma Bhandari

##### Department of Neurophysiology Sir Ganga Ram Hospital, New Delhi, India

## EPV‐1422

### Interactions of cerebrospinal fluid protein level in chronic inflammatory demyelinating neuropathy types

#### R. Gapeshin; D. Rudenko

##### Department of Neurology and Manual Medicine, Pavlov First Saint‐Petersburg State Medical University, Saint‐Petersburg, Russian Federation

## EPV‐1423

### Intravenous immunoglobulin to subcutaneous efgartigimod PH20 transition in CIDP: A phase 4 study in progress

#### Y. M. Hussain ^1^; A. Lerman^2^; A. Remmerie^3^; A. De Roeck^3^; K. Anokhina^3^; A. Gafson^3^; A. Seth^4^

##### ^*1*^ *Austin Neuromuscular Center, Austin, TX, USA;* ^*2*^ *Grove Neurology, Miami, FL, USA;* ^*3*^ *argenx, Ghent, Belgium;* ^*4*^ *Northwestern Medicine, Chicago, IL, USA*

## EPV‐1424

### Late‐onset, autosomal dominant, axonal, sensorimotor neuropathy due to the new variant c.1A_G in MPZ

#### F. Scorza^1^; J. Finsterer ^2^

##### ^*1*^ *Federal University of Sao Paulo, Brazil;* ^*2*^ *Neurology and Neurophysiology Center Vienna, Austria*

## EPV‐1425

### Mass balance and metabolism of BIA 28‐6156, an allosteric activator of beta‐glucocerebrosidase, in humans

#### A. Loureiro

##### Research Department, BIAL‐Portela & Cª, S.A., Coronado (S. Mamede e S. Romão), Portugal

## EPV‐1426

### Misdiagnosis of chronic inflammatory demyelinating polyneuropathy: Frequency, causes, and mimics

#### K. Stepanchenko ^1^; I. Pitenko^2^

##### ^*1*^ *Kharkiv National Medical University, Kharkiv; Ukraine*; ^*2*^ *Kharkiv City Clinical Multidisciplinary Hospital № 25, Kharkiv, Ukraine*

## EPV‐1427

### Morvan'syndrome: A systematic review

#### E. Pacucci; M. Guglielmi; V. Delmonte; E. D'Errico; D. Paolicelli

##### University of Bari “Aldo Moro”, Italy

## EPV‐1428

### Multiple cranial neuropathies as the presenting form of a parotid neoplasm

#### E. Alves; C. Santos

##### Neurology Department, Local Health Unit of Entre Douro e Vouga, Aveiro, Portugal

## EPV‐1429

### Neurological disturbances according to chronic inflammatory demyelinating neuropathy types

#### R. Gapeshin; D. Rudenko

##### Department of Neurology and Manual Medicine, Pavlov First Saint‐Petersburg State Medical University, Saint‐Petersburg, Russian Federation

## EPV‐1430

### Prognostic factors for unfavorable long‐term outcomes in chronic inflammatory demyelinating polyneuropathy

#### K. Stepanchenko ^1^; I. Pitenko^2^

##### ^*1*^ *Kharkiv National Medical University;* ^*2*^ *Kharkiv City Clinical Multidisciplinary Hospital № 25*

## EPV‐1431

### Relapsing focal chronic inflammatory demyelinating polyneuropathy – An uncommon presentation

#### J. Santos ^1^; D. Tribovane^2^; C. Alves^2^; C. Santos Silva^2^; P. Pereira^2^

##### ^*1*^ *ULS Estuário do Tejo, EPE;* ^*2*^ *Unidade Local de Saúde Almada‐Seixal, Almada,* Portugal

## EPV‐1432

### Riliprubart Phase 2 trial results: Efficacy analysis using immune neuropathy‐no evidence of disease activity framework

#### J. Allen^1^; C. Sommer^2^; M. Nunez ^3^; K. Lynch^3^; Y. Lu^4^; L. Xiong^3^; M. Alonso‐Alonso^3^; A. Paker^3^; L. Querol^5^

##### ^*1*^ *Department of Neurology, Division of Neuromuscular Medicine, University of Minnesota, Minneapolis, Minnesota, USA;* ^*2*^ *Neurologische Klinik und Poliklinik, Universitätsklinikum Würzburg, Würzburg, Germany;* ^*3*^ *Sanofi, Cambridge, MA, USA;* ^*4*^ *Sanofi R&D, Evidence Generation and Decision Science, Cambridge, MA, USA;* ^*5*^ *Neuromuscular Diseases Unit, Department of Neurology, Hospital de la Santa Creu i Sant Pau (IR SANT PAU), Barcelona, Spain; Centro para la Investigación Biomédica en Red en Enfermedades Raras (CIBERER), Madrid, Spain*

## EPV‐1433

### Sensory ganglionopathy: The value of integrated clinical and neurophysiological criteria

#### B. del Moral Sahuquillo; D. Arcila Salazar; J. Cajape Mosquera; Á. Antón Conejos

##### Neurology, HCU Lozano Blesa, Zaragoza, Spain

## EPV‐1434

### Sensory neuronopathies: Etiological review in our cohort

#### M. Callejo; A. Jauregui; A. Gonzalez; M. Martinez; A. Lopez; N. Marcos; F. Angoso; L. Pastor; J. Mallada; A. Rodriguez‐Antigüedad

##### Neurology, Cruces University Hospital, Barakaldo, Spain

## EPV‐1435

### Severe sensorimotor axonal polyneuropathy following enfortumab vedotin: Report of two cases from Türkiye

#### M. Balci; A. Yildirim

##### Neurology, İstanbul Medeniyet University, Göztepe City Hospital, İstanbul, Türkiye

## EPV‐1436

### Small fiber neuropathy and autonomic dysfunction in Sjogren's syndrome

#### E. Pal; R. Hejas; D. Varga; A. Sipos; M. Rohonczi

##### University of Pecs, Department of Neurology, Pecs, Hungary

## EPV‐1437

### Structural peripheral nerve changes in systemic lupus erythematosus: A high‐resolution ultrasound study

#### G. Di Pietro ^1^; P. Falco^1^; E. Evangelisti^1^; E. Galosi^1^; D. Iaria^2^; F. Ceccarelli^2^; F. Conti^2^; A. Truini^1^

##### ^*1*^ *Department of Human Neuroscience, Sapienza University, Rome, Italy;* ^*2*^ *Dipartimento di Scienze Cliniche, Internistiche, Anestesiologiche e Cardiovascolari, Lupus Clinic, Rheumatology, Sapienza Università di Roma, Rome, Italy*

## EPV‐1438

### Surgical versus conservative treatment of patients with ulnar neuropathy at the elbow: A randomized clinical trial

#### G. Omejec; S. Podnar

##### Institute of Clinical Neurophysiology, Division of Neurology, University Medical Centre Ljubljana, Slovenia

## Neurorehabilitation

## EPV‐1439

### A community‐based dyadic music therapy social prescribing program for people with dementia: A 12‐week feasibility study in Taiwan

#### L. Huang ^3^; H. Tseng^2^; W. Lin^1^

##### ^*1*^ *Ph.D. Program in Medical Neuroscience, College of Medical Science and Technology, Taipei Medical University, New Taipei City, Taiwan;* ^*2*^ *Professional Member, Music Therapy Association of Taiwan (MTAT), Taiwan;* ^*3*^ *Department of Neurology, Shuang Ho Hospital, Taipei Medical University, New Taipei City, Taiwan*

## EPV‐1440

### A goal‐directed framework for the dopaminergic treatment of apathy and hemispatial neglect following brain injury

#### S. Ilves^1^; D. Aries^1^; A. Swan^1^; E. Fletcher^1^; R. Shafiq^1^; C. Shah^1^; J. Twelftree^1^; J. Buttell^1^; C. Erwee^1^; A. Henson^1^; L. Rendell^1^; A. Joseph^2^; C. Julien^1^; N. Gorgoraptis ^2^

##### ^*1*^ *Regional Neurological Rehabilitation Unit (RNRU), Homerton University Hospital, London, UK;* ^*2*^ *Department of Neurology, Barts Health NHS Trust, London, UK*

## EPV‐1441

### Association between cerebral venous hemodynamic status and three‐month functional recovery in atherosclerotic ischemic stroke

#### F. Mallaev; G. Rakhimboyeva

##### Tashkent State Medical University, Tashkent, Uzbekistan

## EPV‐1442

### Barriers to rehabilitation in patients with mild neurological deficits after elective supratentorial neurosurgery

#### T. Studeniak; A. Minayeva; V. Tsola; M. Pryima; M. Cheipesh; J. Derkach

##### Department of Neurological Diseases, Psychiatry, Neurorehabilitation and Neurophysiology, Uzhhorod National University, Uzhhorod, Ukraine

## EPV‐1443

### Challenges during the rehabilitation for a patient with late‐onset Anti‐Jo 1 positive Antisynthetase syndrome – Case report

#### W. Qu

##### Cairns Hospital, Cairns North, Qld, Australia

## EPV‐1444

### Clinical application of machine learning empowered brain computer interfaces in amyotrophic lateral sclerosis – A systematic review and meta‐analysis

#### A. Gupta ^1^; K. Prasad^1^; E. Gupta^1^; S. Singh^2^; M. Garg^3^; J. George^4^

##### ^*1*^ *Bangalore Medical College and Research Institute, Bangalore, India;* ^*2*^ *Sri Guru Ramdas Institute of Medical Sciences and Research, Sri Amritsar, India;* ^*3*^ *Government Medical College, Amritsar, India;* ^*4*^ *K A P Viswanatham Government Medical College, Tamil Nadu, India*

## EPV‐1445

### Clinical autonomic screening in neurorehabilitation: Associations between objective orthostatic findings and patient‐reported symptoms

#### M. Cebuc ^1^; E. Postovoi^1^; A. Feodorovici^1^; G. Corcea^1^; S. Odobescu^1^; M. Sangheli^2^; O. Grosu^1^; L. Rotaru^1^

##### ^*1*^ *Diomid Gherman Institute of Neurology and Neurosurgery, Chișinău, Republic of Moldova;* ^*2*^ *Nicolae Testemițanu, State University of Medicine and Pharmacy, Chișinău, Republic of Moldova*

## EPV‐1446

### Combined land‐based and aquatic physiotherapy in Parkinson's disease and rheumatoid arthritis: A case study

#### I. Baleia; F. Nunes; R. Brandão

##### Alcoitão School of Health Sciences, Alcabideche, Portugal

## EPV‐1447

### Devices and outcomes in lower‐limb exoskeleton rehab after stroke and spinal cord injury (SCI): evidence map and trial landscape

#### Y. Vynohradova

##### University of Montpellier, LAS2 (MIASHS), Minor in Health Studies, Montpellier, France

## EPV‐1448

### Effectiveness and perspectives on the application of traditional Korean and Western medicine in the treatment of lumbosacral dorsopathy

#### Y. Isamuxametova

##### Department of Medical Rehabilitation, Sport Medicine, Folk Medicine and Physical Education, Tashkent State Medical University, Tashkent, Uzbekistan

## EPV‐1449

### Effectiveness of psychomotor therapy in post‐stroke rehabilitation in the south aral sea region

#### O. Qadamova ^1^; B. Ibadullayev^2^; D. Kamilova^3^; S. Dushamov^4^; I. Abdullayev^5^

##### ^*1*^ *Urgench State Medical Institute, Urgench, Uzbekistan;* ^*2*^ *Urgench State Medical Institute, Urgench, Uzbekistan;* ^*3*^ *Urgench State Medical Institute, Urgench, Uzbekistan;* ^*4*^ *Urgench State Medical Institute, Urgench, Uzbekistan;* ^*5*^ *Urgench State Medical Institute, Urgench, Uzbekistan*

## EPV‐1450

### Effectiveness of screening tools for assessing depressive risk in patients with ischemic stroke

#### R. Stoyanov ^1^; M. Dimitrova^1^; D. Dilkov^2^

##### ^*1*^ *University Multidisciplinary Hospital for Active Treatment and Emergency Medicine “N. I. Pirogov”, Sofia;* ^*2*^ *Psychiatry Clinic, Military Medical Academy, Sofia*

## EPV‐1451

### Effectiveness of telemedicine‐based cognitive rehabilitation in patients with chronic cerebral ischemia stages I–II

#### A. Ernazarov ^1^; A. Radjapov^1^; K. Maksudova^1^

##### ^*1*^ *Neurology, Tashkent State Medical University, Tashkent, Uzbekistan*

## EPV‐1452

### Effectiveness of vocational rehabilitation on return to work after acquired brain injury: A systematic review and meta‐analysis

#### N. Wagdy ^1^; M. Ali^1^; S. Abu Ahmad^2^; E. Bahbah^3^

##### ^*1*^ *Faculty of Medicine, Al‐Azhar University, Damietta, Egypt;* ^*2*^ *Faculty of Medicine, Al‐Quds University, Jerusalem, Palestine;* ^*3*^ *Neurology Department, Faculty of Medicine, Al‐Azhar University, Damietta, Egypt*

## EPV‐1453

### Effects of a home‐based calligraphic exercise program on manual dexterity in Parkinson's disease: A randomized controlled exploratory trial

#### A. Mendez‐Guerrero ^1^; P. Martin‐Jimenez^1^; V. Blanco‐Palmero^1^; J. Lapeña‐Motilva^1^; N. Sanzo Esnaola^2^; A. Sanchez‐Ferro^1^; S. Llamas‐Velasco^1^

##### ^*1*^ *Neurology Department. Hospital 12 de Octubre, Madrid, Spain;* ^*2*^ *Neurology Department. Hospital de Bidasoa, Gipuzkoa, Spain*

## EPV‐1454

### Efficacy of High‐Intensity Laser Therapy (HILT) on hemiplegic shoulder pain in post‐stroke patients: A systematic review and meta‐analysis

#### H. Hassan

##### Faculty of Medicine, Dubai Medical University, Dubai, United Arab Emirates

## EPV‐1455

### Electrophoresis and magnetophoresis in treatment of neuronal retinal changes after non‐ischemic retinal vein occlusion

#### M. Al‐Zamil ^1^; E. Kabardina^2^

##### ^*1*^ *RUDN University;* ^*2*^ *Rostov Regional Clinical Hospital, Rostov‐on‐Don, Russian Federation*

## EPV‐1456

### Exoskeleton‐assisted hand training after stroke: Motor outcomes and patient‐reported experience in an ongoing randomized trial

#### I. Kaleva ^1^; S. Gigl^1^; N. Berberich^2^; J. Lee^2^; E. Grossmann^1^; D. Sander^3^; J. Nassour^2^; C. Gordon^2^; B. Schmitz‐Koep^1^; K. Koch^1^

##### ^*1*^ *Department of Diagnostic and Interventional Neuroradiology, TUM Klinikum Rechts der Isar, School of Medicine and Health, Technical University of Munich, Munich, Germany;* ^*2*^ *Institute for Cognitive Systems, School of Computation, Information and Technology, Technical University of Munich, Munich, Germany;* ^*3*^ *Department of Neurology, Benedictus Krankenhaus Tutzing GmbH & Co. KG, Akademisches Lehrkrankenhaus, Technische Universität of Munich, Tutzing, Germany*

## EPV‐1457

### Feasibility of an online fatigue self‐management programme for people with Parkinson's disease: A pilot RCT

#### S. Alaqeel ^1^; K. Deane^2^; J. Hibberd^2^

##### ^*1*^ *King Saud University, Saudi Arabia;* ^*2*^ *University of East Anglia, UK*

## EPV‐1458

### Immersive virtual reality for post‐stroke upper limb rehabilitation: A systematic review and meta‐analysis with trial sequential analysis

#### S. Qadri^1^; A. Harb ^2^; M. Katta^3^; S. Zahid^1^; T. Asghar^4^; A. Ahmad^5^; S. Qasim^6^; S. Jamil^1^

##### ^*1*^ *Dow University of Health Sciences, Karachi, Pakistan;* ^*2*^ *Faculty of Medicine, Al‐Azhar University, Cairo, Egypt;* ^*3*^ *University of La Verne, California, USA;* ^*4*^ *Akhtar Saeed Medical and Dental College, Lahore, Pakistan;* ^*5*^ *Pakistan institute of Medical Sciences, Federal Medical College, Islamabad, Pakistan;* ^*6*^ *Multan Medical and Dental College, Multan, Pakistan*

## EPV‐1459

### Intermittent Theta Burst Stimulation (iTBS) priming for post‐stroke upper limb motor recovery: A systematic review and meta‐analysis

#### M. A. Wafa ^1,3^; M. Elmekkawi^1^; A. Mohamed^1^; M. H. Khalifa^2^; M. Eltalkhawy^1^; Y. Mohsen^1,3^

##### ^*1*^ *Mansoura University Faculty of Medicine, Mansoura, Egypt;* ^*2*^ *Sinai University, Ismailia, Egypt;* ^*3*^ *Mansoura Manchester Research Society, Mansoura University Faculty of Medicine, Mansoura, Egypt*

## EPV‐1460

### Length of stay after stroke in hospitals and rehabilitation centers: A systematic review and meta‐analysis from Arab Countries and Saudi Arabia

#### A. Alhusayni; A. Alzahrani

##### Department of Health Rehabilitation, College of Applied Medical Sciences, Shaqra University, Shaqra, Riyadh Province, Saudi Arabia

## EPV‐1461

### Mesenchymal stem cells in the complex treatment of diffuse and focal brain lesions

#### L. Novikava ^1^; K. Basiakova^1^; L. Parkhach^1^; S. Pashkevich^2^; V. Hancharou^3^

##### ^*1*^ *1Republican Research and Clinical Center of Neurology and Neurosurgery, Minsk, Belarus;* ^*2*^ *Institute of Physiology of the National Academy of Sciences of Belarus, Minsk, Belarus;* ^*3*^ *Minsk City Emergency Hospital, Minsk, Belarus*

## EPV‐1462

### Perineuronal net‐mediated regulation of calcium homeostasis and oxidative stress in post‐stroke neuroplasticity

#### N. Aderinto^1^; I. Abraham^2^; J. Kwape^3^; A. Abdulkader^4^; A. Akinmeji^5^; M. Ajadi^2^; G. Uzoechina^6^; P. Ajala^7^; C. Agu^8^; D. Babalola^2^; A. Babalola^9^; G. Olatunji^10^; E. Kokori^11^; F. Aljamea ^12^

##### ^*1*^ *Department of Medicine and Surgery, LAUTECH, Ogbomosho, Nigeria;* ^*2*^ *Department of Medicine and Surgery, University of Ilorin, Ilorin, Nigeria;* ^*3*^ *University of Botswana, School of Medicine, Gaborone, Botswana;* ^*4*^ *College of Medicine, Almaarefa University, Riyadh, Saudi Arabia;* ^*5*^ *New York Medical College, New York, USA;* ^*6*^ *Faculty of Clinical Sciences, University of Nigeria Teaching Hospital, Enugu, Nigeria;* ^*7*^ *College of Medicine, University of Ibadan, Ibadan, Nigeria;* ^*8*^ *Enugu State University of Science and Technology, Enugu, Nigeria;* ^*9*^ *Kornberg School of Dentistry, Temple University, Philadelphia, USA;* ^*10*^ *Johns Hopkins Bloomberg School of Public Health, Baltimore, Maryland, USA;* ^*11*^ *Department of Internal Medicine, Cape Fear Valley Medical Center, Fayetteville, North Carolina, USA;* ^*12*^ *College of Medicine, Vision College, Riyadh, Saudi Arabia*

## EPV‐1463

### Post‐stroke home‐based rehabilitation: Functional impact and patient satisfaction

#### M. Martínez Palicio; D. Alonso Vallín; A. O Sanchez; M. Temprano Fernández; C. Antón Gonzalez; S. González González; I. Casado Menendez; A. García Rua

##### Hospital Universitario de Cabueñes, Gijón, Spain

## EPV‐1464

### ReFresh: A feasibility randomised trial of an online fatigue‐management programme for people with Parkinson's disease

#### S. Alageel ^1^; S. Alageel^2^; K. Deane^2^; J. Hibberd^2^

##### ^*1*^ *Occupational Therapy, College of Applied Medical Sciences, King Saud University, Riyadh, Saudi Arabia;* ^*2*^ *School of Health Sciences, University of East Anglia, Norwich, UK*

## EPV‐1465

### Regression of pain and paresthesia symptoms in patients with trigeminal neuralgia following TENS therapy

#### M. Al‐Zamil

##### *RUDN University*, *Moscow, Russian Federation*

## EPV‐1466

### Rehabilitation of patients after stroke through multifactorial analysis

#### Y. Madjidova; F. Rakhimov

##### Tashkent State Medical University, Tashkent, Uzbekistan

## EPV‐1467

### Technology readiness in neurological chronic patients in Denmark

#### J. Lykke ^1^; T. Thomsen^1^; C. Kruuse^1^; B. Sørensen^2^; C. Riberholt^1^

##### ^*1*^ *Department of Brain and Spinal Cord Injury/The Neuroscience Centre/Copenhagen University Hospital Rigshospitalet/Copenhagen/Denmark;* ^*2*^ *Movement Disorder and Pain Research Center (MPRC)/Copenhagen University Hospital Rigshospitalet/Copenhagen/Denmark*

## EPV‐1468

### Telehealth intervention using the HEARTS Technical Package and an activity monitor to increase physical activity post‐stroke: a feasibility study

#### P. da Cruz Peniche ^1^; J. de Paula Magalhães^1^; O. Lennon^2^; J. Melo dos Santos Ferreira^1^; J. Cunha Polese^1^; C. Danielli Coelho de Morais Faria^1^

##### ^*1*^ *Department of Physiotherapy, Universidade Federal de Minas Gerais, Belo Horizonte, Brazil;* ^*2*^ *School of Public Health, Physiotherapy and Sports Science, University College Dublin, Dublin, Ireland*

## EPV‐1469

### The effect of repeated use of BCI‐controlled hand exoskeleton in a patient with post‐stroke severe paresis

#### V. Lizhdvoy; A. Kondur; E. Slyunkova

##### Department of Neurology, 1Moscow Regional Research Clinical Institute n.a. M,F, Vladimirskiy, Moscow, Russian Federation

## EPV‐1470

### The effectiveness of transcranial magnetic stimulation in reducing neurological complications in children with hydrocephalus

#### M. Safarova

##### *Mediolife Private Clinic*, *Northampton, UK*

## EPV‐1471

### The experience of using psychomotor techniques in rehabilitation in psychiatry

#### T. Irmukhamedov; D. Sattarova

##### Kimyo International University in Tashkent, Tashkent, Uzbekistan

## EPV‐1472

### Treatment of post‐COVID symptoms with auricular vagus nerve stimulation

#### P. Gebetsroither ^1^; T. Ettenauer^2^; R. Likar^2^

##### ^*1*^ *Private Practice, Neuhofen, Austria;* ^*2*^ *Department for Anaesthesiology and Intensive Medicine, Klinikum Klagenfurt am Wörthersee, Austria*

## EPV‐1473

### Use of an artificial intelligence–based clinical assistant in cognitive rehabilitation of post‐stroke patients

#### A. Radjapov ^1^; A. Yernazarov^2^

##### ^*1*^ *Department of Neurology, Tashkent State Medical University, Tashkent, Uzbekistan;* ^*2*^ *Department of Neurology, Tashkent State Medical University, Tashkent, Uzbekistan*

## EPV‐1474

### Virtual reality rehabilitation in patients with total knee replacement: A study from Rural Iran

#### F. Yadolahi; A. Sharifi

##### Physiotherapy Department, School of Rehabilitation, Zahedan University of Medical Sciences, Zahedan, Iran

## EPV‐1475

### Vitamin D supplementation on cognitive and functional outcomes in patients with traumatic brain injury: A systematic review and meta‐analysis

#### T. Siriwardana ^1^; C. Pereira^1^; K. Lakmal^2^; D. Hettiarachichi^3^; L. Siriwardana^4^

##### ^*1*^ *Faculty of Medicine, University of Colombo, Sri Lanka;* ^*2*^ *Post Graduate Institute of Medicine, University of Colombo, Sri Lanka;* ^*3*^ *Department of Anatomy, Genetics and Biomedical Informatics Faculty of Medicine, University of Colombo, Sri Lanka;* ^*4*^ *Ministry of health, Sri Lanka*

## Neurosonology

## EPV‐1476

### Correlation between echo intensity and abnormal spontaneous activity in cubital tunnel syndrome: A preliminary study

#### J. Yoon; G. Yoon; J. Kim; E. Koh

##### Korea University Guro Hospital, Seoul, Republic of Korea

## EPV‐1477

### Cranial ultrasonography and circulatory markers of inflammation as predictors for atherosclerosis in patients with depression

#### M. Badr

##### Neurology Section, Neuropsychiatry Department, Tanta University, Tanta, Egypt

## EPV‐1478

### Impact of clinical characteristics and nomogram model in adult patients with ependymoma: A population‐based study

#### A. Almezaine

##### Tanta General Hospital, Tanta, Egypt

## EPV‐1479

### Pediatric stroke in arid regions shows dehydration‐related severity, with hemiparesis, seizures, and ischemic stroke predominating

#### S. Allamurodova

##### Surkhandarya Regional Multidisciplinary Children's Center, Uzbekistan

## EPV‐1480

### Pilot study: Differences on distance between median nerve and ulnar artery in various wrist motions

#### J. Yoon; E. Koh; J. Kim; G. Yoon

##### Korea University Guro Hospital, Seoul, Republic of Korea

## Neurotoxicology/occupational neurology

## EPV‐1481

### Comparative evaluation of neurological adverse effects of typical and atypical antipsychotics in patients with schizophrenia

#### M. Zokirov; L. Shadmanova; A. Tadjibaev

##### Tashkent State Medical University, Department of Psychiatry and Narcology, Tashkent, Uzbekistan

## EPV‐1482

### Environmental neurotoxicology in marginalized communities: A systematic review focusing on lead exposure among Roma children

#### S. Wasti^1^; A. Wasti^2^; M. Iqbal ^1^

##### ^*1*^ *Department of Neurology, King Edward Medical University, Lahore, Pakistan;* ^*2*^ *Department of Neurology, Continental Medical College, Lahore, Pakistan*

## EPV‐1483

### Marchiafava‐Bignami Disease: An Uncommon Corpus Callosum Disorder

#### F. Sánchez García; J. Villamor Rodríguez; M. González Gómez; E. Gismera Fontes; L. Cabral Esquea; M. Mas Serrano

##### Neurology Service, Guadalajara University Hospital, Guadalajara, Spain

## EPV‐1484

### Neurological adverse events of antidepressant medications: A pharmacovigilance study

#### O. Darras ^1^; Y. Zayed^2^

##### ^*1*^ *University of Jordan, Amman, Jordan;* ^*2*^ *Ministry of Health, Amman, Jordan*

## EPV‐1485

### Neurological effects of nitrous oxide: A case of combined spinal cord degeneration secondary to recreational use

#### M. Murga; R. Dodu; M. Hernández; G. Clementson; M. Castro

##### Hospital Regional Universitario de Málaga, Málaga, Spain

## EPV‐1486

### Subacute chorea following pesticide exposure: A case report

#### A. Çolakoğlu; N. Kahveci Erdemir; A. Aksoy Gündoğdu

##### Neurology, Tekirdag Namik Kemal University, Tekirdag, Türkiye

## EPV‐1487

### When mycophenolate strikes: Unveiling the neurotoxic danger of immunosuppressive therapy

#### C. Segura Sanz

##### Department of Neurology, Hospital Virgen de la Victoria, Málaga, Spain

## Neurotraumatology

## EPV‐1488

### A consolidation gate linking ketamines signaling to durable epigenetic remodeling in depression

#### A. Nepton; S. Faber; S. Perez‐Rosal; M. Williams

##### University of Ottawa, School of Psychology, Ottawa, Canada

## EPV‐1489

### Assessing the diagnostic requisites in paediatric Traumatic Brain Injury (TBI) using Artificial Intelligence (AI) – A meta‐analysis

#### K. Prasad ^1^; A. Gupta^1^; E. Gupta^1^; J. Jayavani Jayakaran^2^; A. Kaur^3^

##### ^*1*^ *Bangalore Medical College and Research Institute, Bangalore, India;* ^*2*^ *Stanley Medical College, Chennai, India;* ^*3*^ *Sri Guru Ram Das Institute of Medical Sciences and Research, Amritsar, India*

## EPV‐1490

### Mesenchymal stem cells in the treatment of traumatic brain injury

#### L. Novikava ^1^; K. Basiakova^1^; L. Parkhach^1^; S. Pashkevich^2^; V. Hancharou^3^

##### ^*1*^ *Republican Research and Clinical Center of Neurology and Neurosurgery, Minsk, Belarus;* ^*2*^ *Institute of Physiology of the National Academy of Sciences of Belarus, Minsk, Belarus;* ^*3*^ *Minsk City Emergency Hospital, Minsk, Belarus*

## EPV‐1491

### Severe traumatic brain injury in Brazil: 15 years of outcomes and regional disparities from DATASUS

#### D. Moreira do Amaral

##### São Francisco University, Bragança Paulista, Brazil

## EPV‐1492

### Smartphone quantitative pupillometry for traumatic brain injury: Systematic review of diagnostic performance and feasibility

#### T. Bin‐Islam

##### Graduate Entry Medicine, University of Nottingham, Nottingham, UK

## EPV‐1493

### The bone metabolism disorders in young men with epilepsy

#### A. Voitiuk ^1^; A. Voitiuk^2^

##### ^*1*^ *Department of Neurology with a course in Neurosurgery,Kharkiv National Medical University, Kharkiv, Ukraine;* ^*2*^ *Medical Centre “Neiron”*

## EPV‐1494

### Use of artificial intelligence in the prediction of traumatic brain injury outcomes in paediatric population: A systematic review and meta‐analysis

#### K. Prasad ^1^; A. Gupta^1^; E. Gupta^1^; N. Nayan^2^; D. Pai^3^

##### ^*1*^ *Bangalore Medical College and Research Institute, Bangalore, India;* ^*2*^ *Netaji Subhash Chandra Bose Subharti Medical College, Meerut, India;* ^*3*^ *K.V.G Medical College and Hospital, Sullia Dakshina Kannada, India*

## Pain

## EPV‐1495

### Acute myofascial facial pain management in dentistry patients

#### A. Kornilova ^1^; S. Tsucor^2^; L. Kornilova^3^

##### ^*1*^ *Dentistry Center “Dial‐Dent”, Moscow, Russian Federation;* ^*2*^ *Dentistry Center “Dial‐Dent”, Moscow, Russian Federation;* ^*3*^ *Vinogradov Clinical Hospital by “People”, Friendship University of Russia named after Patrice Lumumba, Moscow, Russian Federation*

## EPV‐1496

### Additional dopaminergic replacement for nociceptive pain in Parkinson's disease: A randomized double‐blind placebo‐controlled trial

#### M. Tohoru Nakamura; G. Taricani Kubota; D. Ciampi Araújo de Andrade; C. Listik; M. Jacobsen Teixeira; E. Reis Barbosa; R. Gisbert Cury

##### Hospital das Clínicas, Faculty of Medicine, University of São Paulo (HCFMUSP), Sao Paulo, Brazil

## EPV‐1497

### Association of allostatic load and diet quality with depression and pain comorbidity: NHANES 2009‐2010

#### H. Tang; J. Chen

##### Shanghai Mental Health Center, Shanghai, China

## EPV‐1498

### Botulinum toxin A in treatment of resistant neuropathic pain syndrome: Case series

#### M. Andreev; E. Badelina

##### Federal Medical‐Biological Agency Institute of Medical Rehabilitation and Curortology, Moscow, Russian Federation

## EPV‐1499

### Can repurposed ibudilast mitigate fibromyalgia pain?

#### T. Roesel

##### Clinical Trials, CERVOE.org, Bozman, Maryland, USA

## EPV‐1500

### Cannabinoids in chronic pain: Clinical outcomes, adverse effects and legal challenges

#### A. Sic ^1^; C. George^2^; D. Ferrer Gonzalez^1^; V. Tseriotis^3^; N. Knezevic^1^

##### ^*1*^ *Department of Anesthesiology, Advocate Illinois Masonic Medical Center, Chicago, USA;* ^*2*^ *Chicago Medical School, Rosalind Franklin University of Medicine and Science, North Chicago, USA;* ^*3*^ *Department of Neurology, Agios Pavlos General Hospital of Thessaloniki, Kalamaria, Thessaloniki, Greece*

## EPV‐1501

### Clinical effectiveness of gabapentin in trigeminal neuralgia

#### A. Iroda ^1^; M. Yoqutxon^1^; X. Dilbar^1^

##### ^*1*^ *Neurology, Tashkent State Medical University, Tashkent, Uzbekistan*

## EPV‐1502

### Complex use of manual and training therapy with audiovisual vibrotactile stimulation in Zero‐G for low back pain treatment in athletes

#### L. Anatskaia ^1^; V. Zabarovski^1^; T. Svinkovskaya^1^; T. Grigorovich^1^; C. Bulynko^2^; P. Bespalova^2^; E. Alekseeva^2^

##### ^*1*^ *Department of Neurology/Republican Research and Practical Center of Neurology and Neurosurgery, Minsk, Belarus;* ^*2*^ *Laboratory of Sports Psychology/Republican Research and Practical Sports Center, Minsk, Belarus*

## EPV‐1503

### Effectiveness and safety of oral herbal medicine for postherpetic neuralgia: A systematic review and meta‐analysis

#### S. Kim ^1^; A. Shim^2^; S. Lee^1^; S. Lee^1^

##### ^*1*^ *Department of Acupuncture and Moxibustion Medicine, Kyung Hee University Medical Center, Seoul, Republic of Korea;* ^*2*^ *College of Korean Medicine, Kyung Hee University, Seoul, Republic of Korea*

## EPV‐1504

### Efficacy and safety of adjunctive 10‐Hz rTMS in central post stroke pain: A randomized double‐blind placebo‐controlled trial

#### J. Kalita ^1^; N. Pandey^1^; P. Pandey^1^; F. Nizami^1^; R. Tandon^1^; R. Mahajan^1^; V. Singh^2^

##### ^*1*^ *Department of Neurology, Sanjay Gandhi Post Graduate Institute of Medical Sciences, Lucknow, Uttar Pradesh, India;* ^*2*^ *Department of Neurology, Sanjay Gandhi Post Graduate Institute of Medical Sciences, Lucknow, Uttar Pradesh, India*

## EPV‐1505

### Examining how non‐pharmacological interventions that affect sleep quality impact pain‐related neurotransmitters in patients with fibromyalgia

#### P. Teli

##### Warwick Medical School, University of Warwick, Coventry, UK

## EPV‐1506

### fMRI markers of pain catastrophizing and nocebo effects in patients with chronic neuropathic pain

#### O. Alenikova ^1^; M. Dymkovskaya^1^; L. Parchach^1^; E. Mikitchuk^2^; S. Tolkach^3^; A. Zelenko^3^

##### ^*1*^ *Republican Research and Clinical Center of Neurology and Neurosurgery*, *Minsk; Belarus*; ^*2*^ *Belarusian State University;* ^*3*^ *Republican Research and Practical Center for Epidemiology and Microbiology*, *Minsk, Belarus*

## EPV‐1507

### Hemiplegic shoulder pain

#### F. Colonna; M. Genesi

##### SC di Neuroriabilitazione e GCA, Ospedale Generale “Moriggia‐Pelascini”, Gravedona e Uniti, Como, Italy

## EPV‐1508

### Optimization of therapy in patients with trigeminal neuralgia

#### A. Iroda ^1^; M. Yoqutxon^1^; M. Hurshida^1^

##### ^*1*^ *Neurology, Tashkent State Medical University, Tashkent, Uzbekistan*

## EPV‐1509

### Pain in children with Duchenne muscular dystrophy: An underrecognized symptom

#### S. Lima ^1^; J. Cardoso^2^; M. Santos^3^; L. Morais^3^; R. Amorim^3^; V. Oliveira^4^; C. Garrido^3^

##### ^*1*^ *Neurology Department, Unidade Local de Saúde de Trás‐os‐Montes e Alto Douro, Vila Real, Portugal;* ^*2*^ *Pediatric Neurology Department, Centro Materno Infantil do Norte, Unidade Local de Saúde de Santo António, Porto, Portugal;* ^*3*^ *Multidisciplinary Pediatric Neuromuscular Diseases Team, Centro Materno Infantil do Norte, Unidade Local de Saúde de Santo António, European Reference Network for Rare Neuromuscular Diseases (EURO‐NMD) Center, Porto, Portugal;* ^*4*^ *Pediatric Pneumology Department, Centro Materno Infantil do Norte, Unidade Local de Saúde de Santo António, Porto, Portugal*

## EPV‐1510

### Pain management in the neurology clinics: Level of knowledge and assessment of the needs of patients suffering from pain among the EAN members

#### A. Szewczyk ^1^; S. Vigneri^2^; G. De Stefano^3^; O. Grosu^4^; M. Rakusa^5^; K. Rukavina^6^

##### ^*1*^ *Department of Neurology, Medical University of Lublin, Poland;* ^*2*^ *Casa Di Cura Santa Maria Maddalena, Neurology and Neurophysiology Service, Occhiobello, Italy;* ^*3*^ *Sapienza University, Department of Human Neuroscience, Rome, Italy;* ^*4*^ *Diomid Gherman Institute of Neurology and Neurosurgery, Headache Center, Chisinau, Republic of Moldova;* ^*5*^ *Division of Neurology, University Medical Centre Maribor, Maribor, Slovenia;* ^*6*^ *Movement Disorders Hospital Beelitz, Beelitz, Germany*

## EPV‐1511

### Paroxysmal extreme pain disorder: A winding road to a definitive diagnosis

#### M. Saini

##### *National Neuroscience Institute*, *Singapore*

## EPV‐1512

### Perception of pain and its management in people with multiple sclerosis

#### J. Chojdak‐Łukasiewicz ^1^; A. Kołtuniuk^2^; A. Greszta^3^; A. Pokryszko‐Dragan^1^

##### ^*1*^ *Clinical Department of Neurology, Wroclaw Medical University, Wroclaw, Poland;* ^*2*^ *Division of Anaesthetic and Surgical Nursing, Wroclaw Medical University, Wroclaw, Poland;* ^*3*^ *University Clinical Hospital, Wroclaw, Poland*

## EPV‐1513

### Real‐world outcomes of intranasal esketamine in treatment‐resistant major depression with chronic pain

#### S. Benavente López ^1^; A. Lara Fernández^2^; L. Jiménez Muñoz^3^; M. Volante Gómez^4^; C. Hernaiz Gordo^5^; J. García‐Carmona^6^

##### ^*1*^ *Psychiatrist, Head of the Department of Psychiatry, Infanta Elena University Hospital, Madrid, Spain;* ^*2*^ *Psychiatrist, Department of Psychiatry, Infanta Elena University Hospital, Madrid, Spain;* ^*3*^ *Psychology, Fundación Jiménez Díaz Health Research Institute (IIS‐FJD), Fundación Jiménez Díaz University Hospital, Madrid, Spain;* ^*4*^ *Clinical Psychology Resident, Department of Psychiatry, Infanta Elena University Hospital, Madrid, Spain;* ^*5*^ *Psychiatry Resident, Department of Psychiatry, Infanta Elena University Hospital, Madrid, Spain;* ^*6*^ *Neurologist, Department of Neurology, Santa Lucía General University Hospital, Murcia, Spain*

## EPV‐1514

### TENS in chronic pain management: An 11‐year nationwide analysis using the French national health data system

#### V. Arcani ^1^; A. Donnet^2^; S. Redon^2^

##### ^*1*^ *Aix‐Marseille Université, AP‐HM, Hôpital de La Timone, Service Pharmacie, Marseille, France;* ^*2*^ *Aix‐Marseille Université, AP‐HM, Hôpital de La Timone, Centre d'Evaluation et de Traitement de la Douleur, Marseille, France*

## EPV‐1515

### The impact of barometric pressure changes on migraine attacks severity, frequency, and duration: A systematic review of the literature

#### A. Farah ^1^; Y. Adam^1^; R. Ahmed^2^; A. Elamir^1^; O. Ahmed^1^; I. Abdelrahman^1^; A. Ahmed^1^; R. Amer^1^; M. Mohammad^1^; H. Elnosha^1^

##### ^*1*^ *School of Medicine, St George's University, St George, Grenada;* ^*2*^ *National Ribat University, Khartoum, Sudan*

## EPV‐1516

### Trigeminal autonomic cephalalgias: A single‐centre retrospective real‐world study from the Erebouni pain clinic

#### M. Manukyan; H. Sargsyan; N. Yeghiazaryan

##### Neurology Department and Pain Clinic, Erebouni Medical Center, Yerevan, Armenia

## Palliative care

## EPV‐1517

### A National neuropalliative care training programme: Experience from the Spanish society of neurology

#### Á. Lambea‐Gil ^1^; I. Zamarbide^2^; J. Lahuerta^3^; D. Ezpeleta^4^; G. Más‐Sésé^5^; F. Escamilla‐Sevilla^6^

##### ^*1*^ *Department of Neurology, Hospital de la Santa Creu i Sant Pau, Barcelona, Spain;* ^*2*^ *Department of Neurology, Fundación Jiménez Díaz, Madrid, Spain;* ^*3*^ *Committee of Humanisation and Palliative Care, Spanish Society of Neurology, Barcelona, Spain;* ^*4*^ *Vithas Neuroscience Institute, Madrid, Spain;* ^*5*^ *Hospital la Pedrera, Denia (Alicante), Spain;* ^*6*^ *Department of Neurology, Hospital Universitario Virgen de las Nieves, Granada, Spain*

## Peripheral nerve disorders

## EPV‐1518

### A single‐center cohort description of patients diagnosed with leprosy in a tertiary hospital in Catalonia

#### P. Carbonell‐Fernandez ^1^; M. Caballero‐Avila^1^; E. Roe^2^; M. Delgado‐Romeu^1^; O. Lopez‐Lombardia^1^; L. Querol^1^; T. Mederer‐Fernandez^1^; R. Collet‐Vidiella^1^; E. Pascual‐Goñi^1^

##### ^*1*^ *Neurology Department, Hospital de la Santa Creu i Sant Pau, Barcelona, Spain;* ^*2*^ *Dermatology Department, Hospital de la Santa Creu i Sant Pau, Barcelona, Spain*

## EPV‐1519

### Acute onset chronic inflammatory demyelinating polyneuropathy mimicking Guillain Barré syndrome: Clinical and electrophysiological insights

#### A. Alhajmabrouk; Y. Boubaker; R. Zouari; D. Ben Mohamed; A. Rachdi; Z. Saied; S. Ben Sassi

##### Mongi Ben Hamida National Institute of Neurology, Tunis, Tunisia

## EPV‐1520

### Atypical sensorimotor polyradiculoneuropathy: Have you considered POEMS syndrome?

#### D. Abanda; M. Rascarache; I. Kolev; C. Vaduva

##### Neurology, Centre Hospitalier de Guingamp, Guingamp, France

## EPV‐1521

### Case report: A diagnostic Odyssey leading to neurosyphilis

#### N. Lambeng^1^; L. Yamashita ^2^; L. Fontao^3^; C. Deffert^1^; A. Lascano^4^; L. Toutous Trellu^3^; M. Uginet^4^; P. Lalive^4^; G. Breville^4^

##### ^*1*^ *Division of Laboratory Medicine, University Hospital of Geneva, Geneve, Switzerland;* ^*2*^ *Center for Neuroinflammation and Experimental Therapeutics, Department of Neurology, The University of Pennsylvania, Philadelphia, USA;* ^*3*^ *Division of Dermatology and Venereology, University Hospital of Geneva, Geneve, Switzerland;* ^*4*^ *Division of Neurology, University Hospital of Geneva, Geneve, Switzerland*

## EPV‐1522

### Characterization of neuropathies associated with IgM monoclonal gammopathy without anti‐MAG antibodies

#### S. Bernardo ^1^; M. Henriques^1^; C. Guerreiro^2^; B. Madureira^2^; M. Brum^2^; L. Medeiros^2^; I. Margarido^3^; M. Pinto^3^; L. Braz^3^; R. Rodrigues^4^; C. Campos^4^; L. Fagundes^5^; J. Alves^5^; L. Almendra^5^; C. Vaz^6^; E. Santos^6^; S. Palma^7^; P. Pereira^7^; M. Mendes^8^; A. Veiga^8^; A. Cabral^9^; H. Costa^9^; G. Soares^10^; S. Moreira^10^; E. Alves^11^; C. Santos^11^; V. Fonseca^12^; J. Castelo^12^; S. Pinto^13^; G. Bonifácio^13^; S. Cruz^1^

##### ^*1*^ *Department of Neurology, ULS Amadora‐Sintra, Portugal;* ^*2*^ *Department of Neurology, ULS São José;* ^*3*^ *Department of Neurology, ULS São João, Portugal;* ^*4*^ *Department of Neurology, ULS Santa Maria, Portugal;* ^*5*^ *Department of Neurology, ULS Coimbra, Portugal;* ^*6*^ *Department of Neurology, ULS Santo António, Portugal;* ^*7*^ *Department of Neurology, ULS Almada/Seixal, Portugal;* ^*8*^ *Department of Neurology, ULS Trás‐os‐Montes, Portugal;* ^*9*^ *Department of Neurology, ULS Gaia/Espinho, Portugal;* ^*10*^ *Department of Neurology, ULS Matosinhos, Portugal;* ^*11*^ *Department of Neurology, ULS Entre Douro e Vouga, Portugal;* ^*12*^ *Department of Neurology, ULS Loures/Odivelas, Portugal;* ^*13*^ *Department of Neurology, ULS Arrábida, Portugal*

## EPV‐1523

### Chronic inflammatory demyelinating polyneuropathy and thymoma

#### D. Mezdad; S. Maguenouche; T. Sari; S. Belarbi

##### Department of Neurology, Ali Ait Idir Hospital, Algiers, Algeria

## EPV‐1524

### Chronic inflammatory demyelinating polyneuropathy: Clinical spectrum and treatment outcomes in a Tunisian cohort

#### Y. Boubaker; A. Alhajmabrouk; R. Zouari; Y. Memmich; A. Rachdi; D. Ben Mohamed; Z. Saied; S. Ben Sassi

##### Mongi Ben Hamida National Institute of Neurology, Tunis, Tunisia

## EPV‐1525

### Clinical, electrophysiological profile and therapeutic outcome in acute, subacute and chronic axonal polyradiculoneuropathy in a tertiary hospital

#### D. pal ^1^; A. Pandit^2^; D. Mukherjee^3^; S. Dubey^4^

##### ^*1*^ *Neurology Department, Bangur Institute of Neurosciences, Kolkata, India;* ^*2*^ *Neurology Department, Bangur Institute of Neurosciences, Kolkata, India;* ^*3*^ *Neurology Department, R G Kar Hospital, Kolkata, India;* ^*4*^ *Neurology Department, Bangur Institute of Neurosciences, Kolkata, India*

## EPV‐1526

### Cognitive dysfunction and peripheral neuropathy among diabetic patients: Clinical features and contributing factors in conflict‐affected region

#### I. Hussein ^1^; M. Gasmalha^2^; Y. Fadlelmoula^3^; M. Abdulrahman^4^; A. Talha^5^; M. Ahmed^6^; I. Eltaeib^7^; H. Fadhl^8^; D. Ahmed^9^; A. Modawy^10^; A. Hassan^11^; N. Al Sheikh^12^; O. Hagtaha^13^; S. Ahmed^14^

##### ^*1*^ *Faculty of Pharmacy, University of Gadarif, Gadarif, Sudan;* ^*2*^ *Faculty of Medicine and Health Sciences, University of Omdurman Islamic, Omdurman, Sudan;* ^*3*^ *Faculty of Medicine, Gazira University, Wad Madani, Sudan;* ^*4*^ *Faculty of Medicine, Alzaiem Alazhari University, Khartoum, Sudan;* ^*5*^ *Faculty of Medicine and Health Sciences, University of Gadarif, Gadarif, Sudan;* ^*6*^ *Faculty of Medicine and Health Sciences, University of Gadarif, Gadarif, Sudan;* ^*7*^ *Faculty of Medicine and Health Sciences, University of Gadarif, Gadarif, Sudan;* ^*8*^ *Yemen, Aden University, Medical Education;* ^*9*^ *Faculty of pharmacy, University of AlMugtaribeen, Khartoum, Sudan;* ^*10*^ *Faculty of Pharmacy, Al Mughtaribeen University, Khartoum, Sudan;* ^*11*^ *University of Gezira, Faculty of Medicine, Wad Madani, Sudan;* ^*12*^ *Faculty of Pharmacy, University of AlMugtaribeen, Khartoum, Sudan;* ^*13*^ *Faculty of Medicine and Health Sciences, University of Bakht Al‐Ruda, Eldwium, Sudan;* ^*14*^ *Faculty of Medicine and Health Sciences, University of Gadarif, Gadarif, Sudan*

## EPV‐1527

### Comparative analysis of laser doppler flowmetry parameters in patients with severe and mild diabetic angiopolineuropathy

#### U. Mukarramov; S. Rustambekova; S. Kuranbayeva; G. Raximbaeva; S. Kalandarova

##### Ferghana Medical Institute of Public Health, Ferghana, Uzbekistan

## EPV‐1528

### Comparison of Asbury criteria versus Brighton collaboration criteria in early diagnosis of patients with Guillain‐Barre syndrome

#### A. Pai ^1^; N. Mukhi^1^; D. Varma^2^

##### ^*1*^ *Department of Neurology, Kasturba Medical College, Manipal Academy of Higher Education, Manipal, India;* ^*2*^ *Department of Infectious Diseases, Kasturba Medical College, Manipal Academy of Higher Education, Manipal, India*

## EPV‐1529

### De novo POLR3B mutation results in demyelinating polyneuropathy and spastic paraparesis with dental abnormalities

#### R. Manso Calderón ^1^; B. Garcia^2^

##### ^*1*^ *Neurology Department, Salamanca University Hospital Complex, Salamanca, Spain;* ^*2*^ *Genetic Department, Salamanca University Hospital Complex, Salamanca, Spain*

## EPV‐1530

### Diagnostic accuracy of ultrasound for carpal tunnel syndrome taking nerve conduction studies as gold standard: A meta‐analysis in Asian populations

#### F. Amin; A. Abid

##### King Edward Medical University, Lahore, Pakistan

## EPV‐1531

### Efficacy and safety of rTMS in peripheral neuropathic pain: A meta‐analysis of randomized sham‐controlled trials

#### O. Abdelsalam ^1^; R. Mohamed^2^; M. Mahmoud^3^; A. Dawoud^4^; R. Labib^5^; B. Hamed^6^; M. AbdelMeseh^7^; S. Hedawy^8^; M. Abouelmagd^9^; A. Abbas^10^; A. Raslan^11^

##### ^*1*^ *Faculty of Medicine, New Mansoura University, New Mansoura, Egypt;* ^*2*^ *Mansoura Manchester Program for Medical Education, Faculty of Medicine, Mansoura University, Mansoura, Egypt;* ^*3*^ *Faculty of medicine, Beni Suef university, Beni Suef, Egypt;* ^*4*^ *Faculty of Medicine Al‐Azher University Damietta, New Damietta, Egypt;* ^*5*^ *Faculty of medicine, Cairo university, Cairo, Egypt;* ^*6*^ *Faculty of Medicine Al‐Azhar cairo University;* ^*7*^ *Faculty of Medicine – Alexandria University;* ^*8*^ *Faculty of Medicine, Al‐Azhar Assiut University, Assiut, Egypt;* ^*9*^ *Faculty of Medicine, Cairo University, Cairo, Egypt;* ^*10*^ *Faculty of Medicine, Al‐Azhar University, Damietta, Egypt;* ^*11*^ *Department of Neurological Surgery, Oregon Health & Science University, Portland, USA*

## EPV‐1532

### Insights on neuralgic amyotrophy pathophysiology from an acute case

#### D. Sparasci ^1^; M. Calcagni^2^; C. Gobbi^1^; P. Ripellino^1^

##### ^*1*^ *Department of Neurology, Neurocenter of Southern Switzerland EOC, Ospedale Regionale di Lugano, Lugano, Switzerland;* ^*2*^ *Hand Institut, Zurich*, *Switzerland*

## EPV‐1533

### Juvenile myasthenia gravis: Clinical features, management, and outcomes

#### M. Mezhoud; H. Klaa; T. Ben Younes; Z. Miladi; A. Zyoudi; M. Jamoussi; H. Ben Rhouma; I. Ben Youssef Turki; I. Kraoua

##### 1‐LR18SP04 and Department of Pediatric Neurology, National Institute of Mongi Ben Hmida of Neurology, Tunis, Tunisia

## EPV‐1534

### Linezolid‐associated sensory axonal neuropathy: Longitudinal clinical observations with neurophysiological correlation

#### L. Kennelly; O. O'Toole

##### Department of Neurology, Cork University Hospital, Cork, Ireland

## EPV‐1535

### Nodopathy with autoantibodies against contactin‐1 and contactin‐associated protein‐1 complex associated with clear cell renal carcinoma

#### Ł. Rzepiński

##### Department of Neurology, 10th Military Research Hospital and Polyclinic, Bydgoszcz, Poland

## EPV‐1536

### Polineuropathy revealing rare TTR Ile127Val amyloidosis

#### O. Noea ^1^; L. Cucu^2^; C. Grosu^2^

##### ^*1*^ *Neurology, Clinical Rehabilitation Hospital, Iasi, Romania;* ^*2*^ *“Grigore T. Popa” University of Medicine and Pharmacy, Iasi, Romania*

## EPV‐1537

### Prevalence of Guillain–Barré syndrome subtypes in Pakistan: A meta‐analysis

#### A. Abid; F. Amin

##### King Edward Medical University, Lahore, Pakistan

## EPV‐1538

### Prognostic factors in Guillain–Barré syndrome: A retrospective cohort study in a tertiary referral centre

#### M. Russo; M. De Luca; A. Squillaci; A. Toscano; C. Rodolico; A. Mazzeo; S. Messina

##### Department of Clinical and Experimental Medicine, University of Messina, Messina, Italy

## EPV‐1539

### Risk factors for pregnancy‐related carpal tunnel syndrome

#### M. Vasilieva^1^; M. Junbei ^2^; M. Lisu^1^; S. Groppa^1^

##### ^*1*^ *Institute of Emergency Medicine, Clinical Department of Neurology, Epileptology and Internal Disease, Chisinau, Republic of Moldova;* ^*2*^ *Institute of Emergency Medicine, Clinical Department of Traumatology of Neurology, Epileptology and Internal Disease, Chisinau, Republic of Moldova*

## EPV‐1540

### Role of upper extremity mixed nerve conduction studies in the electrophysiological diagnosis of chronic inflammatory demyelinating polyneuropathy

#### K. Keskin ^1^; E. Gözke^2^

##### ^*1*^ *Neurology, Silvan Dr. Yusuf Azizoglu State Hospital, Diyarbakır, Türkiye;* ^*2*^ *Neurology, University of Health Sciences Fatih Sultan Mehmet Training and Research Hospital, Istanbul*, *Türkiye*

## EPV‐1541

### Seasonal variation and clinical severity of Guillain–Barré syndrome: A five‐year single‐center experience

#### N. Mahmutbegovic; H. Jatic; N. Masic; E. Hasanbegovic; S. Sabanagic Hajric; S. Drnda; E. Mehmedika Suljic

##### Neurology Clinic, Clinical Center of Sarajevo University, Sarajevo, Bosnia and Herzegovina

## EPV‐1542

### Serum neurofilament light chain as a biomarker in immune‐mediated and non‐inflammatory polyneuropathies

#### Z. Elmas; S. Halbgebauer; C. Herrmann; F. Bachhuber; Ö. Soylu; H. Tumani; J. Dorst

##### Department of Neurology, University Hospital of Ulm, Ulm, Germany

## EPV‐1543

### Abstract withdrawn

## EPV‐1544

### Unilateral peripheral facial paralysis in Guillain‐Barre syndrome: A single‐center study

#### S. Muradkhanova

##### Ondokuz Mayıs University, Samsun, Türkiye

## EPV‐1545

### Unmasking bilateral Parsonage–Turner syndrome: The critical role of neuroimaging

#### J. Oliveira ^1^; C. Santos Silva^1,2^; D. Tribovane^3^; L. Pereira^1^; C. Alves^1^; M. Grunho^1,4^

##### ^*1*^ *Department of Neurology, Hospital Garcia de Orta, Unidade Local de Saúde Almada–Seixal, Almada, Portugal;* ^*2*^ *Faculty of Medicine, Institute of Molecular Medicine, University of Lisbon, Lisbon, Portugal;* ^*3*^ *Department of Neuroradiology, Hospital Garcia de Orta, Unidade Local de Saúde Almada–Seixal, Almada, Portugal;* ^*4*^ *Egas Moniz Center for Interdisciplinary Research (CiiEM), Egas Moniz School of Health Science, Caparica, Almada, Portugal*

## Sleep‐wake disorders

## EPV‐1546

### “Dark Ain't That Dark”: A qualitative study exploring biopsychosocial barriers and facilitators in the everyday lives of patients with RLS

#### E. Odzakovic ^1^; S. Öberg^1^; M. Jakobsson^1^; M. Björk^1^; M. Georgsson^1^; S. Knutsson^2^; B. Fridlund^3^; L. Jonasson^1^; M. Ulander^4^; J. Lind^5^; A. Broström^1^

##### ^*1*^ *School of Health and Welfare, Jönköping University, Jönköping, Sweden;* ^*2*^ *Department of Health and Caring Sciences, Faculty of Health and Life Sciences, Linnaeus University, Växjö, Sweden;* ^*3*^ *Centre for Interprofessional Collaboration within Emergency Care (CICE), Linnaeus University, Växjö, Sweden;* ^*4*^ *Department of Clinical Neurophysiology, Linköping University Hospital, Linköping, Sweden; Department of Biomedical and Clinical Sciences, Division of Neurobiology; Linköping University, Linköping, Sweden;* ^*5*^ *Section of Neurology, Department of Internal Medicine, County Hospital Ryhov, Jönköping, Sweden*

## EPV‐1547

### “We are family”: Experiences of social support, focusing on facilitators and barriers, among family members of patients with RLS

#### E. Odzakovic ^1^; M. Georgsson^1^; M. Jakobsson^1^; S. Öberg^1^; M. Björk^1^; L. Jonasson^1^; S. Knutsson^2^; M. Ulanders^3^; B. Fridlund^4^; A. Broström^1^

##### ^*1*^ *School of Health and Welfare, Jönköping University, Jönköping, Sweden;* ^*2*^ *Department of Health and Caring Sciences, Faculty of Health and Life Sciences, Linnaeus University, Växjö, Sweden;* ^*3*^ *Department of Clinical Neurophysiology, Linköping University Hospital, Linköping, Sweden. Department of Biomedical and Clinical Sciences, Division of Neurobiology. Linköping University, Linköping, Sweden;* ^*4*^ *Centre for Interprofessional Collaboration within Emergency Care (CICE), Linnaeus University, Växjö, Sweden*

## EPV‐1548

### Assessment of the impact of psycho‐emotional disorders on the sleep onset process and determination of the effectiveness of relaxation psychotherapy

#### G. Ernayeva; Z. Ibodullaev; N. Maxamatjanova; S. Qaraxanova; Z. Xosilovna

##### Neurology and Medical Psychology Department, Tashkent State Medical University, Tashkent, Uzbekistan

## EPV‐1549

### Association between neck myoclonus and spontaneous cervical artery dissection: A case‐control study

#### Q. Tang ^1^; A. Ibrahim^1^; B. Dejakum^1^; E. Holzknecht^1^; E. Brandauer^1^; A. Karisik^1^; M. Aktan Süzgün^1^; B. Frauscher^2^; M. Knoflach^1^; S. Kiechl^1^; B. Högl^1^; A. Stefani^1^; L. Mayer‐Suess^1^

##### ^*1*^ *Department of Neurology, Medical University Innsbruck, Austria;* ^*2*^ *Department of Neurology, Duke University Medical Center, Durham, USA; Department of Biomedical Engineering, Duke Pratt School of Engineering, Durham, USA*

## EPV‐1550

### Clinical structure of chronic insomnia in neurotic disorders and its psychocorrection

#### S. Dushamov ^1^; B. Ibadullayev^1^; I. Abdullayev^1^; D. Kamilova^1^; O. Qadamova^1^

##### ^*1*^ *Urgench State Medical Institute, Urgench, Uzbekistan*

## EPV‐1551

### Efficacy and safety of transcranial alternating current stimulation in treating Chinese patients with primary insomnia: A meta‐Analysis

#### S. Wasti ^1^; A. Wasti^2^; A. Soejono^3^

##### ^*1*^ *Department of Neurology, King Edward Medical University, Lahore, Pakistan;* ^*2*^ *Department of Neurology, Continental Medical College, Lahore, Pakistan;* ^*3*^ *Department of Neurology, Universitas Indonesia, Jakarta, Indonesia*

## EPV‐1552

### Evaluation of body mass index and HDL/Triglyceride ratio in patients diagnosed with OSAS: A retrospective cohort study

#### I. Kalyoncu Aslan; B. Karagöz; Ö. Soydemir

##### *Fatih Sultan Mehmet Education and Research Hospital,* Istanbul; Türkiye

## EPV‐1553

### Focusing on minor parasomnia events: Is delta sleep combined with open eyes enough to diagnose disorders of arousal?

#### E. Balian ^1^; L. Atabekyan^1^; E. Gevorgyan^2^; H. Hovakimyan^1^; S. Khachatryan^2^

##### ^*1*^ *Somnus Neurology Clinic, Yerevan, Armenia;* ^*2*^ *Department of Neurology and Neurosurgery, National Institute of Health, Yerevan, Armenia*

## EPV‐1554

### Impact of circadian rhythm correction on treatment‐refractory migraine in night‐shift workers: A wearable‐based pilot study

#### K. Park

##### Wiltse Memorial Hospital, Suwon, Republic of Korea

## EPV‐1555

### Informational needs among patients with restless legs syndrome (RLS): Psychometric testing of the informational needs of RLS inventory

#### E. Odzakovic ^1^; A. Pakpour^1^; M. Ulander^2^; S. Öberg^1^; M. Björk^1^; B. Fridlund^3^; C. Sandlund^4^; H. Bruhn^5^; A. Hellström^6^; A. Broström^1^

##### ^*1*^ *Department of Nursing, School of Health and Welfare, Jönköping University, Jönköping, Sweden;* ^*2*^ *Department of Biomedical and Clinical Sciences, Linköping University, Linköping, Sweden;* ^*3*^ *Department of Clinical Neurophysiology, Linköping University Hospital, Linköping, Sweden; Department of Clinical Neurophysiology, Linköping University Hospital, Li, Sweden;* ^*3*^ *Centre for Interprofessional Collaboration within Emergency Care (CICE), Linnaeus University, Växjö, Sweden;* ^*4*^ *Department of Neurobiology, Care Sciences and Society, Karolinska Institute, Stockholm, Sweden; Academic Primary Health Care Centre, Region Stockholm, Stockholm, Sweden;* ^*5*^ *Section of Neurology, Department of Internal Medicine, County Hospital Ryhov, Jönköping, Sweden;* ^*6*^ *Department of Health and Caring Sciences, Faculty of Health and Life Sciences, Linnaeus University, Kalmar, Sweden; Academic Primary Care, Öland, Kalmar county, Sweden*

## EPV‐1556

### Insomnia among hospitalized inpatients: Old issues, new challenges

#### D. Bottignole ^1^; G. Balella^1^; M. Minetti^1^; L. Gambolò^2^; F. Rausa^3^; G. Ughetti^4^; A. Melpignano^3^; M. Maggio^5^; L. Parrino^4^; C. Mutti^3^

##### ^*1*^ *Neurology Unit, Department of Medicine and Surgery, University of Parma, Parma, Italy;* ^*2*^ *Psychiatric Unit, Local Health Agency of Piacenza, Piacenza, Italy;* ^*3*^ *Sleep Disorders Center, Department of General and Specialized Medicine, University Hospital of Parma, Parma, Italy;* ^*4*^ *“Mario Giovanni Terzano” Interdepartmental Center for Sleep Medicine, Department of Medicine and Surgery, University of Parma, Parma, Italy;* ^*5*^ *Geriatric Clinic Unit, Geriatric‐Rehabilitation Department, University Hospital of Parma, Parma, Italy*

## EPV‐1557

### Neurobehavioral and autonomic correlates of sleepiness severity in central disorders of hypersomnolence

#### S. Mombelli^1^; C. Leitner^2^; F. Bolengo^2^; L. Dell'Acqua^2^; A. Frijo^2^; F. Casoni^2^; P. Proserpio^2^; C. Liguori^3^; A. Galbiati^2^; L. Ferini‐Strambi ^2^

##### ^*1*^ *Center for Advanced Research in Sleep Medicine, Research Center of the Centre Intégré Universitaire de Santé et de Services Sociaux du Nord de l’Île‐de‐Montréal, Montreal, Canada, Montréal, Canada;* ^*2*^ *Sleep Disorders Centre, Department of Clinical Neurosciences, Neurology, IRCCS San Raffaele Scientific Institute, Milan, Italy;* ^*3*^ *Sleep Medicine Center, Department of Systems Medicine, University of Rome Tor Vergata, Rome, Italy*

## EPV‐1558

### Predictive value of the easy sleep apnea predictor in asymptomatic driver's license applicants with increased body mass index

#### B. Zeybek; U. Akyıldız

##### Neurology, Aydın Adnan Menderes University Hospital, Türkiye

## EPV‐1559

### Sleep characteristics and associated factors in healthcare workers: Findings from a nationwide population‐based study

#### E. Yakupov ^1^; A. Mardiev^1^; S. Agliullina^2^; Y. Troshina^1^

##### ^*1*^ *Professor Yakupov's Scientific Research Medical Center, Kazan, Russian Federation;* ^*2*^ *Kazan State Medical University, Ministry of Health of the Russian Federation, Kazan, Russian Federation*

## EPV‐1560

### Sleep in post‐traumatic epilepsy

#### E. Hayrapetyan ^1^; E. Balian^1^; L. Atabekyan^1^; H. Amroyan^2^; S. Khachatryan^2^

##### ^*1*^ *Somnus Neurology Clinic, Yerevan, Armenia;* ^*2*^ *National Institute of Health, Yerevan, Armenia*

## EPV‐1561

### Sleep quality in a series of people with multiple sclerosis

#### D. García Estévez ^1^; O. Álvarez de los Arcos^2^

##### ^*1*^ *Neurology Service, Complejo Hospitalario Universitario de Ourense, Ourense, Spain;* ^*2*^ *Clinical Neuroscience Group, Galicia Sur Health Research Institute (IIS Galicia Sur), Vigo, Spain*

## EPV‐1562

### Somatic symptoms and sleep quality in Ukrainian medical students during wartime stress

#### A. Yevtushenko ^1^; V. Kokashynskyi^1^; O. Onyshchenko^2^

##### ^*1*^ *Dnipro State Medical University*, *Dnipro, Ukraine;* ^*2*^ *Bogomolets National Medical University*, *Ukraine*

## EPV‐1563

### The effect of hand‐wrist pain consistent with carpal tunnel syndrome on sleep quality among employees at Pamukkale

#### N. Ergin ^1^; Gökdeniz^2^; A. Ergin^2^

##### ^*1*^ *Department of Neurology, Faculty of Medicine, Pamukkale University*, *Denizli, Türkiye;* ^*2*^ *Department of Public Health, Faculty of Medicine, Pamukkale University*, *Denizli, Türkiye*

## EPV‐1564

### The relationship between ALPS‐index and motor and domain‐specific cognitive functions in de novo Parkinson's disease

#### V. Rottova ^1^; S. Marecek^1^; T. Krajca^2^; J. Keller^3^; K. Sonka^1^; P. Dusek^1^; O. Bezdicek^1^; J. Nepozitek^1^

##### ^*1*^ *Department of Neurology and Center of Clinical Neuroscience, First Faculty of Medicine, Charles University and General University Hospital in Prague, Czech Republic;* ^*2*^ *Faculty of Biomedical Engineering, Czech Technical University in Prague, Kladno, Czech Republic;* ^*3*^ *Radiodiagnostic Department, Na Homolce Hospital, Prague, Czech Republic*

## Spinal cord and root disorders

## EPV‐1565

### Acute myeloradiculitis unveiling hepatitis B virus infection: Case report

#### K. Chekili ^1^; H. Derbali^1^; I. Bedoui^1^; Y. Mrad^1^; M. Messelmani^1^; M. Mansour^1^; N. Fekih Mrissa^2^; J. Zaouali^1^

##### ^*1*^ *Department of Neurology, Military Hospital of Tunis, Tunisia;* ^*2*^ *Molecular Biology Unit (UR17DN06), Laboratory of Hematology, Military Hospital of Tunis, Tunisia*

## EPV‐1566

### Beyond the usual: Spondylodiscitis as a hidden cause of L5 radiculopathy

#### A. Morales Pérez; G. Lafuente Gómez; D. Vargas Macías; D. Machado Sampedro; Á. Rodríguez Salvador; A. Bonilla Tena; I. Lera Ramírez; R. Olaizola Díaz; O. Úriz Bacaicoa; R. Leal Hidalgo; F. Grandas Pérez

##### Department of Neurology, Hospital General Universitario Gregorio Marañón, Madrid, Spain

## EPV‐1567

### Chronic inflammatory sensory polyradiculopathy: A case report

#### I. Radu ^1^; A. Stefanache^1^; A. Iacob^1^; D. Chitimus^1^; A. Manole^1^; S. Dumitrescu^1^; C. Sirbu^2^

##### ^*1*^ *Department of Neurology, ‘Dr. Carol Davila’ Central Military Emergency University Hospital, Bucharest, Romania;* ^*2*^ *Academy of Romanian Scientists, Bucharest, Romania*

## EPV‐1568

### Early presentations of multiple sclerosis: Two adult cases

#### L. Namazova

##### Silcmedical, Tbilisi, Georgia

## EPV‐1569

### International standards to document autonomic function after spinal cord injury: Third edition

#### E. Hagen

##### Department of Clinical Medicine, Aarhus University, Denmark

## EPV‐1570

### Learning from the two spinal cord infarct cases missed in initial diagnoses

#### K. Lwin ^2^; S. Tun^1^; A. Thet^1^; Z. Tun^1^; T. Thiri^2^; M. Thura^2^

##### ^*1*^ *Department of Neurophysiology, Pinderfields General Hospital, Wakefield, UK;* ^*2*^ *Department of Neurophysiology, Calderdale Royal Hospital, Halifax, UK*

## EPV‐1571

### Neurogenic Bladder in Children with Myelomeningocele

#### A. Sic ^1^; B. Stojanovic^2^; M. Djordjevic^3^

##### ^*1*^ *Faculty of Medicine, University of Belgrade, Belgrade, Serbia;* ^*2*^ *Department of Urology, University Children's Hospital, Belgrade, Serbia;* ^*3*^ *Department of Urology, Icahn School of Medicine at Mount Sinai, New York, USA*

## EPV‐1572

### Spinal dural arteriovenous fistula: A crucial differential diagnosis in myelopathy

#### C. Segura Sanz; L. Rivas

##### *Department of Neurology, University Hospital Virgen de la Victoria*, *Málaga, Spain*

## EPV‐1573

### Uncommon case of methotrexate‐induced myelopathy

#### C. Yip; K. Ng; S. Lim; Z. Chan

##### National Neuroscience Institute (SGH Campus), Singapore

## EPV‐1574

### Use of neural implants and brain‐computer interfaces in patients with spinal cord paralysis

#### A. Bovanegra Arriyo; L. García Kornhauser; M. Sierra Retes; L. Balandran Villalpando

##### Universidad de Guanajuato División Ciencias de la Salud Licenciatura Médico Cirujano Leon Guanajuato, Mexico

## EPV‐1575

### When a benign lesion becomes aggressive: Vertebral hemangioma

#### E. Gismera Fontes; M. González Sánchez; F. Sánchez García; M. González Gómez; L. Cabral Esquea

##### Neurology Department, University Hospital Guadalajara, Guadalajara, Spain

